# Discovery of new potent lysine specific histone demythelase-1 inhibitors (LSD-1) using structure based and ligand based molecular modelling and machine learning†

**DOI:** 10.1039/d2ra05102h

**Published:** 2022-12-15

**Authors:** Shada J. Alabed, Malek Zihlif, Mutasem Taha

**Affiliations:** Department of Pharmacy, Faculty of Pharmacy, Al-Zaytoonah University of Jordan Amman Jordan Shada_alabed@yahoo.com; Department of Pharmacology, Faculty of Medicine, University of Jordan Amman Jordan m.zihlif@ju.edu.jo; Department of Pharmaceutical Sciences, Faculty of Pharmacy, University of Jordan Amman Jordan mutasem@ju.edu.jo

## Abstract

Lysine-specific histone demethylase 1 (LSD-1) is an epigenetic enzyme that oxidatively cleaves methyl groups from monomethyl and dimethyl Lys4 of histone H3 and is highly overexpressed in different types of cancer. Therefore, it has been widely recognized as a promising therapeutic target for cancer therapy. Towards this end, we employed various Computer Aided Drug Design (CADD) approaches including pharmacophore modelling and machine learning. Pharmacophores generated by structure-based (SB) (either crystallographic-based or docking-based) and ligand-based (LB) (either supervised or unsupervised) modelling methods were allowed to compete within the context of genetic algorithm/machine learning and were assessed by Shapley additive explanation values (SHAP) to end up with three successful pharmacophores that were used to screen the National Cancer Institute (NCI) database. Seventy-five NCI hits were tested for their LSD-1 inhibitory properties against neuroblastoma SH-SY5Y cells, pancreatic carcinoma Panc-1 cells, glioblastoma U-87 MG cells and in *vitro* enzymatic assay, culminating in 3 nanomolar LSD-1 inhibitors of novel chemotypes.

## Abstract 2

Structure-based and ligand-based pharmacophores were allowed to compete within genetic algorithm/machine learning to yield three pharmacophores. Subsequent virtual screening identified three nanomolar LSD-1 inhibitors.

## Graphical abstract

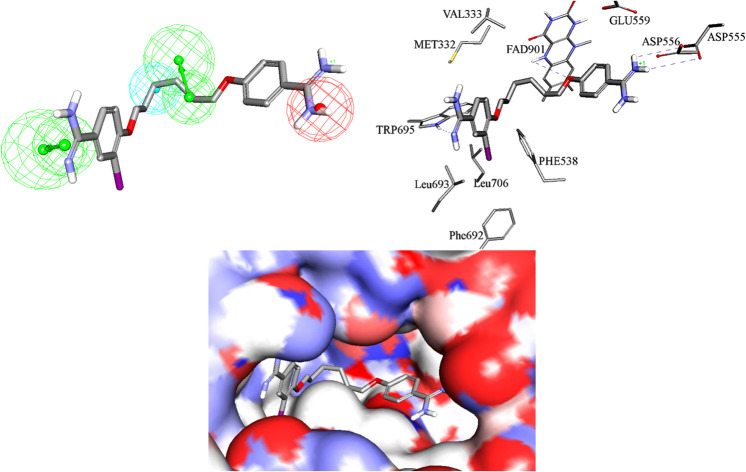

## 1. Introduction

“Epigenetics” are inheritable changes in gene expression with no alterations in DNA sequences, which are sufficiently powerful to regulate the dynamics of gene expression.^1–3^ Covalent modifications of histones can regulate almost all DNA-dependent processes. Recently, it has become more evident that histone modifications, which are controlled by an array of histone modifiers and chromatin-bound proteins, are crucial players in the regulation of transcription activation and repression.^4^ A balance between specific modifications and modifiers must be maintained at the steady state of the cell to maintain the chromatin structure and proper gene expression program. Once this balance is disrupted, cell phenotypes may be altered to allow disease onset and progression.^4^

Histone methylation involves the attachment of methyl groups to nitrogen atoms in amino acid side chains and/or at the amino termini of various residues.^4,5^ This process influences gene activity depending on the modified residues, degree and pattern of methylation, and the genomic context of methylation.^6,7^ Histone methylation was believed to be a stable, inheritable and irreversible process until the identification of FAD (flavin adenine dinucleotide)-dependent nuclear amine oxidase lysine specific demethylase 1 (LSD-1 or KDM1A).^8^ LSD-1 can demethylate mono- and di-methylated Lys4 or Lys9 on histone H3 under diverse biological settings using FAD as a cofactor and O_2_ as electron acceptor.^9^

The pivotal role of LSD-1 in numerous physiological cellular processes, including control of stemness, differentiation, cell motility, epithelial-to-mesenchymal transition and metabolism is well known and studied.^10^ Nevertheless, a large number of studies have highlighted the association between LSD-1 and cancer. It has been found that LSD-1 is involved in several cancers, including prostate, bladder, lung cancers, neuroblastoma, sarcomas and hepatocarcinomas.^11^ High expression levels of LSD-1 in cancer cells suggested LSD-1 as a druggable target for cancer treatment. Several LSD-1 inhibitors have been explored, with some of these inhibitors are in clinical trials as potential anti-cancer therapies.^12^

Computer-Aided Drug Design (CADD) is a widely used approach in the pharmaceutical industry and academia to accelerate development of new drug entities. CADD offers significant reduction in the cost and development time of drug design and discovery research and development.^13–15^ Recently, Wenchao Lu *et al.*, reviewed the computational approaches employed in drug discovery of new epigenitic inhibitors (epi-drugs), highlighting the importance of CADD in the discovery of new inhibitors in this particular field.^16^

**Fig. 1:**
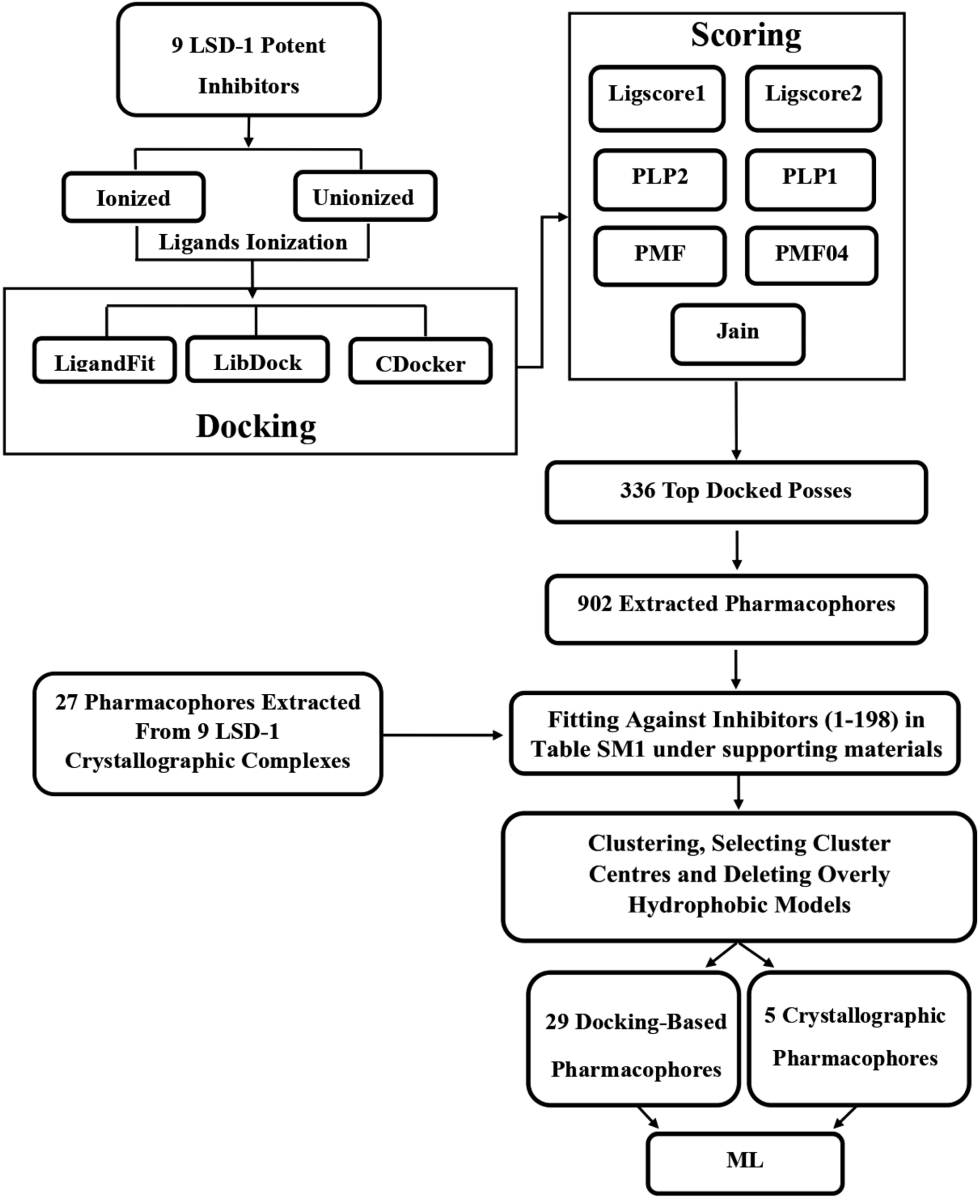
Computational workflow for structure-based pharmacophore modelling.

**Fig. 2:**
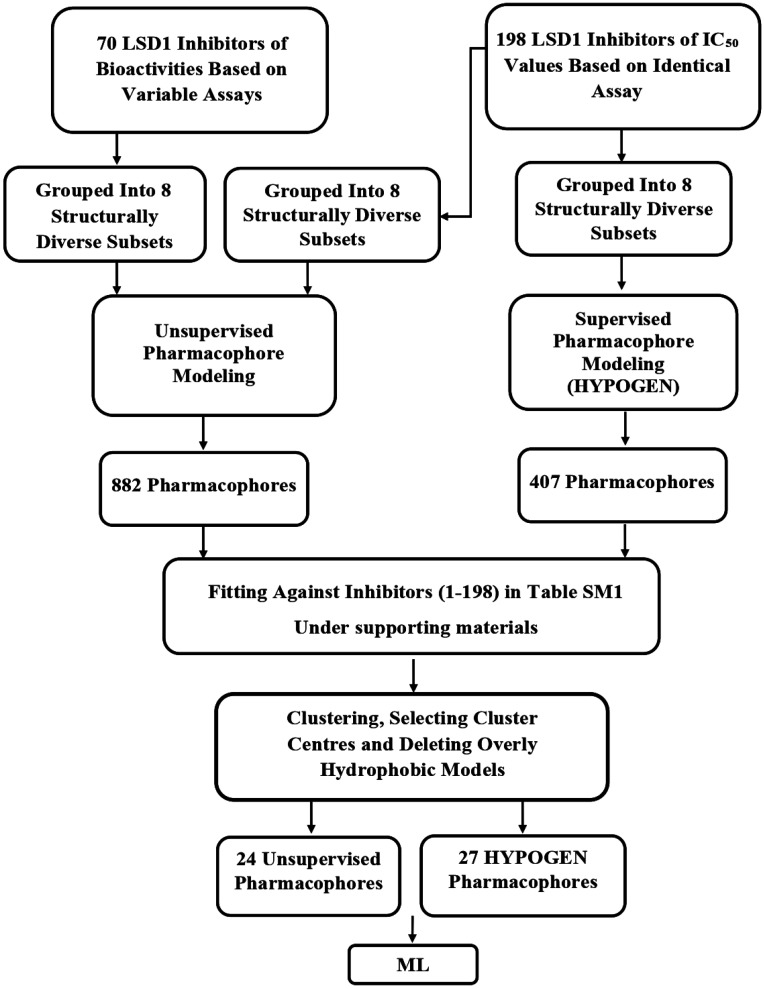
Computational workflow for ligand-based pharmacophore modelling.

**Fig. 3:**
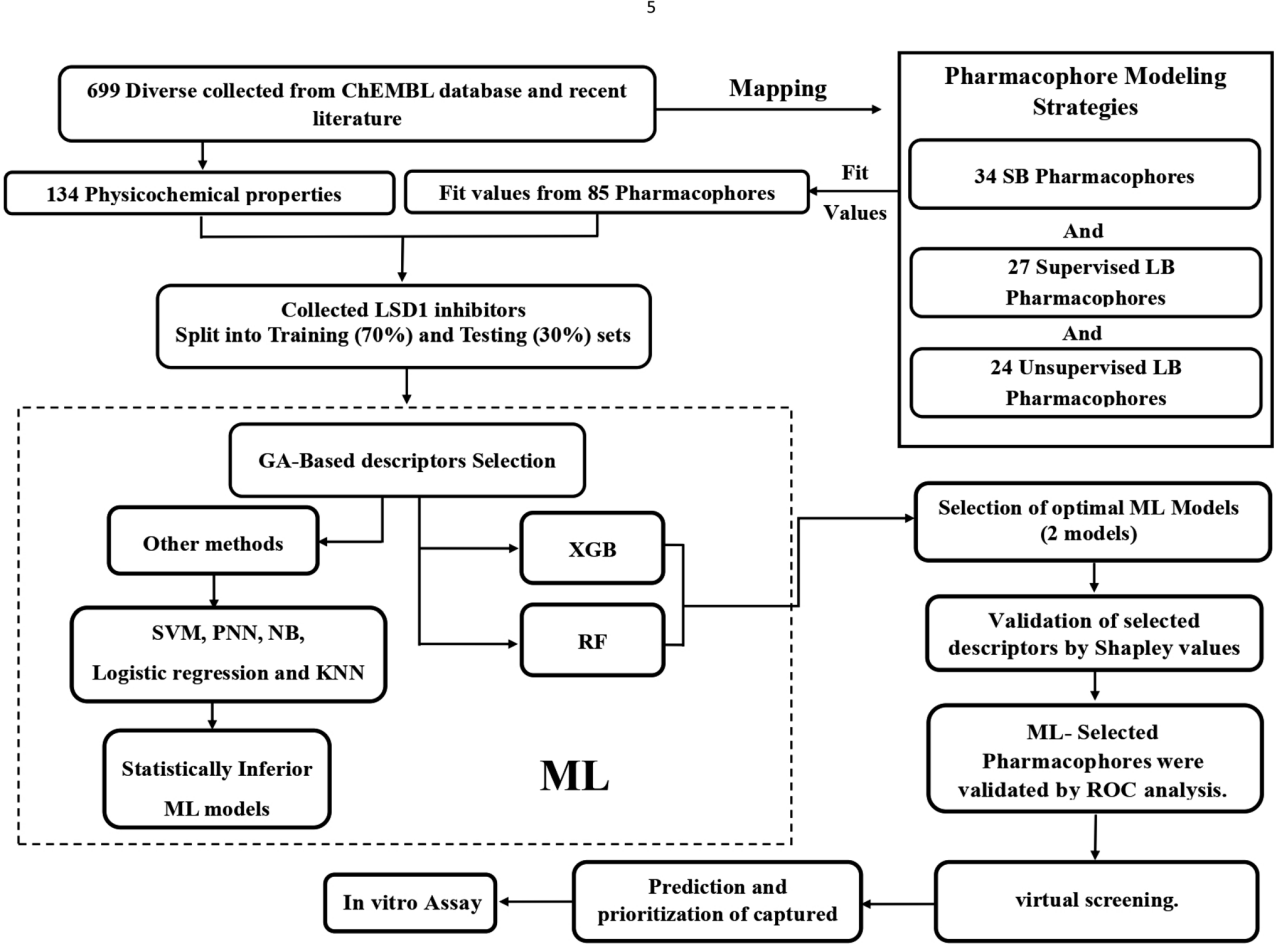
Machine learning workflow.

A variety of CADD techniques were recently explored to identify new LSD-1 modulating compounds and elucidate their binding modes, including molecular dynamic simulation, three-dimensional quantitative structure–activity relationships (3D-QSAR) and pharmacophore modelling.^17–20^ Despite these efforts, there are still no approved LSD-1 inhibitors in the clinical practice until now.^21^ The continued interest in LSD-1 prompted us to combine our innovative pharmacophore modelling methods^39,40,42,51,55,59–63,65,66,69,73,74,76,96,101,105,106,108,109,112^ with machine learning methodologies towards the discovery of new LSD-1 inhibitors. The fundamental novelty of the current project lies in allowing numerous pharmacophore models (of ligand- and structure-based origins) and physicochemical descriptors to compete within genetic algorithm (GA)/machine learning (ML) context for the selection of optimal combination of pharmacophore(s) and physicochemical descriptors within self-consistent and predictive ML model(s). The resulting ML models were judged based on their abilities to explain bioactivity variation within a long list of LSD-1 inhibitors. Optimal ML models and associated pharmacophores were then used to mine the national cancer institute (NCI) list of compounds for novel inhibitory hits and predict their anti-LSD-1 bioactivities. High-ranking hits were bioassayed for their anti-LSD-1 IC_50_ values. Fig. 1 and 2 show the computational workflows of structure-based (SB) and ligand-based (LB) pharmacophore modelling methods, while Fig. 3 shows the overall ML computational workflow.

## 2. Material and methods

### 2.1. Software

The following software packages were utilized in this research:

BIOVIA DiscoveryStudio (Version 4.5), Biovia Inc (https://www.3dsbiovia.com), USA.

KNIME Analytics Platform (Version 4.3.2), https://www.knime.com/.

CS ChemDraw Ultra (Version 11.0), Cambridge Soft Corp. (https://www.cambridgesoft.com), USA.

GraphPad Prism (Version 8), https://www.graphpad.com/.

Discovery studio was used for docking, ligand-based and structure-based pharmacophore modelling and descriptor calculations. On the other hand, all aspects related to machine learning, including genetic selection of descriptors (including pharmacophores), training and deployment of different learners (*i.e.*, random forests and XgBoost), as well as model evaluation *via* accuracy, Cohen's kappa and SHAP analysis, were done using graphical programming of KNIME analytics platform.

### 2.2. Data collection for exploring the pharmacophore space of LSD-1 inhibitors

The European bioinformatics institute database (ChEMBL) (https://www.ebi.ac.uk/chembl/) and recent published literature were searched for known LSD-1 inhibitors. The search identified 714 LSD-1 inhibitors, out of which 43 were reported to have experimental Ki values, and 622 were reported to have experimental IC_50_ values. The bioactivities of the rest (49 compounds) were reported as LSD-1 inhibition percentage at certain concentrations (ranging from 0.1 to 5 μM). One hundred ninety-eight LSD-1 inhibitors were bio-assayed by an identical assay method and were used for supervised/unsupervised and ligand/structure-based pharmacophore modelling. Eighty-three were reported to be bioassayed employing a multitude of assay methods and thus were only used for unsupervised pharmacophore modelling. ESI Table SM1† shows the collected list of compounds (1–281) of which 1–198 were of consistent bioassays, and 199–281 were discretely bioassayed together with their reported Ki or IC_50_ values (expressed in μM).^18,19,22–36^

### 2.3. Structure-based pharmacophore modelling

Structure-based pharmacophore modelling depends on interactions within ligand/receptor complexes to extract binding features and arrange them in 3D context (*i.e.*, pharmacophore model(s)).^37–39^ In addition to crystal structures, which are the main source of structural information,^40^ virtual complexes obtained by docking potent ligands into corresponding receptors were also employed to extract valid structure-based pharmacophores.^40,41^

In this approach, a small, diverse set of potent molecules are either co-crystallized within the target protein or docked into the binding site of the target. The resulting experimental or virtual ligand–receptor complexes are then used to extract pharmacophore models based on binding interactions perceived from the complexes using the “Ligand-Receptor Pharmacophore Generation” protocol within Discovery Studio. The resulting pharmacophores were allowed herein to compete with other ligand-based pharmacophores within GA/ML context to identify optimal pharmacophore(s) that can best explain bioactivity classifications of a list of LSD-1 inhibitors. Fig. 1 summarizes the computational workflow for structure-based pharmacophore modelling.

#### 2.3.1. Crystallographic complex-based pharmacophore modelling

The Protein Data Bank (PDB) (https://www.rcsb.org/pdb) was searched for LSD-1 crystal structures and nine were identified, downloaded and used to extract 27 pharmacophore models using the “Receptor-Ligand Pharmacophore Generation” protocol in Discovery Studio (version 4.5).^42^ The PDB codes, resolutions, chemical structures and bioactivities of complexed ligands of the collected LSD-1 crystal structures are shown in ESI Table SM2.†^24,36,43,44^

#### 2.3.2. Docking-based pharmacophore modelling

Molecularly diverse potent LSD-1 inhibitors (199, 206, 208, 224, 230, 248, 256, 270 and 276 Ki or IC_50_ ≤ 200 nM, ESI Table SM1†) were selected to represent potent LSD-1 ligands as described in ESI Section SM1 and Fig. SM1.† The selected compounds were docked into LSD-1 (PDB code: 5LHI, resolution = 3.40 Å). This particular crystallographic structure (*i.e.*, 5LHI) was selected for the docking study because it showcases one of the largest co-crystallized ligands (*i.e.*, in molecular weight) among all crystallographic LSD-1 structures, hence it should have relatively large binding pocket. A spacious binding site should allow more diverse docking solutions, which enhances the likelihood of unveiling realistic binding modes among docked poses. Moreover, the complexed ligand in 5LHI is the most potent among other complexed ligands in other LSD1 crystallographic structures (IC_50_ = 7.8 nM, as in ESI Table SM2†), therefore we assume that it will more effectively imprint critical binding features within the binding site and increase the chance of revealing realistic binding modes among docked poses. The selected compounds were docked in their ionized and unionized states using three docking engines, namely, LibDock,^45^ LigandFit^46^ and CDOCKER.^47^ The highest-ranking docked conformers/poses were scored using seven scoring functions: Jain,^48^ LigScore1, LigScore2,^46^ PLP1, PLP2,^49^ PMF and PMF04.^50^ The resultant 336 virtual LSD-1 ([7 ionizable compounds × 42 docked poses] + [2 non-ionizable compounds × 21 docked poses] = 336) complexes were imported into Discovery Studio. Subsequently, the Receptor-Ligand Pharmacophore Generation Protocol of Discovery Studio was used to extract a maximum of 10 pharmacophore models from each docked pose. Full details of the docking, scoring, pharmacophore generation, clustering and representative pharmacophores features with success criteria are described in ESI Sections SM2–SM4 and table SM3.†^45–54^

### 2.4. Ligand-based pharmacophore modelling

Ligand-based pharmacophore modelling aims to identify common chemical features among known active compounds irrespective to binding site details.^55^ The binding features are then assembled in a three-dimensional (3D) binding model, *i.e.*, pharmacophore, to represent a hypothetical binding site. The resulting pharmacophore(s) should not fit inactive compounds or, in a worst case scenario, fits only few inactive compounds.^56,57^ The most important step to have a successful ligand-based model with an excellent discriminatory predictive power is the selection of reasonable training set(s).^58^ In this context, ligand-based pharmacophore modelling can either proceed guided by the bioactivities of training compounds (*i.e.*, supervised modelling) or guided by commonalities of binding features among training compounds (*i.e.*, unsupervised modelling).^59^ In this project, we relied on the HYPOGEN module of Discovery Studio software suite (version 4.5) for supervised pharmacophore modelling.^60^ Moreover, it was decided to use the “Common Feature Pharmacophore Generation” (also known as HIPHOP) protocol within Discovery Studio for unsupervised ligand-based pharmacophore modelling, *i.e.*, to identify common binding features among potent ligands independent of their bioactivities,^59^ which allowed us to incorporate additional training compounds irrespective of their bioassay conditions. The computational workflow for ligand-based pharmacophore exploration is summarized in Fig. 2.

#### 2.4.1. Supervised ligand-based pharmacophore modelling

The collected inhibitors (1–198, ESI Table SM1†) ^18,19,27–29,32^ were categorized into 8 diverse training subsets (subsets A, B, C, D, E, F, G and H in ESI Table SM4†). Training compounds in each subset were carefully selected to conform to certain envisaged binding mode assumed by ligands in LSD-1 binding site. These subsets were used to explore the pharmacophoric space of LSD-1 inhibitors over 64 automatic HYPOGEN runs through well-established steps within our group^28,39,40,49,60–68,70,71,76^ described in detail in ESI Sections SM5–SM8 and Tables SM4–SM6.†

#### 2.4.2. Unsupervised ligand-based pharmacophore modelling

Training compounds for unsupervised pharmacophore modelling need not to be bioassayed by the same procedure.^39,59,62–64^ Therefore, the pharmacophoric space of LSD-1 inhibitors was explored through 16 carefully selected training subsets (ESI Table SM7†) from the collected inhibitors (1–281, bioassayed by a variety of procedures as in ESI Table SM1†). Each subset was used to conduct 12 automatic runs (ESI Table SM8†). The training subsets were selected such that each subset represents a particular theoretical binding mode. Subsequent pharmacophore exploration translated the proposed binding modes into corresponding pharmacophore models. Unsupervised pharmacophore modelling was conducted using “Common Feature Pharmacophore Generation” protocol within Discovery Studio 4.5 through established modelling steps within our group as described in ESI Sections SM9 and SM10 and Tables SM7–SM9.†^39,59,62–64^

### 2.5. Machine learning guided selection of pharmacophores models

#### 2.5.1. Preparation of training and testing sets

Searching the ChEMBL database along with recent published literature identified 714 LSD-1 inhibitors, out of which 43 were reported to have experimental Ki values and 622 were reported to have experimental IC_50_ values. The bioactivities of the remainder (49 compounds) were reported as percent inhibition at a variety of concentrations (ranging from 0.1 to 10 μM). The compound list used in this study was prepared by deleting duplicated molecules and molecules that have their bioactivities reported as being more (>) or less (<) than certain values (*i.e.*, only compounds that have their bioactivities reported to be equal “ = ” to certain values were retained). Additionally, compounds that have their anti-LSD-1 bioactivities reported as percentage inhibition at certain concentration were generally excluded unless they were explicitly stated as being “inactive” or have their bioactivities reported to be <5%. Subsequently, activity cliff pairs (identified as being similar compounds of bioactivity difference ≥ 2 logarithmic folds using the “Find Activity Cliffs” protocol implemented in Discovery Studio 4.5 (Biovia Inc., USA)) were excluded from the compound list, yielding 699 compounds. These were divided into three classes based on their anti-LSD-1 bioactivities: (i) actives of IC_50_ or Ki <6000 nM, (ii) inactives of IC_50_ or Ki ≥ 125 000 nM or those of percent inhibition <5%, or compounds explicitly labelled as being “inactive” in ChEMBL database, (iii) moderately active of IC_50_ or Ki ranging from 6000 nM to 125 000 nM.

The refined list (699 compounds) was divided into training and testing sets using the Generate Training and Test Data protocol in Discovery Studio 4.5. This protocol proceeds by clustering collected ML compounds into 20 clusters of maximum dissimilarity between cluster centres calculated based on following properties: logP, molecular weight, number of hydrogen-bond donors (HBDs), number of hydrogen-bond acceptors (HBAs), number of rotatable bonds, number of atoms, number of rings, number of aromatic rings, number of fragments, molecular polar surface, then *ca.* 30% of each cluster was moved to the testing set. This resulted in a training list that included 492 compounds (≈70% of the refined collected list) out of which 37% are actives, 50% are moderates and 13% are inactives, and a testing set that included 207 compounds (≈30%) out of which 35.7% are actives, 35.3% are moderates and 29% are inactives. ML training and testing sets are deposited in ESI Tables SM10 and SM11,† respectively, in simplified molecular-input line-entry system (SMILES) format.^18,19,22–31,33–36^

The ML sets (training and testing) were fitted against structure-based and ligand-base pharmacophores (34 from SB modelling, 27 from supervised LB modelling and 24 from unsupervised LB modelling) using the “Best Fit” option and CAESAR conformation–generation algorithm implemented in Discovery Studio (version 4.5). The fit values were calculated by ESI eqn (4) (in Section SM7†) and were used as descriptors in ML. Additionally, 134 physicochemical descriptors were also calculated for training and testing compounds, including numerous topological and fingerprint descriptors using “Calculate Molecular Properties” protocol implemented in Discovery Studio (version 4.5). Moreover, numerous (7947) engineered descriptors were also added by calculating the squares, cubic powers, square roots and cubic roots of different descriptors (physicochemical and pharmacophoric) as well as values resulting from the combinatorial multiplications of fit values of any two pharmacophores against modelled compounds. Overall, ML modelling included 8033 descriptors. The ML workflow is sketched in Fig. 3.

#### 2.5.2. Building machine learning models

Pharmacophore models generated by different methods: whether SB, supervised LB, or unsupervised LB, were allowed to compete within the context of genetic algorithm (GA) tournaments. In each contest, GA coupled with one machine learner, either Random Forests,^77,78^ or eXtreme Gradient Boosting (XGB),^79–82^ was used to select optimal combination of pharmacophore(s) and other physicochemical descriptors collectively capable of achieving best accuracies (See “ML Model Evaluation” below) in classifying the training and testing compounds into actives, moderates and inactives, according to each machine learning approach. Cohen's kappa values were also calculated as additional assessment of resulting GA-ML models (See “ML Model Evaluation” below).

##### 2.5.2.1 Genetic function algorithm-based ML modelling (GA-ML)

A gene-based encoding system is implemented herein, whereby the presence or absence of a certain descriptor(s) in a suggested model is encoded by chromosome format. That is, each potential ML model is represented as vector (chromosome) composed of string of bins (genes), whereby each bin (gene) represents a particular independent variable (descriptor), such that if a particular bin is filled with “0” then the corresponding descriptor is absent from the corresponding model under evaluation, while if the bin is filled with “1” then the corresponding descriptor is present in the model. Each chromosome (ML model) is associated with a fitness value that reflects how good it is compared to other solutions. High-ranking chromosomes are allowed to “mate”, *i.e.*, to exchange some of their genes, and their “offspring” are evaluated *via* their fitness values. Two important GA control parameters need to be configured prior to modelling, namely, the population of initial random chromosomes and the maximum number of generations to exit from a basic cycle and to complete the algorithm.^83^ In the current project, these were configured as follows: population size = 500 and a maximum number of generations = 10 000. We implemented GA node within KNIME Analytics Platform (Version 4.3.2).

##### 2.5.2.2 Random forest (RF)

RF is a multipurpose ML strategy for classification.^77,78^ RF is based on the ensemble of decision trees (DTs). Each tree predicts a classification independently and “votes” for the related class. Most of the votes decide the overall RF predictions.^84^ GA selected descriptors were used to build corresponding RF models. The fitness criterion for the associated GA models was set to the accuracy of the out-of-bag internal validation, which measures prediction error of RF models utilizing subsampling with replacement to create training and testing samples for the model.^115,116^ The RF learner node within KNIME Analytics Platform (Version 4.3.2) was implemented with the following parameters: Tree options: no minimum node size or maximum tree depth were specified, Split method: GINI, Number of trees = 100.

##### 2.5.2.3 Extreme gradient boosting (XGB)

Extreme Gradient Boosting (XGBoost, or XGB) relies on the ensemble of weak DT-type models to create boosted, DT-type models. This system includes a novel tree learning algorithm, a theoretically justified weighted quantile sketch procedure with parallel and distributed computing.^79–82^ GA selected descriptors were used to build corresponding XGB models. The fitness criterion for the associated GA models was set to the accuracy of the leave-20%-out internal crossvalidation. The XGB Learner node within KNIME Analytics Platform (Version 4.3.2) was implemented with the following parameters: tree booster was implemented with depth wise grow policy, boosting rounds = 100, eta = 0.3, gamma = 0, maximum depth = 6, minimum child weight = 1, maximum delta step = 0, subsampling rate = 1, column sampling rate by tree = 1, column sampling rate by level = 1, lambda = 1, alpha = 1, sketch epsilon = 0.03, scaled positive weight = 1. Maximum number of bins = 256.

#### 2.5.3. ML model evaluation

Optimal ML models were evaluated by calculating their accuracy and Cohen's kappa values.^85,86^ Additionally, Shapley values were calculated to determine the extent by which each GA-selected descriptor is contributing to the final ML model (whether it is RF or XGB).^87^

##### 2.5.3.1 Accuracy

The accuracies of optimal ML models against the training and testing sets were calculated based on the following equation.^85,86^

Where *N* is the number of all compounds in the testing database, TP and TN are the numbers of truly identified “actives” and “inactives”, respectively. Accuracy evaluation against the training set involves removing one (*i.e.*, leave-one-out) or 20% (*i.e.*, leave-20%-out or 5-fold cross-validation) of the data points (*i.e.*, compounds), then building the particular ML model from the remaining data. The model is then used for classifying the removed compounds. The process is repeated until all training data points are removed from the training list and predicted at least once. Accuracy is calculated based on comparing classification results with actual bioactivity classes. Evaluation against the testing set involves calculating the accuracy of the particular ML model by comparing its classification results with the actual bioactivity classes of the testing.^88–92^

##### 2.5.3.2 Cohen's kappa

The resulting ML models were also validated by Cohen's Kappa depending on the following equation.^93,94^
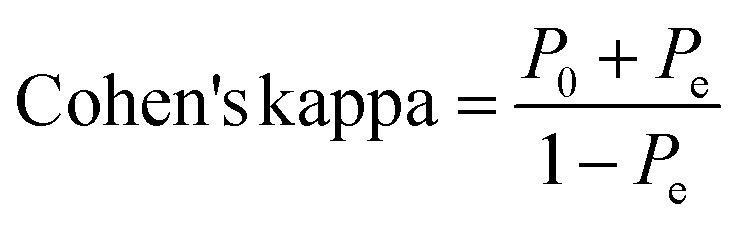
where *P*_0_ is the relative observed agreement among raters (*i.e.*, accuracy), and *P*_e_ is the hypothetical probability of chance agreement. This done by using the observed data to calculate the probabilities of each observer randomly seeing each category. If the raters are in complete agreement, then kappa = 1. If there is no agreement among the raters other than what would be expected by chance (as given by *P*_e_), kappa = 0. Negative Cohen's kappa value implies the agreement is worse than random. Other values are as follows: 0.01–0.20 are considered as none to slight, 0.21–0.40 as fair, 0.41–0.60 as moderate, while 0.61–0.80 as substantial agreement.^93,94^

##### 2.5.3.3 Shapley values (SHAP)

The Shapley value of a particular feature for a certain observation (compound) and prediction indicates how much the feature has contributed to the deviation of the prediction from the base prediction (*i.e.*, the mean prediction over the full sampling data).^87,95^ The SHAP node was implemented within KNIME Analytics Platform (Version 4.3.2) using K-means to summarize the data to 100 prototypes to start the SHAP node loop. The following parameter was used in SHAP loop: explanation set size (the maximum number of samples SHAP is allowed to use for its estimations) = 100.

ML selected pharmacophores were carefully validated as described earlier^39,86,96,97^ (*e.g.*, by ROC analysis). Detailed description of the validation is in ESI Section SM11.†

### 2.6. *In Silico* screening for new LSD-1 inhibitors

Pharmacophore hypotheses that emerged in optimal ML models were employed as 3D search queries to screen the NCI list of compounds (includes 268 667 compounds). Screening was performed employing the “Best Flexible Database Search” protocol implemented within Discovery Studio (version 4.5). To predict the activity class of captured hits, each hit was fitted against pharmacophores within the particular optimal ML model using “best fit” option implemented in Discovery Studio and as calculated by ESI eqn (45) (Section SM4†). Other ML selected descriptors were also calculated for hit molecules. Eventually, fit values and other descriptors were substituted in optimal ML models to predict LSD-1 activity classes of hits. Hits predicted to be within the “Active” category or as combination of “Active” and “Moderate” (76 compounds) were acquired for subsequent *in vitro* testing.

### 2.7. *In vitro* experimental studies

#### 2.7.1. Cell culture

The anticancer effects of captured hits were evaluated against SH-SY5Y (Neuroblastoma, CRL-2266), Panc-1 (pancreatic carcinoma, CRL-1469) and U-87 MG (glioblastoma, HTB-14) cells. Details about growth conditions, cell harvesting and counting are available in ESI Sections SM12–SM14.† The cancer killing profiles of tested compounds were evaluated colorimetrically using the Cell Titer Non-Radioactive Cell Proliferation Assay Kit® (Promega, USA) based on the reduction of a yellow tetrazole, 3-(4,5-Dimethylthiazol-2-yl)-2, 5-diphenyl tetrazolium bromide (MTT), to a purple formazan, a process that takes place in the mitochondria of viable cells (details are in ESI Sections SM12–SM14†).^98,99^

As SH-SY5Y cells highly express ATP binding cassette subfamily B member 1 (ABCB1, MDR1, normalized expression, NX: 36.8%),^100^ cell resistance were reversed using verapamil (10 μM), which was added with each hit, and incubated with cells for 72 hours for subsequent MTT viability assay.^101,102^ Finally, MTT cell proliferation assay results were analysed using GraphPad Prism 8.0 (GraphPad Software, Inc). The inhibitory IC_50_ values (concentration at which 50% of tested cells are viable) were calculated from the logarithmic trend lines of the corresponding dose–cytotoxicity graphs. r^2^ and hill slope were also obtained. Measurements were repeated in triplicates.

#### 2.7.2. *In vitro* LSD1 enzymatic activity

The demethylase activity of LSD-1 was quantitatively detected *in vitro* using the fluorometric LSD-1 Assay Kit from Cayman Chemical-USA (Cat# 700120) according to the manufacturer's instructions. Briefly, the assay is based on a multistep enzymatic reaction in which LSD-1 first produces H_2_O_2_ during the demethylation of lysine 4 on a peptide corresponding to the first 21 amino acids of the N-terminal tail of histone H3. In the presence of horseradish peroxidase (HRP), H_2_O_2_ reacts with ADHP (10-acetyl-3,7-dihydroxyphenoxazine) to produce the highly fluorescent compound resorufin. Resorufin fluorescence was analysed with an excitation wavelength 530–540 nm and an emission wavelength of 585–595 nm. Prominent hits were evaluated against LSD-1 enzyme at different concentrations ranging from 10 μM to 0.01 μM, along with tranylcypromine as assay positive control. The procedure proceeds through the following steps: (i) prepare 100% initial activity wells by adding 120 μl of assay buffer, 20 μl of LSD1, 20 μl of HRP, 10 μl of ADHP, and 10 μl of DMSO to three wells. (ii) Prepare of background wells by adding 140 μl of assay buffer, 20 μl of LSD-1, 20 μl of HRP, 10 μl of ADHP, and 10 μl of DMSO to three wells. (iii) Prepare inhibitor wells by adding 120 μl of assay buffer, 20 μl of LSD-1, 20 μl of HRP, 10 μl of ADHP, and 10 μl of tested hit to three wells. (iv) Initiate the reactions by adding 20 μl of substrate peptide to all the wells being used, except the background wells. (v) Cover the plate with the plate cover and incubate for 30 minutes at 37 °C. (vi) Remove the plate cover and read the plate using an excitation wavelength of 530–540 nm and an emission wavelength of 585–595 nm. (vii) Determine the average fluorescence of each sample. (viii) Subtract the fluorescence of the background wells from the fluorescence of the 100% initial activity and the inhibitor wells. (ix) Determine the percent inhibition for each sample. To do this, subtract each inhibitor sample value from the 100% initial activity sample value. Divide the result by the 100% initial activity value and then multiply by 100 to give the percent inhibition. 
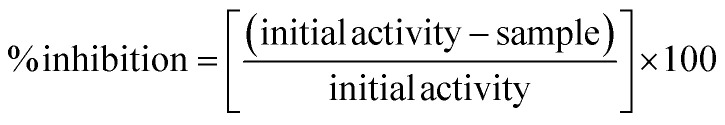
 Finally, plot the percent inhibition as a function of the inhibitor concentration to determine the IC_50_ value (concentration at which there was 50% inhibition).

## 3. Results and discussion

### 3.1. LSD-1 pharmacophore model generation

#### 3.1.1. Structure-based models

In this approach, ligand/LSD-1 crystallographic and virtual (docking-generated) complexes were used to extract pharmacophore models that were subsequently allowed to compete within GA-ML settings.

##### 3.1.1.1 Crystallographic complex-based pharmacophore modelling

Nine LSD-1 crystal structures (5 L3E, 5LBQ, 5LGN, 5LHG, 5LGT, 5LGU, 5LHH, 5LHI and 5YJB) retrieved from the Protein Data Bank (PDB) (https://www.rcsb.org/pdb) and used to extract pharmacophore models using the “Receptor-Ligand Pharmacophore Generation” protocol implemented in Discovery Studio (version 4.5). The resulting pharmacophores (27 models) were mapped against bioassay-consistent inhibitors 1–198 (ESI Table SM1†), sixteen pharmacophores failed to map more than 5 compounds from the list and six of the models had less than four diverse features. These models were discarded, leaving five pharmacophores (shown in ESI Fig. SM2†) for subsequent ML.

##### 3.1.1.2 Docking-based pharmacophore modelling

The available co-crystallized LSD-1 ligands are of limited chemical diversity. Furthermore, crystallographic complexes usually show some redundant ligand–protein interactions (as they do not account for variations in bioactivity across bioactive ligands). Additionally, given that hydrogen atoms cannot be seen by X-ray, it is challenging to predict the ionization state of complexed ligands.^103–107^ These issues prompted us to complement pharmacophore models extracted from crystallographic structures with pharmacophores extracted from virtual complexes generated by docking studies. It is reasonable to assume that high-ranking docking solutions, provided by several docking-scoring algorithms, for a set of diverse potent ligands should include realistic binding modes that resemble actual binding pharmacophore(s). ML can then be used as competition arena to select optimal pharmacophore models capable of explaining bioactivity variation within a modelled list of training compounds. This approach should yield realistic pharmacophore model(s) that might be overlooked in crystallographic complexes.^40,108^

Nine diverse ligands (199, 206, 208, 224, 230, 248, 256, 270 and 276, see ESI Table SM1†) were selected as representatives of the known potent population of LSD-1 inhibitors. Subsequently, they were docked into the binding pocket of LSD-1 in their ionized and unionized states (to compensate for the fact that ligand ionization state is hard to predict within protein binding site).^74,109^ Three docking engines and 7 scoring functions were implemented in the docking study to diversify the docking solutions of each ligand. This was done to enhance the probability of ML converging on some realistic binding pharmacophore(s) that can best classify LSD-1 ligands based on bioactivity. The process yielded 336 virtual complexes ([7 ionizable compounds × 2 ionization states × 3 docking engines × 7 scoring functions] + [2 non-ionizable compounds × 3 docking engines × 7 scoring functions] = 336). Subsequent use of the “Receptor-Ligand Pharmacophore generation” protocol of Discovery Studio on the resulting virtual complexes yielded 902 pharmacophores. However, these were reduced to 29 representative pharmacophores by discarding 289 pharmacophores that fail to map more than 5 compounds or with less than 4 diverse features, and subsequent clustering of the remaining pharmacophores to allow only diverse models to proceed to ML. The later step was done because similar pharmacophores cause co-linearity-related noise-to-signal issues and ML errors.^110,111^

#### 3.1.2. Ligand-based pharmacophores

##### 3.1.2.1 Supervised pharmacophore modelling

The pharmacophoric space of LSD-1 inhibitors was extensively explored using supervised ligand-based pharmacophore modelling.^63,73,75,112^ For this purpose, 8 training subsets were carefully selected from collected LSD-1 inhibitors bioassayed by similar procedures (1–198, ESI Table SM1†). Each training subset was selected in such a way to conform with certain envisaged binding mode. Subsequently, the training subsets were used to build 407 pharmacophore models through 64 bioactivity-supervised pharmacophore generation automatic runs within Discovery Studio. However, these were reduced to 27 unique binding hypotheses by discarding pharmacophores that fail to map more than 5 compounds or exhibit less than 4 diverse features, and by subsequent clustering/selecting best cluster representatives to minimize collinearity among pharmacophore descriptor and improved noise-to-signal ratio in subsequent ML.^110,111^

##### 3.1.2.2 Unsupervised pharmacophore modelling

To account for binding pharmacophores that could emerge from LSD-1 inhibitors bioassayed by discrete bioassay methods, we opted to explore the pharmacophoric space of such inhibitors using unsupervised pharmacophore modelling. This was performed employing the “Common Feature Pharmacophore Generation” protocol within Discovery Studio.^59,108^ For this purpose, 16 diverse training subsets were carefully selected from collected LSD-1 inhibitors regardless of their bioassay-procedure (compound 1–281, ESI Table SM1†). Members of each training subset were selected in such a way to conform to certain envisaged binding mode. Subsequently, the training subsets were used to build 882 pharmacophore models through 192 automatic runs. However, these were reduced to 24 by discarding pharmacophores that fail to map more than 5 compounds and those of less than 4 diverse features, and subsequent clustering to minimize collinearity and improved noise-to-signal ratio in subsequent ML.^110,111^

### 3.2. ML-guided selection of LSD-1 pharmacophores

The fit values of 85 pharmacophores generated by different modelling techniques (SB modelling (34 pharmacophores), supervised LB (27 pharmacophores) and unsupervised LB (24 pharmacophores)) against modelled compounds (using eqn (4), see ESI Section SM7†) were pooled together with 134 additional physicochemical descriptors calculated for the modelled compounds. The collected pharmacophores were allowed to interact by calculating the combinatorial multiplications of any two pharmacophores (*i.e.*, their fit values against modelled compounds), their squares, cubic powers, square roots and cubic roots. This yielded 7813 additional descriptors. Accordingly, the overall number of descriptors enrolled as independent variables in ML were 8032, while the bioactivity class (*i.e.*, active, moderate or inactive) was enlisted as dependent response variable.

The sheer number of available explanatory features (*i.e.*, descriptors) means it is necessary to couple ML with GA to single out critical descriptors that control the bioactivity class within training and testing compounds. Needless to say, ML classifiers fail solely to infer useful information about descriptor(s) of greatest control over response (*i.e.*, bioactivity class).^113^ Accordingly, 8032 features were allowed to compete within the context of GA tournaments using Cohen's kappa of the resulting models as GA fitness criteria.^83^ The reason for including Cohen's kappa as success criteria to judge the resulting models is because the training set is imbalanced^114^*i.e.*, the training list included 37% actives, 50% moderates and 13% inactives. In contrast, the testing set is better balanced with 35.7% actives, 35.3% moderates and 29% inactives.

Seven ML were coupled with GA and evaluated, namely, Support Vector Machine (SVM), Probabilistic Neural Network (PNN), Naïve Bayes (NB), logistic regression, RF, XGB and kNN. However, only two, namely XGB and RF yielded statistically significant classification results.

**Fig. 4:**
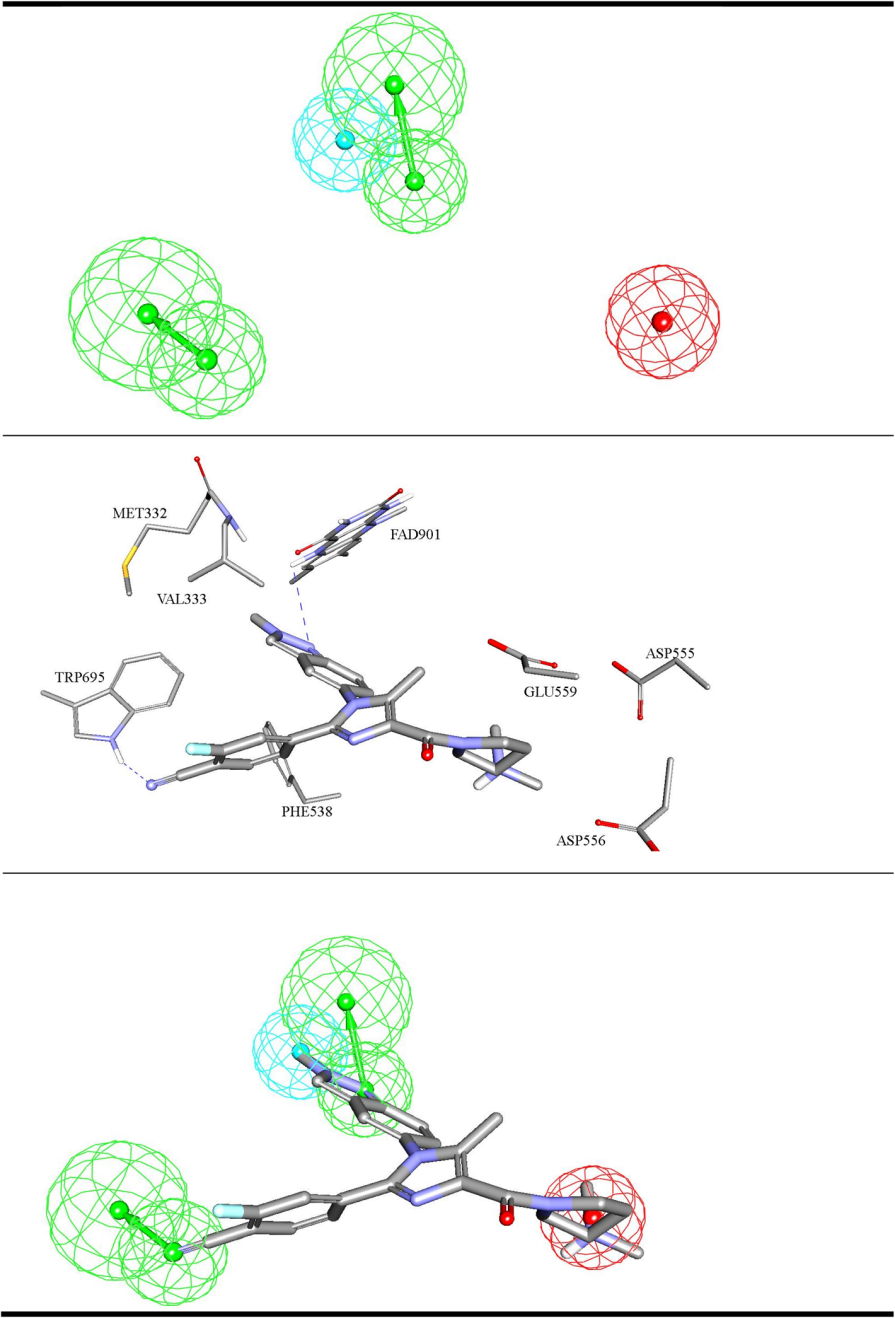
ML-selected structure–based pharmacophore model: Hypo-SB1 (details are as in Table 1). The middle image shows the corresponding virtual docking complexes of 206 (Table SM1†) docked into LSD-1 crystal structure (PDB: 5LHI) (details in Table SM3 under ESI†) from which corresponding pharmacophore was extracted as well as the pharmacophore models fitted against respective docked ligands. PosIon are shown as red spheres, Hbic features as light blue spheres, HBA as green vectored spheres. Hydrogen bonding interactions are shown as blue dashed lines.

**Fig. 5:**
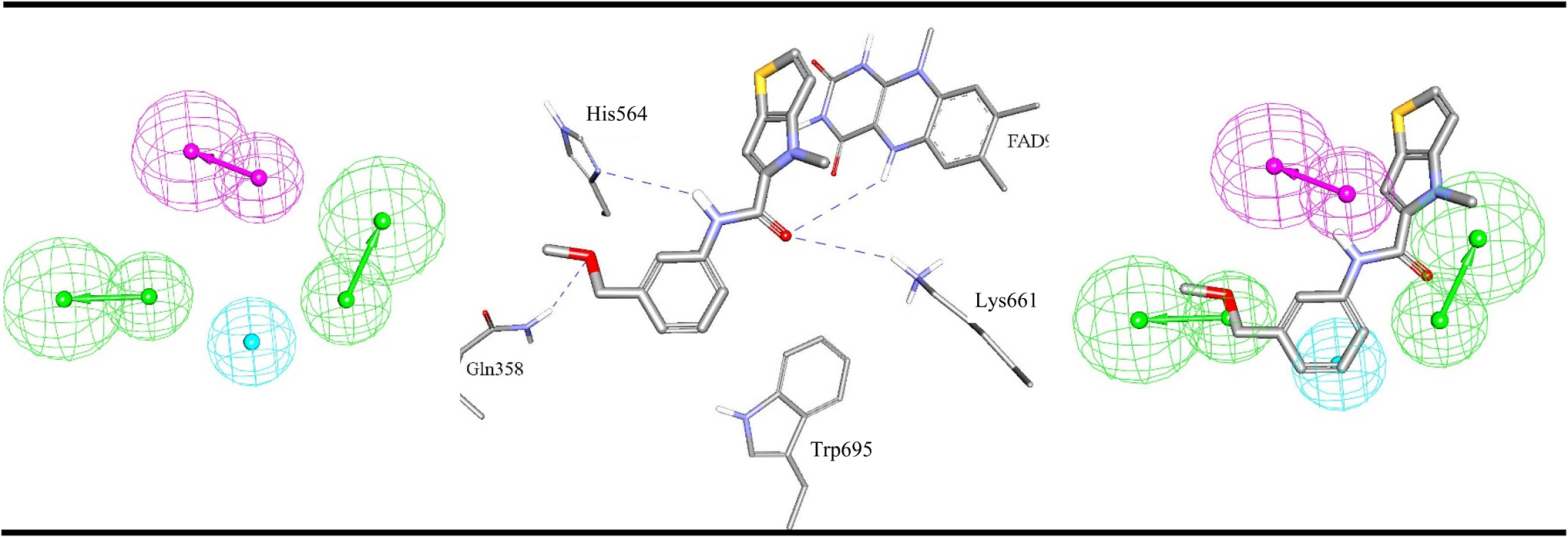
ML-selected ligand–based pharmacophore models generated by supervised modelling (HYPOGEN), Hypo-LB5 (details are as in Table 1). The middle image shows crystallographic complex (PDB codes: 5LGN) showing binding interactions analogous to corresponding pharmacophoric features. Images to the right show the same crystallographic ligands fitted within the corresponding pharmacophore model. Hbic feature is shown as light blue spheres, HBD as purple vectored spheres, HBA as green vectored spheres. Hydrogen bonding interactions are shown as blue dashed lines.

**Fig. 6:**
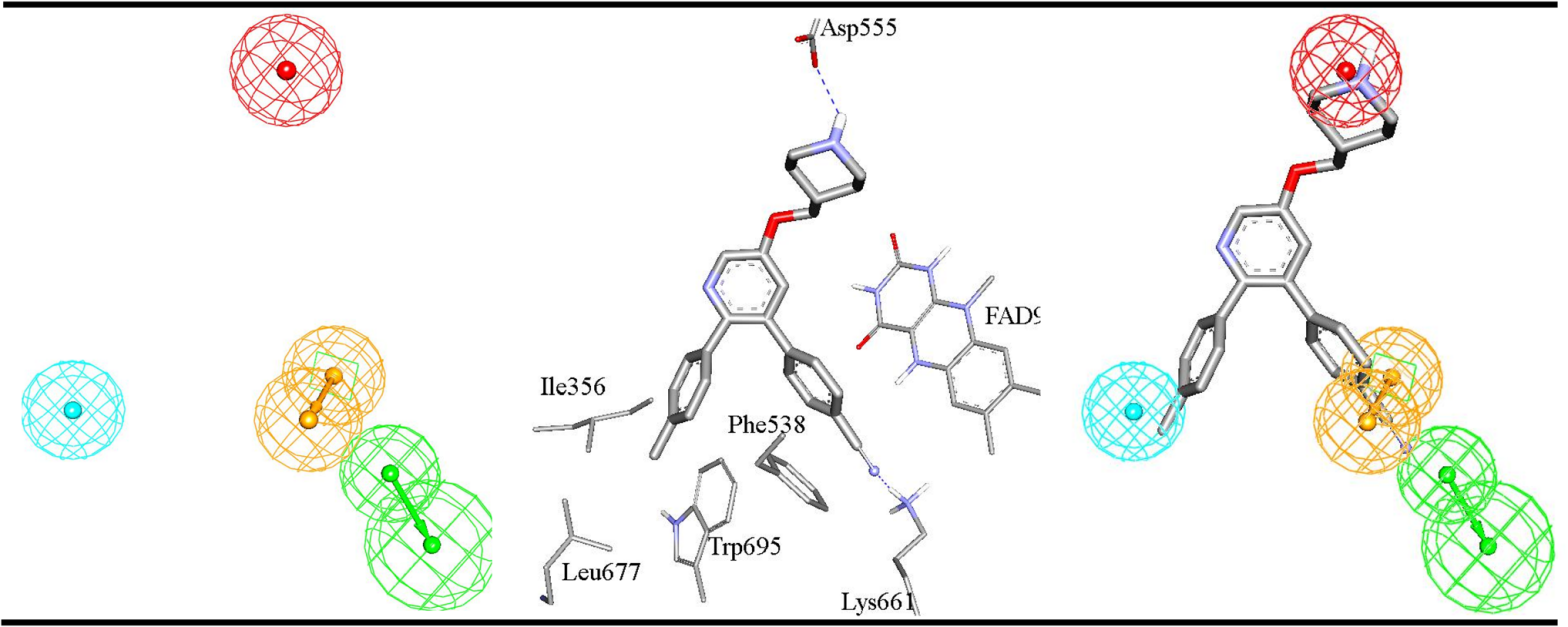
ML-selected ligand–based pharmacophore models generated by unsupervised modelling, Hypo-LB6 (details are as in Table 1). The middle image shows crystallographic complex (PDB code: 5YJB) showing binding interactions analogous to corresponding pharmacophoric features. Image to the right show the same crystallographic ligands fitted within the corresponding pharmacophore model. PosIon are shown as red spheres, Hbic features as light blue spheres, RingArom as brown vectored spheres, HBA as green vectored spheres. Hydrogen bonding interactions are shown as blue dashed lines.

Table 1 shows the selected descriptors and statistical results of XGB and RF classifiers. The XGB model was validated by external testing as well as internal cross validation through leave-one-out, leave-20%-out. However, the RF model was validated by internal out-of-bag cross-validation (measures prediction error of RF models utilizing subsampling with replacement to create training and testing subsamples from the training set^77,78^) and external testing set.^115,116^ The selected pharmacophores were further validated by receiver operating characteristic (ROC) curve analysis shown in Table 2. Fig. 4–6 show ML-selected pharmacophores, while Table 3 shows the *X*, *Y*, *Z* coordinates of the pharmacophore features of each pharmacophore model.

**Table 1:** Accuracy and Cohen's kappa statistics of GA-ML models

ML method^a^	Selected model descriptors^b^^,^^c^	Out-of-bag		Leave-20%-out^f^		Leave-one-out^f^		Testing set^g^	
		Accuracy	Cohen's kappa	Accuracy	Cohen's kappa	Accuracy	Cohen's kappa	Accuracy	Cohen's kappa
XGB	Hypo-SB1, NPlusO_Count, kappa_2, (ES_Sum_tN)^3^, (Num_AromaticBonds)^0.3^	NA^d^	NA^d^	0.83	0.71	070	0.55	0.72	0.57
RF	Hypo-SB1, Hypo-LB5, Hypo-LB6, ES_Count_ssCH_2_, Num_AromaticBonds, JX	0.81	0.69	ND^e^	ND^e^	ND^e^	ND^e^	0.63	0.43

a XGB: eXtreme gradient boosting, RF: random forests.

b Hypo-SB1 (Fig. 4) corresponds to the 8th pharmacophore model (serial number) extracted from the docked pose of ionized 206 (Table SM1) within LSD-1 (PDB code: 5LHI) as generated by LibDock and PMF04 scoring function. Hypo-LB5 (Fig. 5): the 2nd pharmacophore generated from training subset F (Table SM4, under ESI) using settings of run 2 in Table SM5 (under ESI). Hupo-LB6 (Fig. 6): the 4th pharmacophore generated from training subset S (Table SM7, under ESI) using settings of run 1 in (Table SM8, under ESI).

c NPlusO_Count: is the total number of oxygen and nitrogen atoms in the molecule, kappa_2: is the kappa shape index that correlated with compound structure elongation, ES_Sum_tN: electrotopological state number of terminal nitrogen atom, Num_AromaticBonds and, ES_Count_ssCH_2_: electrotopological state counts for sp_2_ hybridized carbon atoms, JX: one of Balaban indices take account of the covalent radii (JX) of the atoms of the molecule.^72^

d Not applicable.

e Not determined.

f Training list include 492 compounds.

g Testing includes 207 compounds.

**Table 2:** Receiver operating characteristic curve information of pharmacophores selected by GA-ML classification models

Pharmacophores^a^	ROC-AUC^b^ (%)	ACC^c^ (%)	SEN^d^ (%)	SPC^e^ (%)
Hypo-SB1	71	59	45	97
Hypo-LB5	65	67	72	55
Hypo-LB6	71	59	42	100

a pharmacophore models are shown in (Fig. 4–6) and their *X*, *Y*, *Z* coordinates are shown in Table 3.

b Area under the receiver operating characteristic curve.

c Accuracy.

d Sensitivity.

e Specificity.

**Table 3:** ML-selected pharmacophores: their binding features, 3D-coordinates and tolerances

Model	Definition	Chemical features														
Hypo-SB1			HBA			HBA					RingArom			PosIon		
	Tolerances		1.60	2.20		1.60			2.20		1.60		2.20	1.60		
	Coordinate	*X*	9.63	10.73		6.67			8.70		7.45		6.68	1.15		
		*Y*	−50.07	−52.29		−56.23			−58.13		−55.38		−54.30	−51.03		
		*Z*	−42.60	−44.28		−37.57			−36.46		−37.46		−40.15	−33.30		
Hypo-LB5			HBA				HBA				HBD					Hbic
	Weights		1.76				1.76				1.76					1.76
	Tolerances		1.60		2.20		1.60		2.20		1.60		2.20			1.60
	Coordinate	*X*	1.74		2.79		−0.63		1.54		4.29		5.94			−1.00
		*Y*	1.64		3.77		−2.11		−3.44		−1.97		−1.63			0.06
		*Z*	−4.62		−6.48		0.99		2.62		−0.81		−3.31			−2.06
Hypo-LB6			HBA					RingArom				Hbic			PosIon	
	Weights		1.00					1.00				1.00			1.00	
	Tolerances		1.70			2.30		1.70		1.70		1.70			1.70	
	Coordinate	*X*	1.33			2.30		4.57		4.56		6.78			−7.33	
		*Y*	3.83			5.30		−1.15		−1.16		−2.56			0.58	
		*Z*	6.60			9.08		0.003		3.00		0.04			−3.60	

SHAP values within the context of cheminformatics machine learning enable the identification and prioritization of features that determine compound activity prediction regardless to the ML model.^87^ The SHAP value of a feature for a certain compound indicates how much the feature has contributed to the deviation of the prediction from the base prediction of that compound (*i.e.*, the mean prediction).

**Fig. 7:**
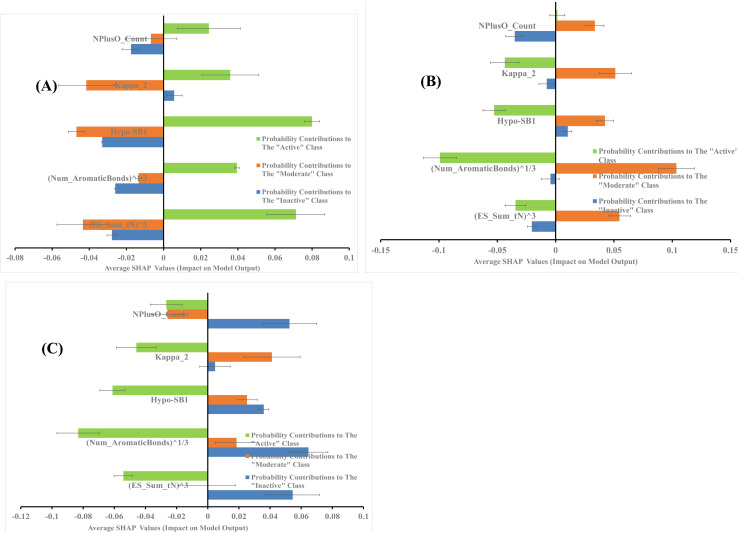
SHAP values probability contributions of descriptors emerging in optimal GA-XGB model. (A) Within the “Active” compounds of the testing set (74 molecules), (B) within the “Moderate” compounds of the testing (73 molecules) (C) within “Inactive” compounds of the testing set (60 molecules). Each SHAP histogram represents the average deviations produced by the corresponding descriptor. Error bars represent the standard error of the average.

**Fig. 8:**
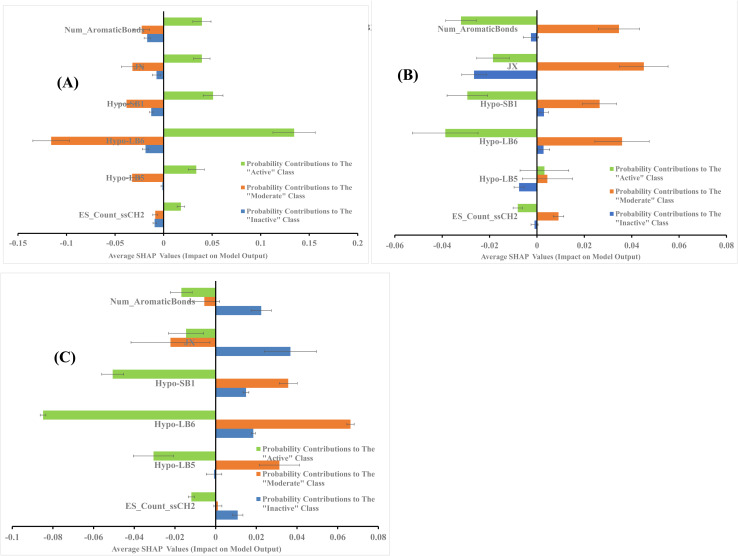
SHAP values probability contributions of descriptors emerging in optimal GA-RF model. (A) Within the “Active” compounds of the testing set (74 molecules), (B) within the “Moderate” compounds of the testing (73 molecules) (C) within “Inactive” compounds of the testing set (60 molecules). Each SHAP histogram represents the average deviations produced by the corresponding descriptor. Error bars represent the standard error of the average.

The average SHAP values for GA-selected descriptors in the best GA-XGB and GA-RF models are shown in Fig. 7 and 8. Each value was calculated as the mean of SHAP contributions of the particular descriptor across active, moderate or inactive testing compounds, respectively (the same list used for validating ML models and for ROC analysis of pharmacophores). Clearly from the figures, the selected ML methods (*i.e.*, XGB and RF) are orthogonal as can be seen from their different features and corresponding contributions. Additionally, most GA-selected descriptors exhibited SHAP probability contributions consistent with the bioactivity categories of testing compounds, *i.e.*, they yielded positive probabilities towards the “Active” classification within active testing compounds, while in the same descriptors penalized the probabilities of having moderate or inactive compounds in the same class. Similarly, they showed positive probability contributions towards “Moderate” bioactivity within the moderate testing category, while they penalized the probabilities of having “Active” or “Inactive” compounds within the same category (moderate). A similar trend is seen in “Inactive” category albeit with significant noise resulting from promoting the probabilities of “Moderate” bioactivities within the inactive category. It can be concluded from Fig. 7 and 8 that the most significant and consistent contributors to probability predictions are produced by Hypo-SB1 in both GA-XGB and GA-RF models, while Hypo-LB5 and Hypo-LB6 in GA-RF model.

Fig. 4–6 show how different ML-selected pharmacophores correlate with binding interactions anchoring potent inhibitors within the binding site of LSD-1. Generally, pharmacophores converge on electrostatic attractive interactions linking cationic centers in the ligands with the carboxylates of Asp555, Asp556 or Glu559. Nevertheless, some potent ligands seem to lack such cationic centers. These are successfully represented by Hypo-LB5 that lack this feature (Fig. 5). Additionally, ML-selected pharmacophores seem to encode for hydrogen bonding (HBA or HBD) connecting the ligands with the aromatic system of FAD cofactor, as can be clearly seen in Hypo-SB1 and Hypo-LB5 (Fig. 4 and 5). Still, this interaction is replaced by hydrogen bonding with Lys661 in Hypo-LB6 (Fig. 6). Less significant hydrogen bonding interactions are highlighted by individual pharmacophores including SB pharmacophore Hypo-SB1 which highlight hydrogen bonding with Trp695 as in Fig. 4. Similarly, LB-pharmacophore Hypo-LB5 highlight the interaction with Gln358 and His564 as in Fig. 5.

Moreover, ML-selected pharmacophores appear to converge on hydrophobic/Van der Waals' interactions that anchor ligands into a hydrophobic pouch composed of the side chains of Met332, Val333, Ile356, Phe538, Leu677 and Trp695 (Fig. 4–6).

### 3.3. *In Silico* screening for new LSD-1 inhibitors followed by *in vitro* validation

**Fig. 9:**
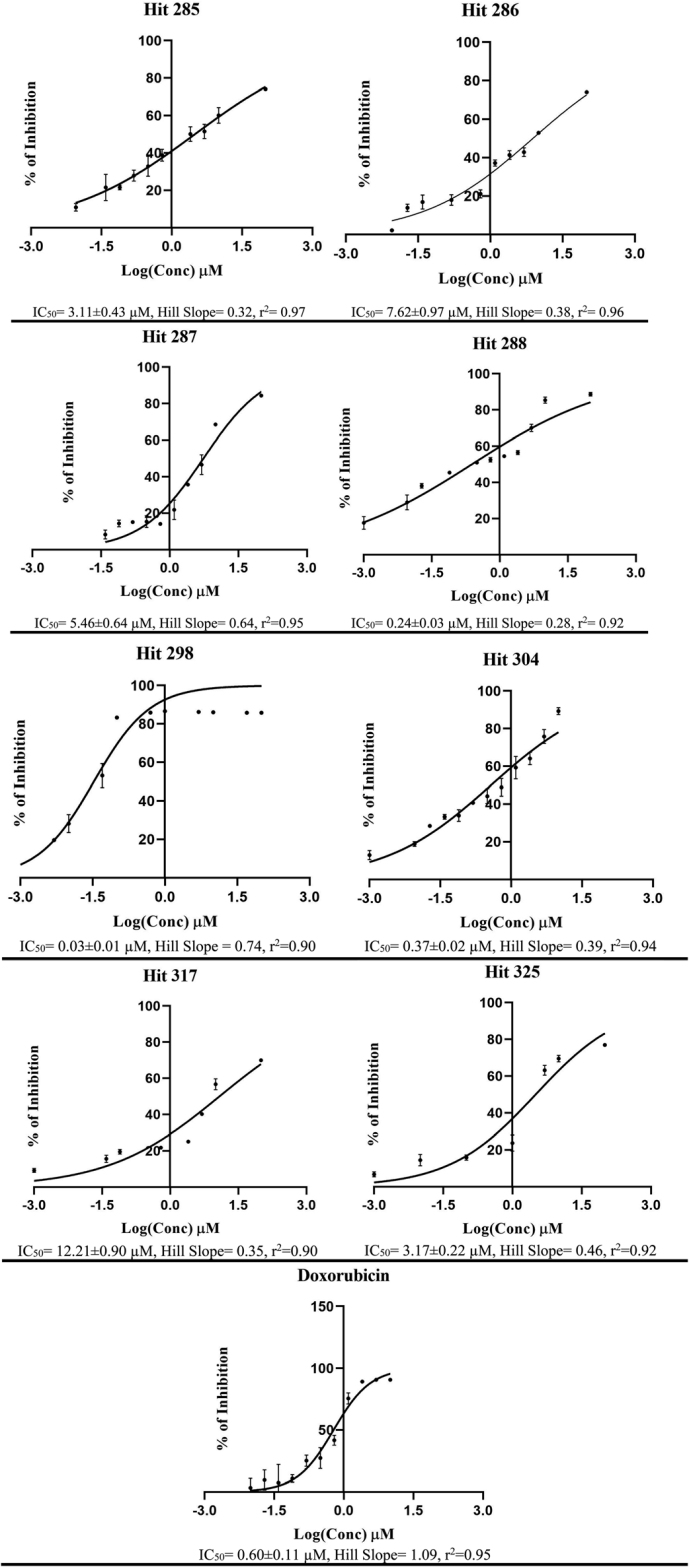
Dose–response curves for most active hits 285, 286, 287, 288, 298, 304, 325, 317 and doxorubicin against neuroblastoma SH-SY5Y cells, corresponding chemical structures are shown in ESI Fig. SM3.† Each point represents triplicate measurements. Compounds 285, 286, 287, 288, 304 and 317 were evaluated in presence of verapamil (10 μM).

The fundamental use of pharmacophores and associated ML models is for the discovery of new chemical scaffolds of similar biological profiles (*i.e.*, scaffold hopping). Therefore, it was decided to use Hypo-SB1, Hypo-LB5 and Hypo-LB6 as 3D search queries to screen the NCI list (265 667 compounds) for new LSD-1 inhibitors. Hypo-SB1 captured 5660 hits, Hypo-LB5 captured 91 885 hits while Hypo-LB6 captured 2593 hits. Captured hits were fitted against the three pharmacophores and their fit values together with other relevant calculated descriptors were substituted in ML models in Table 1 to predict their anti-LSD-1 bioactivity classifications. Top ranking hits (76 compounds), *i.e.*, predicted by ML models to be active or moderate, were requested from the NCI and tested *in vitro*. ESI Fig. SM3† shows the chemical structures of tested hits, while Table 4 shows their NCI codes, predicted and experimental bioactivities against neuroblastoma SH-SY5Y cells (which we selected because they overexpress LSD-1).^117,118^ From Table 4, it is evident that hits 285, 286, 287, 288, 298, 304, 317 and 325 illustrate significant cytotoxic effects against SH-SY5Y cancer cells. Therefore, their bioactivities were further evaluated at different concentrations. Fig. 9 shows their corresponding dose–response curves. Clearly, they are of reasonable steepness and excellent correlation coefficients. Collectively, these qualities together with the fact that SH-SY5Y cells over-expressed LSD-1 (of normalized expression 42.5%)^100^ suggest these compounds are potent inhibitors of LSD-1. Some hits required the addition of the P-glycoprotein efflux pump inhibitor, verapamil (10 μM), to reveal their inhibitory profiles against SH-SY5Y cells (their IC_50_ values marked with asterisks in Table 4).

**Table 4:** High-ranking hits, capturing pharmacophores, prediction of bioactivity class by ML models

Hits^a^	NCI code	Captured by^b^	ML class prediction		Experimental % inhibition^c^			*In vitro* IC_50_ μM^c^
					Without verapamil	With verapamil		
			XGB	RF	At 50 μM	At 10 μM		
282	26 158	1, 2 and 3	Active	Active	6.16	0.06	ND^d^	ND
283	58 439	1 and 2	Active	Active	51.22	34.30	ND	ND
284	76 533	1, 2 and 3	Active	Active	52.91	49.79	ND	ND
285	84 456	1, 2 and 3	Active	Active	72.12	8.76	60.09	3.11^f^
286	85 203	1, 2 and 3	Active	Moderate	63.64	0.06	49.96	7.62^f^
287	106 717	1, 2 and 3	Active	Active	87.63	47.58	60.50	5.46^f^
288	115 848	1 and 2	Active	Active	88.50	9.46	85.41	0.24^f^
289	120 919	1 and 2	Active	Active	12.67	6.79	ND	ND
290	123 463	2	Active	Active	46.79	23.46	ND	ND
291	123 469	1 and 2	Active	Moderate	49.67	46.54	ND	ND
292	125 845	1 and 2	Active	Active	27.42	41.38	ND	ND
293	26 872	1, 2 and 3	Active	Active	26.82	0.02	ND	ND
294	127 751	1 and 2	Active	Moderate	28.17	29.46	ND	ND
295	133 667	1 and 2	Active	Active	47.38	37.92	ND	ND
296	135 759	1 and 2	Active	Active	36.04	7.68	ND	ND
297	135 764	1 and 2	Active	Moderate	30.01	14.05	ND	ND
298	146 496	1, 2 and 3	Active	Active	86.99	86.01	89.54	0.03
299	211 141	1, 2 and 3	Active	Active	43.36	19.02	ND	ND
300	211 284	1 and 2	Active	Moderate	86.13	14.16	21.25	ND
301	211 622	1, 2 and 3	Active	Active	37.58	13.37	ND	ND
302	211 623	1 and 2	Active	Active	24.16	22.58	ND	ND
303	211 632	1, 2 and 3	Active	Active	51.67	42.23	ND	ND
304	34 574	1 and 2	Active	Active	88.87	20.96	89.31	0.37^f^
305	211 641	1, 2 and 3	Active	Active	55.91	34.32	ND	ND
306	212 212	1 and 2	Active	Active	34.55	10.05	ND	ND
307	247 461	1, 2 and 3	Active	Active	36.37	32.73	ND	ND
308	325 622	1, 2 and 3	Active	Moderate	85.37	37.63	16.60	ND
309	333 714	1 and 2	Active	Active	45.58	28.47	ND	ND
310	54 104	2	Active	Active	77.75	42.22	11.76	ND
311	374 899	1, 2 and 3	Active	Active	34.55	25.71	ND	ND
312	375 794	1, 2 and 3	Active	Active	27.02	33.61	ND	ND
313	681 721	1, 2 and 3	Active	Active	38.99	29.69	ND	ND
314	681 731	1 and 2	Active	Moderate	81.70	45.84	32.01	ND
315	45 525	2	Active	Active	12.34	5.58	ND	ND
316	714 540	1 and 2	Active	Moderate	62.77	49.58	45.01	ND
317	35 840	1, 2 and 3	Active	Active	82.71	47.42	56.74	12.21^f^
318	51 186	1 and 2	Active	Moderate	49.47	0	ND	ND
319	55 149	1 and 2	Active	Active	25.91	0	ND	ND
320	55 230	1 and 2	Active	Active	0	0	ND	ND
321	57 155	1, 2 and 3	Active	Active	52.45	5.41	ND	ND
322	18 351	3	Active	Active	33.04	29.78	ND	ND
323	59 228	2	Active	Moderate	26.6	15.79	ND	ND^d^
324	60 850	2	Active	Active	14.29	6.08	ND	ND
325	64 983	3	Active	Active	88.81	88.85	69.57	3.17
326	71 957	2	Active	Active	54.80	17.72	ND	ND
327	81 291	2	Active	Active	44.89	4.87	ND	ND
328	95 623	1 and 2	Active	Moderate	28.62	7.40	ND	ND
329	97 860	2	Active	Active	24.41	9.53	ND	ND
330	97 902	2	Active	Active	16.52	12.96	ND	ND
331	101 100	2	Active	Active	48.65	13.25	ND	ND
332	109 609	2	Active	Active	30.13	40.68	ND	ND
333	32 097	2	Active	Active	70.34	33.95	0.08	ND
334	113 226	2	Active	Active	36.55	16.48	ND	ND
335	113 906	2	Active	Active	27.76	19.05	ND	ND
336	119 798	2	Active	Active	61.77	34.43	27.43	ND
337	121 360	2	Active	Active	23.21	27.77	ND	ND
338	130 229	2	Active	Active	36.22	13.01	ND	ND
339	133 072	2	Active	Active	13.82	12.29	ND	ND
340	157 395	2	Active	Active	35.32	17.36	ND	ND
341	168 523	2	Active	Active	87.71	38.44	36.15	ND
342	203 913	2	Active	Active	72.32	12.8	27.26	ND
343	213 775	2	Active	Moderate	32.21	16.08	ND	ND
344	32 453	2	Active	Moderate	79.40	47.35	40.59	ND
345	216 807	2	Active	Active	41.36	33.83	ND	ND
346	56 737	2	Active	Active	60.50	44.67	24.75	ND
347	270 414	2	Active	Active	13.96	0.06	ND	ND
348	370 162	2	Active	Active	22.73	19.96	ND	ND
349	54 427	2	Active	Moderate	25.69	23.30	ND	ND
350	623 088	2	Active	Moderate	30.47	9.16	ND	ND
351	631 831	2	Active	Active	29.14	15.63	ND	ND
352	632 240	2	Active	Active	17.21	6.32	ND	ND
353	635 116	2	Active	Active	59.36	45.86	ND	ND
354	638 220	2	Active	Moderate	30.33	3.24	ND	ND
355	34 702	2	Active	Active	52.60	37.02	ND	ND
356	638 222	2	Active	Moderate	25.70	11.10	ND	ND
357	39 856	2	Active	Active	7.08	0	ND	ND
Tranylcypromine^e^					27.83	0	ND	ND
Doxorubicin^e^					ND	91	ND	0.60

a Chemical structures are shown in Fig. SM3.

b 1: Hypo-SB1, 2: Hypo-LB5 and 3: Hypo-LB6.

c MTT cell viability assay on SH-SY5 cell line.

d ND: Not determined.

e Positive controls of the experiments.

f IC_50_ values marked with asterisks were determined in presence of verapamil (10 μM).

It must be mentioned that being amines and amidines, these hits are prone to produce toxic nitrosamines in the gastrointestinal tract due to nitrites and nitrates present in the diet. This reaction is optimal in the stomach at pH 3 to 4.^124^ Moreover, synthesis of such compounds is complicated by potential impurities related to nitrosamine formation.^125^

However, to exclude the possibility of targeting other receptors or enzymes, it was decided to (i) directly evaluate their inhibition of LSD-1 enzymatic activity and (ii) evaluate their cytotoxic effects against three more cell lines, namely, pancreatic carcinoma Panc-1 cells (normalized expression of LSD-1 = 29.5%),^100^ glioblastoma U-87 MG cells (normalized expression of LSD-1 = 12.3%)^100^ and normal human dermal fibroblasts (HDF). Panc-1 and U-87 MG cells were used to probe LSD-1 inhibition by candidate inhibitors.^119,120^ However, testing against HDF is aimed at evaluating the selective cytotoxicities of hits.

**Fig. 10:**
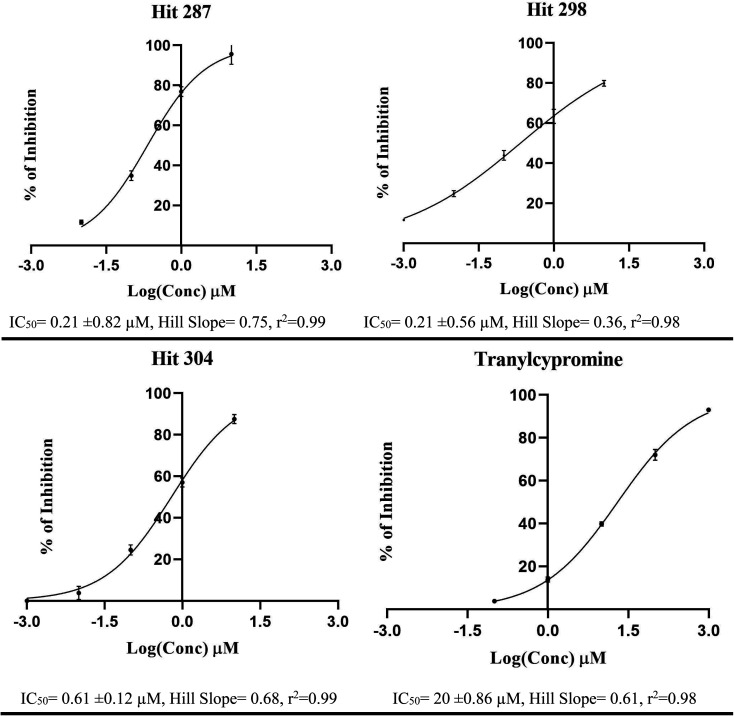
Dose/response curves of prominant compounds 287, 298 and 304 against LSD-1 enzyme. Each point represents duplicate measurements.

**Fig. 11:**
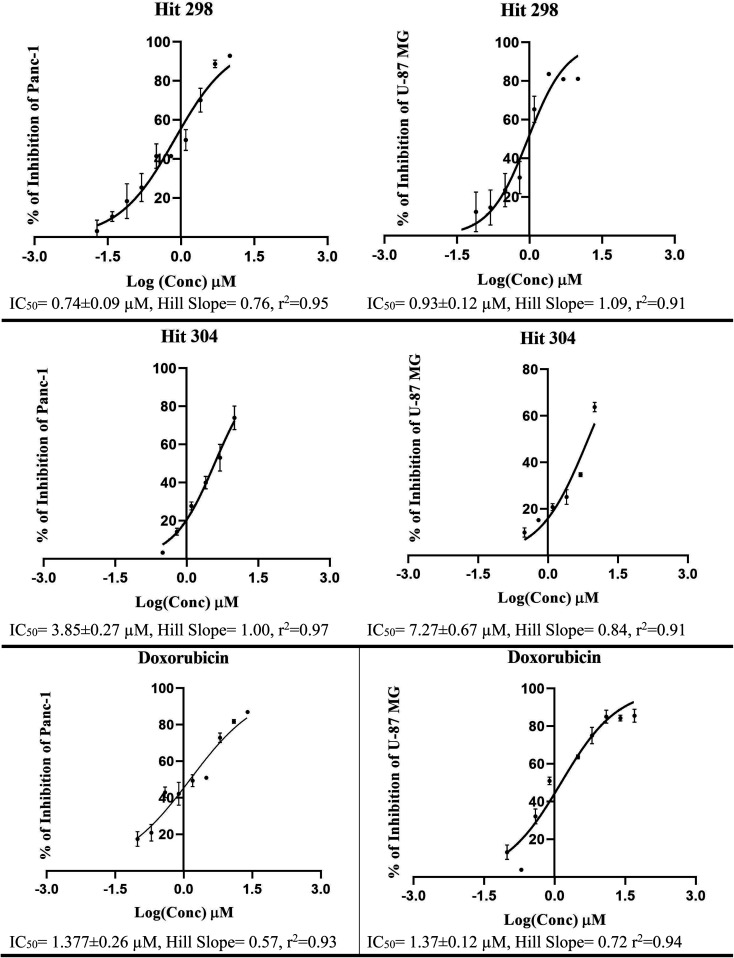
Dose/response curves of prominant compounds 298, 304 and doxorubicin (positive control) against Panc-1 and U-87 MG cancer cell lines. Each point represents triplicate measurements.

Bioassay results are shown in Table 5, Fig. 10 and 11. It can be concluded from the table and figures that hits 287, 298 and 304 are nanomolar inhibitors of LSD-1 with reasonable hill slopes^121^ and consistent cytotoxic effects against tested cancer cell lines, indicating they are authentic LSD-1 inhibitors (*i.e.*, non-promiscuous inhibitors).^121^ However, 287 showed poor cytotoxic properties against Panc-1 and U-87 MG cells probably because they are generally resistant to cytotoxic compounds.^122,123^ Interestingly, though, 287 seems to be the least toxic to normal fibroblasts (HDF) with only 7% inhibition at 10 μM, while 298 and 304 showed more pronounced cytotoxicities against HDF suggesting they are involved in extra cytotoxic mechanisms other than LSD-1 inhibition. The structures and purities of the three hits (287, 298 and 304) were confirmed by NMR and mass spectrometry as shown in ESI Fig. SM4–SM8.†

**Table 5:** Inhibitory effects of potent hits against two cancer cell lines, human dermal fibroblasts (HDF) and LSD-1 enzyme

Cpd	% Inhibition at 10 μM			
	LSD-1 enzyme	Panc-1	U-87 MG	HDF
285	18	3	0	31
286	25	44	33	52 (IC_50_ = 8.33 ± 3.05 μM, *r*^2^ = 0.92, HS = 0.42, SI = 1.09) a^,^^b^^,^^c^
287	96 (IC_50_ = 0.21 ± 0.82 μM, *r*^2^ = 0.98, HS = 0.75)^a^^,^^b^	39	15	7
288	18	41	47	35
298	78 (IC_50_ = 0.21 ± 0.56 μM, *r*^2^ = 0.93, HS = 0.36) a^,^^b^	93 (IC_50_ = 0.74 ± 0.09 μM, *r*^2^ = 0.95, HS = 0.76, SI = 24.6) a^,^^b^^,^^c^	81 (IC_50_ = 0.92 ± 0.12 μM, *r*^2^ = 0.91, HS = 1.09, SI = 30.6) a^,^^b^^,^^c^	69 (IC_50_ = 3.06 ± 0.76 μM, *r*^2^ = 0.95, HS = 0.88, SI = 102) a^,^^b^^,^^c^
304	88 (IC_50_ = 0.61 ± 0.12 μM, *r*^2^ = 0.96, HS = 0.70) a^,^^b^	73 (IC_50_ = 3.85 ± 0.27 μM, *r*^2^ = 0.97, HS = 1.00, SI = 2.6) a^,^^b^^,^^c^	64 (IC_50_ = 7.27 ± 0.67 μM, *r*^2^ = 0.91, HS = 0.84, SI = 2.5) a^,^^b^^,^^c^	68 (IC_50_ = 5.73 ± 0.29 μM, *r*^2^ = 0.83, HS = 2.23, SI = 15.48) a^,^^b^^,^^c^
317	32	30	32	24
325	29	93^d^	66^d^	86 (IC_50_ = 5.40 ± 0.16 μM, *r*^2^ = 0.94, HS = 2.25, SI = 1.7) a^,^^b^^,^^c^
Doxorubicin^e^	ND^e^	76 (IC_50_ = 1.377 ± 0.26 μM, *r*^2^ = 0.93, HS = 0.57)^a^^,^^b^	86 (IC_50_ = 1.37 ± 0.12 μM, *r*^2^ = 0.94, HS = 0.72)^a^^,^^b^	ND^f^
Tranylcypromine^g^	40 (IC_50_ = 20 ± 0.86 μM, *r*^2^ = 0.99, HS = 0.61)	ND^f^		

a *r* ^2^: regression coefficient of the dose–response curve.

b HS: hill Slope.

c SI: selectivity index in comparison with SH-SY5Y cells.

d Dose/response data is erratic, and it was not possible to construct consistent dose/response curve.

e Positive control for the cytotoxicity assays.

f Not determined.

g Positive control for the enzyme assay experiments.

**Fig. 12:**
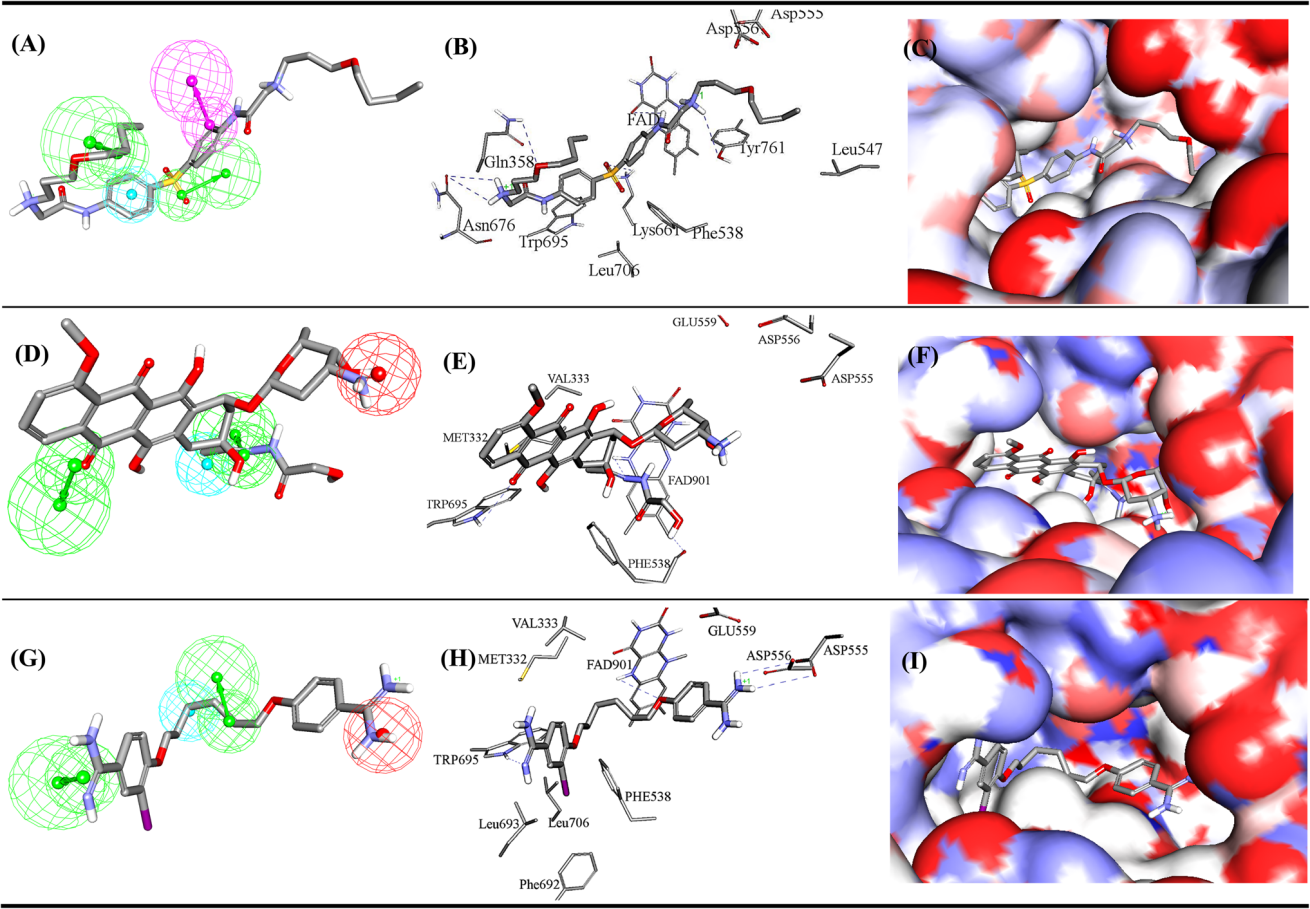
Mapping of most potent hits against corresponding capturing pharmacophores (A) 287 fitted against Hypo-LB5, (B) 287 docked into LSD1 (PDB code: 5LHI), (C) 287 docked into LSD1 with binding site covered with Connolly's surface, (D) 298 fitted against Hypo-SB1, (E) 298 docked into LSD1 (PDB code: 5LHI), (F) 298 docked into LSD1 with binding site covered with Connolly's surface. (G) 304 fitted against Hypo-SB1, (H) 304 docked into LSD1 (PDB code: 5LHI), (I) 304 docked into LSD1 with binding site covered with Connolly's surface. Chemical structures of the compounds are shown in Fig. SM3.†

Active hits 287, 298 and 304 are shown in Fig. 12. The figure also depicts how the three hits map their respective capturing pharmacophores and how they dock into the binding pocket of LSD-1. Clearly, the ligands interact favourably with some common amino acids such as: electrostatic interaction with Asp555 and Asp556, stacking against the aromatic rings of FAD and Phe538. For example, the carboxylate of Asp556 is situated at 6.8 Å from one of the cationic ammoniums of docked 287, suggesting noticeable mutual electrostatic attraction. Likewise, the cationic ammonium of 298 is docked at an average distance of 7.5 Å from the carboxylates of Asp555, Asp556 and Glu559, which also implies significant electrostatic attraction with these residues. However, one of the two amidines of docked 304 is positioned closest to the same carboxylate side chains, with a separating distance of only 5.1 Å from the COO- of Glu559 suggesting 304 to have the strongest electrostatic attraction to this anionic centre among other active hits.

The three hits seem to share hydrogen bonding interactions with FAD and the Trp695. Nevertheless, each hit seems to exhibit certain unique binding interactions: 287 exhibits extra hydrogen bonding interactions with Gln358, Lys661, Asn676 and Tyr761. While the iodo substituent of 304 fits within a hydrophobic pocket comprised of the hydrophobic side chains of Phe538, Phe692, Leu693 and Leu706. Accordingly, it can be concluded that each hit compound assumes different binding mode within the binding pocket.

**Fig. 13:**
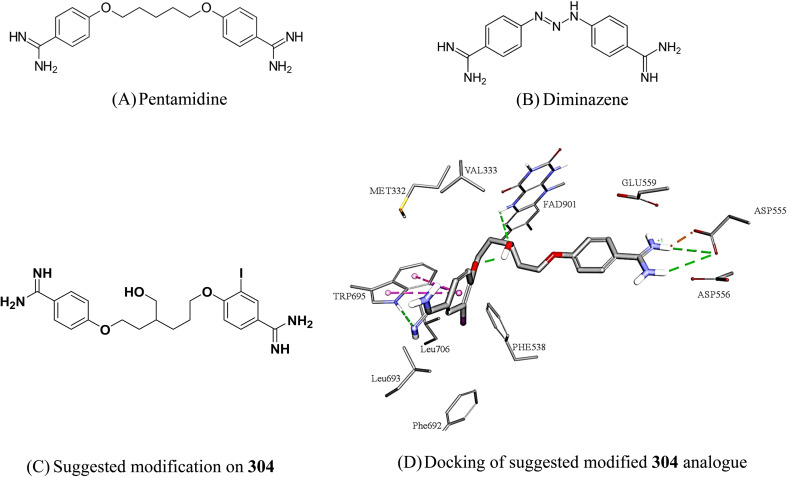
Structures of close medicinal analogues to 304 and suggested modification of 304. Dotted lines represent reversible binding interactions (green: hydrogen bonds, pink: pi stacking, brown: electrostatic attraction).

Based on similarity to known clinically approved medicinal compounds, we believe 304 to be the best candidate for future optimization towards potent clinically viable LSD1 inhibitors. Hit 304 is closely analogous to the clinically approved antiprotozoal pentamidine (Fig. 13a) and the veterinary antiprotozoal diminazene (Fig. 13b). Moreover, we believe it is possible to significantly enhance its affinity to LSD-1, and therefore reduce its dose and potential toxicity, by reducing the entropic cost of binding through rigidifying its aliphatic linker. We suggest introducing a hydroxyl group to the linker should promote intramolecular hydrogen bonding, as in Fig. 13c, which in turn should rigidify the linker moiety and provides better affinity. Additional related modifications can also be considered in future work.

## 4. Conclusion

As an epigenetic enzyme that is overexpressed in various malignancies, lysine-specific histone demethylase 1 (LSD-1) is thought to be a prospective therapeutic target for cancer treatment. This encouraged us to model this target *via* established computational workflows that involve machine learning-guided selection of ligand-based pharmacophore models (either supervised or unsupervised), or machine learning-guided selection of structure-based pharmacophore models (from either co-crystalized ligand or virtual docking of potent LSD-1 inhibitors). The performance of the resulting pharmacophore models was validated and evaluated by ROC curve analysis and SHAP assessment. Three pharmacophore models namely, Hypo-SB1, Hypo-LB5 and Hypo-LB6 were selected and subsequently used as virtual search queries to screen the National Cancer Institute (NCI) database for LSD-1 inhibitors with novel chemotypes. Eight hits: 285, 286, 287, 288, 298, 304, 317 and 385 showed promising LSD-1 inhibition upon testing against neuroblastoma SH-SY5Y cells. Further evaluation against other cancer cell lines (Panc-1 and U-87 MG) and *in vitro* LSD-1 enzymatic assay indicated nanomolar activities for 287, 298 and 304. The latter is particularly interesting because of its close analogy to the approved antiprotozoals pentamidine and diminazene. Additionally, we believe it can be readily optimized to become even more potent clinically approved LSD1 inhibitor.

## Conflicts of interest

There are no conflicts of interest to declare.

## Supplementary Material

RA-012-D2RA05102H-s001
