# Supplementary material for: Discovery of new potent lysine specific histone demythelase-1 inhibitors (LSD-1) using structure based and ligand based molecular modelling and machine learning
Source: RSC Adv. 2022 Dec 15;12(55):35873–95. doi: 10.1039/d2ra05102h (PMC9751883; doi:10.1039/d2ra05102h)
Supplement: RA-012-D2RA05102H-s001 [file RA-012-D2RA05102H-s001.pdf]

Electronic Supplementary Material (ESI) for RSC Advances.
This journal is © The Royal Society of Chemistry 2022



**Discovery of new potent Lysine Specific histone Demythelase-1 inhibitors (LSD-1) using Structure based, Ligand based molecular modeling and machine learning.**

**Shada J. Alabed[1], Malek Zihlif[2], Mutasem Taha[3]***

[1]Department of pharmacy, Faculty of Pharmacy, Al-Zaytoonah University of Jordan, Amman, Jordan. Email: Shada_alabed@yahoo.com.

[2]Department of Pharmacology, Faculty of Medicine, University of Jordan, Amman, Jordan. Email: m.zihlif@ju.edu.jo.

[3]Department of Pharmaceutical Sciences, Faculty of Pharmacy, University of Jordan, Amman, Jordan. Email: mutasem@ju.edu.jo.

*Corresponding Author.

**Supplementary table of contents**

| | |
|---|---|
| **Pages 2-32** | **Table SM1** |
| **Pages 33-36** | **Table SM2** |
| **Page 37** | **Figure SM1 and Section SM1** |
| **Pages 38-42** | **Sections SM2-SM4** |
| **Pages 43-44** | **Table SM3** |
| **Page 45** | **Section SM5** |
| **Page 46-47** | **Section SM6** |
| **Page 47** | **Table SM4** |
| **Page 48** | **Table SM5** |
| **Pages 48-50** | **Section SM7** |
| **Page 50** | **Section SM8** |
| **Pages 51-52** | **Table SM6** |
| **Pages 53-54** | **Section SM9** |
| **Pages 55-57** | **Table SM7** |
| **Page 58** | **Table SM8** |
| **Page 58** | **Section SM10** |
| **Page 59** | **Table SM9** |
| **Pages 60-61** | **Section SM11** |
| **Page 61** | **Section SM12** |
| **Page 62** | **Section SM13** |
| **Pages 62-63** | **Section SM14** |
| **Page 66** | **Figure SM2** |
| **Pages 67-80** | **Figure SM3** |
| **Page 81** | **Figure SM4** |
| **Page 82** | **Figure SM5** |
| **Page 83** | **Figure SM6** |
| **Page 84** | **Figure SM7** |
| **Page 85** | **Figure SM8** |
| **Pages 86-134** | **Table SM10** |
| **Pages 135-159** | **Table SM11** |

**Table SM1:** The structures of LSD-1 inhibitors utilized in Pharmacophore hypothesis generation.

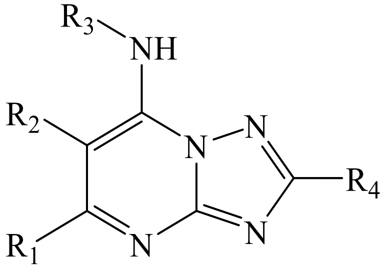


| Cpd | $R_1$ | $R_2$ | $R_3$ | $R_4$ | $IC_{50}$ (µM) | Reference |
|---|---|---|---|---|---|---|
| **1** | -Methyl | -H | 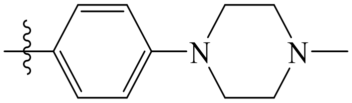 | -H | 31.35 | [19] |
| **2** | -Ethyl | -H | 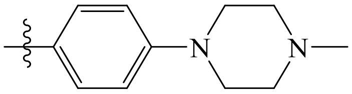 | -H | 38.13 | [19] |
| **3** | -Phenyl | -H | 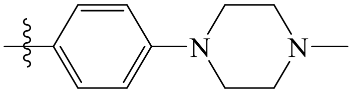 | -H | >50 | [19] |
| **4** | 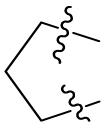 | | 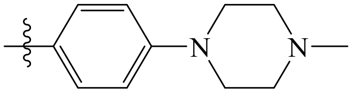 | 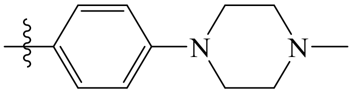 | >50 | [19] |
| **5** | -Methyl | -H | 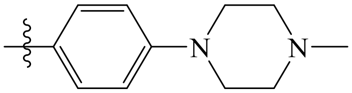 | 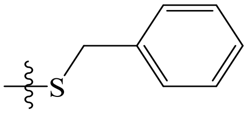 | 12.39 | [19] |
| **6** | -Methyl | -H | 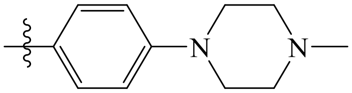 | 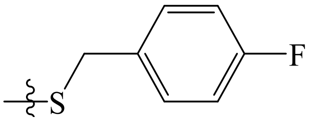 | 18.51 | [19] |
| **7** | -Methyl | -H | 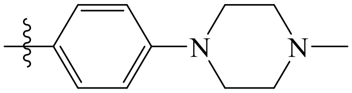 | 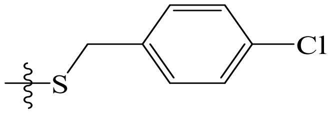 | 22.86 | [19] |
| **8** | -Methyl | -H | 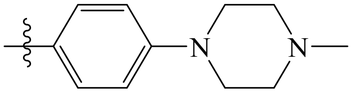 | 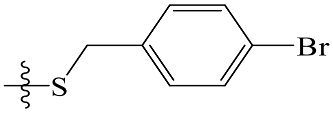 | 23.09 | [19] |
| **9** | -Methyl | -H | 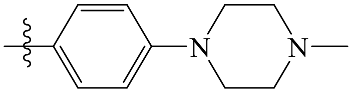 | 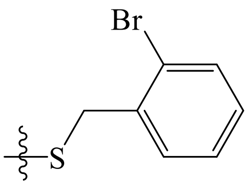 | 20.85 | [19] |
| **10** | -Methyl | -H | 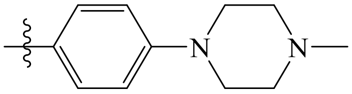 | 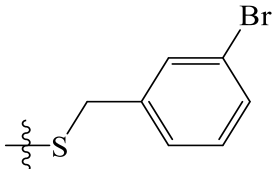 | 29.24 | [19] |
| **11** | -Methyl | -H | 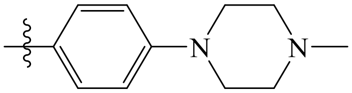 | 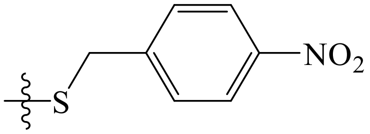 | 32.56 | [19] |

| Cpd | R1 | R2 | R3 | R4 | IC50(μM) | Reference |
|---|---|---|---|---|---|---|
| **12** | -Methyl | -H | 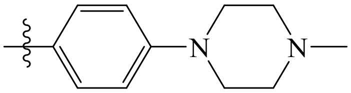 | 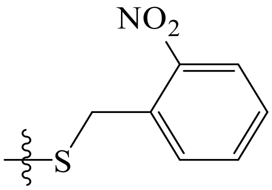 | >50 | 19 |
| **13** | -Methyl | -H | 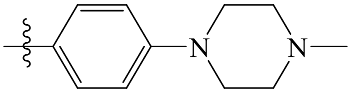 | 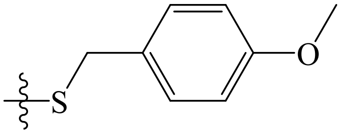 | 17.38 | 19 |
| **14** | -Methyl | -H | 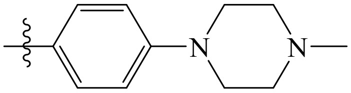 | 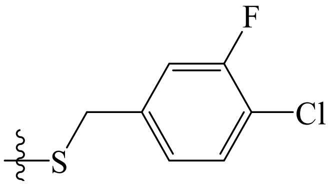 | 21.20 | 19 |
| **15** | -Methyl | -H | 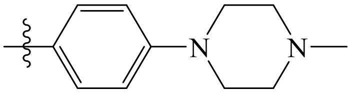 | 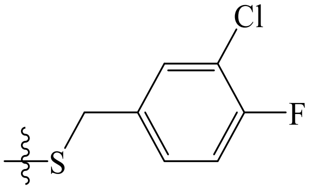 | 25.75 | 19 |
| **16** | -Methyl | -H | 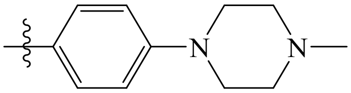 | 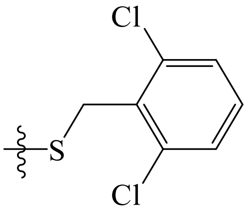 | 19.85 | 19 |
| **17** | -Methyl | -H | 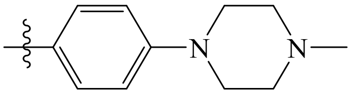 | 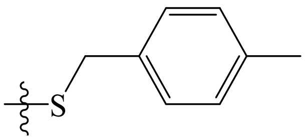 | 25.78 | 19 |
| **18** | -Methyl | -H | 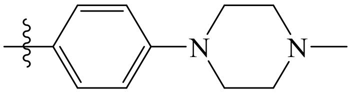 | 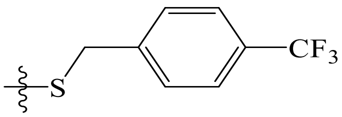 | 14.25 | 19 |
| **19** | -Methyl | -H | 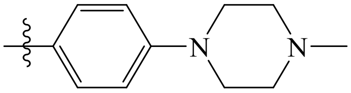 | 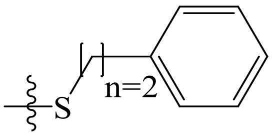 | 28.13 | 19 |
| **20** | -Methyl | -H | 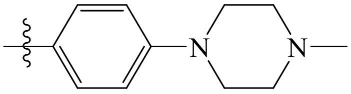 | 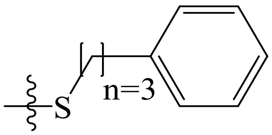 | >50 | 19 |
| **21** | -Methyl | -H | 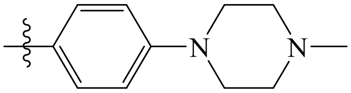 | 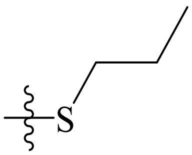 | 28.43 | 19 |
| **22** | -Methyl | -H | 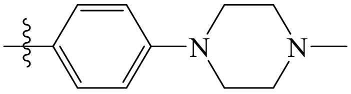 | 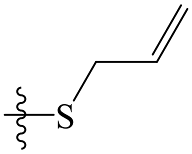 | 30.14 | 19 |

| Cpd | R1 | R2 | R3 | R4 | $IC_{50}$(μM) | Reference |
|---|---|---|---|---|---|---|
| **23** | -Methyl | -H | 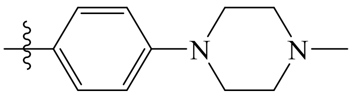 | 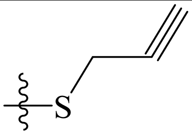 | 14.84 | [19] |
| **24** | -Methyl | -H | 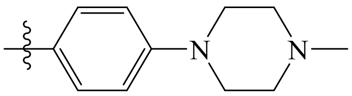 | 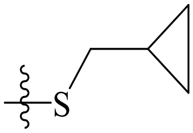 | >50 | [19] |
| **25** | -Methyl | -H | 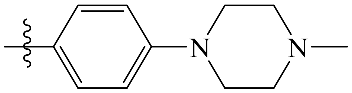 | 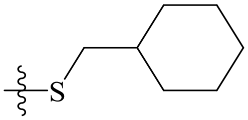 | 24.42 | [19] |
| **26** | -Methyl | -H | 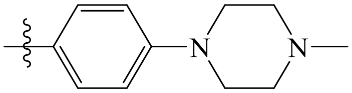 | 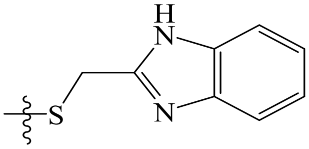 | 1.72 | [19] |
| **27** | -Methyl | -H | 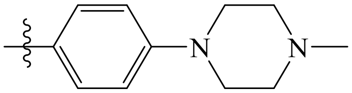 | 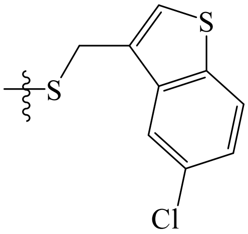 | 16.17 | [19] |
| **28** | -Methyl | -Methyl | 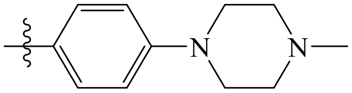 | 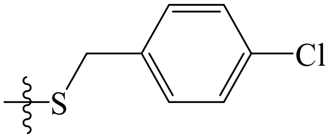 | >50 | [19] |
| **29** | -Methyl | $-C_5H_{11}$ | 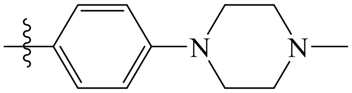 | 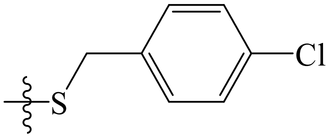 | >50 | [19] |
| **30** | -Methyl | -H | 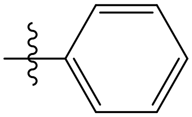 | 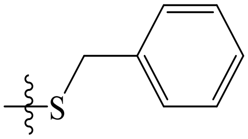 | >50 | [19] |
| **31** | -Methyl | -H | -H | 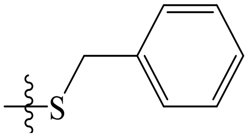 | >50 | [19] |
| **32** | -Methyl | -H | 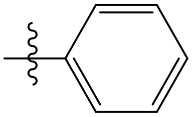 | 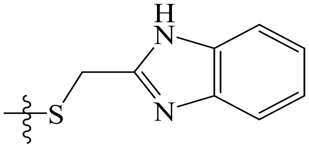 | >50 | [19] |

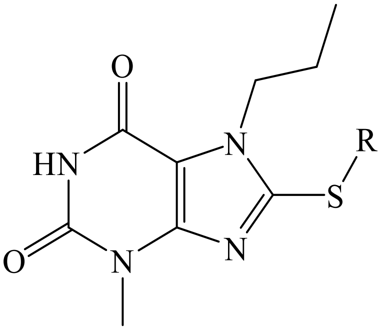


| Cpd | R | $IC_{50}$(μM) | Reference |
|---|---|---|---|
| **33** | -H | 6.45 | 32 |
| **34** | 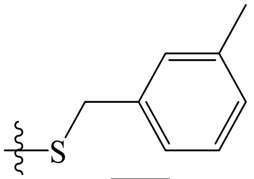 | 8.89 | 32 |
| **35** | 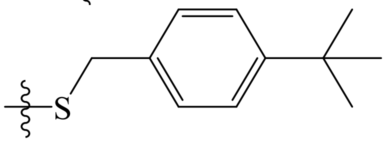 | 53.0 | 32 |
| **36** | 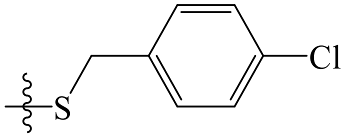 | 25.66 | 32 |
| **37** | 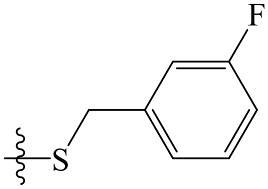 | 8.98 | 32 |
| **38** | 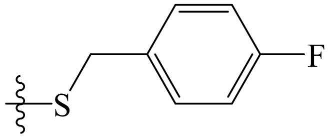 | 14.2 | 32 |
| **39** | 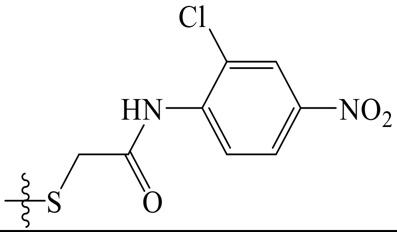 | 80.67 | 32 |

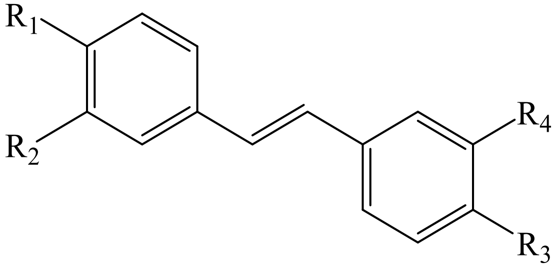

| Cpd | $R_1$ | $R_2$ | $R_3$ | $R_4$ | $IC_{50}$(μM) | Reference |
|---|---|---|---|---|---|---|
| **40** | -OH | 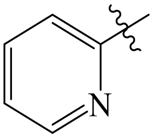 | -H | -OH | 4.24 | [29] |
| **41** | -OH | 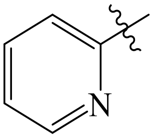 | -H | 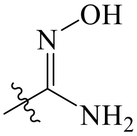 | 0.72 | [29] |
| **42** | -OH | 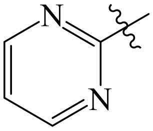 | -H | 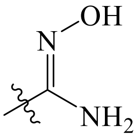 | 1.29 | [29] |
| **43** | -OH | 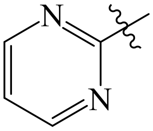 | 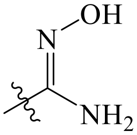 | -H | 0.92 | [29] |
| **44** | -OH | 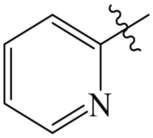 | -H | $-NH_2$ | 0.301 | [29] |
| **45** | -OH | 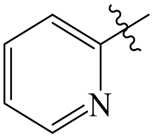 | $-NH_2$ | -H | 3.57 | [29] |
| **46** | -OH | 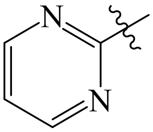 | -H | $-NH_2$ | 0.859 | [29] |
| **47** | -OH | 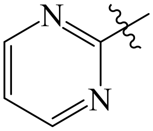 | $-NH_2$ | -H | 9.55 | [29] |
| **48** | -OH | 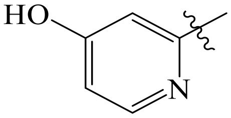 | -H | $-NH_2$ | 1.47 | [29] |

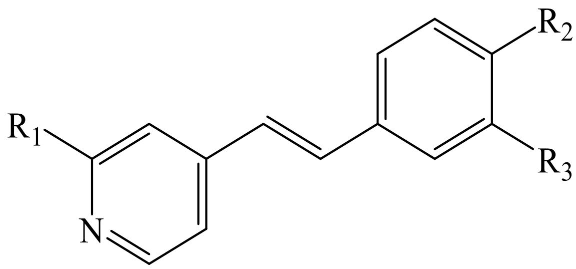


| Cpd | R₁ | R₂ | R₃ | IC₅₀(μM) | Reference |
|---|---|---|---|---|---|
| **49** | 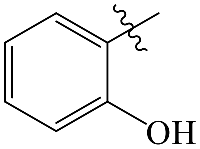 | -H | 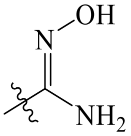 | 0.364 | [29] |
| **50** | 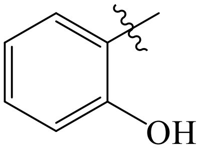 | 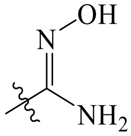 | -H | 0.764 | [29] |
| **51** | 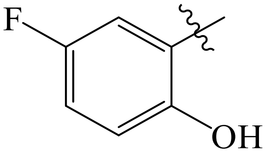 | -H | 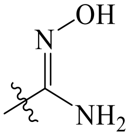 | 0.283 | [29] |
| **52** | 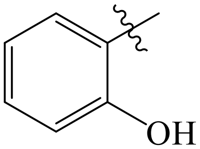 | $-NH_2$ | -H | 2.96 | [29] |
| **53** | 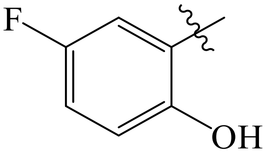 | $-NH_2$ | -H | 9.03 | [29] |
| **54** | 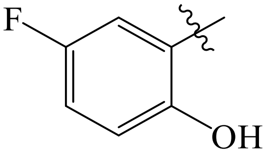 | -H | $-NH_2$ | 11.78 | [29] |

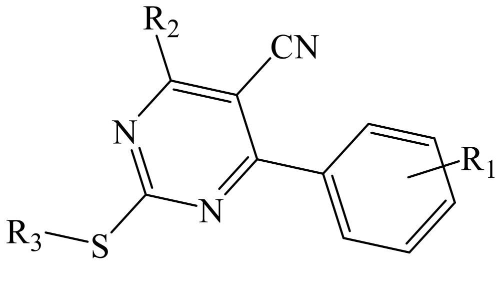


| Cpd | R₁ | R₂ | R₃ | IC₅₀(μM) | Reference |
|---|---|---|---|---|---|
| **55** | -H | 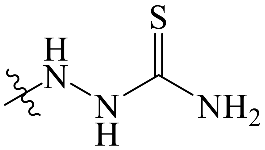 | 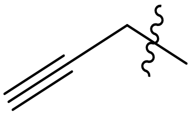 | 1.59 | [18] |

| Cpd | R1 | R2 | R3 | IC50(μM) | Reference |
|---|---|---|---|---|---|
| **56** | -*m*,*p*,*m*-*tri*$OCH_3$ | 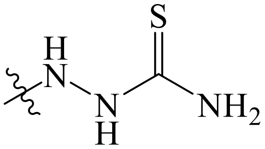 | 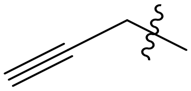 | 0.65 | [18] |
| **57** | -*p*-$CH_3$ | 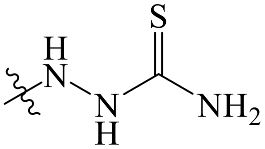 | 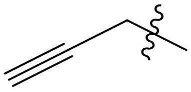 | 1.10 | [18] |
| **58** | -*p*-$CH(CH_3)_2$ | 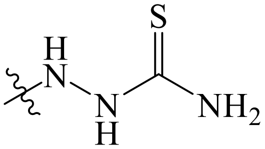 | 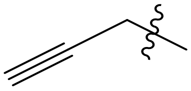 | 1.08 | [18] |
| **59** | -*p*-F | 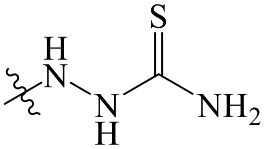 | 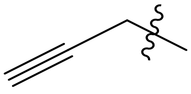 | 1.82 | [18] |
| **60** | -*p*-$NO_2$ | 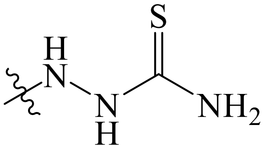 | 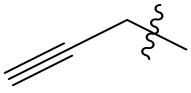 | 2.58 | [18] |
| **61** | -*p*-Cl | 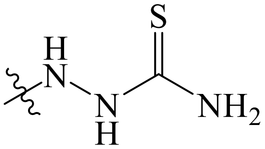 | 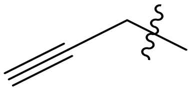 | 1.59 | [18] |
| **62** | -*m*,*p*-*di*F | 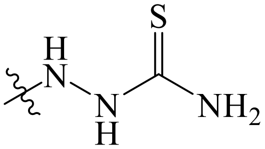 | 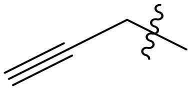 | 1.66 | [18] |
| **63** | -*p*-Br | 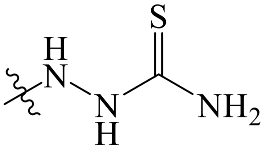 | 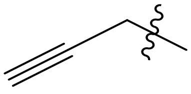 | 1.59 | [18] |
| **64** | -*m*-Cl | 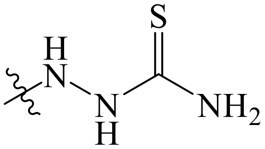 | 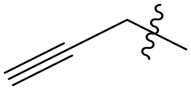 | 3.58 | [18] |
| **65** | -*m*-$OCH_3$ | 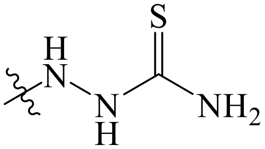 | 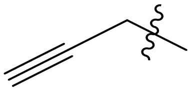 | 2.61 | [18] |
| **66** | -*p*-Cl | 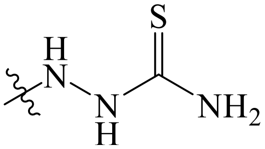 | 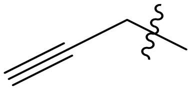 | 2.64 | [18] |
| **67** | -*m*,*p*-*di*F | 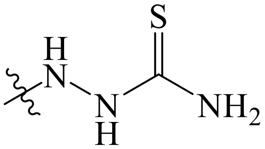 | 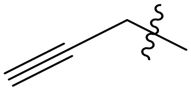 | 7.40 | [18] |

| Cpd | $R_1$ | $R_2$ | $R_3$ | $IC_{50}$(μM) | Reference |
|---|---|---|---|---|---|
| **68** | -*p*-$CH_3$ | 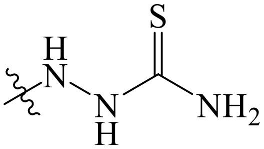 | 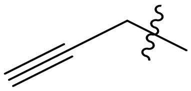 | 3.16 | [18] |
| **69** | -*p*-$CH(CH_3)_2$ | 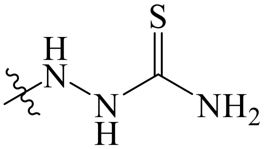 | 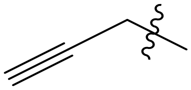 | 4.88 | [18] |
| **70** | -*m*-Cl | 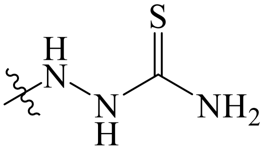 | 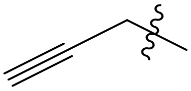 | 5.06 | [18] |
| **71** | -*p*-$NO_2$ | 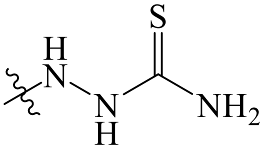 | 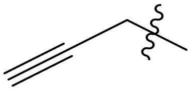 | 11.19 | [18] |
| **72** | -*p*-Br | 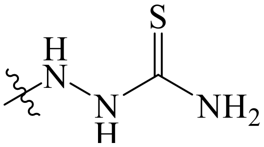 | 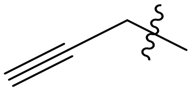 | 41.51 | [18] |
| **73** | -*p*-$NO_2$ | 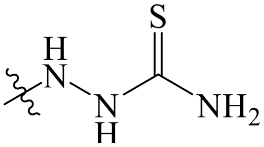 | 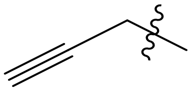 | 125 | [18] |
| **74** | -H | -Cl | 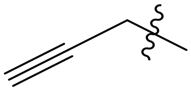 | 45.75 | [18] |
| **75** | -*m*,*p*,*m*-*tri*$OCH_3$ | -Cl | 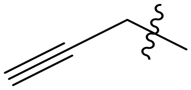 | 3.13 | [18] |
| **76** | -*p*-$CH_3$ | -Cl | 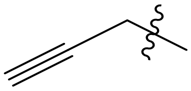 | 59.16 | [18] |
| **77** | -*p*-$CH(CH_3)_2$ | -Cl | 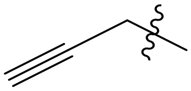 | 26.48 | [18] |
| **78** | -*p*-F | -Cl | 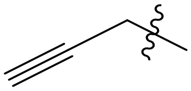 | 21.82 | [18] |
| **79** | -*p*-$NO_2$ | -Cl | 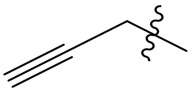 | 125 | [18] |
| **80** | -*p*-Cl | -Cl | 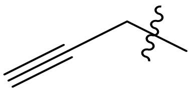 | 8.33 | [18] |
| **81** | -*m*,*p*-*di*F | -Cl | 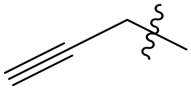 | 24.37 | [18] |

| Cpd | $R_1$ | $R_2$ | $R_3$ | $IC_{50}$(μM) | Reference |
|---|---|---|---|---|---|
| **82** | -*p*-Br | -Cl | 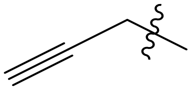 | 24.71 | 18 |
| **83** | -*m*-Cl | -Cl | 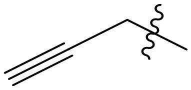 | 125 | 18 |
| **84** | -H | 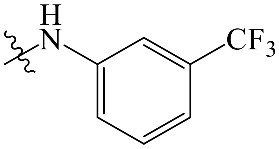 | 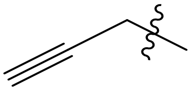 | 125 | 18 |
| **85** | -H | 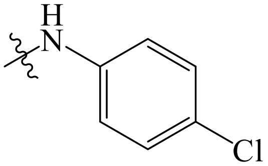 | 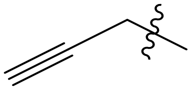 | 125 | 18 |
| **86** | -H | 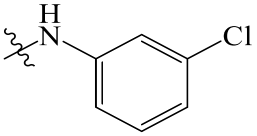 | 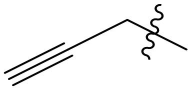 | 125 | 18 |
| **87** | -H | 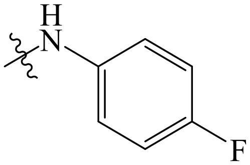 | 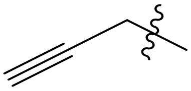 | 125 | 18 |
| **88** | -H | 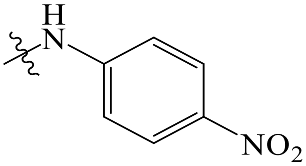 | 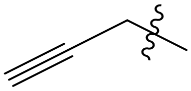 | 125 | 18 |
| **89** | -H | 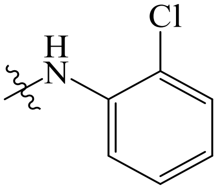 | 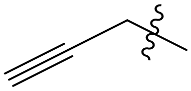 | 125 | 18 |
| **90** | -H | 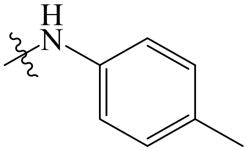 | 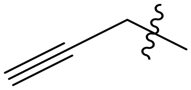 | 125 | 18 |
| **91** | -H | 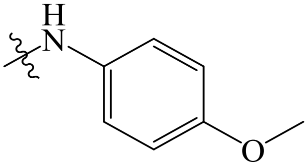 | 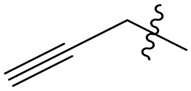 | 125 | 18 |
| **92** | -*p*-Cl | 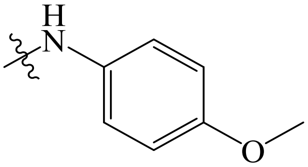 | 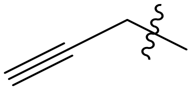 | 125 | 18 |

| Cpd | R1 | R2 | R3 | IC50(μM) | Reference |
|---|---|---|---|---|---|
| **93** | -*p*-Cl | 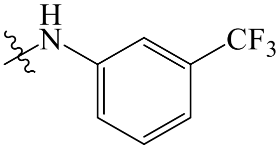 | 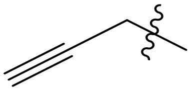 | 125 | [18] |
| **94** | -*p*-Br | 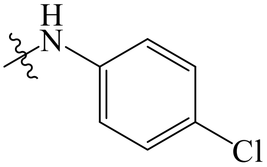 | 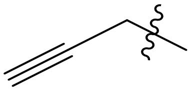 | 125 | [18] |
| **95** | -*m*,*p*,*m*-*tri*$OCH_3$ | 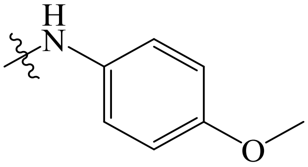 | 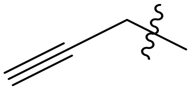 | 125 | [18] |
| **96** | -*m*,*p*,*m*-*tri*$OCH_3$ | 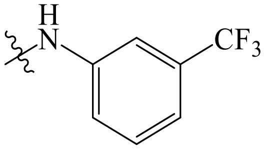 | 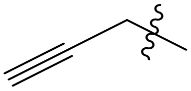 | 125 | [18] |
| **97** | -H | -Cl | 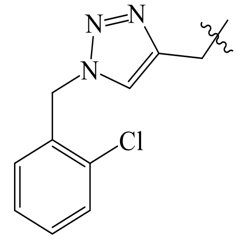 | 125 | [18] |
| **98** | -*p*-Cl | -Cl | 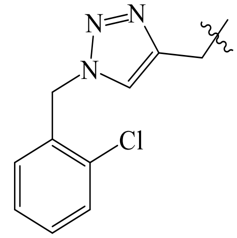 | 125 | [18] |
| **99** | -*m*,*p*,*m*-*tri*$OCH_3$ | 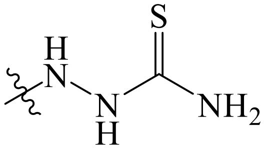 | 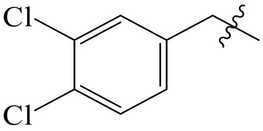 | 98.57 | [18] |
| **100** | -*m*,*p*,*m*-*tri*$OCH_3$ | 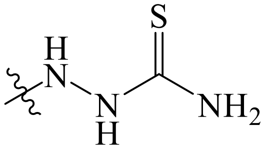 | 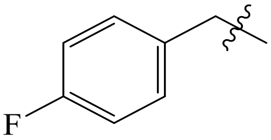 | 125 | [18] |

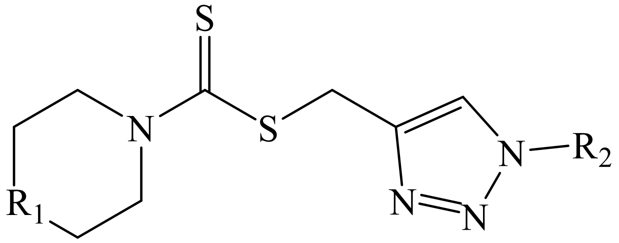

| Cpd | R$_1$ | R$_2$ | IC$_{50}$(μM) | Reference |
|---|---|---|---|---|
| **101** | 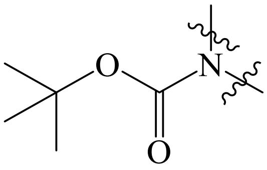 | 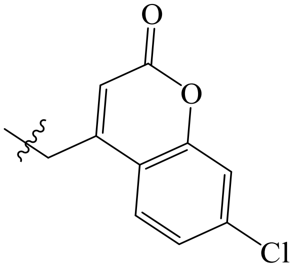 | 0.67 | [27] |
| **102** | 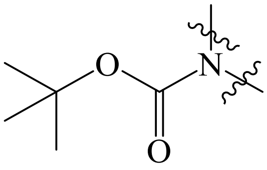 | 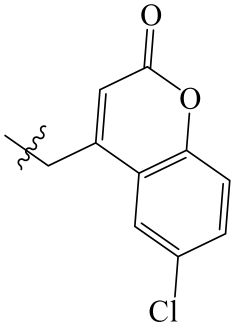 | 125 | [27] |
| **103** | 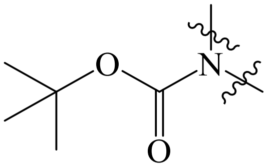 | 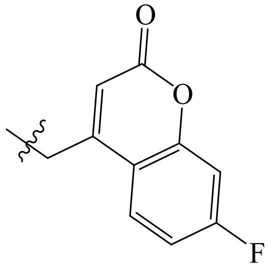 | 84.2 | [27] |
| **104** | 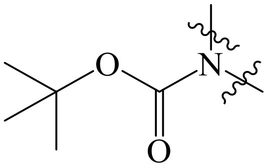 | 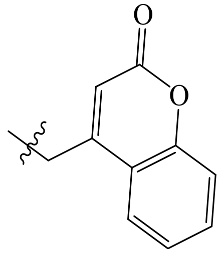 | 10.33 | [27] |
| **105** | 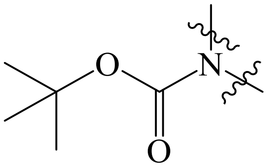 | 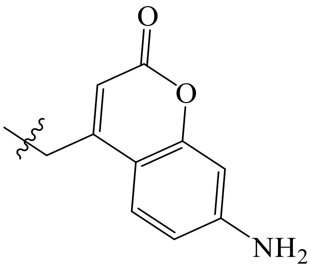 | 0.53 | [27] |

| Cpd | R1 | R2 | $IC_{50}$(μM) | Reference |
|---|---|---|---|---|
| **106** | 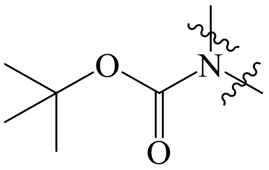 | 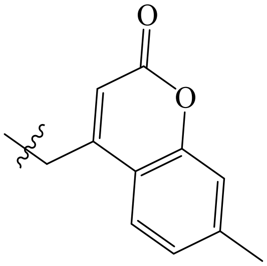 | 0.71 | [27] |
| **107** | 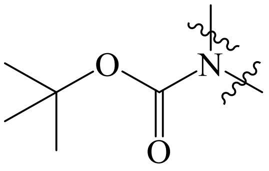 | 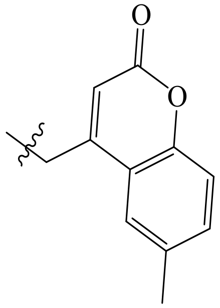 | 125 | [27] |
| **108** | 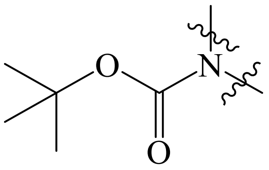 | 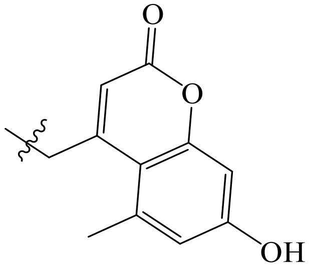 | 125 | [27] |
| **109** | 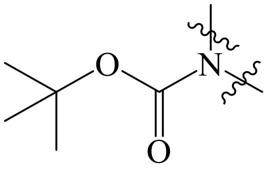 | 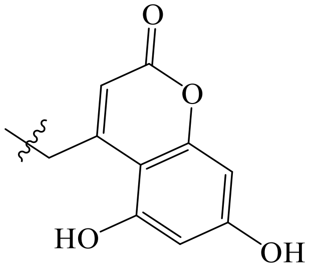 | 3.00 | [27] |
| **110** | 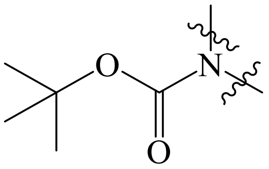 | 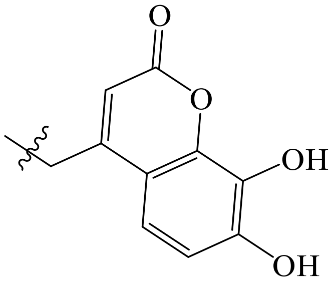 | 0.83 | [27] |
| **111** | 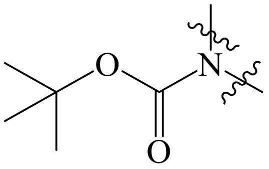 | 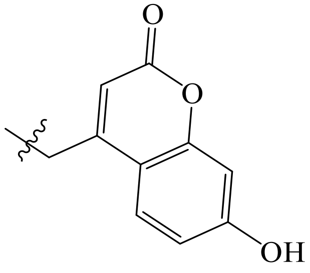 | 0.39 | [27] |

| Cpd | R₁ | R₂ | IC₅₀(µM) | Reference |
|---|---|---|---|---|
| **112** | 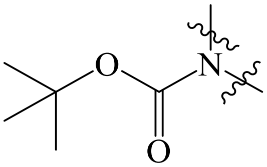 | 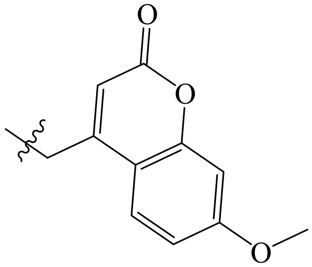 | 0.81 | 27 |
| **113** | 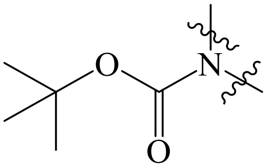 | 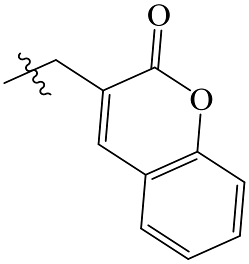 | 125 | 27 |
| **114** | 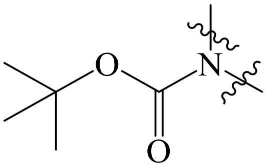 | 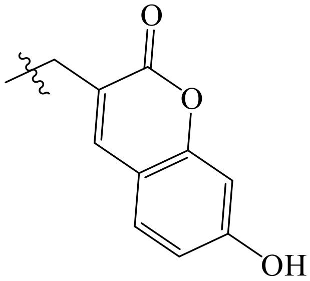 | 102.56 | 27 |
| **115** | $CH_2$ | 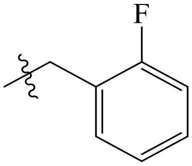 | 125 | 28 |
| **116** | $CH_2$ | 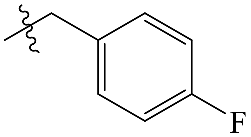 | 125 | 28 |
| **117** | $CH_2$ | 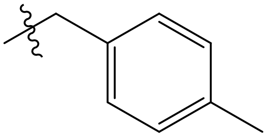 | 125 | 28 |
| **118** | $CH_2$ | 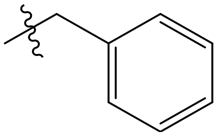 | 125 | 28 |
| **119** | $CH_2$ | 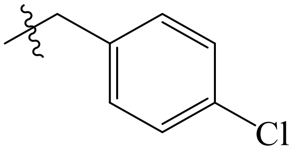 | 125 | 28 |
| **120** | O | 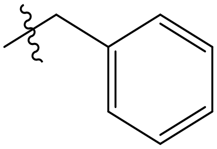 | 125 | 28 |

| Cpd | $R_1$ | $R_2$ | $IC_{50}$(µM) | Reference |
|---|---|---|---|---|
| **121** | O | 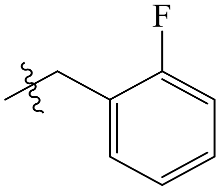 | 125 | [28] |
| **122** | O | 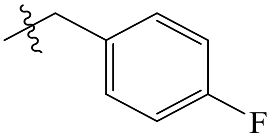 | 125 | [28] |
| **123** | O | 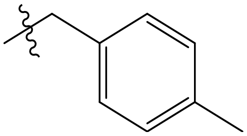 | 125 | [28] |
| **124** | O | 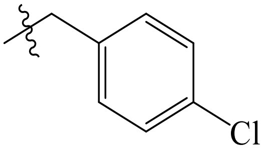 | 125 | [28] |
| **125** | 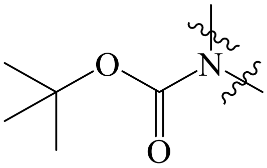 | 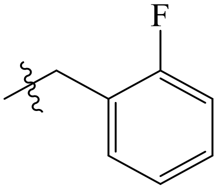 | 8.00 | [28] |
| **126** | 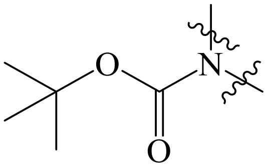 | 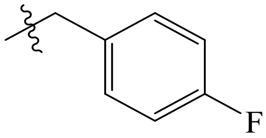 | 21.60 | [28] |
| **127** | 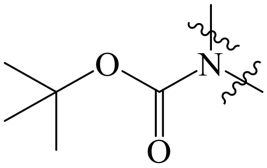 | 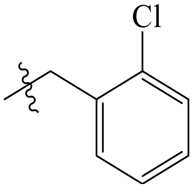 | 11.50 | [28] |
| **128** | 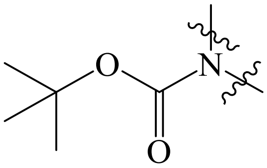 | 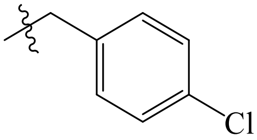 | 56.50 | [28] |
| **129** | 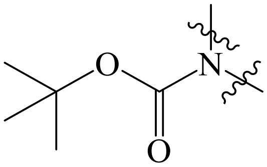 | 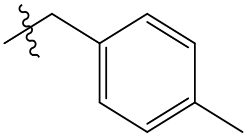 | 2.10 | [28] |
| **130** | 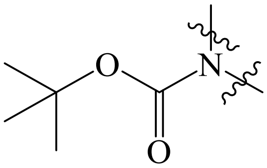 | 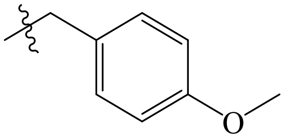 | 18.40 | [28] |
| **131** | 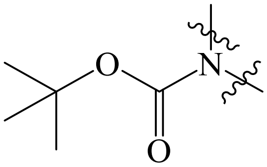 | 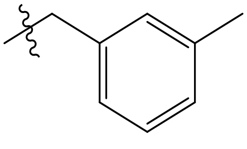 | 43.30 | [28] |

| Cpd | R$_1$ | R$_2$ | IC$_{50}$(μM) | Reference |
|---|---|---|---|---|
| **132** | 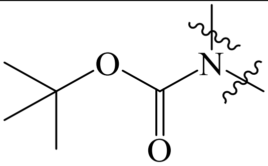 | 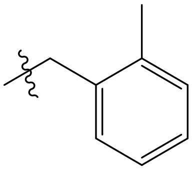 | 125 | [28] |
| **133** | 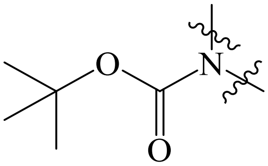 | 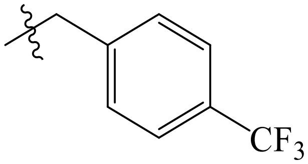 | 125 | [28] |
| **134** | 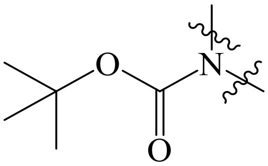 | 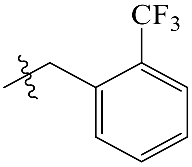 | 125 | [28] |
| **135** | 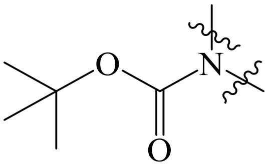 | 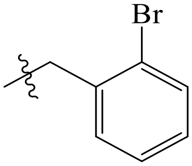 | 27.50 | [28] |
| **136** | 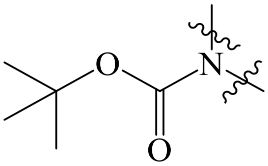 | 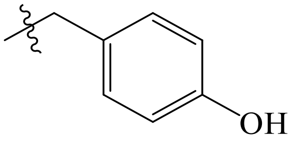 | 70.70 | [28] |
| **137** | 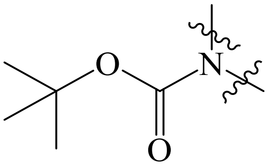 | 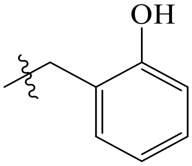 | 69.20 | [28] |
| **138** | 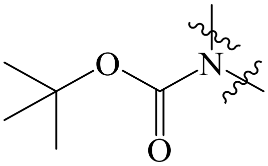 | 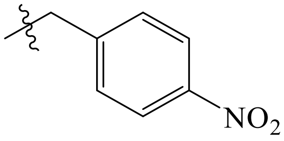 | 125 | [28] |
| **139** | 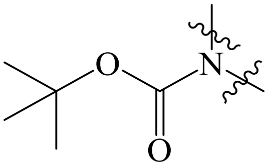 | 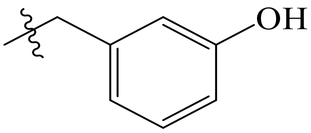 | 77.10 | [28] |
| **140** | 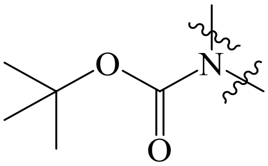 | 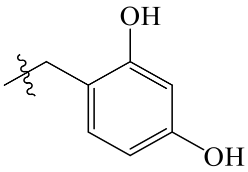 | 81.30 | [28] |
| **141** | 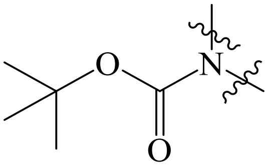 | 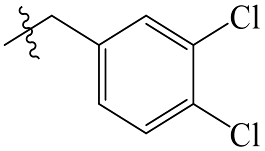 | 88.90 | [28] |

| Cpd | $R_1$ | $R_2$ | $IC_{50}$(μM) | Reference |
|---|---|---|---|---|
| **142** | 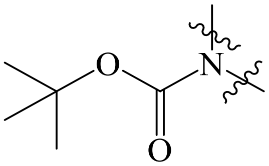 | 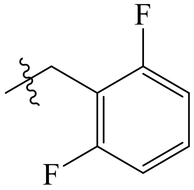 | 51.20 | [28] |
| **143** | 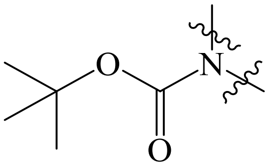 | 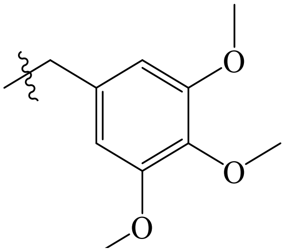 | 19.00 | [28] |
| **144** | 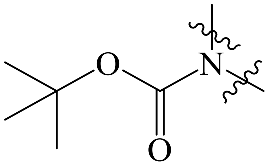 | 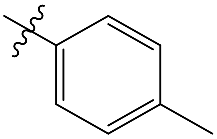 | 125 | [28] |
| **145** | 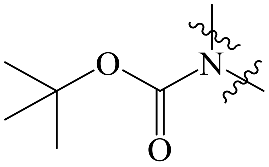 | 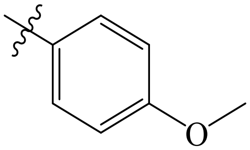 | 125 | [28] |
| **146** | 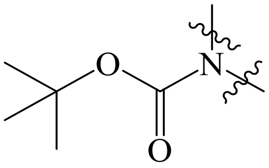 | 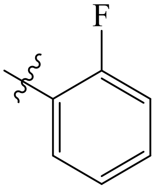 | 125 | [28] |
| **147** | 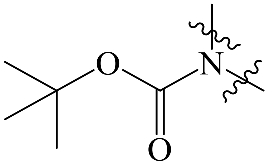 | 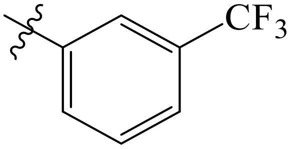 | 125 | [28] |
| **148** | 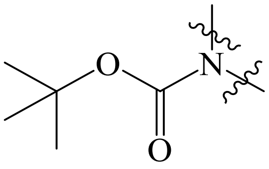 | 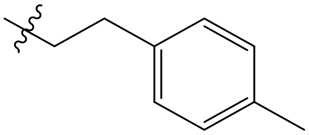 | 125 | [28] |
| **149** | 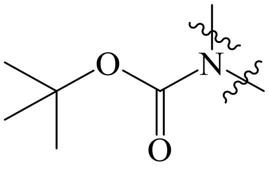 | 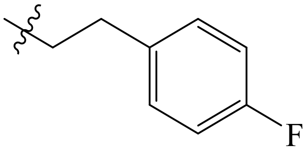 | 125 | [28] |
| **150** | 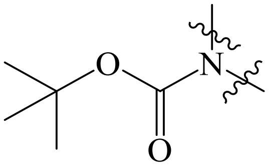 | 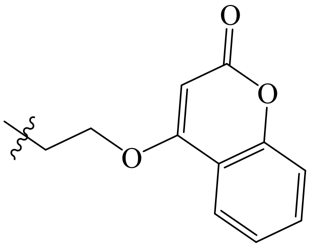 | 125 | [28] |

| Cpd | R1 | R2 | IC50(µM) | Reference |
|---|---|---|---|---|
| **151** | 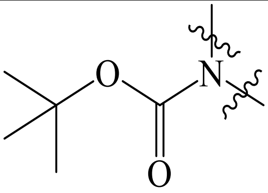 | 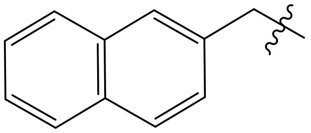 | 125 | [28] |
| **152** | 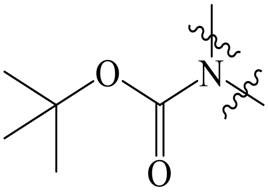 | 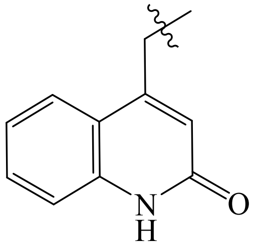 | 125 | [28] |
| **153** | 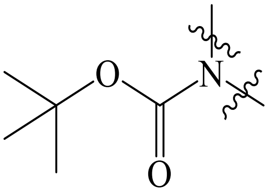 | 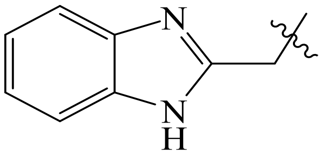 | 125 | [28] |
| **154** | 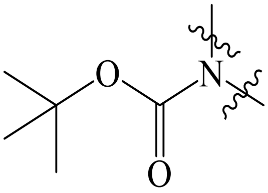 | 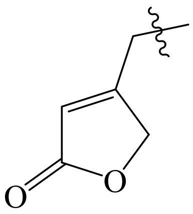 | 125 | [28] |
| **155** | 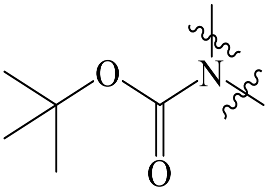 | 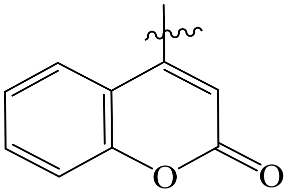 | 125 | [28] |
| **156** | 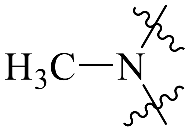 | 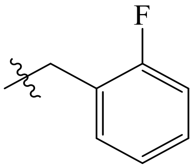 | 125 | [28] |
| **157** | 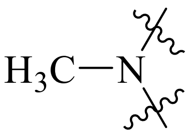 | 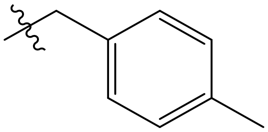 | 125 | [28] |
| **158** | 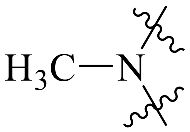 | 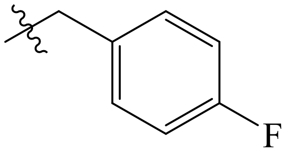 | 125 | [28] |
| **159** | 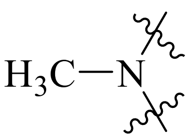 | 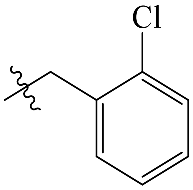 | 125 | [28] |
| **160** | 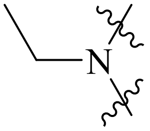 | 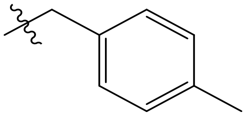 | 125 | [28] |

| Cpd | R1 | R2 | IC50(μM) | Reference |
|---|---|---|---|---|
| **161** | 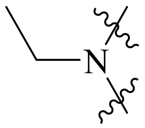 | 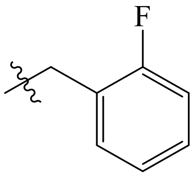 | 125 | [28] |
| **162** | 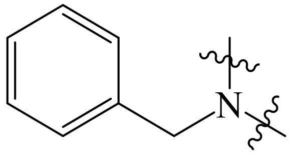 | 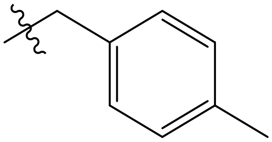 | 125 | [28] |
| **163** | 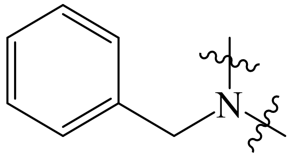 | 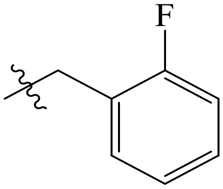 | 125 | [28] |
| **164** | 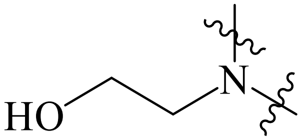 | 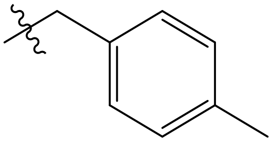 | 83.40 | [28] |
| **165** | 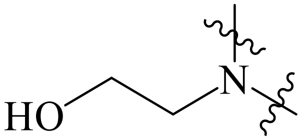 | 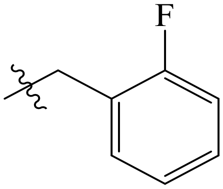 | 100.10 | [28] |
| **166** | 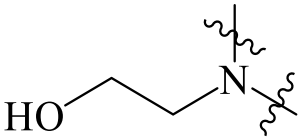 | 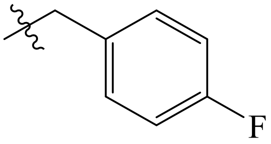 | 125 | [28] |
| **167** | 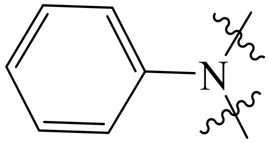 | 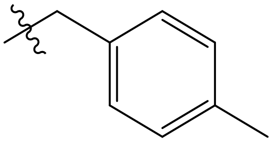 | 40.30 | [28] |
| **168** | 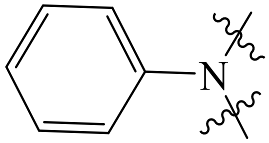 | 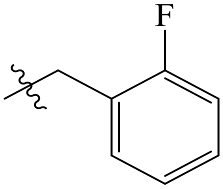 | 125 | [28] |
| **169** | 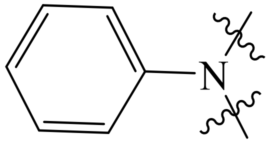 | 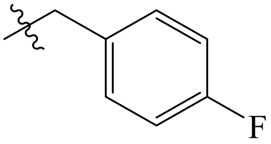 | 125 | [28] |
| **170** | 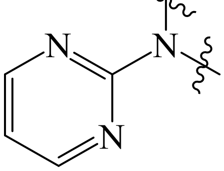 | 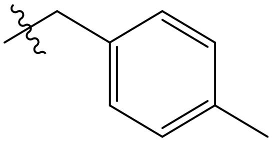 | 27.60 | [28] |

| Cpd | $R_1$ | $R_2$ | $IC_{50}$(µM) | Reference |
|---|---|---|---|---|
| **171** | 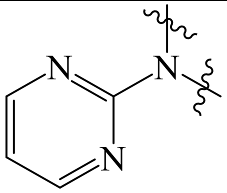 | 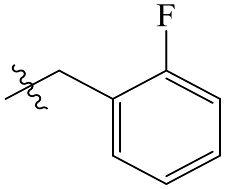 | 39.80 | [28] |
| **172** | 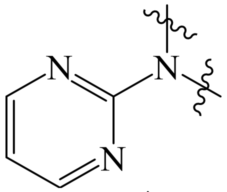 | 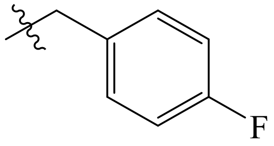 | 43.90 | [28] |
| **173** | 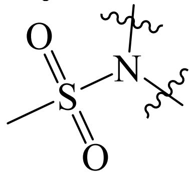 | 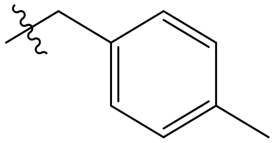 | 35.70 | [28] |
| **174** | 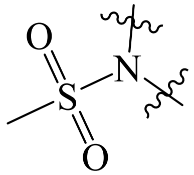 | 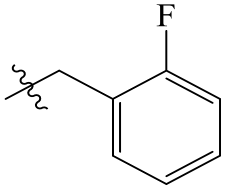 | 45.70 | [28] |
| **175** | 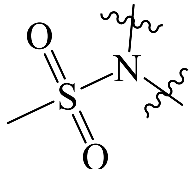 | 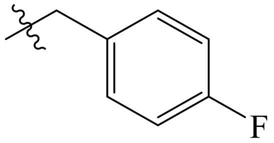 | 55.10 | [28] |
| **176** | NH | 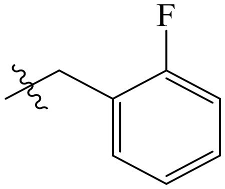 | 17.30 | [28] |
| **177** | NH | 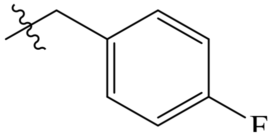 | 69.30 | [28] |
| **178** | NH | 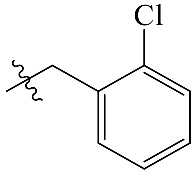 | 125 | [28] |
| **179** | NH | 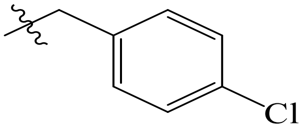 | 125 | [28] |
| **180** | NH | 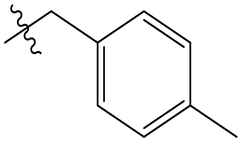 | 28.90 | [28] |

| Cpd | R1 | R2 | IC50(μM) | Reference |
|---|---|---|---|---|
| **181** | NH | 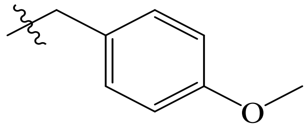 | 19.50 | [28] |
| **182** | NH | 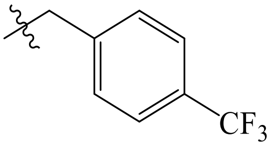 | 87.40 | [28] |
| **183** | NH | 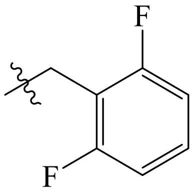 | 125 | [28] |
| **184** | NH | 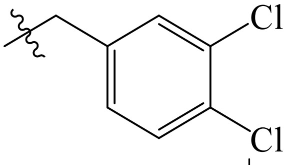 | 125 | [28] |
| **185** | NH | 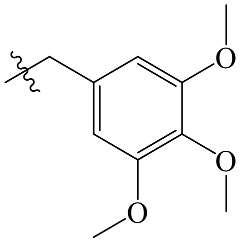 | 125 | [28] |
| **186** | 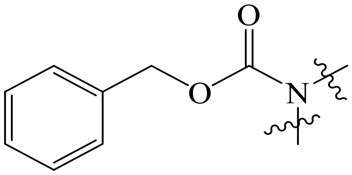 | 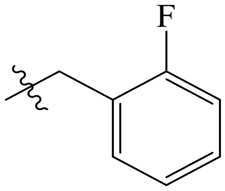 | 32.80 | [28] |
| **187** | 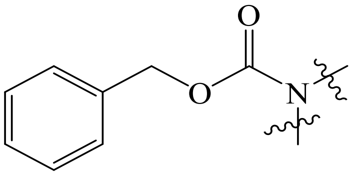 | 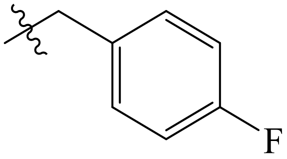 | 47.60 | [28] |
| **188** | 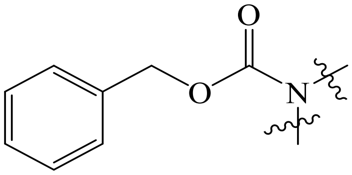 | 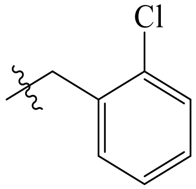 | 53.20 | [28] |
| **189** | 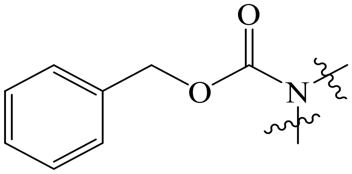 | 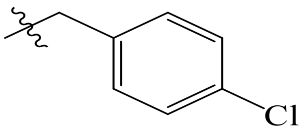 | 125 | [28] |

| Cpd | R1 | R2 | IC50(μM) | Reference |
|---|---|---|---|---|
| **190** | 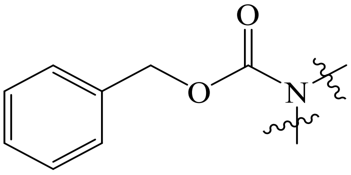 | 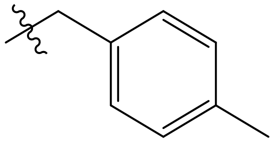 | 22.40 | [28] |
| **191** | 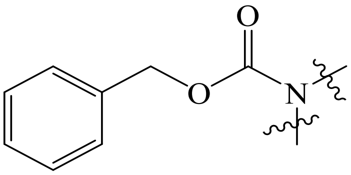 | 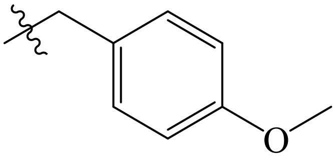 | 35.10 | [28] |
| **192** | 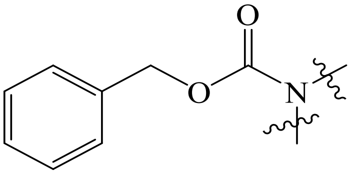 | 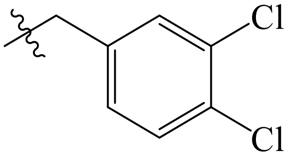 | 29.50 | [28] |
| **193** | 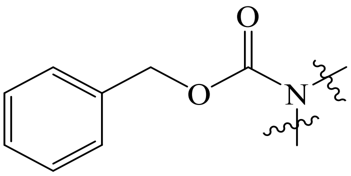 | 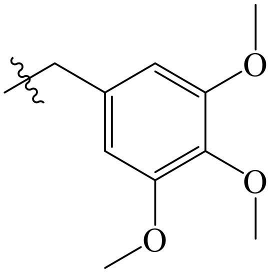 | 43.50 | [28] |
| **194** | 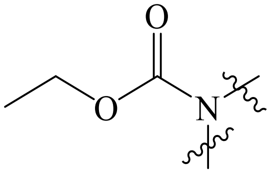 | 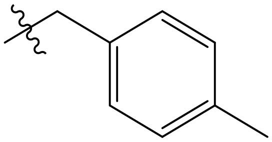 | 17.00 | [28] |
| **195** | 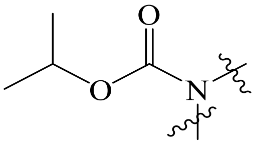 | 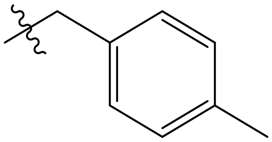 | 15.00 | [28] |
| **196** | 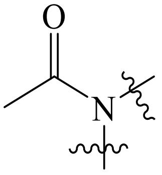 | 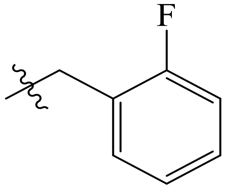 | 30.20 | [28] |
| **197** | 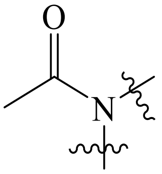 | 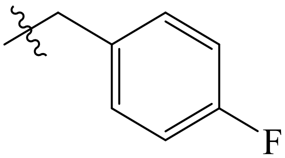 | 47.10 | [28] |

| Cpd | R₁ | R₂ | IC50(µM) | Reference |
|---|---|---|---|---|
| **198** | 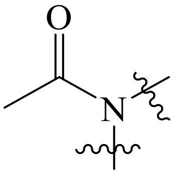 | 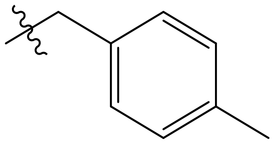 | 25.30 | 28 |

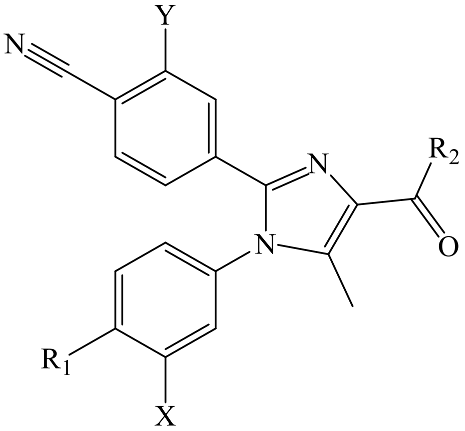


| Cpd | R₁ | X | Y | R₂ | IC50 (µM) | Reference |
|---|---|---|---|---|---|---|
| **199** | $-CH_3$ | -H | -H | 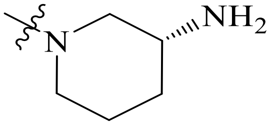 | 0.0080 | 35 |
| **200** | $-OCH_3$ | -H | -F | 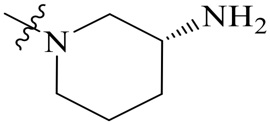 | 0.0040 | 35 |
| **201** | $-OCH_3$ | -F | -F | 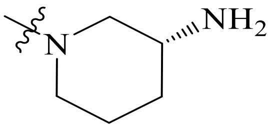 | 0.0070 | 35 |
| **202** | $-OCH_3$ | -F | -F | 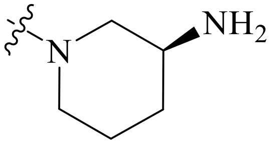 | 0.0180 | 35 |
| **203** | $-OCH_3$ | -F | -F | 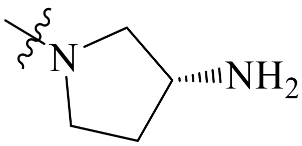 | 0.0170 | 35 |
| **204** | $-OCH_3$ | -F | -F | 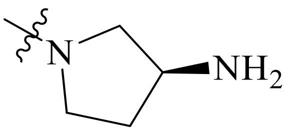 | 0.0040 | 35 |
| **205** | $-OCH_3$ | -F | -F | 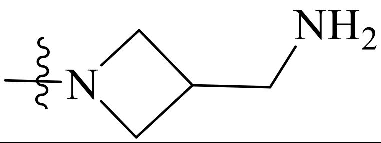 | 0.0100 | 35 |

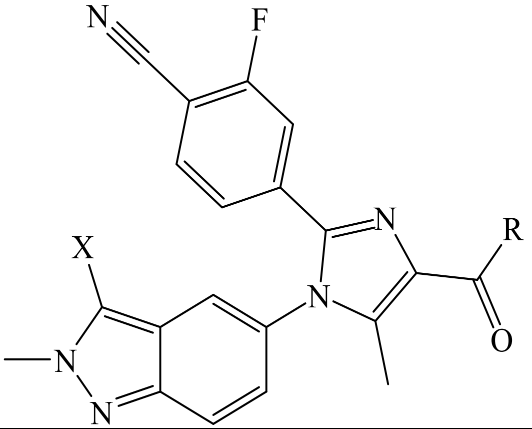


| Cpd | X | R | $IC_{50}$ (µM) | Reference |
|---|---|---|---|---|
| **206** | -H | 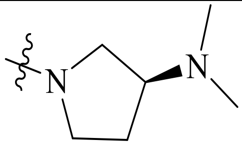 | 0.0070 | [35] |
| **207** | -Cl | 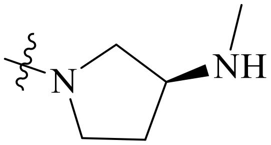 | 0.0007 | [35] |
| **208** | -Cl | 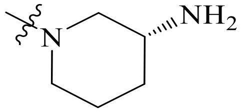 | 0.0020 | [35] |
| **209** | -Cl | 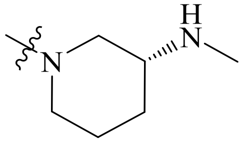 | 0.0010 | [35] |

| Cpd | Structure | $IC_{50}$ (µM) | Reference |
|---|---|---|---|
| **210** | 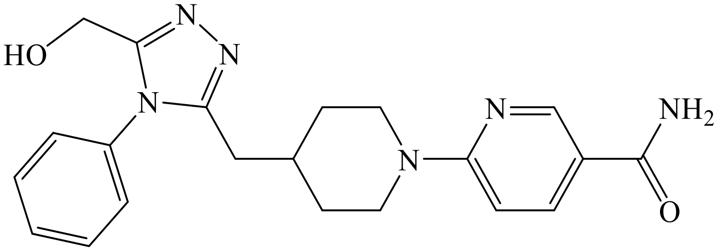 | 58.8000 | [31] |
| **211** | 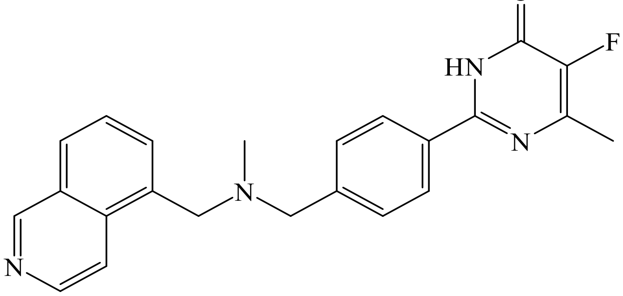 | 100.0000 | [31] |

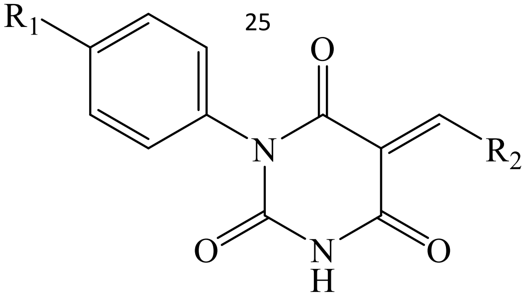

| Cpd | R₁ | R₂ | IC₅₀ (µM) | Reference |
|---|---|---|---|---|
| **212** | -Br | 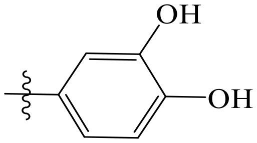 | 0.30 | [26] |
| **213** | $-OCH_3$ | 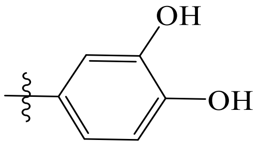 | 3.10 | [26] |
| **214** | -Br | 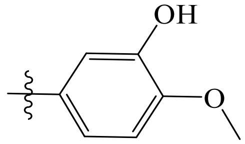 | 3.60 | [26] |
| **215** | -Br | 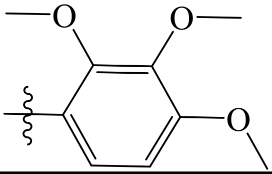 | 3.60 | [26] |

| Cpd | Structure | IC₅₀ (µM) | Reference |
|---|---|---|---|
| **216** | 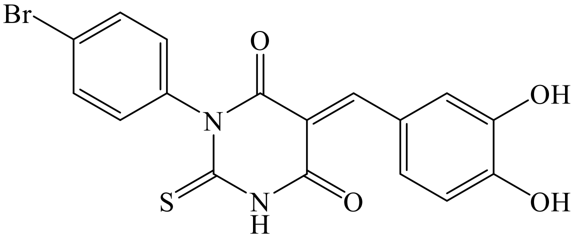 | 0.41 | [26] |
| **217** | 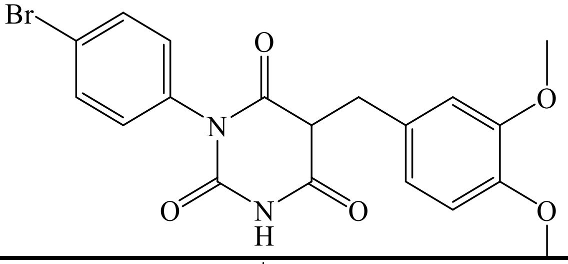 | 75.00 | [26] |
| **218** | 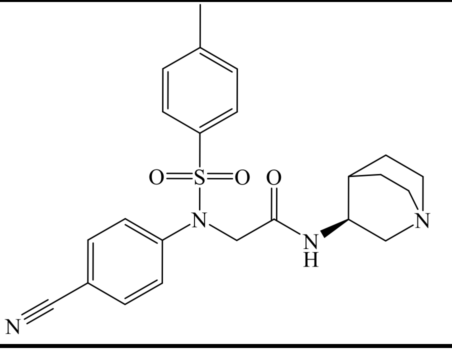 | 14.20 | [33] |

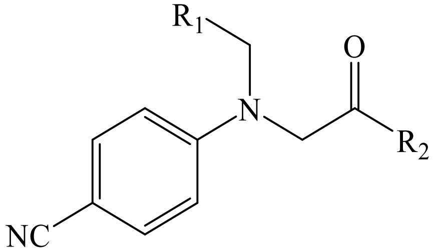

| Cpd | R1 | R2 | IC50 (µM) | Reference |
|---|---|---|---|---|
| **219** | 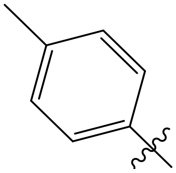 | 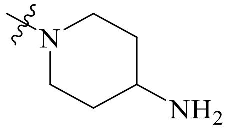 | 1.30 | [33] |
| **220** | 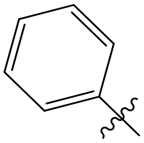 | 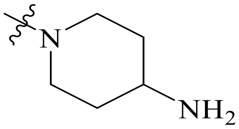 | 4.10 | [33] |
| **221** | 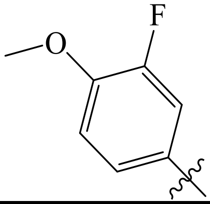 | 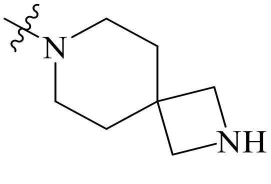 | 0.083 | [33] |

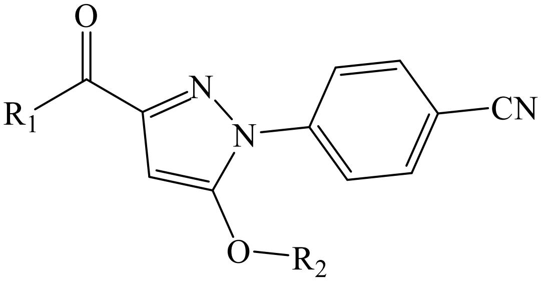

| Cpd | R1 | R2 | IC50 (µM) | Reference |
|---|---|---|---|---|
| **222** | 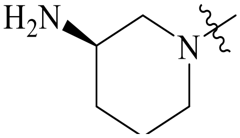 | 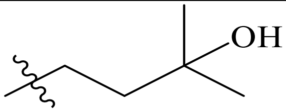 | 0.270 | [34] |
| **223** | 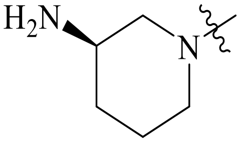 | 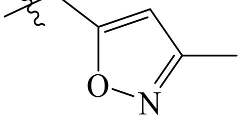 | 0.150 | [34] |
| **224** | 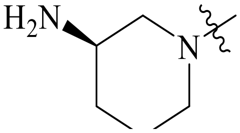 | 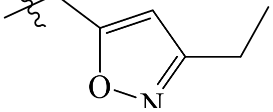 | 0.079 | [34] |
| **225** | 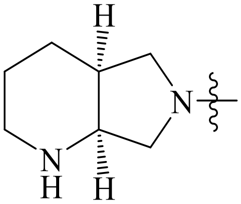 | 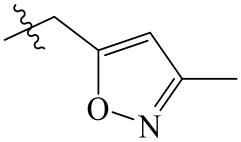 | 0.720 | [34] |

| Cpd | R1 | R2 | IC50 (µM) | Reference |
|---|---|---|---|---|
| **226** | 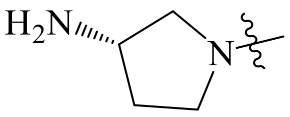 | 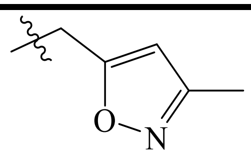 | 0.17 | 34 |

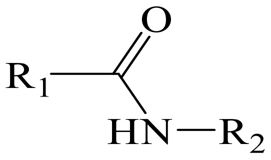

| Cpd | R1 | R2 | IC50 (µM) | Reference |
|---|---|---|---|---|
| **227** | 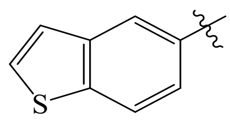 | 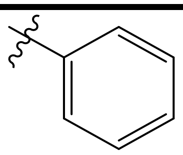 | 100.000 | 36 |
| **228** | 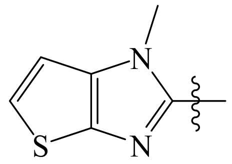 | 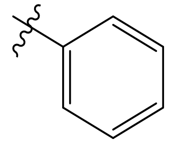 | 100.000 | 36 |
| **229** | 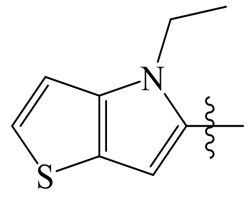 | 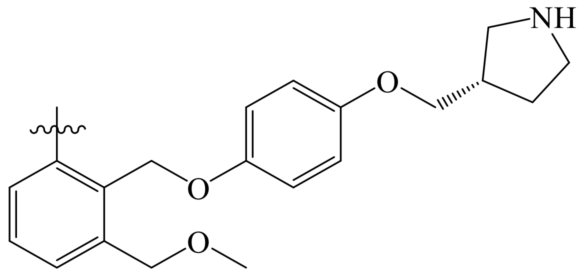 | 0.0067 | 24 |

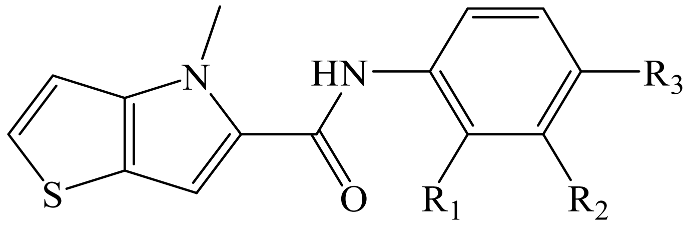

| Cpd | R1 | R2 | R3 | IC50 (µM) | Reference |
|---|---|---|---|---|---|
| **230** | -H | 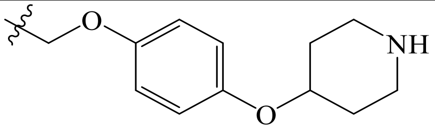 | -H | 0.162 | 36 |
| **231** | $-CH_2OCH_3$ | -H | -H | 100.000 | 24 |
| **232** | -H | -H | $-CH_2OCH_3$ | 100.000 | 24 |

| Cpd | R₁ | R₂ | R₃ | IC₅₀ (µM) | Reference |
|---|---|---|---|---|---|
| **233** | $-OCH_3$ | -H | -H | 100.000 | [24] |
| **234** | -H | -H | $-OCH_3$ | 100.000 | [24] |
| **235** | 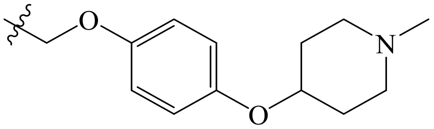 | -H | -H | 0.310 | [24] |
| **236** | 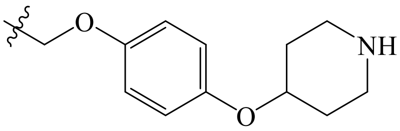 | -H | -H | 0.094 | [24] |
| **237** | 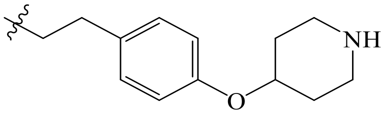 | -H | -H | 0.177 | [24] |
| **238** | 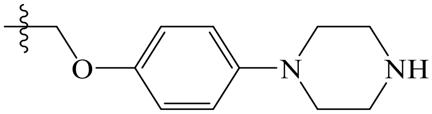 | -H | -H | 1.500 | [24] |
| **239** | 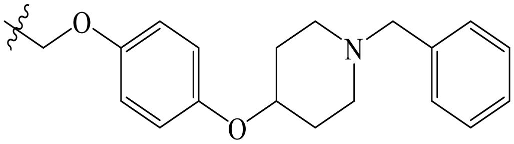 | -H | -H | 100.000 | [24] |
| **240** | 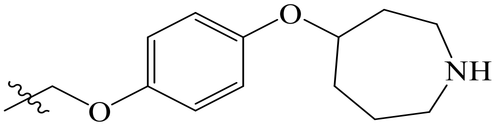 | -H | -H | 0.064 | [24] |
| **241** | 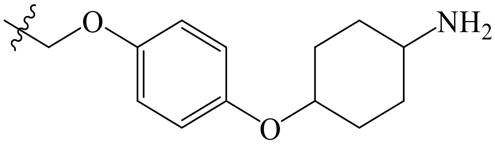 | -H | -H | 0.340 | [24] |
| **242** | 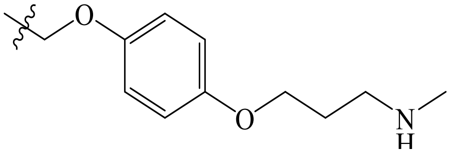 | -H | -H | 1.300 | [24] |
| **243** | 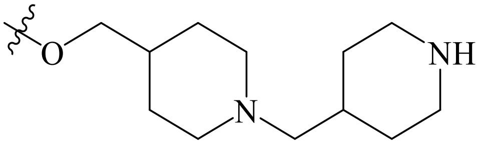 | -H | -H | 1.700 | [24] |
| **244** | 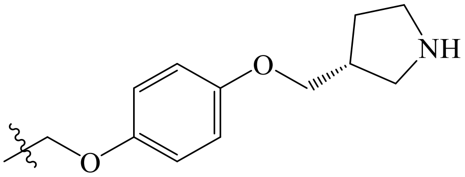 | $-CH_2OCH_3$ | -H | 0.0084 | [24] |
| **245** | 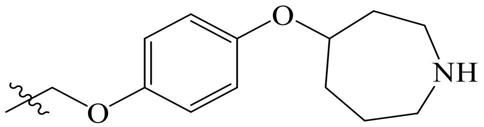 | $-CH_2OCH_3$ | -H | 0.0292 | [24] |

| Cpd | R1 | R2 | R3 | IC50 (µM) | Reference |
|---|---|---|---|---|---|
| **246** | 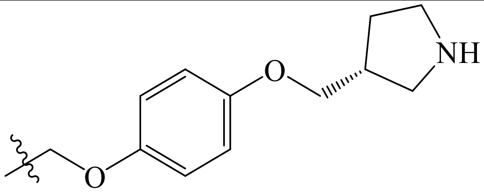 | $-CH_2OCH_2CH_3$ | -H | 0.0078 | [24] |
| **247** | 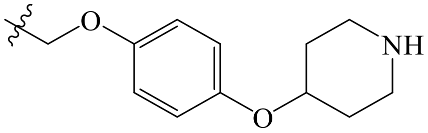 | $-CH_2OCH_2CH_3$ | -H | 0.056 | [24] |
| **248** | 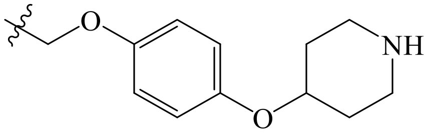 | 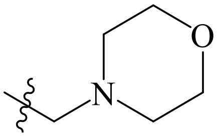 | -H | 0.065 | [24] |

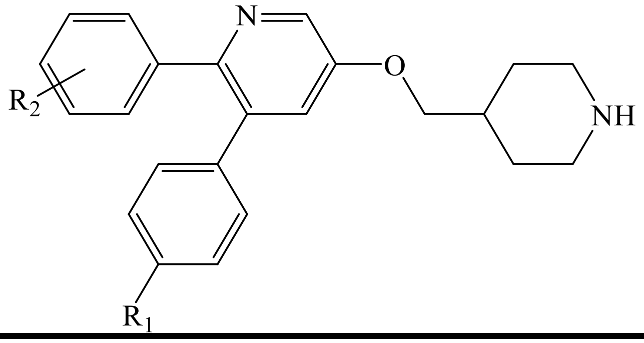

| Cpd | R1 | R2 | Ki (µM) | Reference |
|---|---|---|---|---|
| **249** | -CN | -H | 2.30 | [25] |
| **250** | -H | -H | 50.00 | [25] |
| **251** | -CN | 4-CN | 4.00 | [25] |
| **252** | -CN | 4-F | 0.22 | [25] |
| **253** | -CN | $4-CF_3$ | 0.058 | [25] |
| **254** | -CN | $4-CH_3$ | 0.029 | [25] |
| **255** | -CN | $3-CH_3$ | 0.800 | [25] |
| **256** | -CN | $4-CH_2CH_3$ | 0.200 | [25] |
| **257** | -CN | 4-*i*-Pr | 0.088 | [25] |
| **258** | -CN | $4-OCF_3$ | 0.046 | [25] |
| **259** | 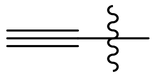 | $4-CH_3$ | 50.000 | [25] |
| **260** | $4-CO_2H$ | $4-CH_3$ | 50.000 | [25] |
| **261** | $-CH_2OH$ | $4-CH_3$ | 50.00 | [25] |
| **262** | 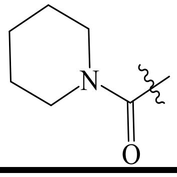 | $4-CH_3$ | 50.000 | [25] |

| Cpd | R1 | R2 | Ki (µM) | Reference |
|---|---|---|---|---|
| **263** | 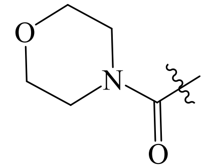 | 4-$CH_3$ | 50.000 | [25] |
| **264** | 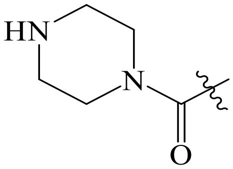 | 4-$CH_3$ | 50.000 | [25] |

| Cpd | Structure | Ki (µM) | Reference |
|---|---|---|---|
| **265** | 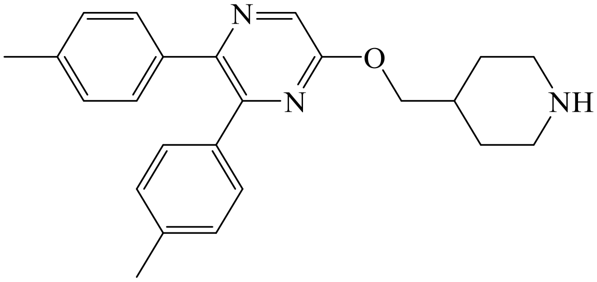 | 47.80 | [25] |
| **266** | 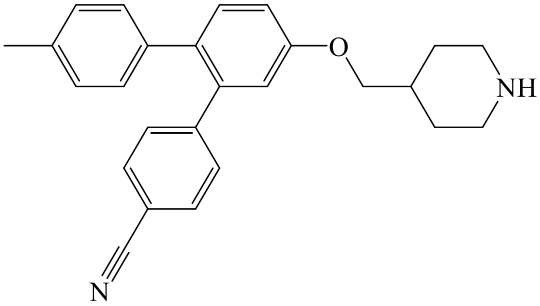 | 4.900 | [25] |
| **267** | 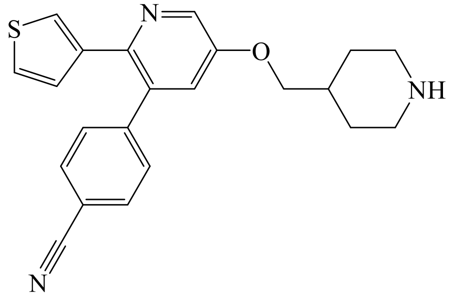 | 2.400 | [25] |
| **268** | 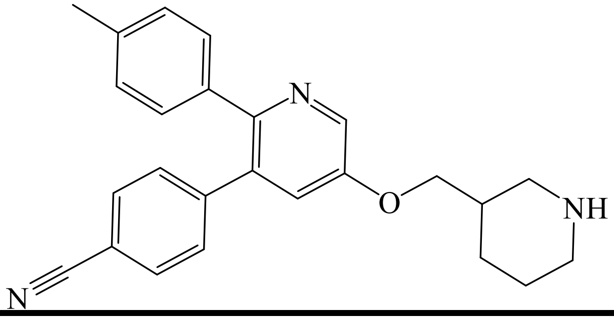 | 0.650 | [25] |

| Cpd | Structure | Ki (μM) | Reference |
|---|---|---|---|
| **269** | 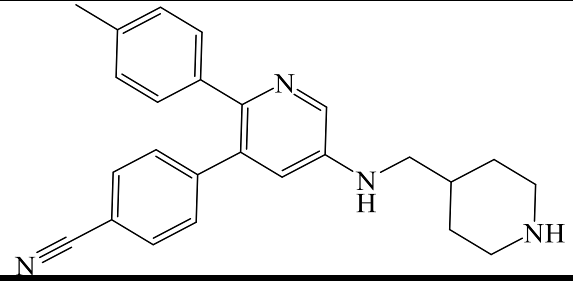 | 1.200 | 25 |

| Cpd | Structure | $IC_{50}$ (μM) | Reference |
|---|---|---|---|
| **270** | 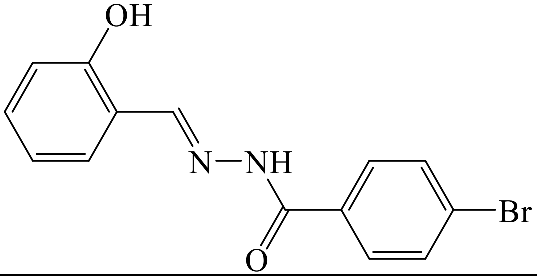 | 0.196 | 23 |

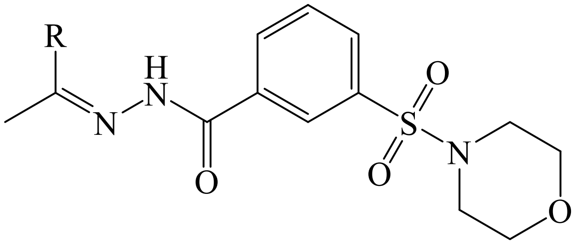

| Cpd | R | $IC_{50}$ (μM) | Reference |
|---|---|---|---|
| **271** | 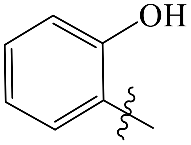 | 0.019 | 23 |
| **272** | 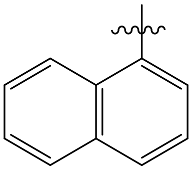 | 10.000 | 23 |
| **273** | 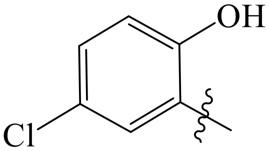 | 0.013 | 23 |
| **274** | 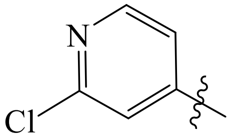 | 3.000 | 23 |
| **275** | 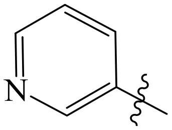 | 3.000 | 23 |
| **276** | 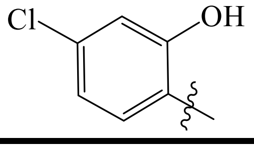 | 0.040 | 30 |

| Cpd | Structure | IC50 (μM) | Reference |
|---|---|---|---|
| **277** | 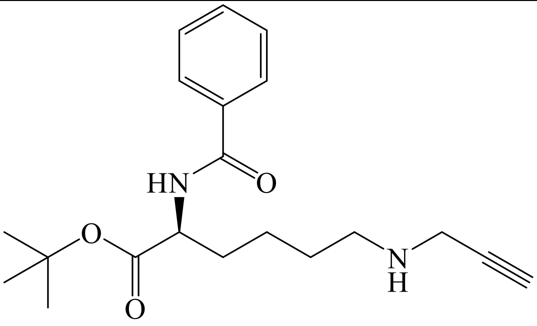 | 143.6 | [22] |

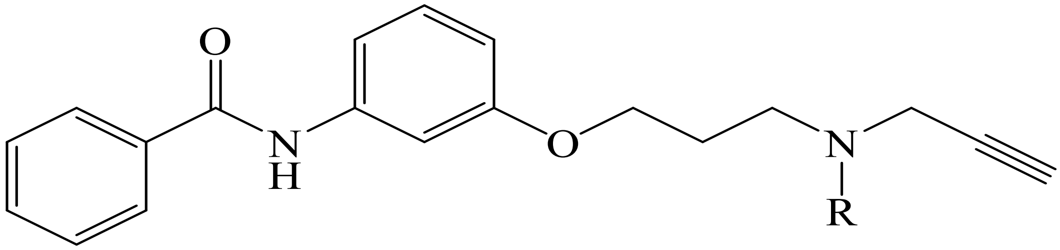

| Cpd | R | IC50 (μM) | Reference |
|---|---|---|---|
| **278** | -H | 184.2 | [22] |
| **279** | $-CH_3$ | 93.1 | [22] |

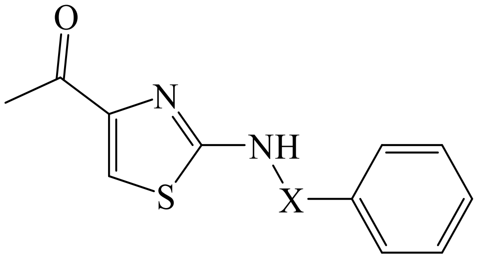

| Cpd | X | IC50 (μM) | Reference |
|---|---|---|---|
| **280** | -CO | 188 | [30] |
| **281** | -CONH | 188 | [30] |

**Table SM2:** PDB codes and crystallographic criteria of LSD-1 proteins utilized in SB modelling together with structures and bioactivities of their co-crystallized ligands.

| No. | Protein | | R | | Co-crystallized ligand | $IC_{50}$ (μM) | Reference |
|---|---|---|---|---|---|---|---|
| | PDB code | Resolution (Å) | Free[a] | Work[b] | | | |
| **282** | **5L3E** | 2.80 | 0.224 | 0.2 | 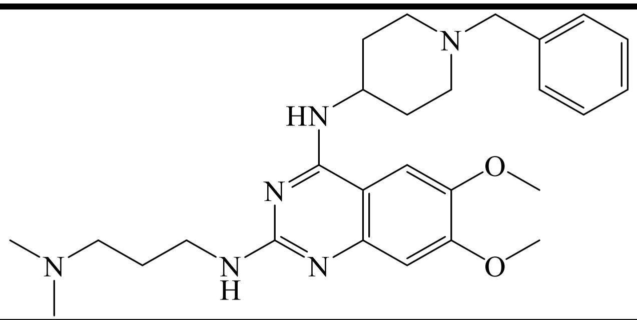 | 0.778 | [43] |
| **283** | **5LBQ** | 3.30 | 0.25 | 0.231 | 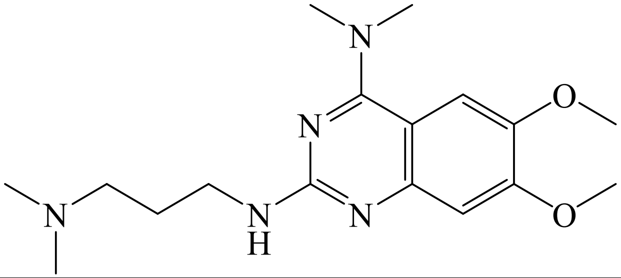 | ND | [43] |
| **284** | **5LGN** | 3.20 | 0.22 | 0.193 | 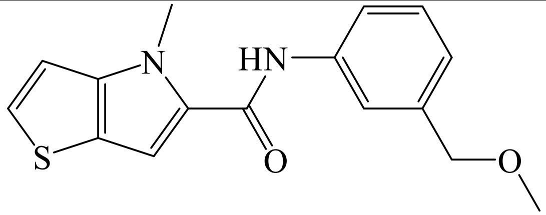 | 2.9 | [36] |
| **285** | **5LHG** | 3.34 | 0.192 | 0.159 | 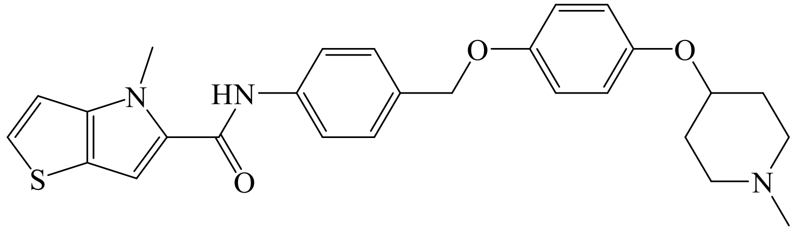 | 2.5 | [24] |

**Table SM2:** PDB codes and crystallographic criteria of LSD-1 proteins utilized in SB modeling together with structures and bioactivities of their co-crystallized ligands.

| No. | Protein | | R | | Co-crystallized ligand | $IC_{50}$ (μM) | Reference |
|---|---|---|---|---|---|---|---|
| | PDB code | Resolution (Å) | Free[a] | Work[b] | | | |
| **235*** | **5LGT** | 3.00 | 0.23 | 0.212 | 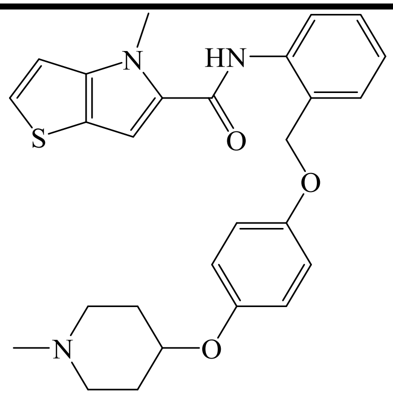 | 0.310 | 24 |
| **286** | **5LGU** | 3.20 | 0.206 | 0.184 | 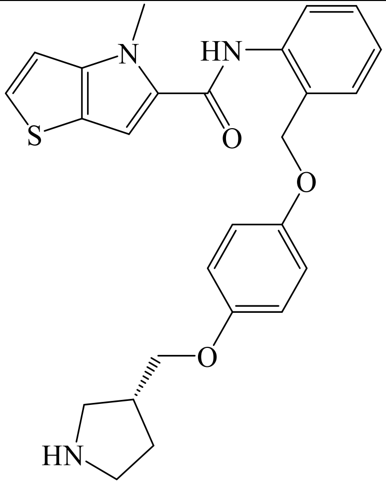 | 0.022 | 24 |

**Table SM2:** PDB codes and crystallographic criteria of LSD-1 proteins utilized in SB modeling together with structures and bioactivities of their co-crystallized ligands.

| No. | Protein | | R | | Co-crystallized ligand | $IC_{50}$ (µM) | Reference |
|---|---|---|---|---|---|---|---|
| | PDB code | Resolution (Å) | Free[a] | Work[b] | | | |
| **244*** | **5LHH** | 3.05 | 0.186 | 0.159 | 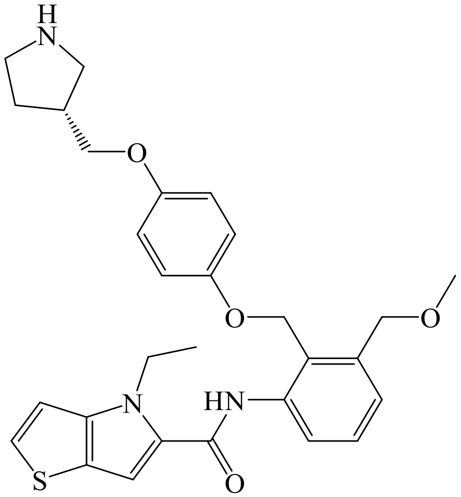 | 0.0084 | 24 |
| **246** | **5LHI** | 3.40 | 0.225 | 0.195 | 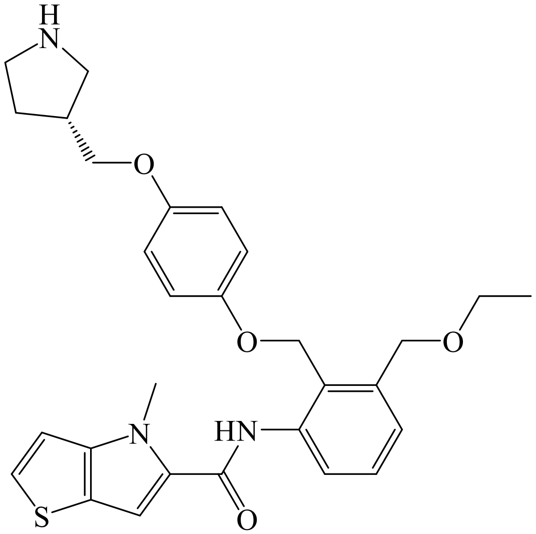 | 0.0078 | 24 |

**Table SM2:** PDB codes and crystallographic criteria of LSD-1 proteins utilized in SB modeling together with structures and bioactivities of their co-crystallized ligands.

| **No.** | **Protein** | | **R** | | **Co-crystallized ligand** | **Ki (µM)** | **Reference** |
|---|---|---|---|---|---|---|---|
| | **PDB code** | **Resolution (Å)** | **Free[a]** | **Work[b]** | | | |
| **254*** | **5YJB** | 2.96 | 0.219 | 0.189 | 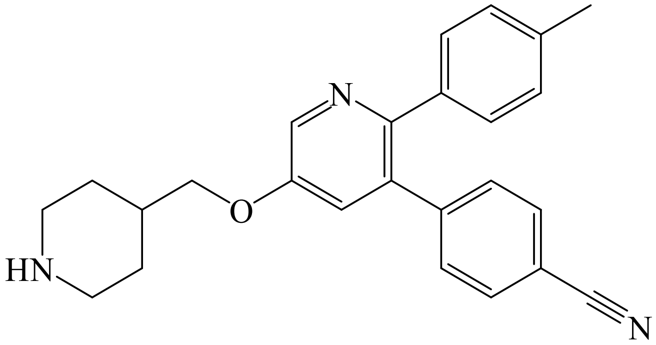 | 0.029 | 44 |

[a]R-Free: assesses possible over-modeling of the data.
[b]R-Work: measures of the agreement between the crystallographic model and the experimental X-ray diffraction data
*Compound with asterisk as mentioned in table SM1

## Section SM1. Docking-Based Pharmacophore Modeling: Preparation of the docking ligand-protein complexes.

Molecularly Diverse potent LSD-1 inhibitors (**199**, **206**, **208**, **224**, **230**, **248**, **256**, **270** and **276** Ki or IC50 ≤ 200 nM, Table SM1) were selected to represent potent LSD-1 ligands. Figure SM1 shows the distribution of the selected compounds within the most potent LSD-1 inhibitors ($IC_{50}$ or Ki ≤ 1 μM in Table SM1) based on LogP, molecular weight, sum of hydrogen bond donors and acceptors. Clearly, the selected compounds are well distributed within the potent LSD-1 ligands, therefore, should reflect significant differences in how they dock into LSD-1, and therefore, provide better coverage of possible binding modes assumed within LSD-1 binding pocket.

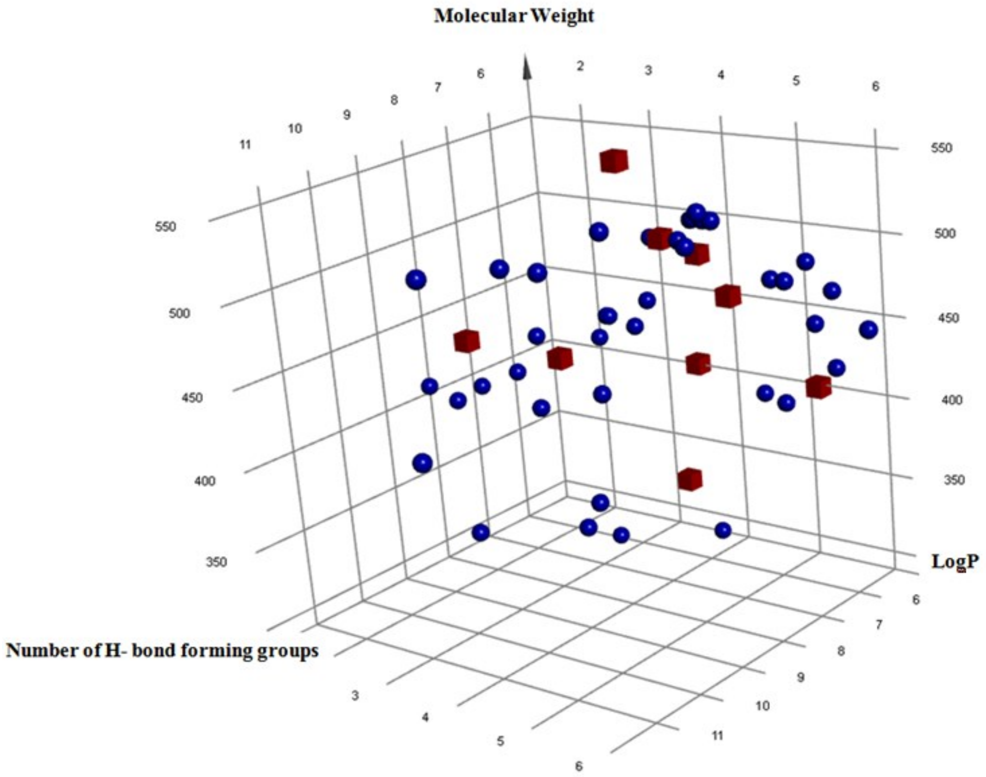


**Figure SM1:** 3D-plot showing the molecular diversity of the docked ligands. Red cubes (■) represent selected potent ligands, while blue spheres (●) represent potent compounds in training list (Activity ≤ 1 μM).

**Section SM2. Docking-Based Pharmacophore Modelling: Ligand Docking.**

Three docking engines were utilized: LibDock [45], LigandFit [46] and CDOCKER [47]. The binding pocket was defined as the cavity volume occupied by co-crystallized ligand within LSD-1 (PDB: 5LHI) [51,52].

- **LibDock**

The site-feature docking algorithm (LibDock) [45] docks ligands, after removing their hydrogen atoms, into an existing active site as guided by binding hotspots. The ligands' conformations are aligned to polar and apolar receptor interactions sites, i.e., hotspots [45,53]. Docking with LibDock have a number of steps [45,53]: (i) Remove hydrogen atoms, (ii) Rank ligand conformations and prune by solvent accessible surface area (SASA), (iii) find hotspots using a grid that is placed into the binding site using polar and apolar probes. (iv) Align ligand poses to binding site hotspots using triplets (i.e., three ligand atoms are aligned to three receptor hotspots). (v) Poses exhibiting clashes with binding site are removed. (vi) A final rigid body optimization is performed using a simple pair-wise score. Top scoring ligand poses are retained. (vii) Hydrogen atoms are added back to the docked ligands. (viii) CHARMm minimization can be carried out to minimize steric clashes caused by newly added hydrogen atoms [45,53].

The following LibDock parameters were applied in the current study: CAT-CONFIRM module of Discovery Studio was used to generate a maximum of 255 conformers (not exceeding an energy threshold of 20kcal/mol from the most stable conformer) for each ligand employing "BEST" conformation generation option. A binding site sphere of 8.15Å radius surrounding the center of the co-crystallized ligand (**246** table SM2, PDB code: 5LHI) [54] was used to define the binding site. The number of binding site hotspots (polar and apolar) was set to 100. The ligand-to-hotspots matching RMSD tolerance value was set to 0.25 Å. The maximum number of poses saved for each ligand during hotspots matching before final pose minimization = 100. Maximum number of poses to be

saved for each ligand in the binding pocket = 100. Minimum LibDock score (poses below this score are not reported) = 100. Maximum number of rigid body minimization steps during the final pose optimization (using BFGS method) = 50. Maximum number of steric clashes allowed before the pose-hotspot alignment is terminated (specified as a fraction of the heavy atom count) = 0.1. Maximum value for nonpolar solvent accessible surface area for a particular pose to be reported as successful = 15.0 Å$^2$. Maximum value for polar solvent accessible solvent area for a particular pose to be reported as successful = 5.0 Å$^2$. No final ligand minimization was implemented (i.e., in the binding pocket).

- **LigandFit**

LigandFit uses Monte-Carlo techniques to generate ligand conformations and docks them into the active site using a shape-based initial docking. The docked poses may optionally be minimized with CHARMm and evaluated with a set of scoring functions. LigandFit docking deals with the receptor as rigid and the ligand as flexible. In LigandFit, docking is composed of following sub-steps [46]: (i) Conformational exploration of flexible ligands employing Monte Carlo search. (ii) Pose and conformation selection based on shape similarity with the binding site. (iii) Candidate conformers and poses exhibiting low shape dissimilarity are further enrolled in calculation of dock energies. (iv) each docked conformation/pose is further fitted into the binding pocket through rigid-body minimization iterations. (v) Docked conformers and poses of docking energies below certain threshold (user-defined) are clustered according to their root mean square (RMS) similarities. Representative conformers/poses are further energy-minimized within the binding site and saved for subsequent scoring [46].

The following LigandFit settings were implemented in the current research. A binding site composed of 6783 grid points (grid spacing of 0.5Å in the X, Y and Z dimensions) surrounding the co-

crystallized ligand within LSD-1 (PDB code: 5LHI) [54] was used. Monte Carlo search parameters: number of trials = 30000 and search step for torsions with polar hydrogens = 30.0. The RMS threshold for ligand-to-binding site shape match was set to 3.0 Å. Interaction energy parameters: The interaction energies were assessed employing CFF force field (version 1.02) with a non-bonded cutoff distance of 10.0 Å and distance dependent dielectric. An energy grid extending 3.0 Å from the binding site was implemented. The interaction energy was estimated by a trilinear interpolation value using soft potential energy approximations. Rigid body ligand minimization parameters: 40 steepest descend followed by 80 BFGS minimization iterations were applied to every orientation of the docked ligand.

- **CDOCKER**

CDOCKER is based on simulated annealing-molecular dynamics for docking [47] CDOCKER protocol includes the following steps. (i) A set of ligand conformations are generated using high-temperature molecular dynamics starting with different starting conformers. (ii) Random orientations of the conformations are produced by translating the center of the ligand to a specified location within the receptor active site. (iii) A softened energy is calculated, and the orientation is kept if the energy is less than a specified threshold. This process continues until the desired number of low-energy orientations is found, or a maximum number of bad orientations have been tested. (iv) Each orientation is subjected to simulated annealing molecular dynamics by heated up to a high preset temperature then cooled to the target temperature. (v) A final minimization of the ligand in the rigid receptor using non-softened potential is performed. For each final pose, the energy (interaction energy plus ligand strain calculated by CHARMm force field) and the interaction energy alone are calculated. The poses are sorted by CHARMm energy, and the top scoring (most negative, thus favourable to binding) poses are retained. To enhance performance and shorten calculation times, non-bond energy

grid is used for interaction energy calculations instead of full potential energy terms [47] The following CDOCKER parameters were implemented in the current project: A binding site sphere of 8.15 Å radius surrounding the center of the co-crystallized ligand (PDB code: 5LHI) [54] was implemented. Starting ligands' conformers were energy-minimized then heated to 1000 K over 1000 molecular dynamics steps to generate 10 starting random conformations for each ligand. Each random conformer was rotated 10 times within the binding pocket for subsequent energy refinement. The van der Waals energies of the resulting conformers/poses were evaluated and those of ≥ 300 kcal/mol were discarded. Surviving conformers/poses were exposed to a cycle of simulated annealing over 2000 heating steps to targeted temperature of 700 K followed by 5000 cooling steps to targeted temperature of 300 K. The docked poses were energy minimized to a final minimization gradient tolerance zero Kcal/mol/Å. Top 10 poses were saved for subsequent scoring.

**Section SM3. Docking-Based Pharmacophore Modelling: Scoring of the Docked Poses.**

Highest ranking docked poses generated by LibDock, LigandFit, and CDOCKER were scored employing 7 scoring functions: Jain [48], LigScore1, LigScore2 [46], PLP1, PLP2 [49], PMF and PMF04 [50]. LigScore1 and LigScore2 scores were calculated employing CFF force field (version 1.02) and using grid-based energies with a grid extension of 7.5 Å across the binding site. PMF scores were calculated employing cutoff distances of 12.0 Å for carbon-carbon interactions and other atomic interactions, while PMF04 scores were calculated employing cutoff values of 6.0 and 9.0 Å for carbon-carbon interactions and other atomic interactions, respectively.

**Section SM4. Docking-Based Pharmacophore Modelling: Pharmacophore Generation from Docked Poses.**

Each ionizable compound yielded 42 docked poses (2 ionization states x 3 docking engines x 7 scoring functions), non-ionizable compound yielded 21 poses (3 docking engines x 7 scoring functions). The resultant 336 virtual LSD-1 ([7 ionizable compounds x 42 docked poses] + [2 non-ionizable compounds x 21 docked poses] = 336) complexes were imported into Discovery Studio 4.5. Hydrogen atoms were added to the proteins using the Discovery Studio 4.5 protein template for hydrogen atoms. The Receptor-Ligand Pharmacophore Generation Protocol of Discovery Studio 4.5 was then used to extract a maximum of 10 pharmacophore models from each docked pose. A total of 902 pharmacophores were generated and fitted against inhibitors (**1-198**, Table SM1) end up with 669 pharmacophores that succeeded in mapping more than 5 compounds (less successful pharmacophores were discarded). Pharmacophores were clustered into 85 groups using the average hierarchal linkage clustering algorithm available in Discovery Studio (each cluster include approximately 8 pharmacophores). A total of 85 pharmacophores that best explain training compounds' bioactivities, i.e., of best correlations (F-statistic) connecting fit-values (calculated by equation 4) with training list bioactivities (-Log ($IC_{50}$) for compounds **1-198** in Table SM1), were selected to represent their clusters. However, pharmacophores with less than 4 features were discarded, leaving 29 models employed subsequently in ML. Table SM3 shows information about representative pharmacophores including their features, success criteria.

**Table SM3:** The performance of the best representatives (cluster centers) of clustered pharmacophore hypotheses generated for LSD-1 using Docking-based methodology.

| Docked Cpds[a] | Ionization state | Docking Engine | Scoring function | Hypotheses | Pharmacophoric Features in Generated Hypotheses | F[a] |
|---|---|---|---|---|---|---|
| **199** | Ionized | LibDock | PLP1 | 3 | HBA, POSIon, RingArom, 2xHbic | 0.01 |
| | Ionized | LibDock | LigScore1 | 1 | HBA, POSIon, RingArom, 2xHbic | 0.06 |
| **206** | Un-ionized | LibDock | LigScore2 | 3 | HBA, POSIon, RingArom, 2xHbic | 0.23 |
| | Un-ionized | LibDock | LigScore1 | 5 | HBA, POSIon, RingArom, 2xHbic | 0.004 |
| | Un-ionized | LibDock | Jain | 3 | HBA, POSIon, RingArom, Hbic | 4.87 |
| | Un-ionized | Ligandfit | PMF04 | 5 | 2xHBA, POSIon, Hbic | 0.2 |
| | Ionized | CDocker | PLP1 | 4 | 2xHBA, POSIon, Hbic | 0.12 |
| | Ionized | LibDock | PLP1 | 3 | HBA, POSIon, RingArom, 2xHbic | 1.33 |
| | Ionized | LibDock | PMF | 3 | HBA, POSIon, RingArom, Hbic | 0.01 |
| | **Ionized** | **LibDock** | **PMF04** | **8** | **2xHBA, POSIon, Hbic** | **0.02** |
| | Ionized | LibDock | PMF04 | 10 | 2xHBA, POSIon, RingArom | 0.12 |
| **208** | Un-ionized | LibDock | PMF | 5 | HBA, POSIon, RingArom, 2xHbic | 0.01 |
| | Ionized | LibDock | PLP2 | 3 | HBA, POSIon, RingArom, 3xHbic | 0.004 |
| | Ionized | CDocker | PMF04 | 9 | HBA, POSIon, RingArom, 2xHbic | 0.01 |
| | Ionized | LibDock | PMF | 9 | HBA, POSIon, RingArom, 3xHbic | 34.61 |
| **224** | Un-ionized | LibDock | Jain | 5 | 2xHBA, POSIon, Hbic | 0.24 |
| **256** | Un-ionized | CDocker | LigScore1 | 10 | HBA, POSIon, RingArom, Hbic | 0.14 |

**Table SM3:** The performance of the best representatives (cluster centers) of clustered pharmacophore hypotheses generated for LSD-1 using Docking-based methodology.

| Docked Cpds[a] | Ionization state | Docking Engine | Scoring function | Hypotheses | Pharmacophoric Features in Generated Hypotheses | F[b] |
|---|---|---|---|---|---|---|
| **276** | Un-ionized | LibDock | Jain | 6 | 4xHBA, RingArom, Hbic | 1.90 |
| | Un-ionized | Ligandfit | LigScore1 | 2 | 2xHBD, HBA, Hbic | 0.24 |
| | Un-ionized | LibDock | PMF | 5 | 3xHBA, RingArom, 2xHbic | 17.31 |
| **230** | Un-ionized | LibDock | Jain | 9 | HBD, HBA, RingArom, 2xHbic | 17.58 |
| | Un-ionized | LibDock | PMF | 6 | HBA, POSIon, RingArom, Hbic | 5.60 |
| | Un-ionized | Ligandfit | PLP2 | 1 | HBA, POSIon, RingArom, 2xHbic | 0.02 |
| | Un-ionized | Ligandfit | PMF | 1 | 2xHBD, HBA, Hbic | 3.43 |
| | Ionized | Ligandfit | Jain | 3 | 2xHBA, POSIon, 2xHbic | 0.58 |
| | Ionized | Ligandfit | PLP1 | 5 | 2xHBA, POSIon, 3xHbic | 0.16 |
| **248** | Un-ionized | LibDock | PLP1 | 1 | 2xHBA, POSIon, RingArom | 27.08 |
| | Ionized | CDocker | LigScore1 | 6 | HBA, POSIon, RingArom, Hbic | 16.82 |
| | Ionized | LibDock | PMF04 | 10 | HBD, HBA, RingArom, 2xHbic | 0.25 |

[a]Compound as in table SM1. [b]Fisher statistic calculated based on the linear regression between the fit values of collected inhibitors (**1-198**, Table SM1) against pharmacophore hypothesis (employing the "best fit" option and equation (4), supporting section SM7) and their respective LSD-1 inhibitors bioactivities.
[c]Bolded underlined row correspond to pharmacophore model that emerged in the best ML models.

**Section SM5. Supervised ligand-based pharmacophore modeling**: **Selection of Training Subsets**

The collected list of compounds (**1-198**, Table SM1) were exported from ChEMBL or sketched from recent published articles [18,19,27–29,32] and transformed into single conformer 3D-structures in Discovery Studio 4.5, then saved in structure database (SD) format. The conformational space of each collected compound (Table SM1) was explored by DiscoveryStudio 4.5 using the "BEST conformer generation" option to generate reasonable multi conformers for each ligand. Conformation generation is based on the generalized CHARMm force field implemented in Discovery Studio 4.5 [39,61–63]. Default parameters were used in the conformation generation procedure, i.e., a conformational group was generated with an energy threshold of 20 kcal/ mol from the local minimized structure which has the lowest energy level and a maximum limit of 255 conformers per molecule [39,61–63]. These compounds were then categorized into 8 diverse training subsets of bioactivity ranges extending over 1.8-2.6 logarithmic cycles (subsets A, B, C, D, E, F, G, and H in Table SM4). Each set's member compounds were selected in such a way that each compound in each structural cluster was sampled at least once [39,63,64]. Training compounds were carefully selected to conform to certain envisaged hypothetical binding mode, and that their inter-member LSD-1 inhibitory differences are attributable to pharmacophoric features (e.g., hydrogen bond acceptor (HBA), hydrogen bond donor (HBD), hydrophobic (Hbic), positive ionizable (PosIon), hydrophobic ring aromatic (HbicArom) or ring aromatic (RingArom)) and not steric and/or electronic effects (electron-donating or -withdrawing groups) [28,61,65–67,70].

**Section SM6. Supervised ligand-based pharmacophore modeling: Exploration of Pharmacophoric Space.**

Molecules of each training subset (and associated conformational models) were placed into spreadsheets together with their bioactivities ($IC_{50}$ values). The bioactivities were reported with "Uncertainty" of 3, i.e., the real bioactivity of any inhibitor is assumed to be situated in an interval ranging from ⅓ to 3 times the reported value of that inhibitor [64,69,70]. HYPOGEN module of Discovery Studio 4.5 was used for automatic pharmacophore generation.

The training subsets were used to explore the pharmacophoric space of LSD-1 inhibitors over 64 automatic HYPOGEN modeling runs that included probing two inter feature distances (100 and 300 picometers), 4 or 5 pharmacophoric features and presence or absence of exclusion spheres, as in Tables SM4 and SM5.

Pharmacophore modeling using HYPOGEN proceeds through three successive phases: (i) constructive phase, (ii) subtractive phase, and (iii) optimization phase. During the constructive phase, HYPOGEN produces common conformational alignments among potent training compounds in a training subset.

A particular inhibitor is defined as being within this category if it satisfies equation (1) [39,71].

$$(MAct \times UncMAct) - (Act/ UncAct) > 0.0 \ldots\ldots\ldots\ldots (1)$$

Where "MAct" is the activity of the most active compound, "Unc" is the uncertainty of the compounds, and "Act" is the activity of the training compounds under question.

In the subsequent subtractive phase, HYPOGEN removes some pharmacophores that fit inactive inhibitors as defined based on equation (2) [39,71].

$$Log (Act) - \log (MAct) > BS \ldots\ldots\ldots\ldots (2)$$

Where BS is the bioactivity spread. This value is set 3.5 by default; however, in the current project we modified to be between 1.8 and 2.6 due to limitations in the availability of inactive compounds of more than 3.5 log cycle's difference of the most potent member. Table SM4 shows the bioactivity spread values implemented for each training subset.

Finally, in the optimization phase, HYPOGEN implements fine perturbations to pharmacophores that survived the subtractive phase (vectored feature rotation, adding new feature and/or subtracting an existing feature) to find new models of better correlation with bioactivity.

**Table SM4**: Training subsets employed in exploring the pharmacophoric space of LSD-1 inhibitors using HYPOGEN and corresponding explored pharmacophoric features.

| Subsets | No. of compounds | Compounds[b] | | | Bioactivity Spread[d] | Explored Features[e] |
|---|---|---|---|---|---|---|
| | | Most active[a] | Moderate active | Least active[c] | | |
| **A** | 18 | **26** | **1, 3, 7, 8, 9, 12, 16, 17, 19, 20, 21, 28, 29** | **108, 117, 118,151** | 1.8 | HBA (0-4), HBD (1-2), Hbic (0-1), POSIon (0-1), HbicArom (0-3) |
| **B** | 19 | **41, 42, 43, 49, 50, 51** | **40, 45, 47, 52, 53, 54** | **79, 83, 97, 108, 117, 118,151** | 2.6 | HBA (1-1), HBD (1-2), HbicArom (3-3) |
| **C** | 12 | **49, 50, 51** | **52, 53, 54** | **79, 83, 97, 108, 117, 118** | 2.6 | HBA (0-2), HBD (1-3), HbicArom (1-3) |
| **D** | 12 | **41, 42, 43** | **47, 54** | **79, 83, 97, 108, 117, 118, 151** | 2.2 | HBA (1-2), HBD (1-3), HbicArom (1-3) |
| **E** | 13 | **57, 58, 61, 63, 70** | **74, 77, 78, 82** | **97, 98, 117, 118** | 2 | HBA (2-2), HBD (1-1), Hbic (0-2), HbicArom (1-1) |
| **F** | 18 | **105, 111, 125** | **104, 127, 135, 170, 171, 173, 180, 190, 194, 195, 198** | **79, 83, 123, 162** | 2.5 | HBA (1-2), HBD (1-1), Hbic (0-2), HbicArom (0-1) |
| **G** | 13 | **101, 129** | **171, 173, 180, 190, 194, 195, 198** | **79, 83, 123, 162** | 2.2 | HBA (1-2), Hbic (1-2), Hbic-Arom (1-2) |
| **H** | 18 | **105, 111** | **104, 125, 127, 135, 170, 171, 173, 180, 190, 194, 195, 198** | **79, 83, 123, 162** | 2.5 | HBA (1-2), HBD (1-1), Hbic (0-2), RingArom (0-1) |

[a,c]Potency categories as defined by equations (1) and (2) under supporting section SM6.[b]Structures are shown in table SM1.
[d]Bioactivity spread determines the separation between actives and inactive compounds for the "Subtractive Phase" during HYPOGEN pharmacophore modelling as in equation (2) and related text. [e]HBA: Hydrogen Bond Acceptor, HBD: Hydrogen Bond Donor, RingArom: Ring Aromatic, Hbic: Hydrophobic, HbicArom: Hydrophobic Aromatic POSIon: positive ionizable feature. Numbers in brackets refer to the allowed ranges of corresponding features.

**Table SM5:** HYPOGEN automatic runs and parameters implemented to explore the pharmacophoric space of each training subset in table SM4**.**

| Run | Min-Max Features[a] | Spacing[b] | EV[c] |
|---|---|---|---|
| 1 | 4-5 | 100 | 0 |
| 2 | 4-5 | 300 | 0 |
| 3 | 5-5 | 100 | 0 |
| 4 | 5-5 | 300 | 0 |
| 5 | 4-5 | 100 | 10 |
| 6 | 4-5 | 300 | 10 |
| 7 | 5-5 | 100 | 10 |
| 8 | 5-5 | 300 | 10 |

[a]Max-Min refers to the allowed range of pharmacophoric features in each model.
[b]Spacing refers to the maximum interfeature distance in picometers. Other parameters were set to their default values.
[c]EV: maximum number of allowed exclusion volumes.

**Section SM7. Supervised ligand-based pharmacophore modelling**: **Assessment of the Generated Pharmacophore Hypotheses**

During generation of pharmacophore hypotheses, HYPOGEN attempts to reduce certain cost criteria consisting of three components: Weight cost, error cost, and configuration cost [39,49,72] Weight cost increases when the feature weight (bioactivity contribution of each feature) in a model diverges from 2. The deviation between the experimentally determined bioactivities and estimated values adds to the error cost. The activity of any training compound can be predicted from certain pharmacophore model through equation (3) [39,60,62,63].

$$\text{Log (Estimated Activity)} = I + \text{Fit} \ldots\ldots\ldots\ldots\ldots\ldots\ldots \ (3)$$

Where, I = the intercept of the regression line gained by plotting the log of the biological activity of the training set compounds against the Fit values of the training compounds. The Fit value for any compound is calculated automatically by equation (4).

$$\text{Fit} = \text{mapped hypothesis features} \times W\,[1 - \Sigma(\text{disp/tol})^2] \ldots\ldots\ldots\ (4)$$

Where, mapped hypothesis features represent the number of pharmacophore features that successfully superimpose (i.e., overlap or map with) corresponding chemical moieties within the fitted compound. "W" is the weight of the corresponding hypothesis feature spheres. This value is fixed to 1.0. "disp" is the distance between the center of a particular pharmacophoric sphere (feature centroid), and the center of the corresponding overlapped chemical moiety of the fitted compound. "Tol or Tolerance" is the radius of the pharmacophoric feature sphere. "$\Sigma(\text{disp/tol})^2$" is the summation of $(\text{disp/tol})^2$ values for all pharmacophoric features that successfully map corresponding chemical functionalities in the fitted compound [73,74].

The third cost term, known as "configuration cost" (Config cost), penalize the complexity of the hypothesis. The more features in a generated hypothesis the higher the value of this cost.

The overall cost (total cost) of a hypothesis is obtained by summing over the three cost factors. However, error cost is the main contributor to total cost. Furthermore, HYPOGEN calculates the cost of a null hypothesis that assumes no relationship in the data, i.e., experimental activities are normally distributed about their mean. Accordingly, the greater the difference from the null hypothesis cost, the more likely that the pharmacophore hypothesis represents significant correlation. HYPOGEN ranks the generated pharmacophores according to their total costs [39,60,62,63]. HYPOGEN presents the highest-ranking models (10 by default) as optimal pharmacophores generated from certain automatic modeling run.

Another approach to assess the quality of Discovery Studio-HYPOGEN pharmacophores is to cross-validate them based on Fisher's randomization test (Fisher, 1966) via Cat-Scramble program implemented in HYPOGEN. In this validation HYPOGEN is instructed to generate 9 random spreadsheets by the Cat-Scramble command. Subsequently, HYPOGEN is challenged to use these

random spreadsheets to generate pharmacophore hypotheses using same features and parameters used in generating the initial unscrambled hypotheses. Success in generating pharmacophores of comparable cost criteria to those produced by the original unscrambled data reduces the confidence in the unscrambled original pharmacophore models and the training compounds [39,60,62,63,75]. Based on Fisher randomization criteria, only pharmacophores that scored more than 80% significance were included in subsequent processing (clustering and ML analyses). Therefore, 407 pharmacophore models were selected for subsequent clustering.

**Section SM8. Supervised ligand-based pharmacophore modeling: Clustering of the Generated Pharmacophore Hypotheses**

Surviving models were clustered into 40 groups using the hierarchical average linkage method in HYPOGEN. Subsequently, best representatives based on their F-values (calculated from the correlation of fit values against the bioactivities (-Log ($IC_{50}$)) for the total list of collected LSD-1 inhibitors **1-198** in Table SM1) were selected. Subsequently, pharmacophores of less than 4 diverse features or only fitted less than 5 compounds in Table SM1, were removed leaving 27 surviving pharmacophores to proceed to the subsequent ML modeling. Supplementary table SM6 shows information about representative pharmacophores including their features, and success criteria.

**Table SM6:** The performance of the best representatives (cluster centers) of clustered pharmacophore hypotheses generated for LSD-1 using HYPOGEN pharmacophores generation methodology.

| Training Set[a] | Run[b] | Hypotheses[c] | Pharmacophoric Features in Generated Hypotheses | Total cost | Cost of null hypothesis | Residual Cost[d] | R run[e] | F total[f] |
|---|---|---|---|---|---|---|---|---|
| A | 1 | 2 | HBA, HBD, Hbic, POSIon | 77.15 | 67.70 | 9.45 | 0.95 | 0.48 |
| | 1 | 4 | 2xHBA, HBD, HbicArom | 77.59 | 67.70 | 9.89 | 0.94 | 1.21 |
| | 3 | 7 | HBA, HBD, HbicArom, Hbic, POSIon | 78.14 | 67.70 | 10.44 | 0.89 | 1.28 |
| | 4 | 5 | HBA, HBD, HbicArom, Hbic, POSIon | 77.97 | 67.70 | 10.27 | 0.94 | 0.38 |
| | 4 | 7 | HBA, HBD, HbicArom, Hbic, POSIon | 78.58 | 67.70 | 10.88 | 0.94 | 0.18 |
| | 5 | 10 | 2xHBA, HBD, HbicArom | 77.38 | 67.70 | 9.68 | 0.93 | 1.03 |
| | 6 | 2 | 2xHBA, HBD, Hbic | 75.38 | 67.70 | 7.68 | 0.97 | 3.28 |
| | 6 | 9 | 2xHBA, HBD, Hbic | 76.10 | 67.70 | 8.4 | 0.92 | 8.33 |
| | 7 | 6 | HBA, HBD, HbicArom, Hbic, POSIon, 3xEV | 76.69 | 67.70 | 8.99 | 0.93 | 1.93 |
| C | 1 | 1 | HBA, 2xHBD, HbicArom | 56.76 | 68.69 | 11.93 | 0.99 | 32.08 |
| | 2 | 1 | HBA, 2xHBD, HbicArom | 56.53 | 68.69 | 12.16 | 0.99 | 133.56 |
| | 2 | 7 | HBA, 2xHBD, HbicArom | 57.47 | 68.69 | 11.22 | 0.97 | 35.03 |
| | 5 | 9 | HBA, 2xHBD, HbicArom, 3xEV | 58.06 | 68.69 | 10.63 | 0.97 | 37.76 |
| | 6 | 5 | HBA, 2xHBD, HbicArom | 56.50 | 68.69 | 12.19 | 0.98 | 43.96 |
| | 6 | 6 | 2xHBA, HBD, HbicArom | 56.62 | 68.69 | 12.07 | 0.98 | 34.25 |
| E | 1 | 2 | 2xHBA, HBD, HbicArom | 58.03 | 62.34 | 4.31 | 0.99 | 2.34 |
| | 2 | 9 | 2xHBA, HBD, HbicArom | 57.54 | 62.34 | 4.8 | 0.99 | 10.63 |
| | 6 | 1 | 2xHBA, HBD, HbicArom, 3xEV | 56.67 | 62.34 | 5.67 | 0.99 | 13.50 |
| | 6 | 6 | 2xHBA, HBD, HbicArom, 2xEV | 57.24 | 62.34 | 5.1 | 0.98 | 7.02 |
| | 6 | 8 | 2xHBA, HBD, HbicArom | 57.34 | 62.34 | 5 | 0.98 | 19.12 |

| Training Set[a] | Run[b] | Hypotheses[c] | Pharmacophoric Features in Generated Hypotheses | Total cost | Cost of null hypothesis | Residual Cost[d] | R run[e] | F total[f] |
|---|---|---|---|---|---|---|---|---|
| | 1 | 5 | 2xHBA, HBD, Hbic | 79.9 | 79.20 | 0.7 | 0.95 | 0.20 |
| | 1 | 8 | 2xHBA, HBD, Hbic | 80.09 | 79.20 | 0.89 | 0.96 | 13.04 |
| F | 1 | 9 | 2xHBA, HBD, Hbic | 80.22 | 79.20 | 1.02 | 0.97 | 4.79 |
| | **2** | **2** | **2xHBA, HBD, Hbic** | **78.24** | **79.20** | **0.96** | **0.97** | **5.26** |
| | 5 | 5 | HBA, HBD, 2xHbic, 1xEV | 78.36 | 79.20 | 0.84 | 0.96 | 15.56 |
| H | 1 | 2 | 2xHBA, HBD, Hbic | 79.27 | 79.20 | 0.07 | 0.98 | 0.16 |
| | 1 | 8 | 2xHBA, HBD, Hbic | 79.87 | 79.20 | 0.67 | 0.97 | 6.03 |

[a]Correspond to training subset in Table SM4.
[b]Correspond to runs in Table SM5.
[c]Best models among their cluster members, as judged based on F-statistic generated by correlating fit values of the whole list of collected compounds with the corresponding bioactivities (Table SM1). Numbers correspond to rank of each hypothesis in each DiscoveryStudio automatic pharmacophore generating run.
[d]The difference between the total cost and the cost of the corresponding null hypotheses.
[e]The correlation coefficients between bioactivity estimates and bioactivities of corresponding training compounds in the subset.
[f]Fisher statistic calculated based on the linear regression between the fit values of collected inhibitors (**1-198**, Table SM1) against pharmacophore hypothesis (employing the "best fit" option and equation (4), see supporting section SM7) and their respective LSD1 inhibitors bioactivities.
[g]Bolded underlined row correspond to pharmacophore model that emerged in the best ML models.

**Section SM9. Unsupervised ligand-based pharmacophore modelling: Selection of Training Subsets and Exploration of Pharmacophoric Space**

The pharmacophoric space of LSD-1 inhibitors was explored through 16 carefully selected training subsets from the collected inhibitors (see Supplementary Table SM7). Each subset was used to conduct 12 automatic runs (Table SM8). Needless to say, that training compounds for unsupervised pharmacophore modelling need not to be bioassayed by the same procedure.

The training subsets were selected such that each subset represents certain hypothetical binding mode. Subsequent pharmacophore exploration translated the proposed binding modes into corresponding pharmacophore models. The training compounds of each subset were selected in such a way that the inter-member bioactivity differences within each subset are attributable to the presence or absence of pharmacophoric features (e.g., hydrogen bond acceptor [HBA], hydrogen bond donor [HBD], hydrophobic [Hbic], positive ionizable, or ring aromatic [RingArom]) rather than bioactivity-enhancing or -reducing auxiliary groups (e.g., electron-donating or -withdrawing groups). The conformational space of each inhibitor was explored adopting the "CAESAR" option within Discovery Studio 4.5. Default parameters were used in the conformation generation procedure, i.e., a conformational group was generated with an energy threshold of 20 kcal/ mol from the local minimized structure which has the lowest energy level and a maximum limit of 255 conformers per molecule [39,62,63]. The unsupervised pharmacophore generation package "Common Feature Pharmacophore Generation" evaluates active training set members based on the types of chemical features they contain, along with the ability to adopt certain conformation that allows those features to be superimposed on a particular configuration. The user defines how many molecules must map completely or partially to the hypothesis via the Principal and MaxOmitFeat parameters. Compounds that were considered as "active" were assigned a principal value of 2 and

MaxOmitFeat of 0 to ensure that all of their chemical features will be considered in pharmacophore generation. On the other hand, compounds that were treated as “moderately active” were assigned a principal value of 1 to denote their intermediate status. Inactive compounds were assigned a principal value of 0. Each moderately active or inactive compound was carefully evaluated to assess whether its low potency is attributable to missing one or more pharmacophoric features (i.e., compared to active compounds) or related to possible steric clashes within the binding pocket, or due to both factors. Therefore, inactive (or moderately active) compounds suspected of missing one or more pharmacophoric features were assigned MaxOmitFeat values of 1 or 2, respectively. Spaces occupied by conformers of this group of compounds and free from conformers of active compounds are filled with exclusion volumes. On the other hand, compounds that seem to be inactive mainly due to steric clashes within the binding pocket were assigned MaxOmitFeat value of 0. This value instructs unsupervised LB-modeling to force inactive compound(s) to fit all the pharmacophoric features of the binding model and therefore permits the software to identify spaces occupied by excess structural fragments of inactive compounds and fill them with exclusion volumes. Table SM7 shows training subsets, “Common Feature Pharmacophore Generation” parameters and explored pharmacophoric features employed in unsupervised exploration of the pharmacophoric space of LSD-1 inhibitors. For each training subset and related parameters, unsupervised LB-modelling was instructed to explore 4, 5 or 6-featured pharmacophores, at 1.0 or 3.0 Å interfeature distances and with or without exclusion volumes, as in Table SM8. The resultant pharmacophores are ranked as they are built. The ranking is a measure of how well the active training molecules map onto the proposed pharmacophores, as well as the rarity of the pharmacophore model. If a particular pharmacophore is “rare,” then it will be less likely to map to an inactive compound and therefore it will be given a higher rank [59,64].

**Table SM7:** Training subsets, Unsupervised LB-pharmacophores modeling parameters and explored pharmacophoric features employed in unsupervised exploration of the pharmacophoric space of LSD-1 inhibitors.

| Subsets | No. of Compounds | Principal/MaxOmitFeat Combination | | | | | | Explored Features[b] |
|---|---|---|---|---|---|---|---|---|
| | | 2/0 | 1/ 1 | 1/ 0 | 0/ 2 | 0/1 | 0/0 | |
| **I** | 18 | **26[a]** | **1, 3, 7, 8, 9, 12, 16, 17, 19, 20, 21, 28, 29** | | **108, 117, 118, 151** | | | HBA (0-4), HBD (1-2), Hbic (0-1), POSIon (0-1), RingArom (0-3) |
| **J** | 19 | **41, 42, 43, 49, 50, 51, 52** | **40, 45, 47, 53, 54** | | **79, 83, 97, 108, 117, 118, 151** | | | HBA (1-1), HBD (1-2), RingArom (1-3) |
| **K** | 12 | **49, 50, 51, 52** | **53, 54** | | **79, 83, 97, 108, 117, 118** | | | HBA (0-1), HBD (1-3), RingArom (1-3) |
| **L** | 12 | **41, 42, 43** | **47, 54** | | **79, 83, 97, 108, 117, 118, 151** | | | HBA (1-2), HBD (1-3), RingArom (1-3) |
| **M** | 13 | **57, 58, 61, 63** | **70, 74, 77, 78, 82** | | **97, 98, 117, 118** | | | HBA (1-2), HBD (1-1), Hbic (0-2), RingArom (1-1) |
| **N** | 18 | **105, 111** | **104, 125, 127, 135, 170, 171, 173, 180, 190, 194, 195, 198** | | **79, 83, 123, 162** | | | HBA (1-2), HBD (0-1), Hbic (0-2), RingArom (0-1) |

**Table SM7:** Training subsets, Unsupervised LB-pharmacophores modeling parameters and explored pharmacophoric features employed in unsupervised exploration of the pharmacophoric space of LSD-1 inhibitors.

| Subsets | No. of compounds[a] | Principal/MaxOmitFeat Combination[b] | | | | | | Explored Features[c] |
|---|---|---|---|---|---|---|---|---|
| | | 2/0 | 1/ 1 | 1/ 0 | 0/ 2 | 0/1 | 0/0 | |
| **O** | 13 | **101, 129** | **171, 173, 180, 190, 194, 195, 198** | | **79, 83, 123, 162** | | | HBA (1-2), Hbic (1-2), Hbic-Arom (1-2) |
| **P** | 18 | **105, 111** | **104, 125, 127, 135, 170, 171, 173, 180, 190, 194, 195, 198** | | **79, 83, 123, 162** | | | HBA (1-2), HBD (0-1), Hbic (0-2), RingArom (0-1) |
| **Q** | 9 | **254, 256, 257** | **265, 266** | | **250** | | **262, 263, 264** | HBA (1-3), Hbic (0-1), POSIon (0-1), RingArom (2-3) |
| **R** | 10 | **252, 253, 258** | **249, 255, 267, 268, 269** | | **250, 259** | | | HBA (2-4), Hbic (0-1), POSIon (0-1), RingArom (2-3) |
| **S** | 9 | **207, 208, 209** | **218, 219** | | **223, 225, 226, 279** | | | HBA (1-4), Hbic (1-3), POSIon (0-1), RingArom (1-3) |
| **T** | 11 | **200, 201, 202, 203, 204, 205** | **221, 222, 224** | | **210, 279** | | | HBA (2-4), Hbic (0-1), POSIon (0-1), RingArom (1-3) |

**Table SM7:** Training subsets, Unsupervised LB-pharmacophores modeling parameters and explored pharmacophoric features employed in unsupervised exploration of the pharmacophoric space of LSD-1 inhibitors.

| Subsets | No. of compounds[a] | Principal/MaxOmitFeat Combination[b] | | | | | | Explored Features[c] |
|---|---|---|---|---|---|---|---|---|
| | | 2/0 | 1/ 1 | 1/ 0 | 0/ 2 | 0/1 | 0/0 | |
| **U** | 10 | **271, 273, 276** | **272, 274, 275** | | **277, 278, 280, 281** | | | HBA (2-4), Hbic (0-1), RingArom (0-2) |
| **V** | 13 | **229, 244, 245, 246, 247** | **235, 237, 241** | | **227, 228, 231, 232, 233** | | | HBA (2-4), Hbic (0-2), POSIon (0-1), RingArom (1-3) |
| **W** | 12 | **236, 240** | **238, 243** | **242** | **227, 228, 231, 232, 233, 234** | | **239** | HBA (1-3), Hbic (0-1), POSIon (0-1), RingArom (1-3) |
| **X** | 7 | **212, 216** | **213, 214, 215** | | **211, 215** | | | HBA (0-3), HBD (1-3), Hbic (0-1), RingArom (0-2) |

[a]Compounds' structures are shown in Table SM1. [b]2/0: Active compounds with no missed feature, 1/1: Intermediate compounds with one missed feature, 1/0: Intermediate compounds with no missed feature, 0/2: Inactive compounds with tow missed features, 0/1: Inactive compounds with one missed feature, 0/0: Inactive compounds with no missed features. [c]HBA: Hydrogen Bond Acceptor, HBD: Hydrogen Bond Donor, RingArom: Ring Aromatic, Hbic: Hydrophobic, Hbic-Arom: Hydrophobic Ring Aromatic POSIon: positive ionizable feature. Numbers in brackets refer to the allowed ranges of corresponding features.

**Table SM8:** "Common Feature Pharmacophore Generation" automatic runs performed to explore the pharmacophoric space of each training subset in table SM7.

| Run | Min-Max Features[a] | Spacing[b] | EV[c] |
|---|---|---|---|
| 1 | 4-4 | 1.0 | 0 |
| 2 | 4-4 | 3.0 | 0 |
| 3 | 5-5 | 1.0 | 0 |
| 4 | 5-5 | 3.0 | 0 |
| 5 | 6-6 | 1.0 | 0 |
| 6 | 6-6 | 3.0 | 0 |
| 7 | 4-4 | 1.0 | 10 |
| 8 | 4-4 | 3.0 | 10 |
| 9 | 5-5 | 1.0 | 10 |
| 10 | 5-5 | 3.0 | 10 |
| 11 | 6-6 | 1.0 | 10 |
| 12 | 6-6 | 3.0 | 10 |

[a]Max-Min refers to the allowed range of pharmacophoric features in each model. [b]Spacing refers to the maximum inter-feature distance in Å. Other parameters were set to their default values. [c]EV: maximum number of allowed exclusion volumes.

**Section SM10. Unsupervised ligand-based pharmacophore modeling**: **Clustering of the Generated Pharmacophore Hypotheses**

Eventually, 882 pharmacophore models resulted from automatic runs. These were fitted against LSD-1 inhibitors **1-198** Table SM1, and their fit values (generated by equation 4, supporting section SM7) were correlated with their corresponding bioactivities ($IC_{50}$ values, Table SM1) to determine their correlation F-values. Subsequently, the resulting pharmacophores were clustered using hierarchal average linkage clustering algorithm into 60 groups (about 14 pharmacophores per cluster). Thereafter, pharmacophore models that best explain bioactivity variations within LSD-1 inhibitors **1-198** (Table SM1), based on F-statistic, were selected to represent their clusters yielding 60 pharmacophores. However, models with less than four features were discarded leaving 24 pharmacophores to proceed for subsequent ML. Table SM9 shows the performances of surviving pharmacophores.

**Table SM9:** The performance of the best representatives (cluster centers) of clustered pharmacophore hypotheses generated for LSD-1 using Unsupervised-LB pharmacophore modeling.

| Training Set[a] | Run[b] | Hypotheses | Pharmacophoric Features in Generated Hypotheses | F[c] |
|---|---|---|---|---|
| I | 1 | 3 | HBA, HBD, POSIon, RingArom | 0.37 |
| J | 1 | 9 | HBA, 2xHBD, RingArom | 60.58 |
| K | 3 | 3 | HBA, 2xHBD, 2xRingArom | 25.58 |
| L | 2 | 5 | 2xHBA, HBD, RingArom | 24.91 |
| | 8 | 8 | 2xHBA, HBD, RingArom, 10xEV | 12.25 |
| M | 1 | 4 | HBA, HBD, RingArom, Hbic | 3.32 |
| | 2 | 8 | HBA, HBD, RingArom, Hbic | 8.87 |
| | 3 | 5 | HBA, HBD, RingArom, 2xHbic | 9.37 |
| | 3 | 10 | HBA, HBD, RingArom, 2xHbic | 5.07 |
| | 4 | 4 | 2xHBA, HBD, RingArom, Hbic | 4.96 |
| | 4 | 6 | 2xHBA, HBD, RingArom, Hbic | 5.57 |
| | 6 | 2 | 2xHBA, HBD, RingArom, 2xHbic | 11.10 |
| | 11 | 3 | 2xHBA, HBD, RingArom, 2xHbic | 6.24 |
| P | 7 | 4 | 2xHBA, RingArom, Hbic, 10xEV | 0.44 |
| | 7 | 6 | 2xHBA, RingArom, Hbic, 10xEV | 0.85 |
| | 9 | 6 | 2xHBA, RingArom, Hbic, 10xEV | 0.06 |
| Q | 3 | 5 | HBA, POSIon, 2xRingArom, Hbic | 8.88 |
| | 3 | 7 | HBA, POSIon, 2xRingArom, Hbic | 8.32 |
| | 9 | 9 | HBA, POSIon, 2xRingArom, 10xEV | 3.11 |
| **S** | **1** | **4** | **HBA, POSIon, RingArom, Hbic** | **0.08** |
| | 1 | 10 | HBA, POSIon, RingArom, Hbic | 0.38 |
| | 2 | 9 | HBA, POSIon, RingArom, Hbic | 0.003 |
| W | 4 | 7 | HBA, POSIon, 2xRingArom, Hbic | 0.003 |
| | 6 | 8 | HBA, POSIon, 3xRingArom, Hbic | 0.52 |

[a]Correspond to subsets in Table SM7.
[b]Correspond to runs in Table SM8.
[c]Fisher statistic calculated based on the linear regression between the fit values of collected inhibitors (**1-198**, Table SM1) against pharmacophore hypothesis (employing the "best fit" option and equation (4), supporting section SM7) and their respective LSD-1 inhibitors bioactivities.
[d]Bolded underlined row correspond to pharmacophore model that emerged in the best ML models.

**SM11. Validation of generated Pharmacophore Models using Receiver-operating Characteristic (ROC) Curve Analysis.**

ML selected pharmacophores were validated using receiver-operating characteristic (ROC) curve analysis. ROC analysis evaluates the capability of a particular pharmacophore model(s) to correctly classify a group of compounds into actives and Inactives. It affords the Area Under the ROC curve (ROC-AUC) [39,86,96]. ROC curve is plotted by considering the highest score of an active molecule as the first threshold then counting the number of inactive compounds within this cut-off value, and both the corresponding sensitivity (Se) and specificity (Sp) are calculated using equations (5) and (6), respectively. This process is repeated using the active molecule possessing the second-highest score and so on until the scores of all active compounds are considered as selection cut-off values [86,97].

$$Se = \frac{Number\ of\ Selected\ Actives}{Total\ Number\ of\ Actives} = \frac{TP}{TP + FN} \quad \text{........ (5)}$$

$$Sp = \frac{Number\ of\ Discarded\ Inactives}{Total\ Number\ of\ Inactives} = \frac{TN}{TN + FP} \quad \text{......... (6)}$$

Where, TP (true positive) is the number of active compounds that are captured by the pharmacophore under concern, FN (false negative) is the number of active compounds discarded from the hits list, TN (true negative) is the number of discarded inactives, while FP (false positive) is the number of captured inactives.

The ROC curve for ideal distributions (where active compounds are separate and distinct from the inactives, i.e., no overlap between actives and inactives) arise vertically to the upper-left corner (Se = Sp = 1) and then joins the upper-right corner horizontally. Hence, the more a ROC curve bends towards the upper left corner of the diagram, the more distinct the signal appears [86].Success

of a virtual screening workflow depends on the area under the ROC curve (AUC): with an optimal value of 1 and random distribution of 0.5. Any virtual screening that performs better than random discrimination of actives and inactives get an AUC value between 0.5 and 1, whereas an AUC value lower than 0.5 represents the unfavorable case of a virtual screening method that has a higher probability to assign the best scores to inactives than to actives [39,86].

**Section SM12. *In vitro* experimental studies- Cell culture: Growth conditions.**

SH-SY5Y (Neuroblastoma, CRL-2266), Panc-1 (pancreatic carcinoma, CRL-1469) and U-87 MG (glioblastoma, HTB-14) originally obtained from the American Type Culture Collection (ATCC; USA) and were kindly provided by Stem Cell Therapy Center (CTC), University of Jordan.

Each cell line was cultured in specific media. SH-SY5YH and U-87 MG cells were cultured with Dulbecco's modified Eagle medium: Nutrient Mixture F-12 (DMEM/F-12) (DMEM) (Gibco, USA), While Panc-1 cells were cultured in Advanced Dulbecco's modified Eagle medium (Advanced DMEM) (Gibco, USA), supplemented with 15% (v/v) heat-inactivated fetal bovine serum (FBS) (biowest, USA), 1% v/v L-glutamine (EuroClone, Italy), and 1% (v/v) penicillin-streptomycin (EuroClone, Italy). Cells were grown in 75 $cm^2$ attached types, filter-cap culture flasks (Membrane Solutions, USA). Then were incubated at 37°C in a 90% humidified atmosphere of 5% CO2 (normoxic conditions) and were maintained in a tissue culture incubator (Nuaire, China). The equipment's used in cell culture were commercially pre-sterilized and disposable, and cell culture grade quality solutions were used. All procedures involving the cell culture were performed under class II biological safety cabinet (Heal-Force, China).

**Section SM13. *In vitro* experimental studies- Cell culture: Cell Harvesting (Passaging)**.

When the confluency of the attached cells on a flask surface exceeds 80%, (i.e., observed under an inverted light microscope (Olympus, USA)), trypsin-EDTA (Ethylene diamine tetra acetic acid) was used to trypsinize the cells. The medium was aspirated, cells attached to the flask surface were washed with phosphate buffer saline (PBS) (Hyclon,USA). Thereafter, 2 ml of 1X trypsin-EDTA (Gibco, USA) was added. Afterwards, the flasks were incubated at 37°C for 3-5 minutes (until detachment of cells was observed from outside of the flasks and under the inverted light microscope), and FBS-supplemented growth medium (4 ml) was added to deactivate the trypsin. Detached cells' suspension was centrifuged (Biofuge, Heraeus, Germany) at 1020 RPM, 4 ºC for 5 minutes in 15 ml or 50mL polypropylene tubes (Isolab, Germany). Eventually, cells were re-suspended in fresh media and passed into new culture flasks or separated for further experiments, it has to be mentioned that SH-SY5Y cells were with passage number not exceeding 14, U-87 MG with passage number 6-8, while Panc-1 with passage number equal 32.

**Section SM14. *In vitro* experimental studies- Cell culture: Cell Proliferation Assays.**

- **Viable cell count**

An aliquot of trypsinized cells was used to count viable cells, they were stained with trypan blue (Sigma-Aldrich, UK) in 1: 1 ratio (Trypan blue: Cell suspension). As viable cell membrane is impermeable to trypan blue, the viable cells appear as bright sphere, while dead cells stained in dark blue colour. Stained cell mixture was loaded on Countess II slide, to accurately count the viable cell by applying a deep-learning neural network algorithm implemented in Invitrogen Countess II.

- **MTT Cell Proliferation Assay**

Inhibitory effect of the hits on SH-SY5Y cells were evaluated by a colorimetric method of CellTiter Non-Radioactive Cell Proliferation Assay Kit® (Promega, USA), based on the reduction of a yellow tetrazole, 3-(4,5-Dimethylthiazol-2-yl)-2, 5-diphenyl tetrazolium bromide (MTT), to a purple formazan, a process that take place in the mitochondria of viable cells.

Briefly, the cells were seeded onto 96-well plates (Greiner, Germany) at a concentration of $2\times10^4$ cells/well and incubated for 24 hours through which cells are attached to the walls of the wells. Subsequently, hits under concern were screened on 50 µM and 10 µM concentrations (100 µL/ well). Each concentration was added in triplicates, and every plate contained a control of cells in plain medium. The cells were then incubated at 37 °C for 72 hours. After incubation, the media were aspirated from the wells and replaced by fresh media (100 µL/ well), and 15 µl MTT dye solution. The plates were incubated at 37 °C for four hours, and then 50 µL of solublization/stop solution was added to each well. Optical density (OD) at 560 nm wavelength was recorded one hour later using a 96-well plate reader (GloMax, Promega).

- **Reversal of SH-SY5Y resistance by verapamil**

As SH-SY5Y cells are highly expressing ATP binding cassette subfamily B member 1 (ABCB1, MDR1) (Normalized expression, NX: 36.8%) [100], cell resistance were reversed using 10 µM concentration of verapamil added with each hit and incubated with cells for 72 hours for subsequent MTT viability assay [101,102].

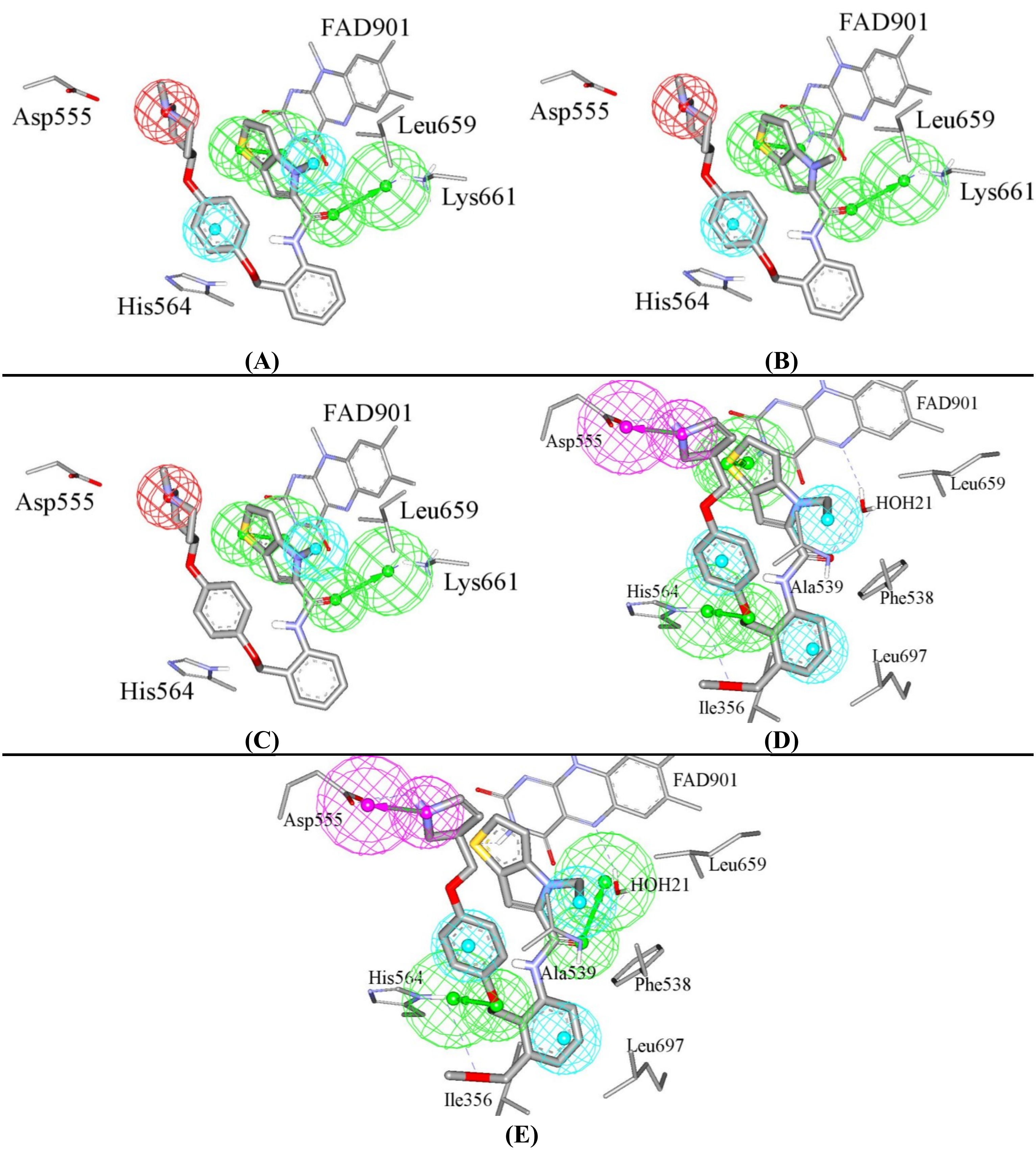


**Figure SM2:** Crystallographic complexes 5LGT and 5LHH with corresponding pharmacophore models fitted onto the co-crystallized ligands. (A, B and C) Three crystallographic SB pharmacophores generated from co-crystallized complexed ligand in 5LGT (compound **235** in Table SM1). (D and E) Two pharmacophore models extracted from the complexed ligand in 5LHH (compound **229** in Table SM1). Hbic features are represented as light blue spheres, HBD as purple vectored spheres, HBA as green vectored spheres, RingArom as orange spheres.

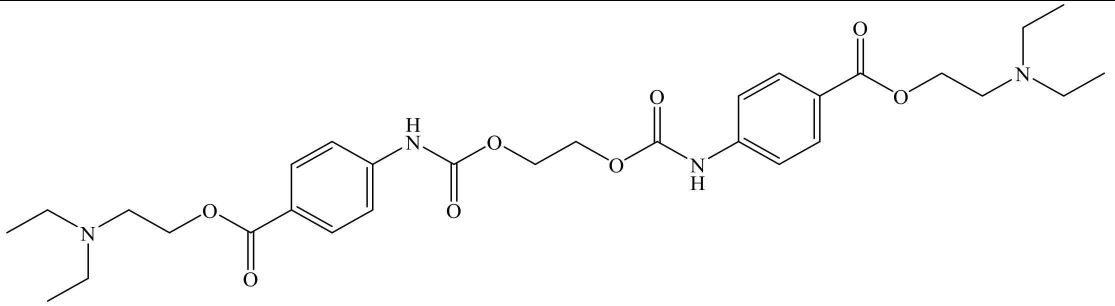

**282** (NCI code: 26158)

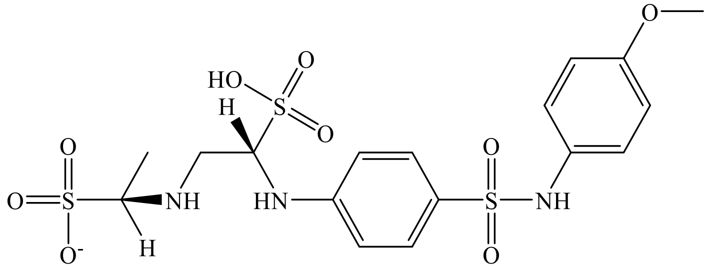

**283** (NCI code: 58439)

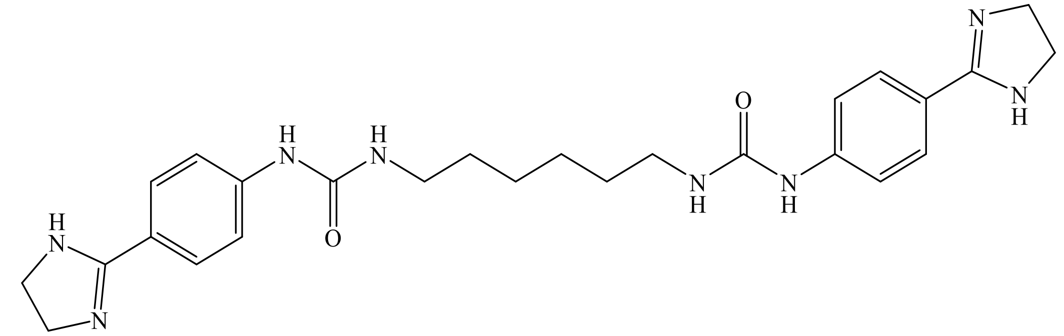

**284** (NCI code: 76533)

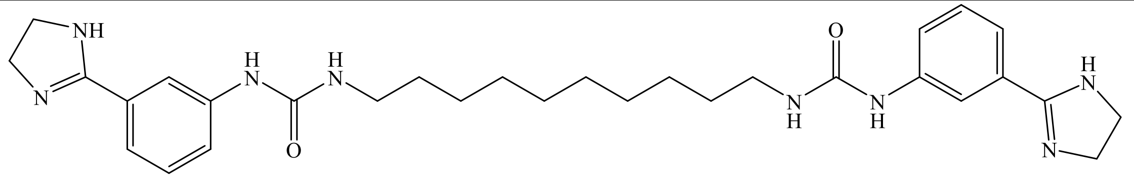

**285** (NCI code: 84456)

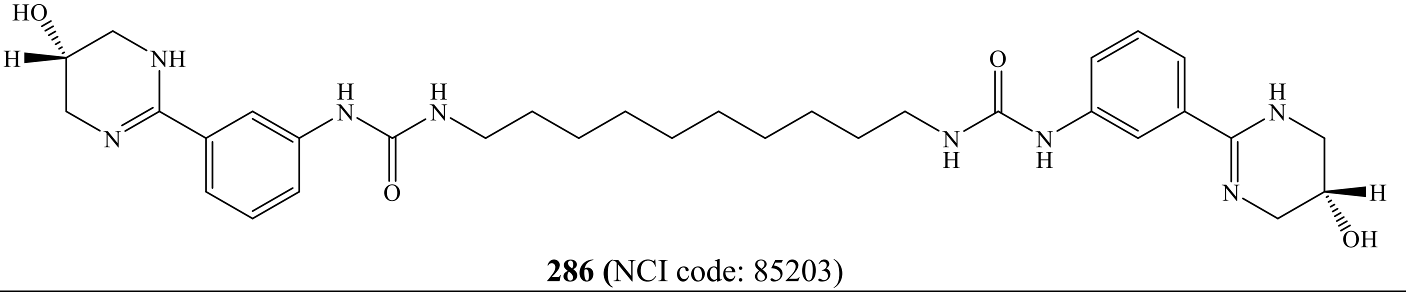

**286** (NCI code: 85203)

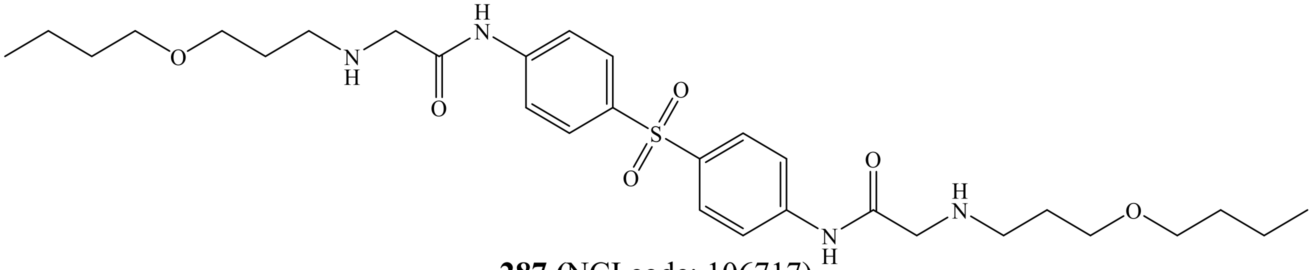

**287** (NCI code: 106717)

**Figure SM3:** Chemical structures of hits ordered from the NCI.

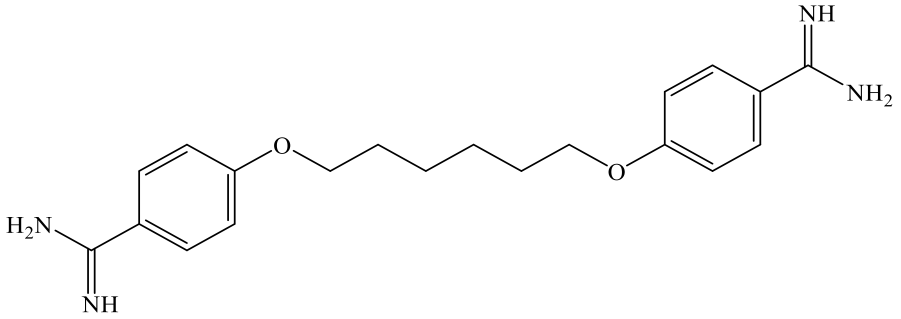

**288 (**NCI code: 115848)

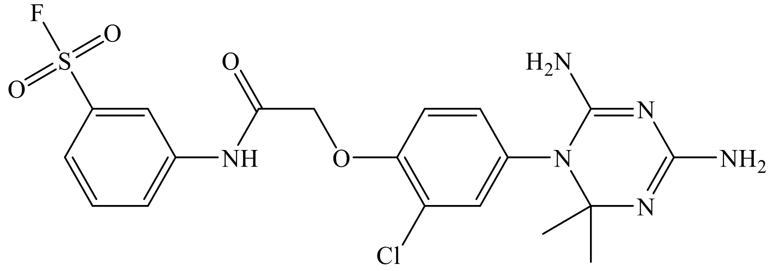

**289 (**NCI code: 120919)

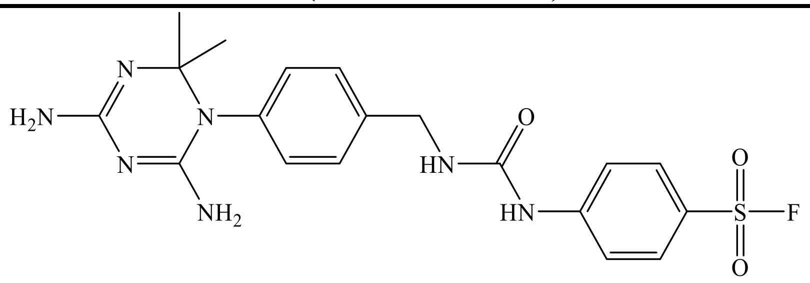

**290 (**NCI code: 123463)

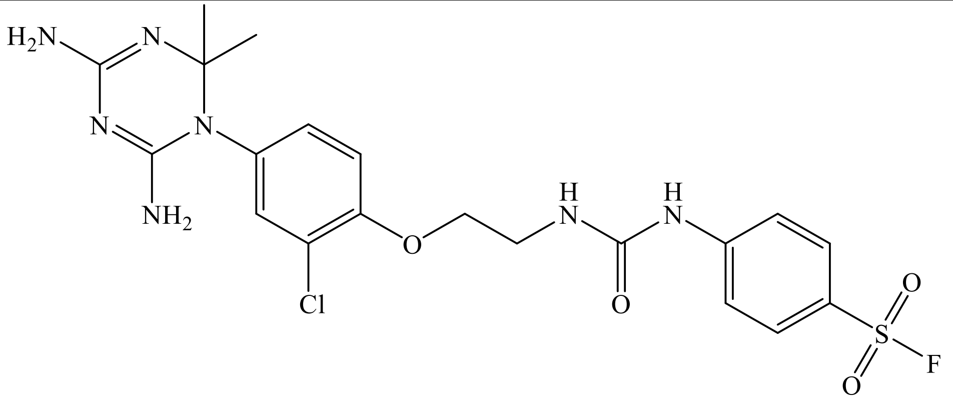

**291 (**NCI code: 123469)

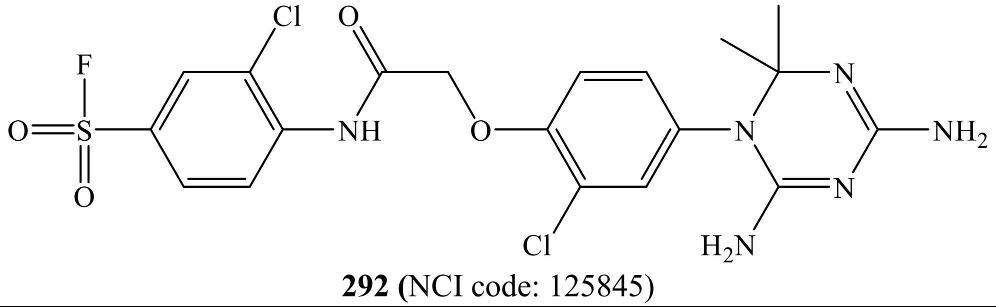

**292 (**NCI code: 125845)

**Figure SM3:** Chemical structures of hits ordered from the NCI.

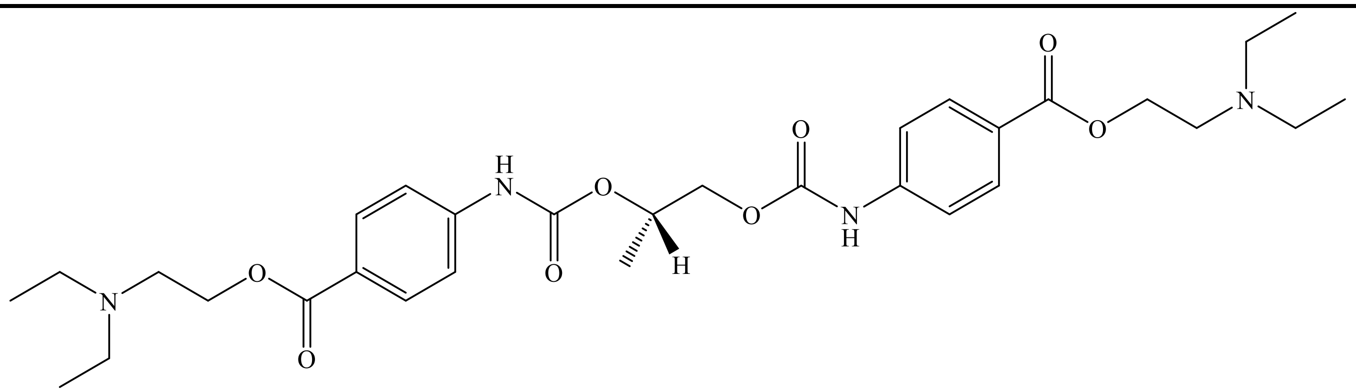


**293 (**NCI code: 26872)

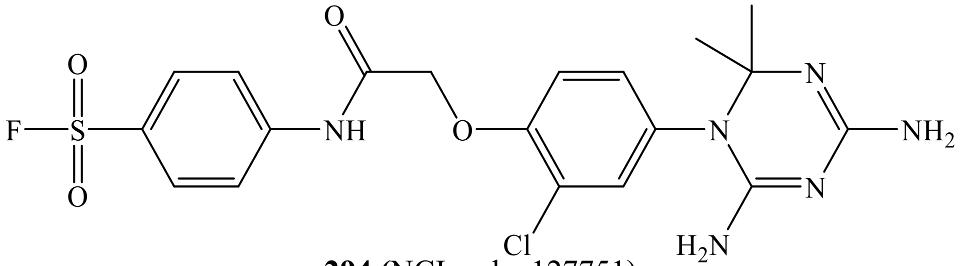


**294 (**NCI code: 127751)

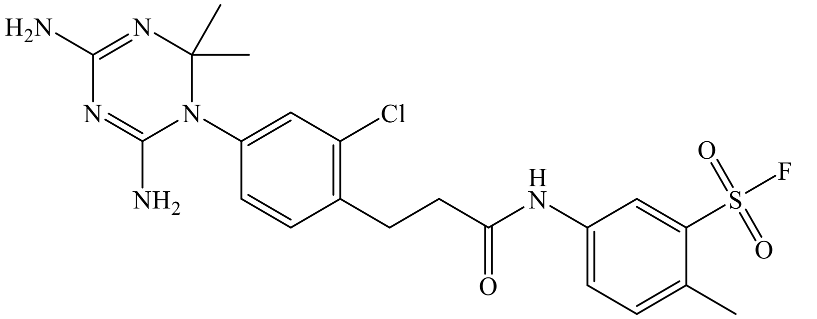


**295 (**NCI code: 133667)

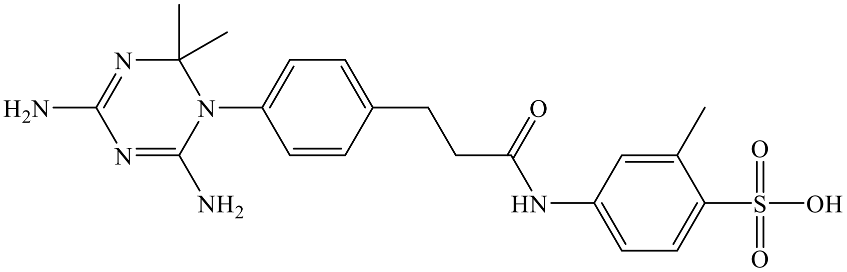


**296 (**NCI code: 135759)

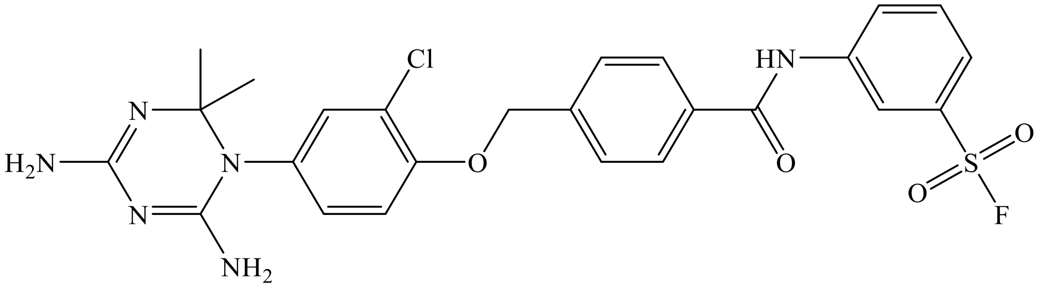


**297 (**NCI code: 135764)

**Figure SM3:** Chemical structures of hits ordered from the NCI.

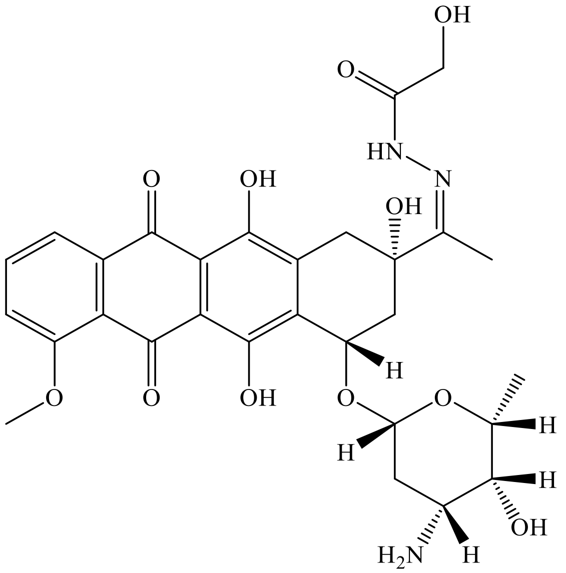


**298 (**NCI code: 146496)

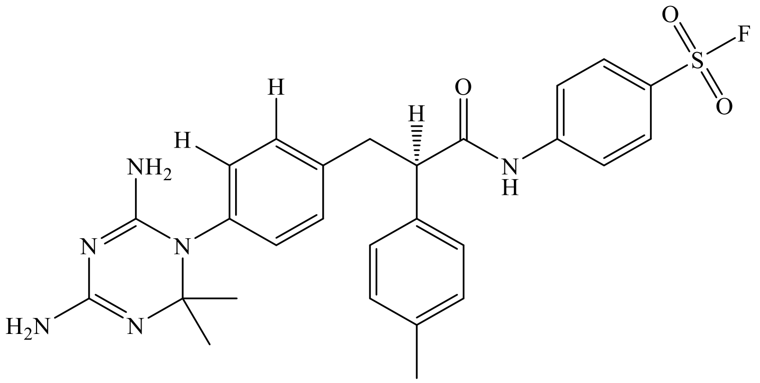


**299 (**NCI code: 211141)

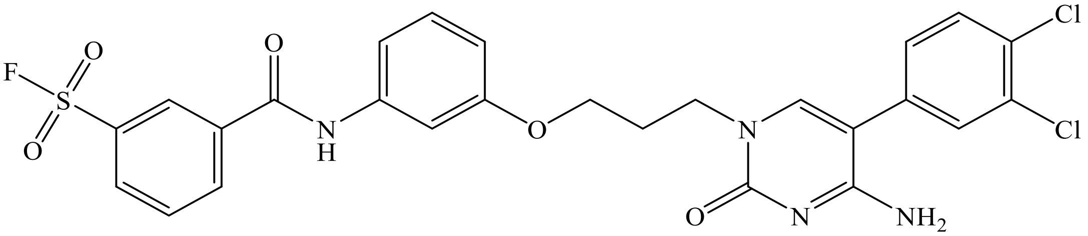


**300 (**NCI code: 211284)

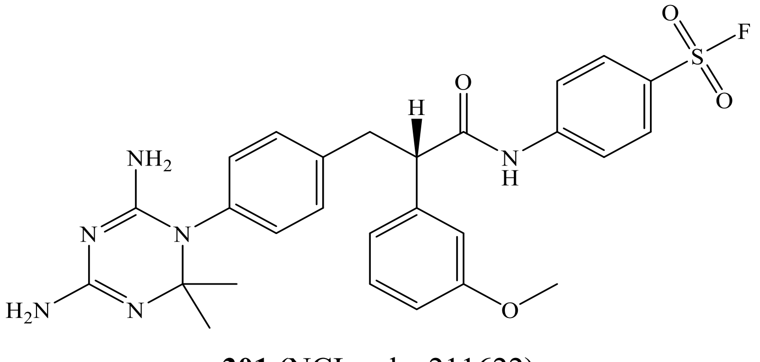


**301 (**NCI code: 211622)

**Figure SM3:** Chemical structures of hits ordered from the NCI.

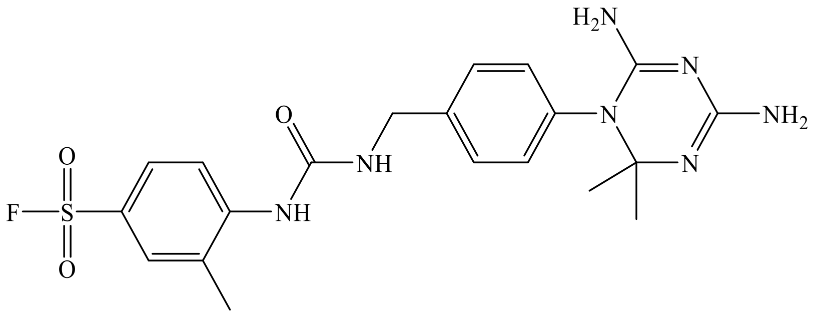

**302** (NCI code: 211623)

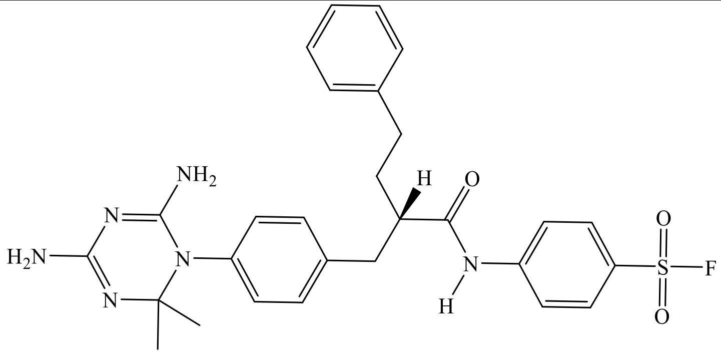

**303** (NCI code: 211632)

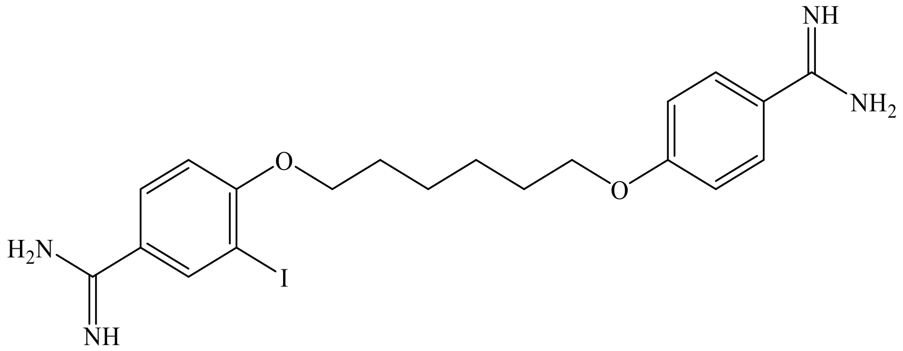

**304** (NCI code: 34574)

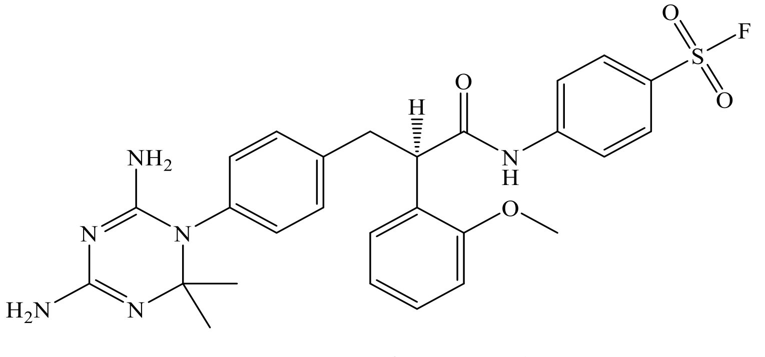

**305** (NCI code: 211641)

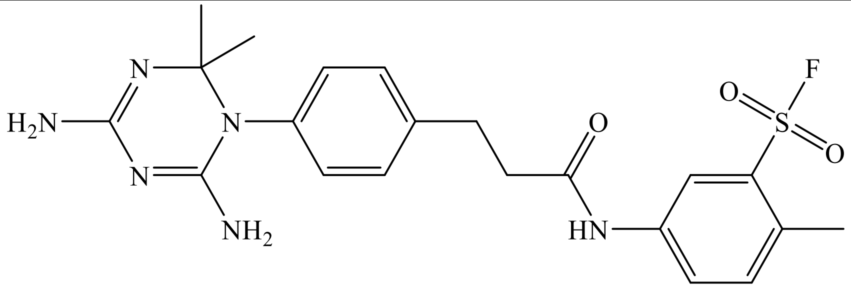

**306** (NCI code: 212212)

**Figure SM3:** Chemical structures of hits ordered from the NCI.

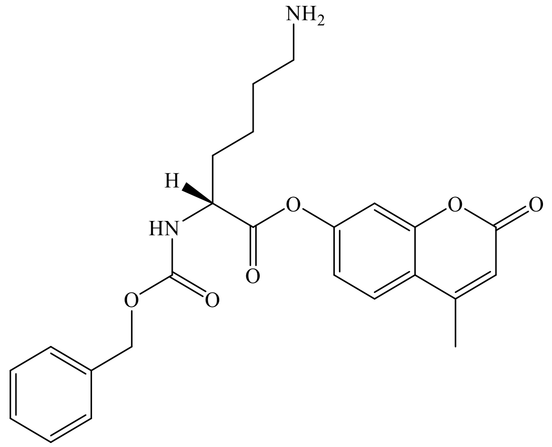


**307 (**NCI code: 247461)

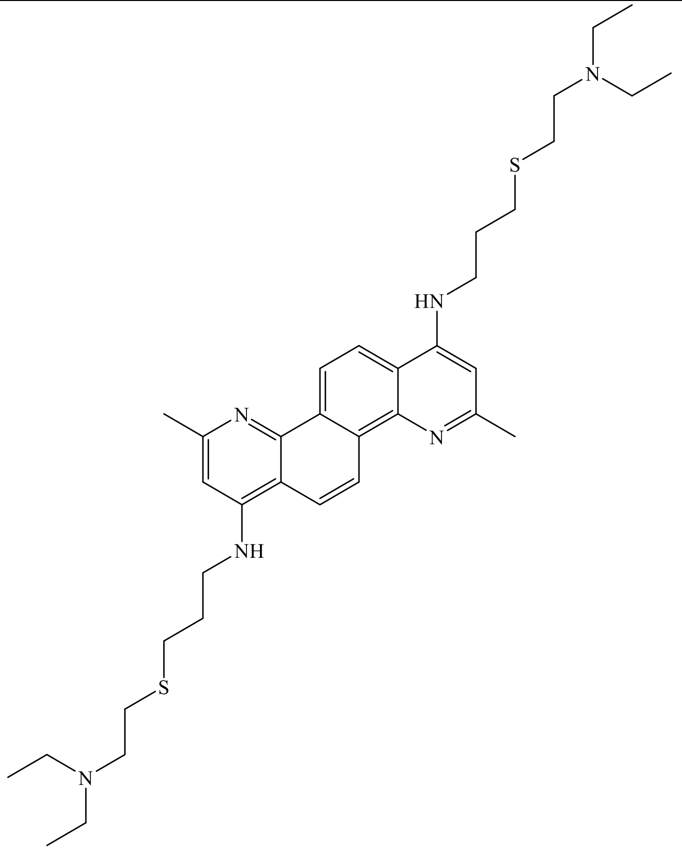


**308 (**NCI code: 325622)

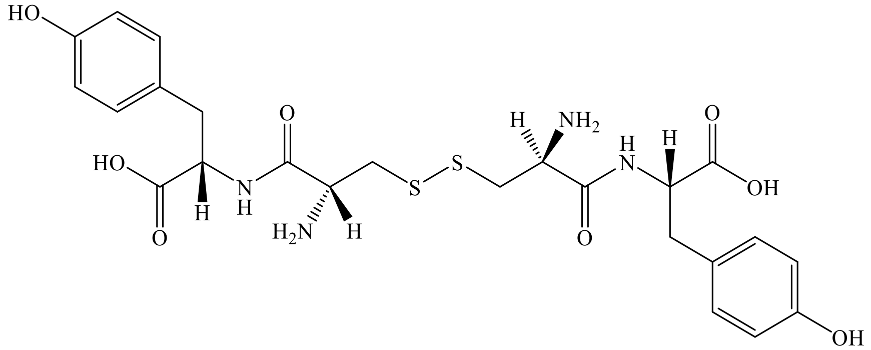


**309 (**NCI code: 333714)

**Figure SM3:** Chemical structures of hits ordered from the NCI

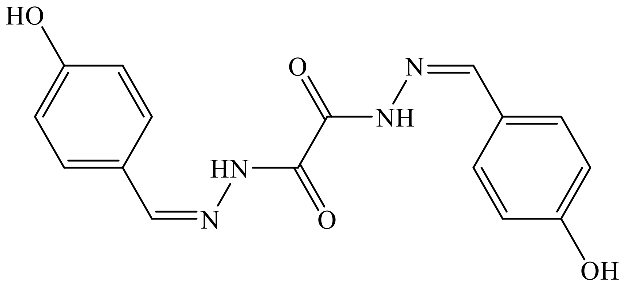


**310** (NCI code: 54104)

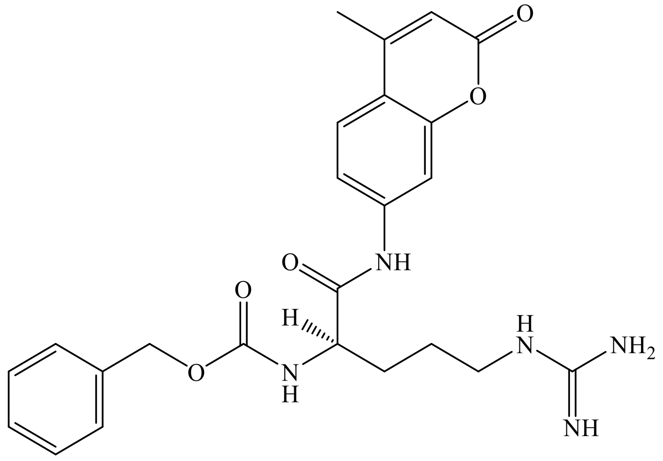


**311** (NCI code: 374899)

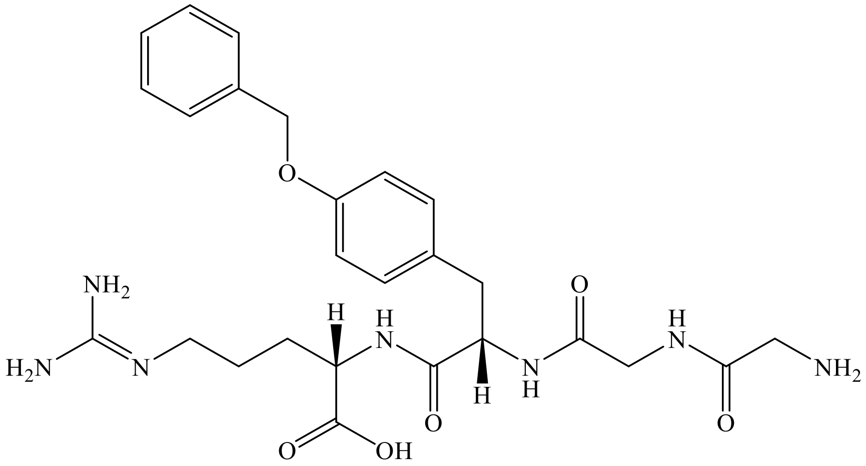


**312** (NCI code: 375794)

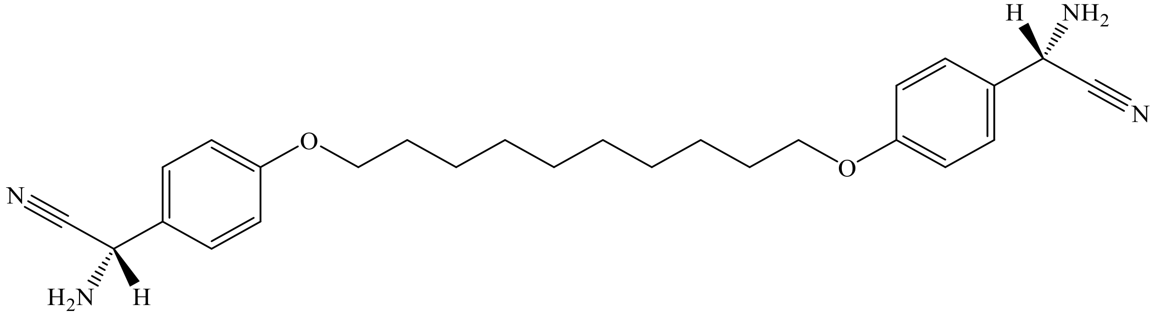


**313** (NCI code: 681721)

**Figure SM3:** Chemical structures of hits ordered from the NCI

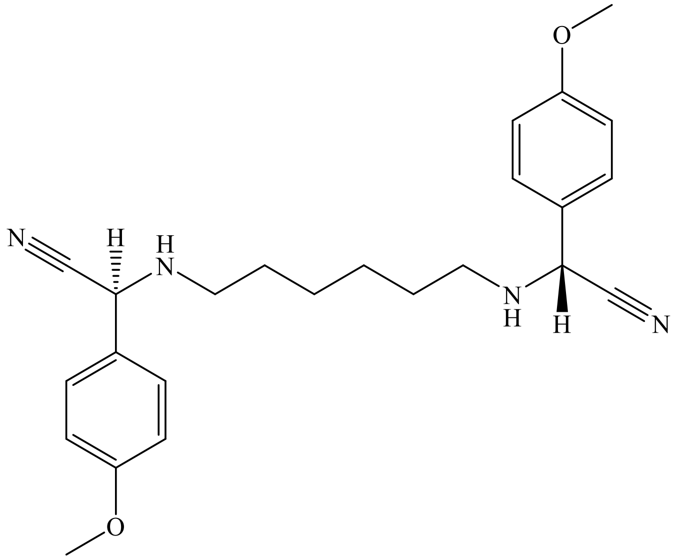


**314 (**NCI code: 681731)

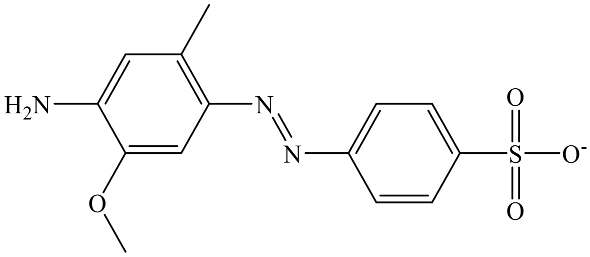


**315 (**NCI code: 45525)

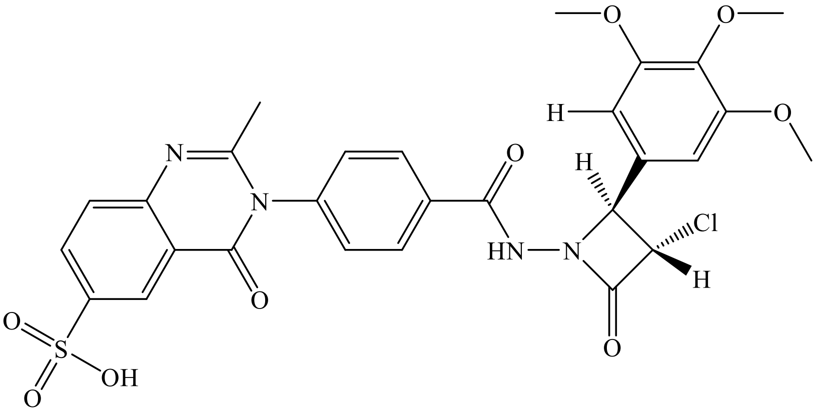


**316 (**NCI code: 714540)

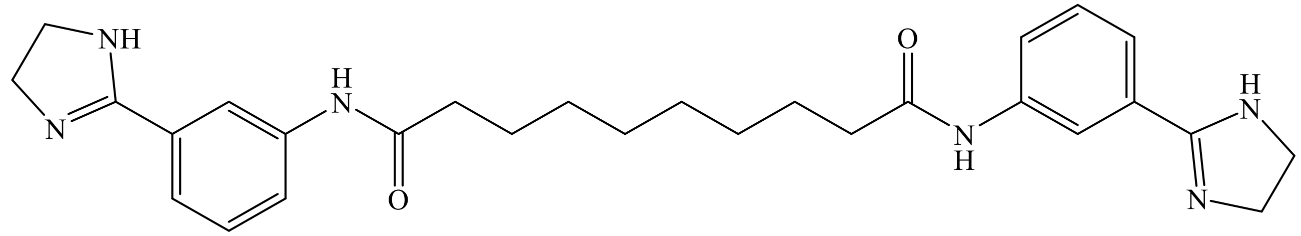


**317 (**NCI code: 35840)

**Figure SM3:** Chemical structures of hits ordered from the NCI

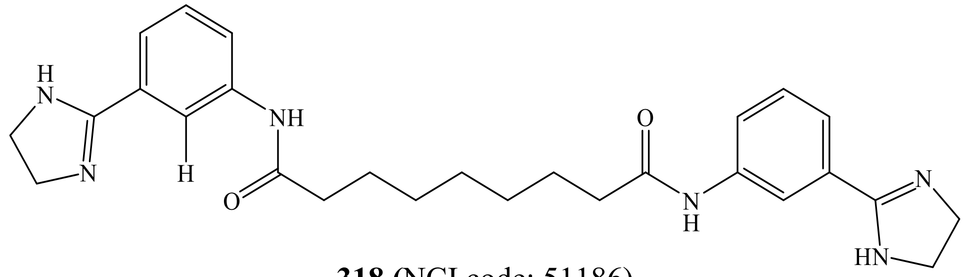

**318 (**NCI code: 51186)

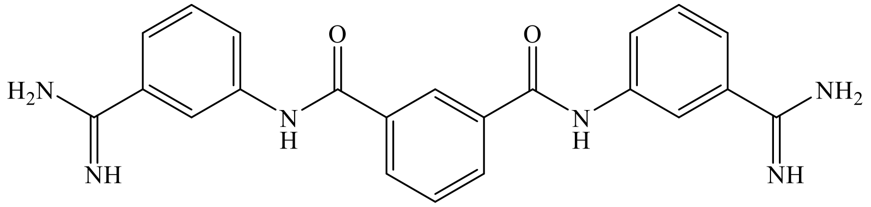

**319 (**NCI code: 55149)

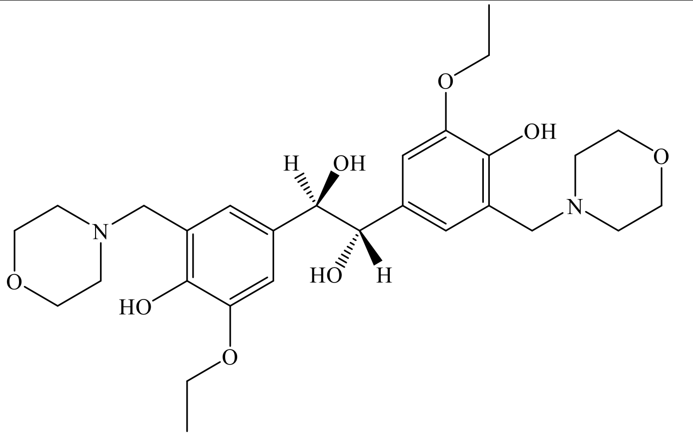

**320 (**NCI code: 55230)

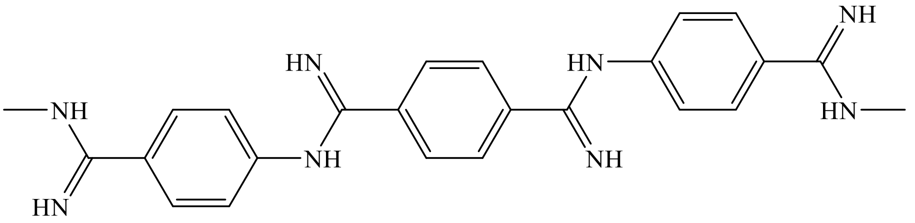

**321 (**NCI code: 57155)

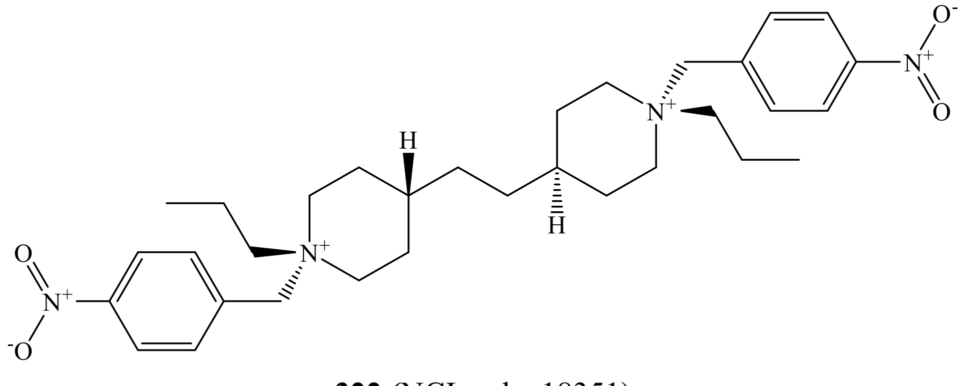

**322 (**NCI code: 18351)

**Figure SM3:** Chemical structures of hits ordered from the NCI

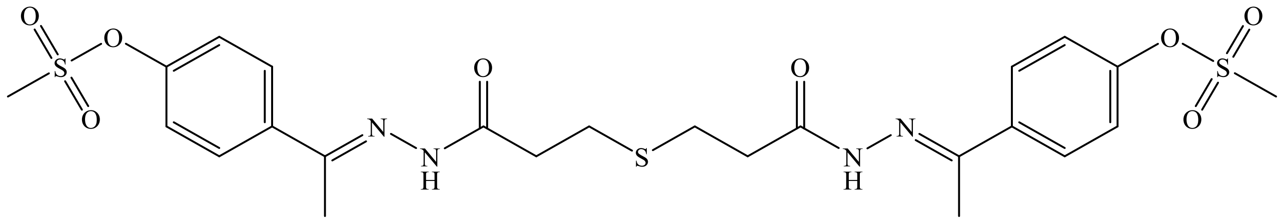


**323 (**NCI code: 59228)

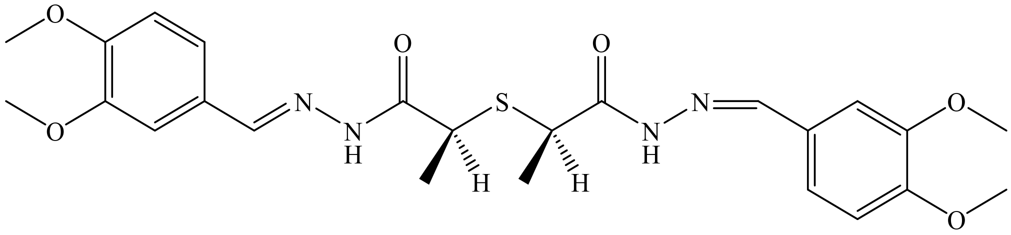


**324 (**NCI code: 60850)

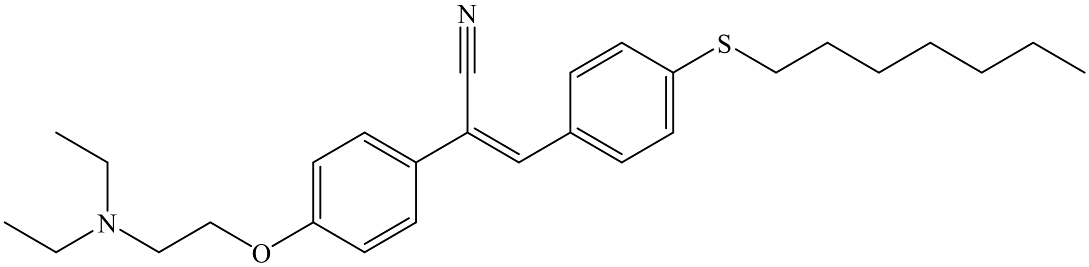


**325 (**NCI code: 64983)

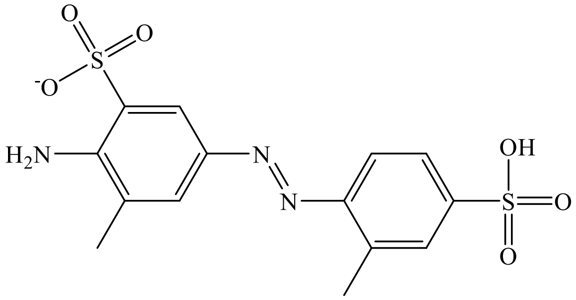


**326 (**NCI code: 71957)

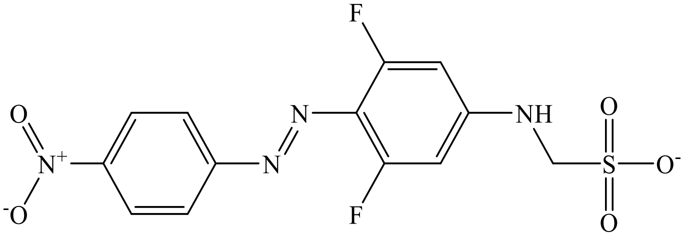


**327 (**NCI code: 81291)

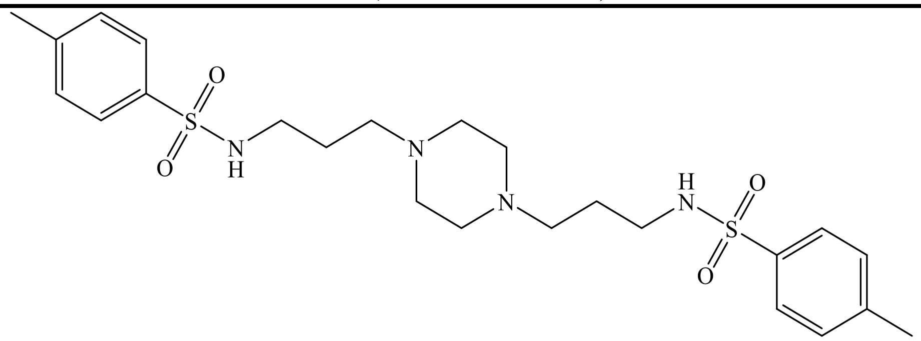


**328 (**NCI code: 95623)

**Figure SM3:** Chemical structures of hits ordered from the NCI

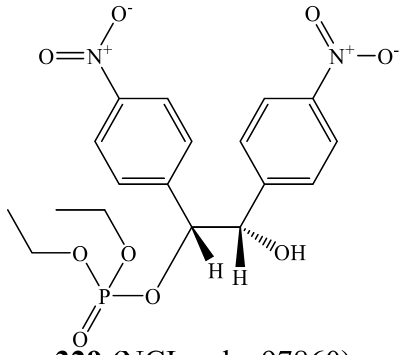

**329 (**NCI code: 97860)

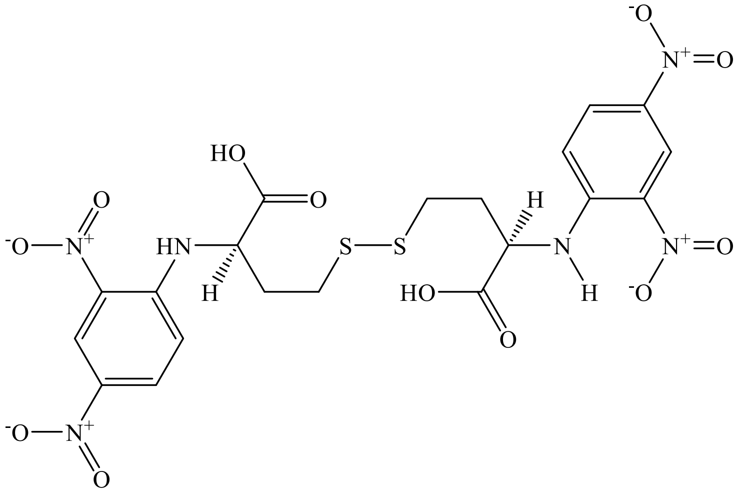

**330 (**NCI code: 97902)

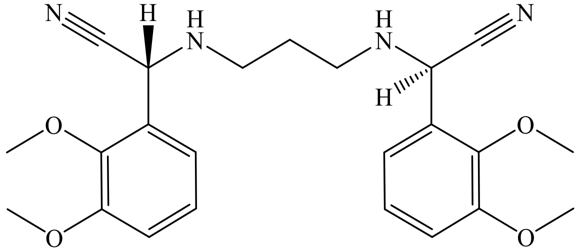

**331 (**NCI code: 101100)

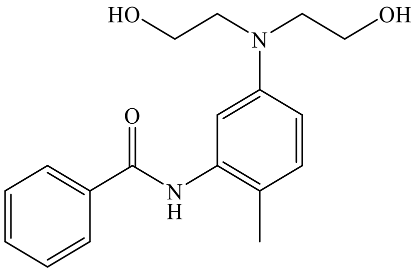

**332 (**NCI code: 109609)

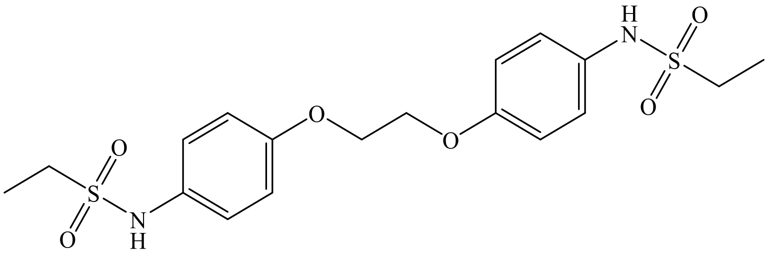

**333 (**NCI code: 32097)

**Figure SM3:** Chemical structures of hits ordered from the NCI

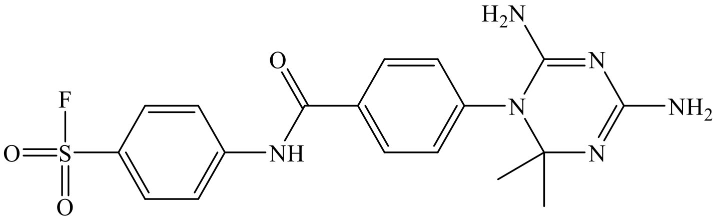

**334 (**NCI code: 113226)

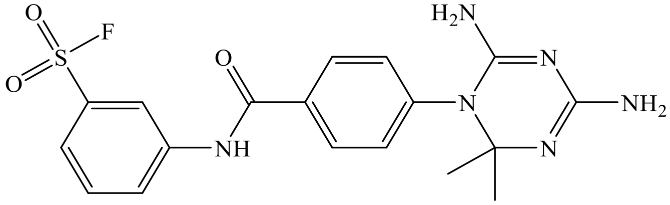

**335 (**NCI code: 113906)

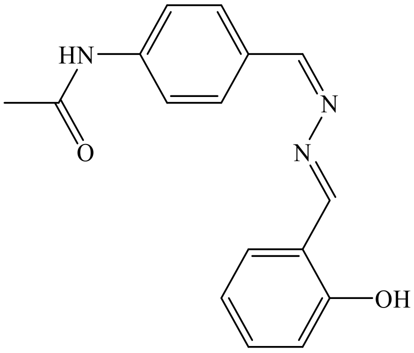

**336 (**NCI code: 119798)

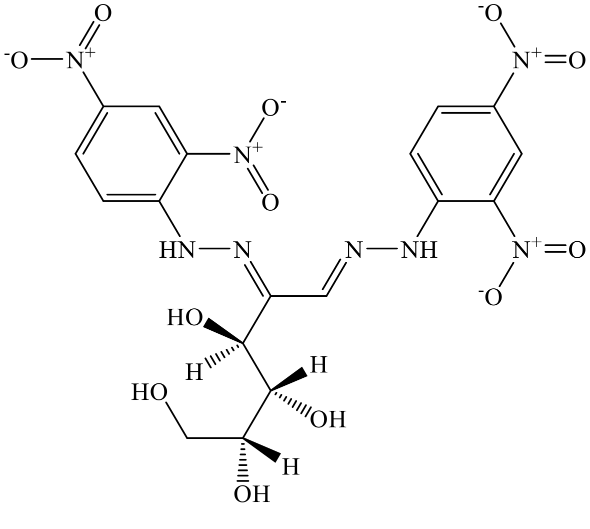

**337 (**NCI code: 121360)

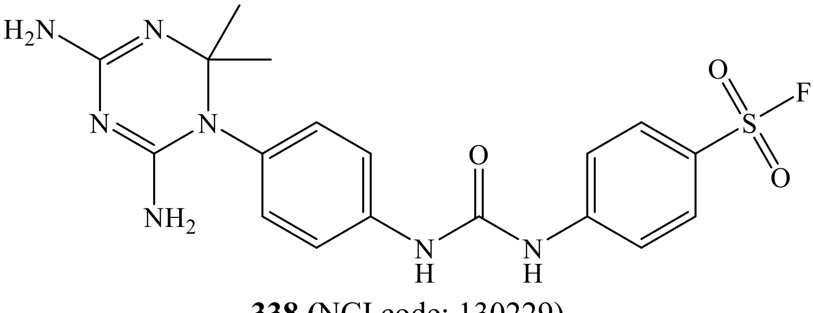

**338 (**NCI code: 130229)

**Figure SM3:** Chemical structures of hits ordered from the NCI

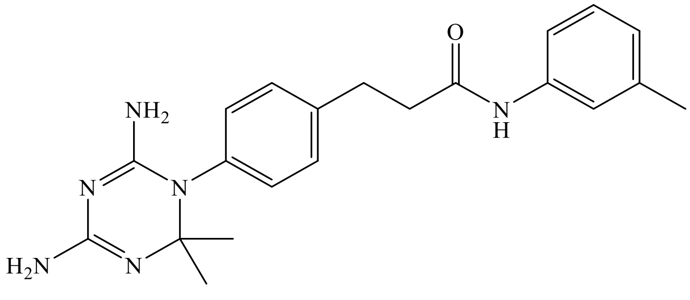

**339 (**NCI code: 133072)

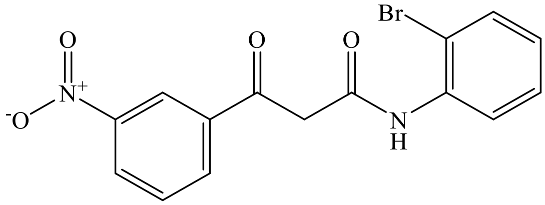

**340 (**NCI code: 157395)

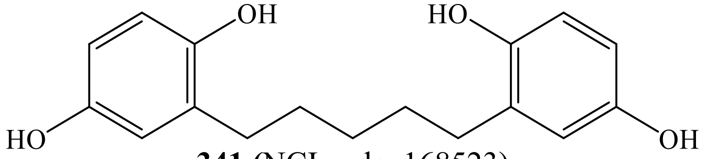

**341 (**NCI code: 168523)

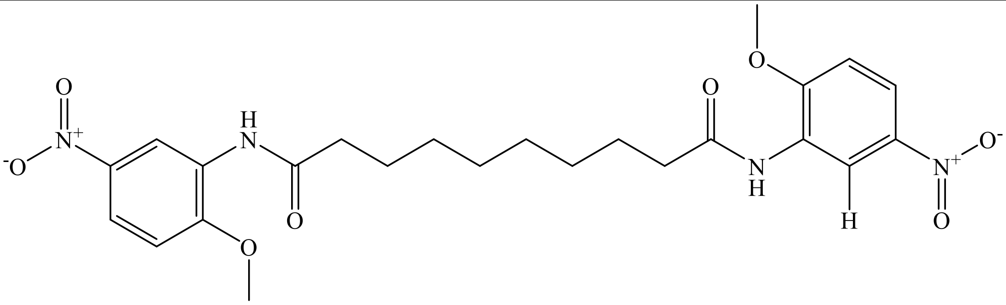

**342 (**NCI code: 203913)

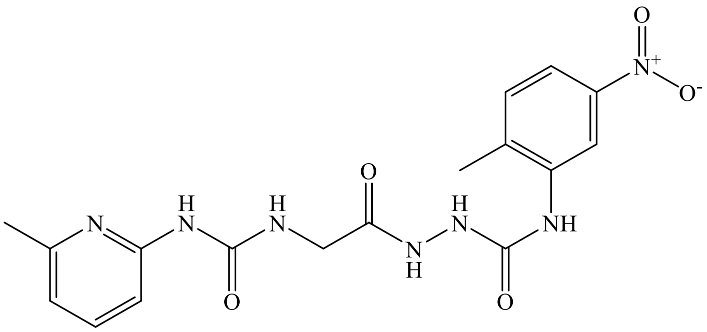

**343 (**NCI code: 213775)

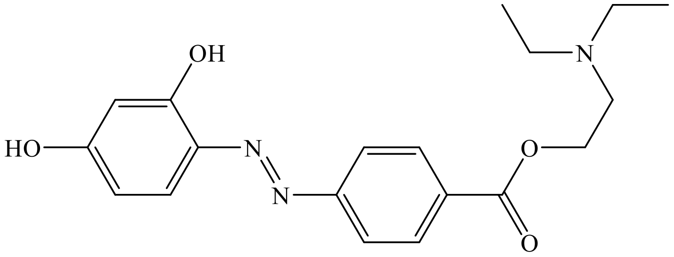

**344 (**NCI code: 32453)

**Figure SM3:** Chemical structures of hits ordered from the NCI

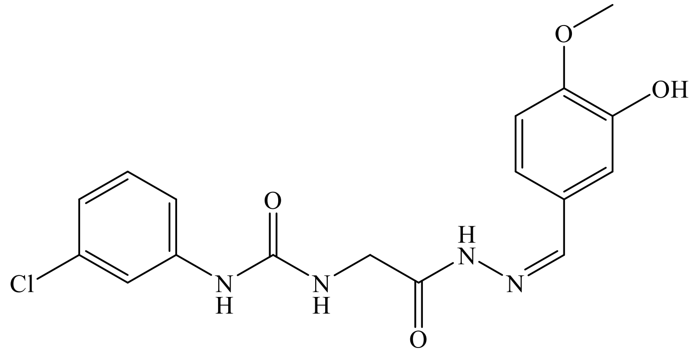


**345 (**NCI code: 216807)

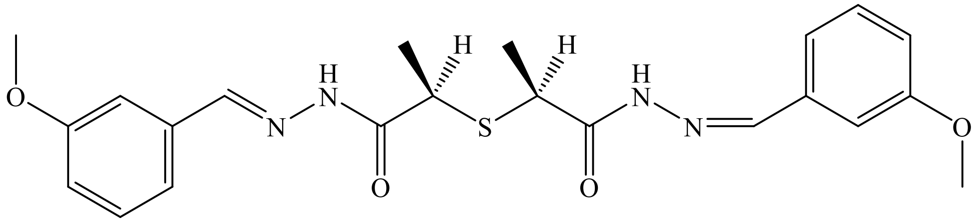


**346 (**NCI code: 56737)

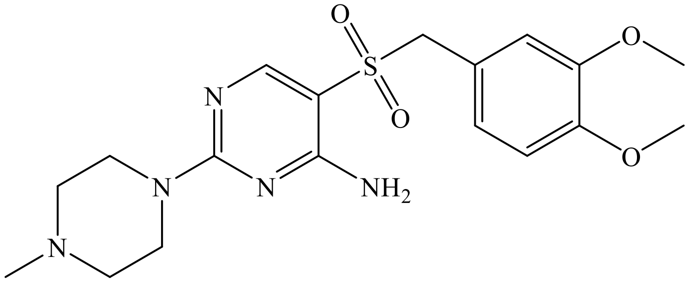


**347 (**NCI code: 270414)

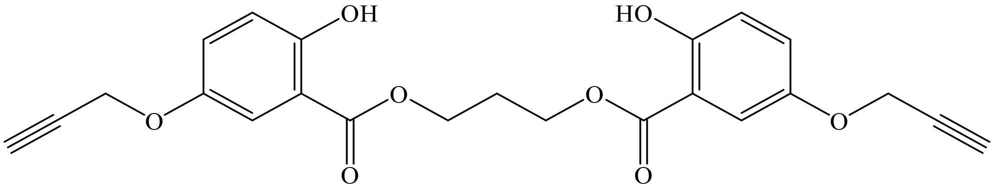


**348 (**NCI code: 370162)

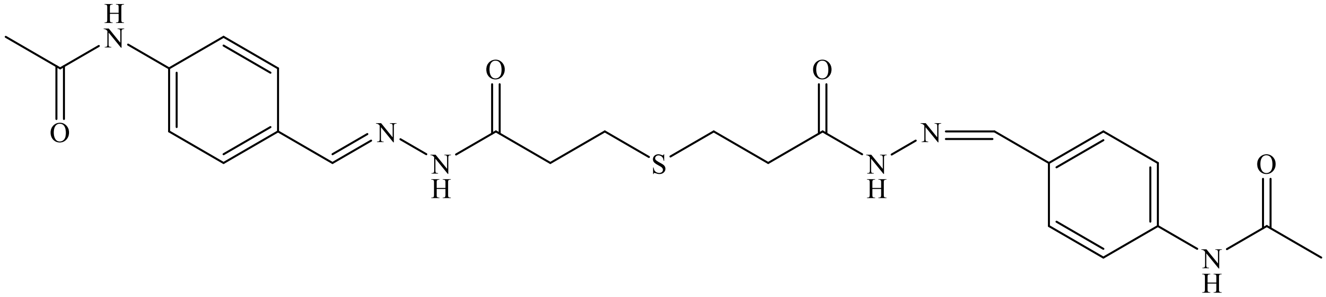


**349 (**NCI code: 54427)

**Figure SM3:** Chemical structures of hits ordered from the NCI

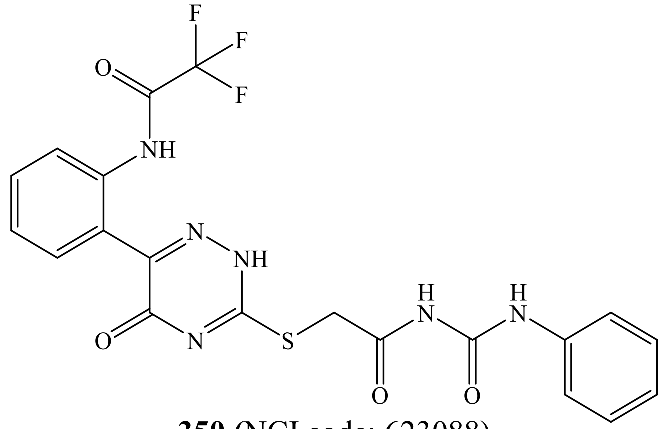

**350 (**NCI code: 623088)

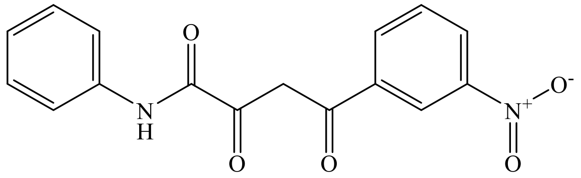

**351 (**NCI code: 631831)

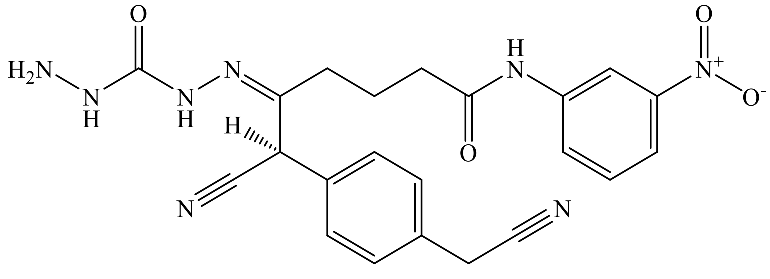

**352 (**NCI code: 632240)

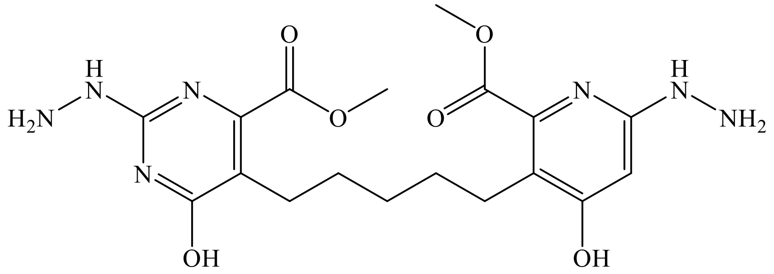

**353 (**NCI code: 635116)

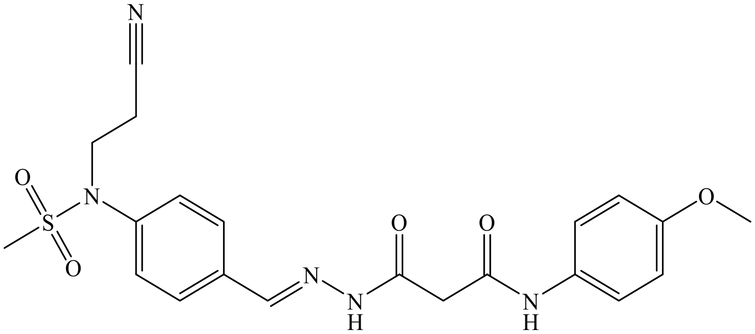

**354 (**NCI code: 638220)

**Figure SM3:** Chemical structures of hits ordered from the NCI

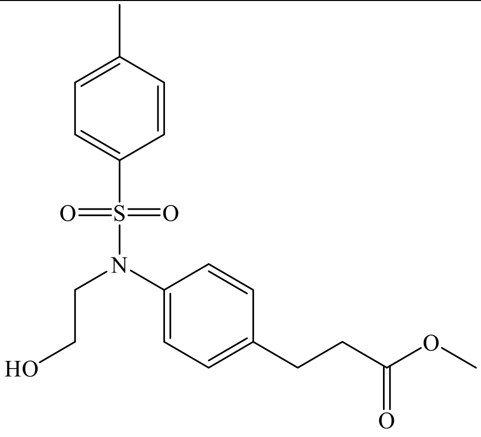


**355 (**NCI code: 34702)

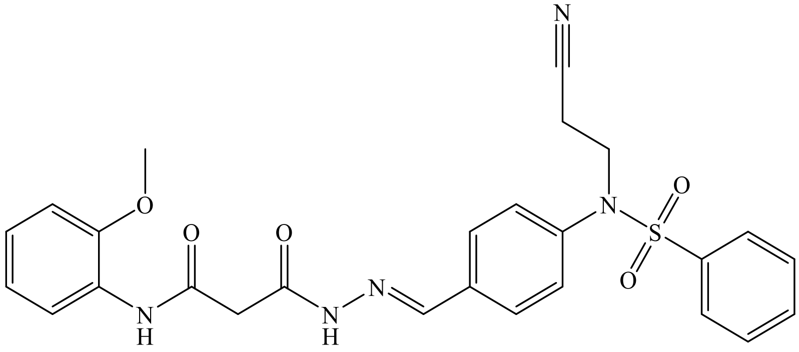


**356 (**NCI code: 638222)

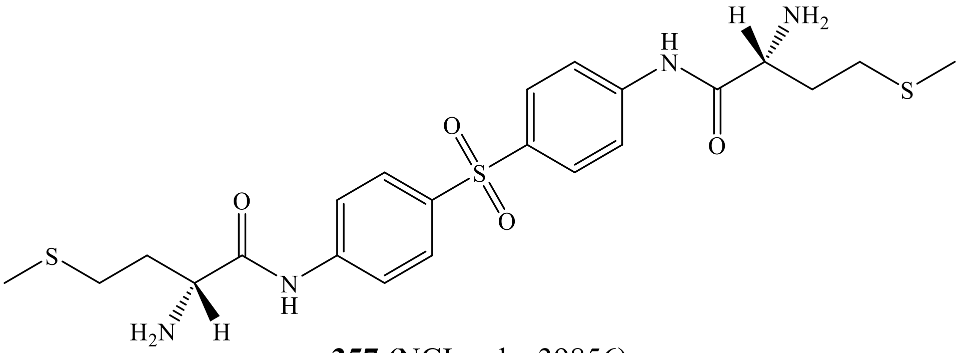


**357 (**NCI code: 39856)

**Figure SM3:** Chemical structures of hits ordered from the NCI

**Hit 287**

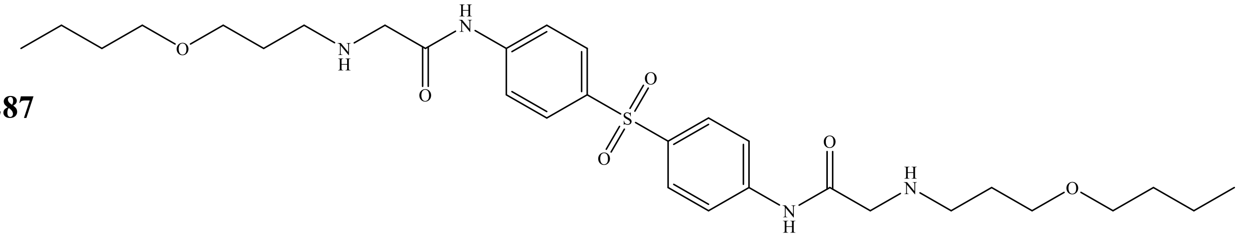

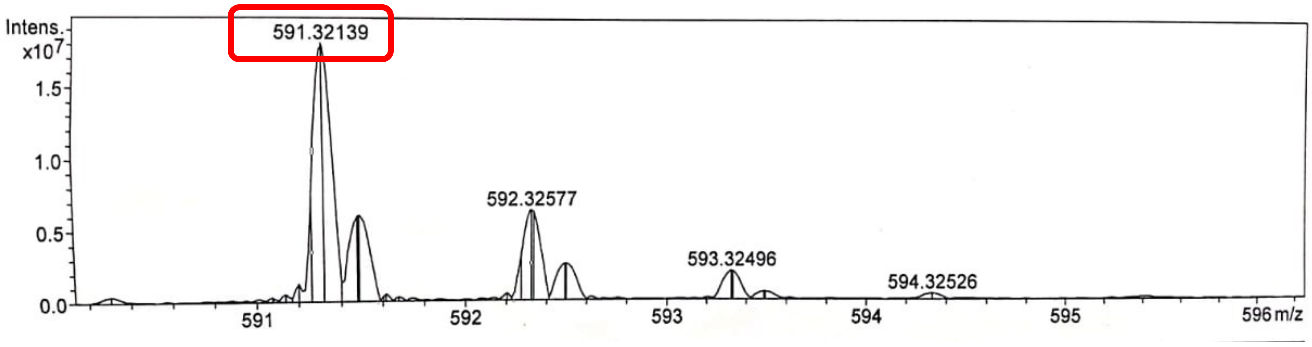


**HRMS-ESI** *m/z* calcd for $C_{30}H_{47}N_4O_6S_1^+$, $[M+H]^+$: 591.32108, found: 591.32139.

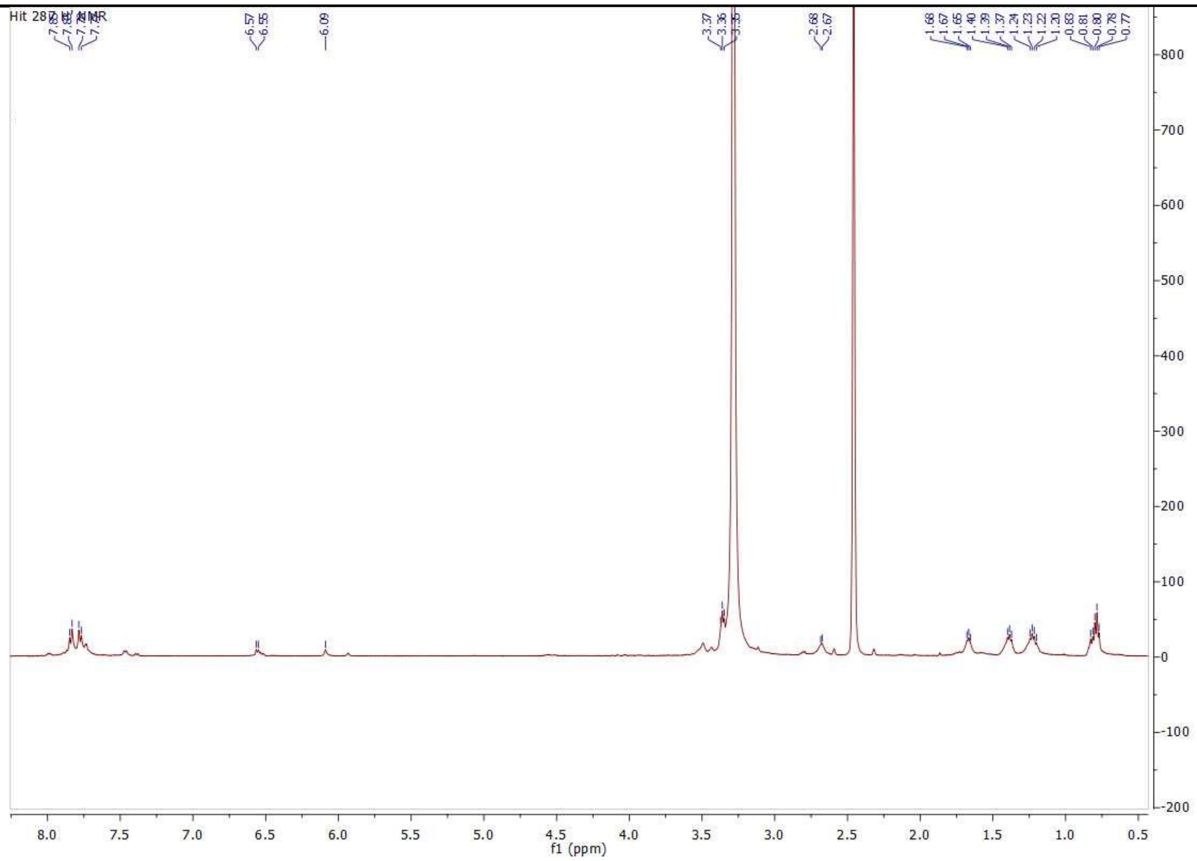


**Figure SM4:** HRMS and $^1$H-NMR of compound **287**.

**Hit 298**

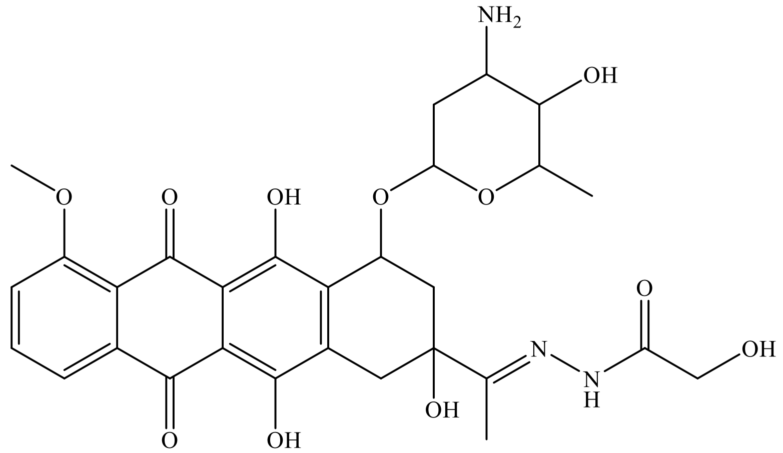


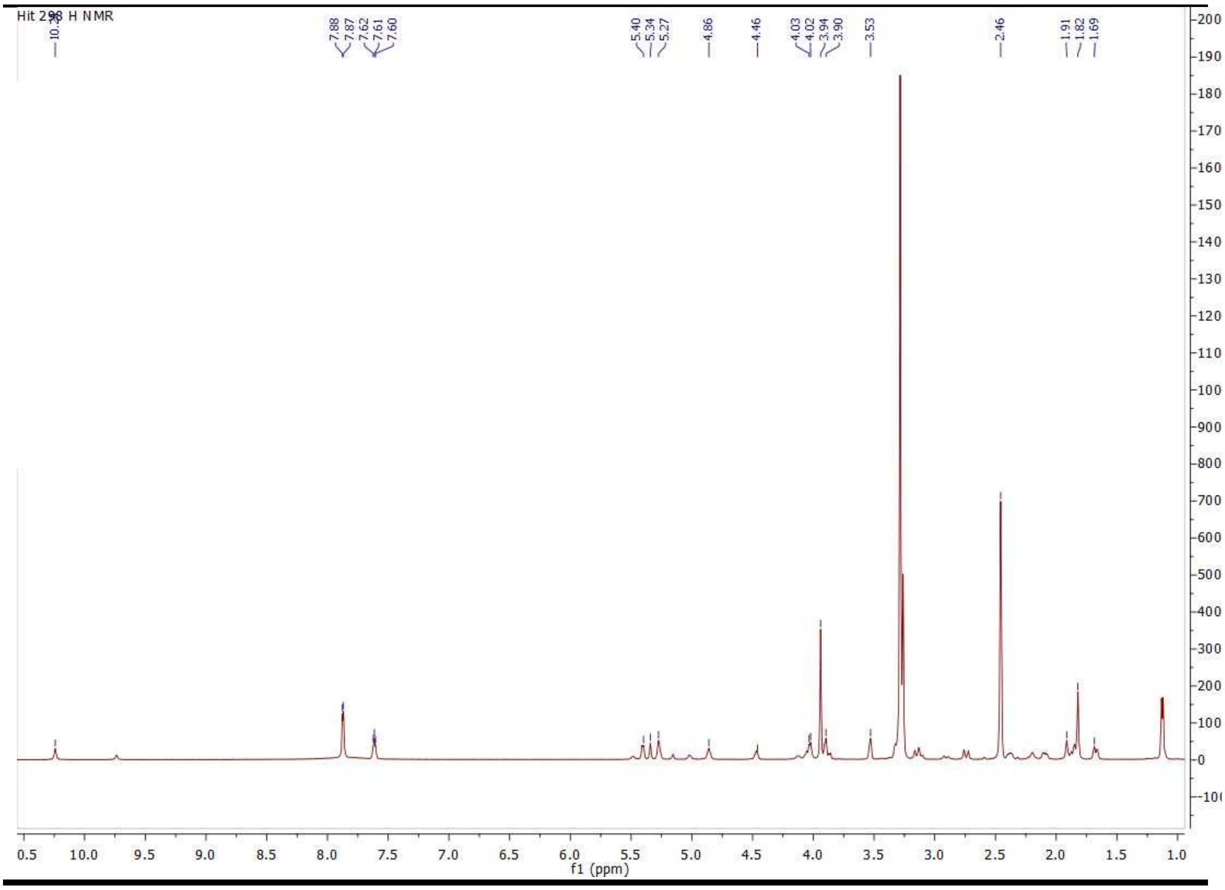


**Figure SM5:** $^{1}$H-NMR of compound **298**.

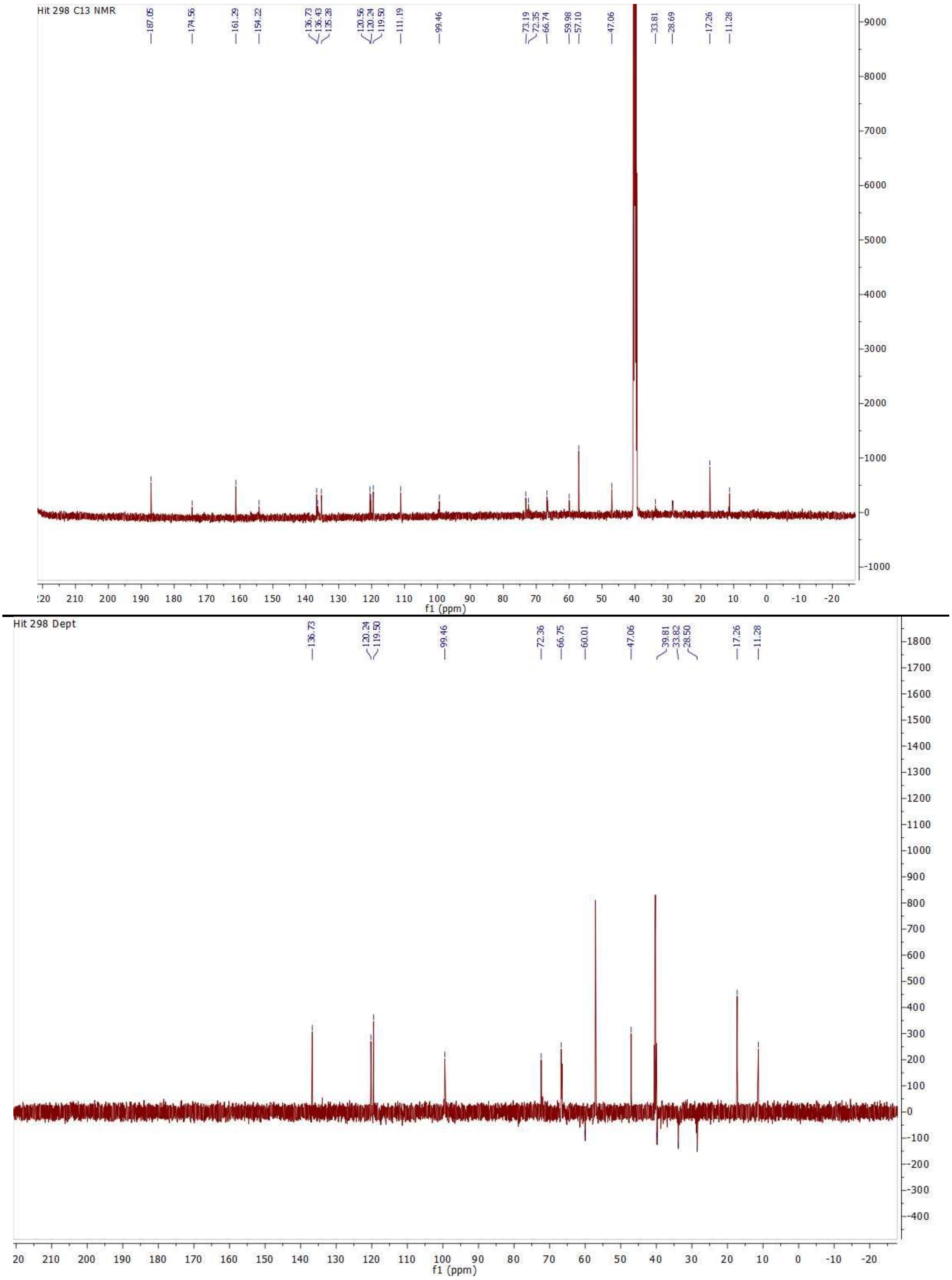


**Figure SM6:** $^{13}$C-NMR and dept135 of compound **298**.

**Hit 304**

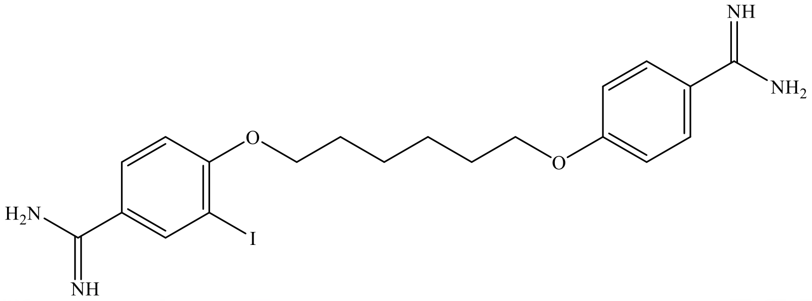


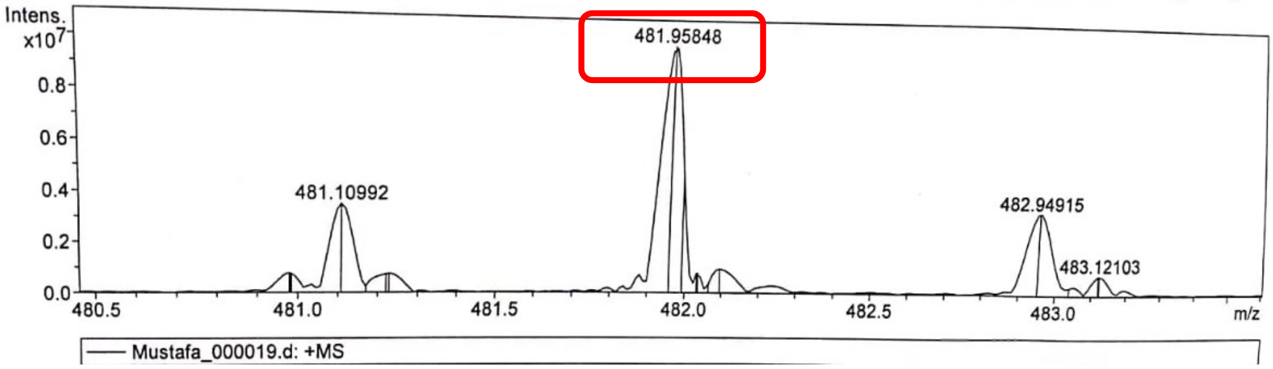


**HRMS-ESI** *m/z* calcd for $C_{20}H_{26}IN_4O_2^+$, $[M+H]^+$: 481.10950, found: 481.95848.

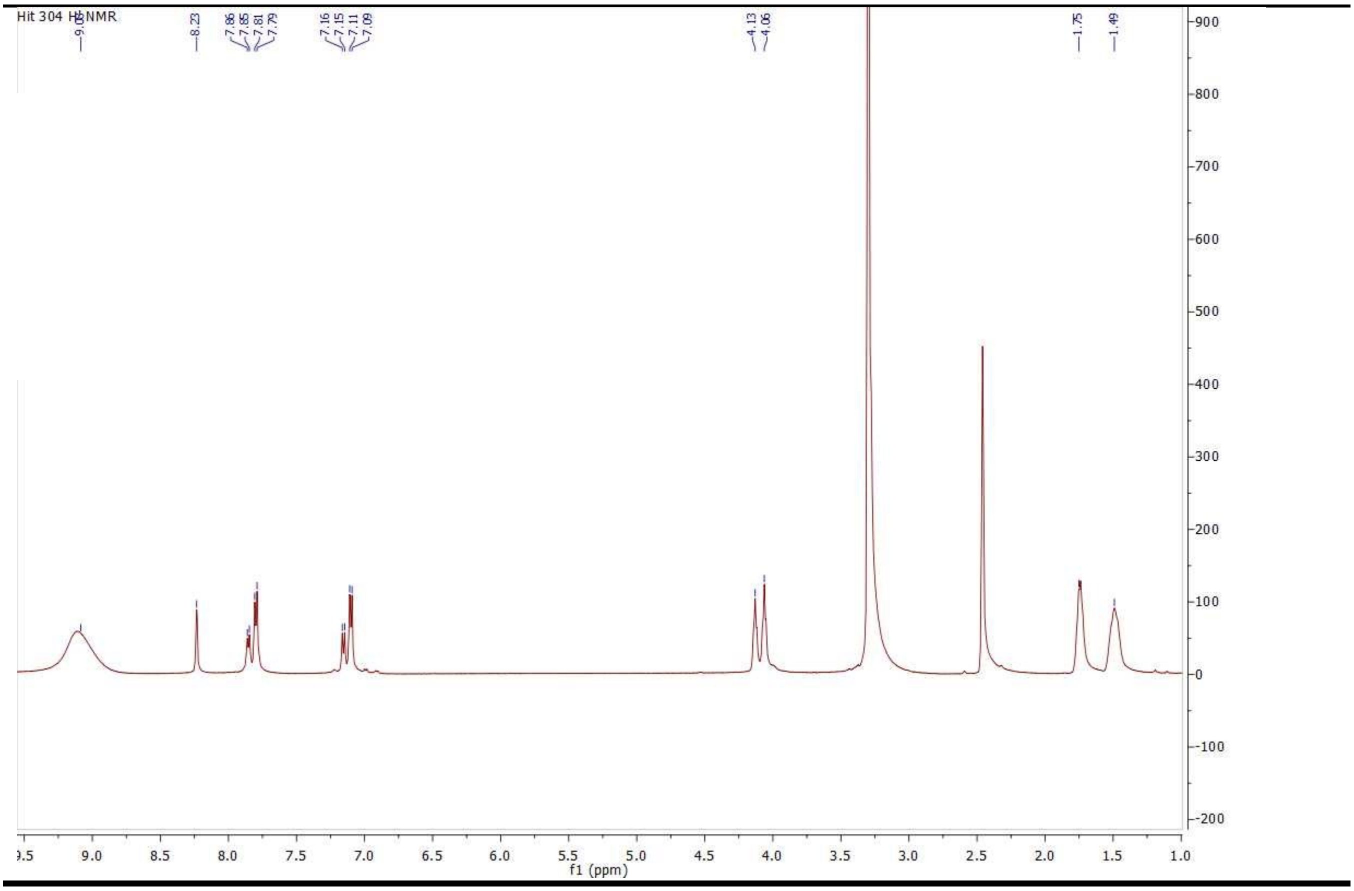


**Figure SM7:** $^1$H-NMR and HRMS of Hit **304**.

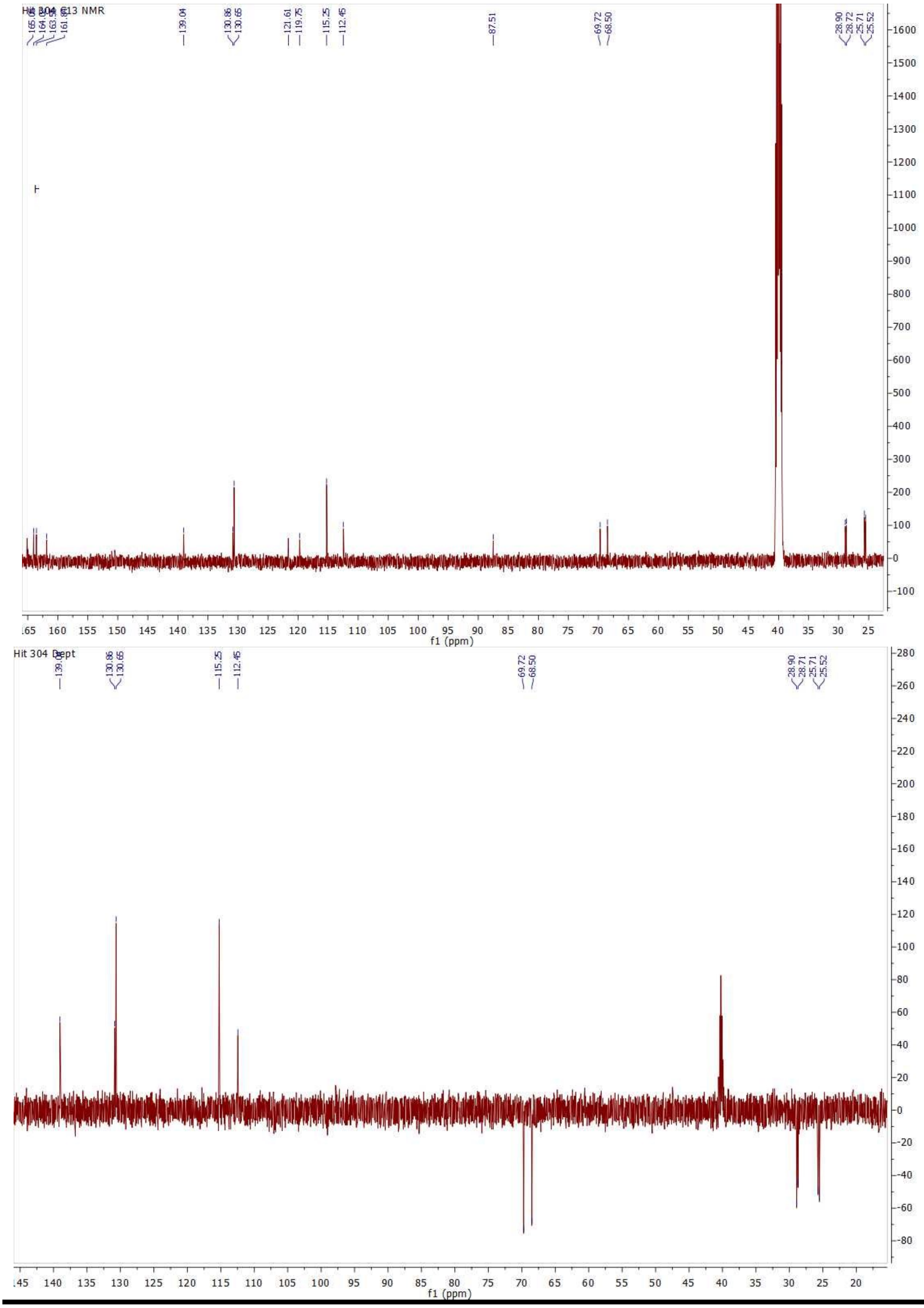


**Figure SM8:** $^{13}$C-NMR and dept135 of Hit **304**.

**Table SM10**: ML Training list compounds

| Compound ID | SMILES | Activity class | Source |
|---|---|---|---|
| **ML- 1** | O=C(OC(C([H])([H])[H])(C([H])([H])[H])C([H])([H])[H])N4C([H])([H])C([H])([H])N(C(=[S])[S]C([H])([H])C=1N=NN(C=1[H])C([H])([H])C3=C([H])C(=O)OC=2C([H])=C(O[H])C([H])=C([H])C=23)C([H])([H])C4([H])[H] | Active | 27 |
| **ML- 2** | O=C(N1C([H])([H])C([H])([H])C([H])(N([H])[H])C([H])([H])C1([H])[H])C([H])([H])N(/C2=C(\[H])C([H])=C(C#N)C([H])=C2[H])C([H])([H])C3=C([H])C([H])=C(C([H])=C3[H])C([H])([H])[H] | Active | 33 |
| **ML- 3** | O=C(OC(C([H])([H])[H])(C([H])([H])[H])C([H])([H])[H])N4C([H])([H])C([H])([H])N(C(=[S])[S]C([H])([H])C=1N=NN(C=1[H])C([H])([H])C3=C([H])C(=O)OC=2C([H])=C([Cl])C([H])=C([H])C=23)C([H])([H])C4([H])[H] | Active | 27 |
| **ML- 4** | O=C(N1C([H])([H])C([H])([H])C([H])(N([H])[H])C([H])([H])C1([H])[H])C([H])([H])N(/C2=C(\[H])C([H])=C(C#N)C([H])=C2[H])C([H])([H])C3=C([H])C([H])=C([H])C([H])=C3[H] | Active | 33 |
| **ML- 5** | O=C(N1C([H])([H])C([H])([H])C([H])(N([H])[H])C([H])([H])C1([H])[H])C([H])([H])N(/C2=C(\[H])C([H])=C(C#N)C([H])=C2[H])C([H])([H])C3=C([H])C([H])=C4/C(=C3/[H])C([H])=NN4C([H])([H])[H] | Active | 33 |
| **ML- 6** | O=C(OC(C([H])([H])[H])(C([H])([H])[H])C([H])([H])[H])N4C([H])([H])C([H])([H])N(C(=[S])[S]C([H])([H])C=1N=NN(C=1[H])C([H])([H])C3=C([H])C(=O)OC=2C([H])=C(N([H])[H])C([H])=C([H])C=23)C([H])([H])C4([H])[H] | Active | 27 |
| **ML- 7** | O=C(N1C([H])([H])C([H])([H])C([H])(C([H])([H])C1([H])[H])C([H])([H])N([H])[H])C([H])([H])N(/C2=C(\[H])C([H])=C(C#N)C([H])=C2[H])C([H])([H])C3=C([H])C([H])=C(C([H])=C3[H])C([H])([H])[H] | Active | 33 |
| **ML- 8** | O=C(N1C([H])([H])C([H])([H])C([H])(C([H])([H])C1([H])[H])C([H])([H])N([H])[H])C([H])([H])N(/C2=C(\[H])C([H])=C(C#N)C([H])=C2[H])C([H])([H])C3=C([H])C([H])=C([Cl])C([H])=C3[H] | Active | 33 |
| **ML- 9** | O=C(N1C([H])([H])C([H])([H])C([H])(C([H])([H])C1([H])[H])C([H])([H])N([H])[H])C([H])([H])N(/C2=C(\[H])C([H])=C(C#N)C([H])=C2[H])C([H])([H])C3=C([H])C([H])=C([F])C([H])=C3[H] | Active | 33 |
| **ML- 10** | O=C(N1C([H])([H])C([H])([H])C2(C([H])([H])C1([H])[H])C([H])([H])N([H])C2([H])[H])C([H])([H])N(/C3=C(\[H])C([H])=C(C#N)C([H])=C3[H])C([H])([H])C4=C([H])C([H])=C(OC([H])([H])[H])C([F])=C4[H] | Active | 33 |

**Table SM10**: ML Training list compounds

| Compound ID | SMILES | Activity class | Source |
|---|---|---|---|
| **ML- 11** | N#C/C4=C(\[H])C([H])=C(/C2=C(/N=C(/[H])C(OC([H])([H])[C@]1([H])C([H])([H])C([H])([H])N([H])C1([H])[H])=C2[H])C3=C([H])C([H])=C(C([H])=C3[H])C([H])([H])[H])C([H])=C4[H] | Active | 34 |
| **ML- 12** | N#C/C1=C(\[H])C([H])=C(C([H])=C1[H])[C@@]4([H])[C@]([H])(C2=C([H])C([H])=C(C([H])=C2[H])C([H])([H])[H])C([H])([H])N(C(=O)[C@]3([H])C([H])([H])C([H])([H])N([H])C([H])([H])C3([H])[H])C4([H])[H] | Active | 34 |
| **ML- 13** | N#C/C1=C(\[H])C([H])=C(C([H])=C1[H])[C@@]4([H])[C@]([H])(C2=C([H])C([H])=C(C([H])=C2[H])C([H])([H])[H])C([H])([H])N(C(=O)[C@@]3([H])C([H])([H])C([H])([H])C([H])([H])[C@]([H])(N([H])[H])C3([H])[H])C4([H])[H] | Active | 34 |
| **ML- 14** | N#C/C1=C(\[H])C([H])=C(C([H])=C1[H])[C@@]4([H])[C@]([H])(C2=C([H])C([H])=C(C([H])=C2[H])C([H])([H])[H])C([H])([H])N(C(=O)C([H])([H])[C@]3([H])C([H])([H])C([H])([H])N([H])C([H])([H])C3([H])[H])C4([H])[H] | Active | 34 |
| **ML- 15** | N#C/C1=C(\[H])C([H])=C(C([H])=C1[H])[C@@]4([H])[C@]([H])(C2=C([H])C([H])=C(C([H])=C2[H])C([H])([H])[H])C([H])([H])N(C(=O)C([H])([H])[C@]3([H])C([H])([H])N([H])C([H])([H])C([H])([H])C3([H])[H])C4([H])[H] | Active | 34 |
| **ML- 16** | N#C/C1=C(\[H])C([H])=C(C([H])=C1[H])[C@@]4([H])[C@]([H])(C2=C([H])C([H])=C(C([H])=C2[H])C([H])([H])[H])C([H])([H])N(C(=O)C([H])([H])[C@]3([H])C([H])([H])C([H])([H])[C@]([H])(N([H])[H])C([H])([H])C3([H])[H])C4([H])[H] | Active | 34 |
| **ML- 17** | N#C/C1=C(\[H])C([H])=C(C([H])=C1[H])[C@@]4([H])[C@]([H])(C2=C([H])C([H])=C(C([H])=C2[H])C([H])([H])[H])C([H])([H])N(C(=O)[C@]3([H])C([H])([H])C([H])([H])[C@@]([H])(C([H])([H])C3([H])[H])C([H])([H])N([H])[H])C4([H])[H] | Active | 34 |
| **ML- 18** | N#C/C1=C(\[H])C([H])=C(C([H])=C1[H])[C@@]4([H])[C@]([H])(C2=C([H])C([H])=C(C([H])=C2[H])C([H])([H])[H])C([H])([H])N(C(=O)[C@@]3([H])C([H])([H])C([H])([H])[C@]([H])(C([H])([H])C3([H])[H])C([H])([H])N([H])C([H])([H])[H])C4([H])[H] | Active | 34 |
| **ML- 19** | N#C/C1=C(\[H])C([H])=C(C([H])=C1[H])[C@@]4([H])[C@]([H])(C2=C([H])C([H])=C(/N=C2/[H])OC([H])([H])[H])C([H])([H])N(C(=O)C([H])([H])[C@@]3([H])C([H])([H])C([H])([H])[C@@]([H])(N([H])[H])C([H])([H])C3([H])[H])C4([H])[H] | Active | 34 |
| **ML- 20** | N#C/C1=C(\[H])C([H])=C(C([H])=C1[H])[C@@]5([H])[C@]([H])(C2=C([H])C([H])=C3/C(=C2/[H])C([H])=NN3C([H])([H])[H])C([H])([H])N(C(=O)[C@@]4([H])C([H])([H])C([H])([H])[C@@]([H])(N([H])[H])C([H])([H])C4([H])[H])C5([H])[H] | Active | 34 |

**Table SM10**: ML Training list compounds

| Compound ID | SMILES | Activity class | Source |
|---|---|---|---|
| **ML- 21** | [S]=C([S]C([H])([H])C=1N=NN(C=1[H])C([H])([H])C=2C([H])=C([H])C(=C([H])C=2[H])C([H])([H])[H])N3C([H])([H])C([H])([H])N(C(=O)OC(C([H])([H])[H])(C([H])([H])[H])C([H])([H])[H])C([H])([H])C3([H])[H] | Active | 28 |
| **ML- 22** | N#C/C1=C(\[H])C([H])=C(C([H])=C1[H])[C@@]5([H])[C@]([H])(C2=C([H])C([H])=C3/C(=C2/[H])C([H])=NN3C([H])([H])[H])C([H])([H])N(C(=O)C4=C([H])C([H])=C(C([H])=C4[H])C([H])([H])N([H])[H])C5([H])[H] | Active | 34 |
| **ML- 23** | N#C/C1=C(\[H])C([H])=C(C([H])=C1[H])[C@@]5([H])[C@]([H])(C2=C([H])C([H])=C3/C(=C2/[H])C([H])=NN3C([H])([H])[H])C([H])([H])N(C(=O)[C@]4([H])C([H])([H])C([H])([H])[C@@]([H])(C([H])([H])C4([H])[H])C([H])([H])N([H])[H])C5([H])[H] | Active | 34 |
| **ML- 24** | N#C/C1=C(\[H])C([H])=C(C([H])=C1[H])[C@@]5([H])[C@]([H])(C2=C([H])C([H])=C3/C(=C2/[H])C([H])=NN3C([H])([H])[H])C([H])([H])N(C(=O)[C@@]4([H])C([H])([H])C([H])([H])[C@]([H])(C([H])([H])C4([H])[H])C([H])([H])N([H])C([H])([H])[H])C5([H])[H] | Active | 34 |
| **ML- 25** | N#C/C3=C(\[H])C([H])=C(N1N=C(C([H])=C1OC([H])([H])C([H])([H])C(O[H])(C([H])([H])[H])C([H])([H])[H])C(=O)N2C([H])([H])[C@]([H])(N([H])[H])C([H])([H])C([H])([H])C2([H])[H])C([H])=C3[H] | Active | 34 |
| **ML- 26** | N#C/C4=C(\[H])C([H])=C(N1N=C(C([H])=C1OC([H])([H])C=2ON=C(C=2[H])C([H])([H])C([H])([H])[H])C(=O)N3C([H])([H])[C@]([H])(N([H])[H])C([H])([H])C([H])([H])C3([H])[H])C([H])=C4[H] | Active | 34 |
| **ML- 27** | N#C/C5=C(\[H])C([H])=C(N1N=C(C([H])=C1OC([H])([H])C=2ON=C(C=2[H])C([H])([H])[H])C(=O)N4C([H])([H])[C@]3([H])[C@@]([H])(N([H])C([H])([H])C([H])([H])C3([H])[H])C4([H])[H])C([H])=C5[H] | Active | 34 |
| **ML- 28** | N#C/C4=C(\[H])C([H])=C(N1N=C(C([H])=C1OC([H])([H])C=2ON=C(C=2[H])C([H])([H])[H])C(=O)N3C([H])([H])[C@@]([H])(N([H])[H])C([H])([H])C3([H])[H])C([H])=C4[H] | Active | 34 |
| **ML- 29** | N#CC=4C([H])=C([H])C(/C2=C(/N=C(/[H])C(OC([H])([H])C1([H])C([H])([H])C([H])([H])N([H])C([H])([H])C1([H])[H])=C2[H])C=3C([H])=C([H])C(=NC=3[H])C([H])([H])[H])=C([H])C=4[H] | Active | 25 |
| **ML- 30** | [F]C=1C([H])=C([H])C(=C([H])C=1[F])C=3/N=C(/[H])C(OC([H])([H])C2([H])C([H])([H])C([H])([H])N([H])C([H])([H])C2([H])[H])=C([H])C=3C=4C([H])=C([H])C(C#N)=C([H])C=4[H] | Active | 25 |

**Table SM10**: ML Training list compounds

| Compound ID | SMILES | Activity class | Source |
|---|---|---|---|
| **ML- 31** | N#CC=4C([H])=C([H])C(/C2=C(/N=C(/[H])C(N([H])C([H])([H])[C@@]1([H])C([H])([H])C([H])([H])C([H])([H])N([H])C1([H])[H])=C2[H])C=3C([H])=C([H])C(=C([H])C=3[H])C([H])([H])[H])=C([H])C=4[H] | Active | 25 |
| **ML- 32** | N#CC=1C([H])=C([H])C(=C([H])C=1[H])C=3C([H])=C(OC([H])([H])C2([H])C([H])([H])C([H])([H])N([H])C([H])([H])C2([H])[H])C([H])=NC=3C=4C([H])=C([H])C(C#N)=C([H])C=4[H] | Active | 25 |
| **ML- 33** | N#CC=1C([H])=C([H])C(=C([H])C=1[H])C=3C([H])=C(OC([H])([H])C2([H])C([H])([H])C([H])([H])N([H])C([H])([H])C2([H])[H])C([H])=NC=3C=4C([H])=C([H])N=C([H])C=4[H] | Active | 25 |
| **ML- 34** | [F]C([F])([F])OC=1C([H])=C([H])C(=C([H])C=1[H])C=3/N=C(/[H])C(OC([H])([H])C2([H])C([H])([H])C([H])([H])N([H])C([H])([H])C2([H])[H])=C([H])C=3C=4C([H])=C([H])C(C#N)=C([H])C=4[H] | Active | 25 |
| **ML- 35** | [F]C([F])([F])C1=C([H])C([H])=C(C([H])=C1[H])C([H])([H])[S]C=3N=C2N=C(C([H])=C(N2N=3)N([H])N([H])[H])C([H])([H])[H] | Active | 31 |
| **ML- 36** | [F]C=1C([H])=C([H])C(=C([H])C=1[H])C=3/N=C(/[H])C(OC([H])([H])C2([H])C([H])([H])C([H])([H])N([H])C([H])([H])C2([H])[H])=C([H])C=3C=4C([H])=C([H])C(C#N)=C([H])C=4[H] | Active | 25 |
| **ML- 37** | N#CC=1C([H])=C([H])C(=C([H])C=1[H])C=3C([H])=C(OC([H])([H])C2([H])C([H])([H])C([H])([H])N([H])C([H])([H])C2([H])[H])C([H])=NC=3C=4C([H])=C([H])C([H])=C([H])C=4[H] | Active | 25 |
| **ML- 38** | [H]C([H])([H])C=1C([H])=C([H])C(=C([H])C=1[H])C=3/N=C(/[H])C(OC([H])([H])C2([H])C([H])([H])C([H])([H])N([H])C([H])([H])C2([H])[H])=NC=3C=4C([H])=C([H])C(=C([H])C=4[H])C([H])([H])[H] | Active | 25 |
| **ML- 39** | N#CC=4C([H])=C([H])C(/C2=C(/N=C(/[H])C(OC([H])([H])C1([H])C([H])([H])C([H])([H])N([H])C([H])([H])C1([H])[H])=C2[H])C=3C([H])=C([H])C(=C([H])C=3[H])C([H])([H])C([H])([H])[H])=C([H])C=4[H] | Active | 25 |
| **ML- 40** | N#CC=4C([H])=C([H])C(/C2=C(/N=C(/[H])C(OC([H])([H])C1([H])C([H])([H])C([H])([H])N([H])C([H])([H])C1([H])[H])=C2[H])C3=C([H])C([H])=C([H])C(=C3[H])C([H])([H])[H])=C([H])C=4[H] | Active | 25 |
| **ML- 41** | N#CC=4C([H])=C([H])C(/C2=C(/N=C(/[H])C(OC([H])([H])[C@@]1([H])C([H])([H])C([H])([H])C([H])([H])N([H])C1([H])[H])=C2[H])C=3C([H])=C([H])C(=C([H])C=3[H])C([H])([H])[H])=C([H])C=4[H] | Active | 25 |

| **Table SM10**: ML Training list compounds | | | |
|---|---|---|---|
| **Compound ID** | **SMILES** | **Activity class** | **Source** |
| **ML- 42** | [F]C=1C([H])=C([H])C(=C([F])C=1[H])C=3/N=C(/[H])C(OC([H])([H])C2([H])C([H])([H])C([H])([H])N([H])C([H])([H])C2([H])[H])=C([H])C=3C=4C([H])=C([H])C(C#N)=C([H])C=4[H] | Active | 25 |
| **ML- 43** | N#CC=4C([H])=C([H])C(/C3=C(\C=1C([H])=C([H])C(=C([H])C=1[H])C([H])([H])[H])C([H])=C([H])C(OC([H])([H])C2([H])C([H])([H])C([H])([H])N([H])C([H])([H])C2([H])[H])=C3[H])=C([H])C=4[H] | Active | 25 |
| **ML- 44** | [F]C([F])([F])C=1C([H])=C([H])C(=C([H])C=1[H])C=3/N=C(/[H])C(OC([H])([H])C2([H])C([H])([H])C([H])([H])N([H])C([H])([H])C2([H])[H])=C([H])C=3C=4C([H])=C([H])C(C#N)=C([H])C=4[H] | Active | 25 |
| **ML- 45** | [Br]C=1C([H])=C([H])C(=C([H])C=1[H])C=3/N=C(/[H])C(OC([H])([H])C2([H])C([H])([H])C([H])([H])N([H])C([H])([H])C2([H])[H])=NC=3C=4C([H])=C([H])C(C#N)=C([H])C=4[H] | Active | 25 |
| **ML- 46** | [F]/C1=N/C([H])=C(C([H])=C1[H])C=3/N=C(/[H])C(OC([H])([H])C2([H])C([H])([H])C([H])([H])N([H])C([H])([H])C2([H])[H])=C([H])C=3C=4C([H])=C([H])C(C#N)=C([H])C=4[H] | Active | 25 |
| **ML- 47** | N#CC=1C([H])=C([H])C(=C([H])C=1[H])C=3C([H])=C(OC([H])([H])C2([H])C([H])([H])C([H])([H])N([H])C([H])([H])C2([H])[H])C([H])=NC=3C=4C([H])=C([H])C([H])=NC=4[H] | Active | 25 |
| **ML- 48** | N#CC=4C([H])=C([H])C(/C2=C(/N=C(/[H])C(OC([H])([H])C1([H])C([H])([H])C([H])([H])N([H])C([H])([H])C1([H])[H])=C2[H])C=3C([H])=C([H])C(=C([H])C=3[H])C([H])([H])[H])=C([H])C=4[F] | Active | 25 |
| **ML- 49** | N#CC=1C([H])=C([H])C(=C([H])C=1[H])C=3C([H])=C(OC([H])([H])C2([H])C([H])([H])C([H])([H])N([H])C([H])([H])C2([H])[H])C([H])=NC=3C=4C([H])=C([H])[S]C=4[H] | Active | 25 |
| **ML- 50** | N#CC=4C([H])=C([H])C(/C2=C(/N=C(/[H])C(OC([H])([H])C1([H])C([H])([H])C([H])([H])N([H])C([H])([H])C1([H])[H])=C2[H])C=3C([H])=C([H])C(=C([H])C=3[H])C([H])(C([H])([H])[H])C([H])([H])[H])=C([H])C=4[H] | Active | 25 |
| **ML- 51** | [F]/C4=C(\C2=C(\N=C(\[H])C(OC([H])([H])C1([H])C([H])([H])C([H])([H])N([H])C([H])([H])C1([H])[H])=C2[H])C=3C([H])=C([H])C(=C([H])C=3[H])C([H])([H])[H])C([H])=C([H])C(C#N)=C4[H] | Active | 25 |
| **ML- 52** | N#CC=4C([H])=C([H])C(/C2=C(/N=C(/[H])C(OC([H])([H])C1([H])C([H])([H])C([H])([H])N([H])C([H])([H])C1([H])[H])=C2[H])C=3C([H])=C([H])C(OC([H])([H])[H])=C([H])C=3[H])=C([H])C=4[H] | Active | 25 |

| Table SM10: ML Training list compounds | | | |
|---|---|---|---|
| **Compound ID** | **SMILES** | **Activity class** | **Source** |
| **ML- 53** | [H]C=2C(=NC1=NC(=NN1C=2N([H])C3=C([H])C([H])=C(C([H])=C3[H])N4C([H])([H])C([H])([H])N(C([H])([H])C4([H])[H])C([H])([H])[H])[S]C([H])([H])C5=NC=6C(N5[H])=C([H])C([H])=C([H])C=6[H])C([H])([H])[H] | Active | 19 |
| **ML- 54** | O=C(C=1C([H])=C([H])C(O[H])=C([H])C=1[H])N([H])N=C([H])C=2C([H])=C([H])C([H])=C([H])C=2O[H] | Active | 23 |
| **ML- 55** | O=C(N([H])N=C(C=1C([H])=C([Cl])C([H])=C([H])C=1O[H])C([H])([H])[H])C=2C([H])=C([H])C([H])=C([H])C=2[H] | Active | 23 |
| **ML- 56** | O=C(C=1C([H])=C([H])C(O[H])=C([H])C=1[H])N([H])N=C([H])C=2C([H])=C([Cl])C([H])=C([H])C=2O[H] | Active | 23 |
| **ML- 57** | O=C(N([H])N=C(C=1C([H])=C([H])C([H])=C([H])C=1O[H])C([H])([H])[H])C=2C([H])=C([H])C(O[H])=C([H])C=2[H] | Active | 23 |
| **ML- 58** | O=C(N([H])N=C([H])C1=C(O[H])C([H])=C([H])C([H])=C1[H])C=2C([H])=C([H])C([Br])=C([H])C=2[H] | Active | 23 |
| **ML- 59** | O=C(N([H])N=C([H])C1=C(O[H])C([H])=C([H])C([H])=C1[H])C=2C([H])=C([H])C([H])=C([Cl])C=2[H] | Active | 23 |
| **ML- 60** | O=C(N([H])N=C(C=1C([H])=C([H])C([H])=C([H])C=1O[H])C([H])([H])[H])C=2C([H])=C([H])C([H])=C(C=2[H])[S](=O)(=O)N3C([H])([H])C([H])([H])OC([H])([H])C3([H])[H] | Active | 23 |
| **ML- 61** | O=C(N([H])N=C(C=1C([H])=C([Cl])C([H])=C([H])C=1O[H])C([H])([H])[H])C=2C([H])=NC([Cl])=C([Br])C=2[H] | Active | 23 |
| **ML- 62** | O=C(N([H])N=C(C=1C(O[H])=C([H])C([H])=C([H])C=1O[H])C([H])([H])[H])C=2C([H])=C([H])C([H])=C([H])C=2[H] | Active | 23 |
| **ML- 63** | O=C(N([H])N=C(C=1C([H])=C([Cl])C([H])=C([H])C=1O[H])C([H])([H])[H])C=2C([H])=NC([H])=C([Cl])C=2[H] | Active | 23 |
| **ML- 64** | O=C(N([H])N=C(C=1C([H])=C([H])N=C([Cl])C=1[H])C([H])([H])[H])C=2C([H])=C([H])C([H])=C(C=2[H])[S](=O)(=O)N3C([H])([H])C([H])([H])OC([H])([H])C3([H])[H] | Active | 23 |
| **ML- 65** | O=C(/C1=C(\[H])C([H])=C([H])C(N(C([H])([H])C([H])([H])[H])C([H])([H])C([H])([H])[H])=C1[H])N([H])N=C(C=2C([H])=C([H])C([H])=C(C=2[H])C([H])([H])[H])C([H])([H])[H] | Active | 23 |
| **ML- 66** | O=C(N([H])N=C(C=1C([H])=C([Cl])C([H])=C([H])C=1O[H])C([H])([H])[H])C=2C([H])=C([H])C([Cl])=C([Br])C=2[H] | Active | 23 |
| **ML- 67** | O=C(N([H])N=C(C=1C([H])=C([H])C([H])=NC=1[H])C([H])([H])[H])C=2C([H])=C([H])C([H])=C(C=2[H])[S](=O)(=O)N3C([H])([H])C([H])([H])OC([H])([H])C3([H])[H] | Active | 23 |

**Table SM10**: ML Training list compounds

| Compound ID | SMILES | Activity class | Source |
|---|---|---|---|
| **ML- 68** | O=C(N([H])N=C(C=1C([H])=C([H])C([Cl])=C([H])C=1O[H])C([H])([H])[H])C=2C([H])=C([H])C([H])=C(C=2[H])[S](=O)(=O)N3C([H])([H])C([H])([H])OC([H])([H])C3([H])[H] | Active | 30 |
| **ML- 69** | [H]C=2C(=NC1=NC(=NN1C=2N([H])N([H])[H])[S]C([H])([H])C3=C([H])C([H])=C([H])C([H])=C3[H])C([H])([H])[H] | Active | 31 |
| **ML- 70** | N#C/C4=C(\[H])C([H])=C(/C1=N/C(=C(N1/C2=C(\[H])C([H])=C(C([H])=C2[H])C([H])([H])[H])C([H])([H])[H])C(=O)N3C([H])([H])C([H])([H])C([H])([H])[C@@]([H])(N([H])[H])C3([H])[H])C([H])=C4[H] | Active | 35 |
| **ML- 71** | O=C(N([H])C2=C([H])C([H])=C([H])C(OC([H])([H])C1([H])C([H])([H])C([H])([H])N(C([H])([H])C1([H])[H])C([H])([H])[H])=C2[H])C=4N(C3=C([S]C([H])=C3[H])C=4[H])C([H])([H])[H] | Active | 36 |
| **ML- 72** | N#C/C4=C(\[F])C([H])=C(/C1=N/C(=C(N1/C2=C(\[H])C([H])=C(OC([H])([H])[H])C([F])=C2[H])C([H])([H])[H])C(=O)N([H])[C@]3([H])C([H])([H])C([H])([H])C([H])([H])N([H])C3([H])[H])C([H])=C4[H] | Active | 35 |
| **ML- 73** | O=C(N([H])C1=C([H])C([H])=C([H])C(=C1[H])C([H])([H])O/C3=C(\[H])C([H])=C(OC2([H])C([H])([H])C([H])([H])N([H])C([H])([H])C2([H])[H])C([H])=C3[H])C=5N(C4=C([S]C([H])=C4[H])C=5[H])C([H])([H])[H] | Active | 36 |
| **ML- 74** | N#C/C4=C(\[F])C([H])=C(/C1=N/C(=C(N1/C2=C(\[H])C([H])=C(OC([H])([H])[H])C([F])=C2[H])C([H])([H])[H])C(=O)N([H])[C@@]3([H])C([H])([H])C([H])([H])N([H])C3([H])[H])C([H])=C4[H] | Active | 35 |
| **ML- 75** | O=C(/C2=C(\[H])C=1[S]C([H])=C([H])C=1N2C([H])([H])[H])N([H])C3=C(C([H])=C([H])C([H])=C3[H])C([H])([H])O/C5=C(\[H])C([H])=C(OC4([H])C([H])([H])C([H])([H])N(C([H])([H])C4([H])[H])C([H])([H])[H])C([H])=C5[H] | Active | 24 |
| **ML- 76** | O=C(/C2=C(\[H])C=1[S]C([H])=C([H])C=1N2C([H])([H])[H])N([H])C3=C(C([H])=C([H])C([H])=C3[H])C([H])([H])C([H])([H])C5=C([H])C([H])=C(OC4([H])C([H])([H])C([H])([H])N([H])C([H])([H])C4([H])[H])C([H])=C5[H] | Active | 24 |
| **ML- 77** | O=C(/C2=C(\[H])C=1[S]C([H])=C([H])C=1N2C([H])([H])[H])N([H])C3=C(C([H])=C([H])C([H])=C3[H])C([H])([H])O/C4=C(\[H])C([H])=C(C([H])=C4[H])N5C([H])([H])C([H])([H])N([H])C([H])([H])C5([H])[H] | Active | 24 |

**Table SM10**: ML Training list compounds

| Compound ID | SMILES | Activity class | Source |
|---|---|---|---|
| **ML- 78** | O=C(/C2=C(\[H])C=1[S]C([H])=C([H])C=1N2C([H])([H])[H])N([H])C3=C(C([H])=C([H])C([H])=C3[H])C(=O)N([H])C5=C([H])C([H])=C(OC4([H])C([H])([H])C([H])([H])N([H])C([H])([H])C4([H])[H])C([H])=C5[H] | Active | 24 |
| **ML- 79** | O=C(/C2=C(\[H])C=1[S]C([H])=C([H])C=1N2C([H])([H])[H])N([H])C3=C(C([H])=C([H])C([H])=C3[H])C([H])([H])O/C4=C(\[H])C([H])=C(C([H])=C4[H])N([H])C5([H])C([H])([H])C([H])([H])N([H])C([H])([H])C5([H])[H] | Active | 24 |
| **ML- 80** | O=C(/C2=C(\[H])C=1[S]C([H])=C([H])C=1N2C([H])([H])[H])N([H])C3=C(C([H])=C([H])C([H])=C3[H])C([H])([H])O/C5=C(\[H])C([H])=C(OC([H])([H])C4([H])C([H])([H])N([H])C4([H])[H])C([H])=C5[H] | Active | 24 |
| **ML- 81** | O=C(/C2=C(\[H])C=1[S]C([H])=C([H])C=1N2C([H])([H])[H])N([H])C3=C(C([H])=C([H])C([H])=C3[H])C([H])([H])O/C4=C(\[H])C([H])=C(OC([H])([H])N([H])C(=N[H])N([H])[H])C([H])=C4[H] | Active | 24 |
| **ML- 82** | N#C/C4=C(\[F])C([H])=C(/C1=N/C(=C(N1/C2=C(\[H])C([H])=C(OC([H])([H])[H])C([F])=C2[H])C([H])([H])[H])C(=O)N([H])C3([H])C([H])([H])C([H])([H])N([H])C([H])([H])C3([H])[H])C([H])=C4[H] | Active | 35 |
| **ML- 83** | O=C(/C2=C(\[H])C=1[S]C([H])=C([H])C=1N2C([H])([H])[H])N([H])C3=C(C([H])=C([H])C([H])=C3[H])C([H])([H])O/C5=C(\[H])C([H])=C(OC([H])([H])[C@@]4([H])C([H])([H])N([H])C([H])([H])C4([H])[H])C([H])=C5[H] | Active | 24 |
| **ML- 84** | O=C(/C2=C(\[H])C=1[S]C([H])=C([H])C=1N2C([H])([H])[H])N([H])C3=C(C([H])=C([H])C([H])=C3[H])C([H])([H])O/C5=C(\[H])C([H])=C(O[C@@]4([H])C([H])([H])C([H])([H])C([H])([H])N([H])C([H])([H])C4([H])[H])C([H])=C5[H] | Active | 24 |
| **ML- 85** | O=C(/C2=C(\[H])C=1[S]C([H])=C([H])C=1N2C([H])([H])[H])N([H])C3=C(C([H])=C([H])C([H])=C3[H])C([H])([H])O/C5=C(\[H])C([H])=C(O[C@@]4([H])C([H])([H])C([H])([H])[C@]([H])(N([H])[H])C([H])([H])C4([H])[H])C([H])=C5[H] | Active | 24 |
| **ML- 86** | O=C(/C2=C(\[H])C=1[S]C([H])=C([H])C=1N2C([H])([H])[H])N([H])C3=C(C([H])=C([H])C([H])=C3[H])C([H])([H])O/C5=C(\[H])C([H])=C(OC([H])([H])[C@]4([H])C([H])([H])N([H])C([H])([H])C4([H])[H])C([H])=C5[H] | Active | 24 |

**Table SM10**: ML Training list compounds 

| Compound ID | SMILES | Activity class | Source |
|---|---|---|---|
| **ML- 87** | O=C(/C2=C(\[H])C=1[S]C([H])=C([H])C=1N2C([H])([H])[H])N([H])C3=C(C([H])=C([H])C([H])=C3[H])C([H])([H])O/C5=C(\[H])C([H])=C(O[C@@]4([H])C([H])([H])C([H])([H])[C@]([H])(N([H])[H])C([H])([H])C4([H])[H])C([H])=C5[H] | Active | 24 |
| **ML- 88** | O=C(/C2=C(\[H])C=1[S]C([H])=C([H])C=1N2C([H])([H])[H])N([H])C3=C(C([H])=C([H])C([H])=C3[H])C([H])([H])O/C5=C(\[H])C([H])=C(O[C@]4([H])C([H])([H])N([H])C([H])([H])C([H])([H])C4([H])[H])C([H])=C5[H] | Active | 24 |
| **ML- 89** | O=C(/C2=C(\[H])C=1[S]C([H])=C([H])C=1N2C([H])([H])[H])N([H])C3=C(C([H])=C([H])C([H])=C3[H])C([H])([H])O/C5=C(\[H])C([H])=C(O[C@@]4([H])C([H])([H])C([H])([H])N([H])C4([H])[H])C([H])=C5[H] | Active | 24 |
| **ML- 90** | N#C/C4=C(\[F])C([H])=C(/C1=N/C(=C(N1/C2=C(\[H])C([H])=C(OC([H])([H])[H])C([F])=C2[H])C([H])([H])[H])C(=O)N3C([H])([H])C([H])(C3([H])[H])C([H])([H])N([H])[H])C([H])=C4[H] | Active | 35 |
| **ML- 91** | O=C(/C2=C(\[H])C=1[S]C([H])=C([H])C=1N2C([H])([H])[H])N([H])C3=C(C([H])=C([H])C([H])=C3[H])C([H])([H])O/C4=C(\[H])C([H])=C(OC([H])([H])C([H])([H])C([H])([H])N([H])C([H])([H])[H])C([H])=C4[H] | Active | 24 |
| **ML- 92** | O=C(/C2=C(\[H])C=1[S]C([H])=C([H])C=1N2C([H])([H])[H])N([H])C5=C(OC([H])([H])[C@]3([H])C([H])([H])C([H])([H])N(C([H])([H])C3([H])[H])C([H])([H])[C@@]4([H])C([H])([H])C([H])([H])N([H])C([H])([H])C4([H])[H])C([H])=C([H])C([H])=C5[H] | Active | 24 |
| **ML- 93** | O=C(/C2=C(\[H])C=1[S]C([H])=C([H])C=1N2C([H])([H])[H])N([H])C3=C(/C(=C(/[H])C([H])=C3[H])C([H])([H])OC([H])([H])[H])C([H])([H])O/C5=C(\[H])C([H])=C(OC([H])([H])[C@@]4([H])C([H])([H])C([H])([H])N([H])C4([H])[H])C([H])=C5[H] | Active | 24 |
| **ML- 94** | O=C(/C2=C(\[H])C=1[S]C([H])=C([H])C=1N2C([H])([H])[H])N([H])C3=C(/C(=C(/[H])C([H])=C3[H])C([H])([H])OC([H])([H])[H])C([H])([H])O/C5=C(\[H])C([H])=C(O[C@]4([H])C([H])([H])C([H])([H])C([H])([H])N([H])C([H])([H])C4([H])[H])C([H])=C5[H] | Active | 24 |
| **ML- 95** | O=C(/C2=C(\[H])C=1[S]C([H])=C([H])C=1N2C([H])([H])C([H])([H])[H])N([H])C3=C(/C(=C(/[H])C([H])=C3[H])C([H])([H])OC([H])([H])[H])C([H])([H])O/C5=C(\[H])C([H])=C(OC([H])([H])[C@@]4([H])C([H])([H])C([H])([H])N([H])C4([H])[H])C([H])=C5[H] | Active | 24 |

**Table SM10**: ML Training list compounds

| Compound ID | SMILES | Activity class | Source |
|---|---|---|---|
| **ML- 96** | O=C(/C2=C(\[H])C=1[S]C([H])=C([H])C=1N2C([H])([H])[H])N([H])C3=C(/C(=C(/[H])C([H])=C3[H])C([H])([H])OC([H])([H])C([H])([H])[H])C([H])([H])O/C5=C(\[H])C([H])=C(OC([H])([H])[C@@]4([H])C([H])([H])C([H])([H])N([H])C4([H])[H])C([H])=C5[H] | Active | 24 |
| **ML- 97** | O=C(/C2=C(\[H])C=1[S]C([H])=C([H])C=1N2C([H])([H])[H])N([H])C3=C(/C(=C(/[H])C([H])=C3[H])C([H])([H])OC([H])([H])C([H])([H])[H])C([H])([H])O/C5=C(\[H])C([H])=C(OC4([H])C([H])([H])C([H])([H])N([H])C([H])([H])C4([H])[H])C([H])=C5[H] | Active | 24 |
| **ML- 98** | N#C/C5=C(\[F])C([H])=C(/C1=N/C(=C(N1/C3=C(\[H])C=2C(=NN(C=2[H])C([H])([H])[H])C([H])=C3[H])C([H])([H])[H])C(=O)N4C([H])([H])C([H])([H])C([H])([H])[C@@]([H])(N([H])[H])C4([H])[H])C([H])=C5[H] | Active | 35 |
| **ML- 99** | O=C(/C2=C(\[H])C=1[S]C([H])=C([H])C=1N2C([H])([H])[H])N([H])C3=C(/C(=C(/[H])C([H])=C3[H])C([H])([H])N4C([H])([H])C([H])([H])OC([H])([H])C4([H])[H])C([H])([H])O/C6=C(\[H])C([H])=C(OC5([H])C([H])([H])C([H])([H])N([H])C([H])([H])C5([H])[H])C([H])=C6[H] | Active | 24 |
| **ML- 100** | N#C/C5=C(\[F])C([H])=C(/C1=N/C(=C(N1/C2=C(\[H])C3=C(C([H])=C2[H])N(/N=C3/[H])C([H])([H])[H])C([H])([H])[H])C(=O)N4C([H])([H])C([H])([H])C([H])([H])[C@@]([H])(N([H])[H])C4([H])[H])C([H])=C5[H] | Active | 35 |
| **ML- 101** | [H]C=2C(=NC1=NC(=NN1C=2N([H])N([H])[H])[S]C([H])([H])C#C[H])C([H])([H])[H] | Active | 31 |
| **ML- 102** | [H]C=1/C(=C(/O[H])C([H])=C([H])C=1C([H])=C([H])C2=C([H])C([H])=C([H])C(O[H])=C2[H])C=3/N=C(/[H])C([H])=C([H])C=3[H] | Active | 29 |
| **ML- 103** | N#C/C5=C(\[F])C([H])=C(/C1=N/C(=C(N1/C3=C(\[H])C=2C(=NN(C=2[H])C([H])([H])[H])C([H])=C3[H])C([H])([H])[H])C(=O)N4C([H])([H])[C@@]([H])(N([H])[H])C([H])([H])C4([H])[H])C([H])=C5[H] | Active | 35 |
| **ML- 104** | [H]C=1/C(=C(/O[H])C([H])=C([H])C=1C([H])=C([H])C2=C([H])C([H])=C([H])C(=C2[H])C(=NO[H])N([H])[H])C=3/N=C(/[H])C([H])=C([H])C=3[H] | Active | 29 |
| **ML- 105** | N#C/C5=C(\[F])C([H])=C(/C1=N/C(=C(N1/C3=C(\[H])C=2C(=NN(C=2[H])C([H])([H])[H])C([H])=C3[H])C([H])([H])[H])C(=O)N4C([H])([H])[C@@]([H])(N(C([H])([H])[H])C([H])([H])[H])C([H])([H])C4([H])[H])C([H])=C5[H] | Active | 35 |
| **ML- 106** | [H]C=1/C(=C(/O[H])C([H])=C([H])C=1C([H])=C([H])C2=C([H])C([H])=C([H])C(=C2[H])C(=NO[H])N([H])[H])C3=NC([H])=C([H])C([H])=N3 | Active | 29 |

| | | | |
|---|---|---|---|
| **Table SM10**: ML Training list compounds | | | |
| **Compound ID** | **SMILES** | **Activity class** | **Source** |
| **ML- 107** | N#C/C5=C(\[F])C([H])=C(/C1=N/C(=C(N1/C3=C(\[H])C=2C(=NN(C=2[Cl])C([H])([H])[H])C([H])=C3[H])C([H])([H])[H])C(=O)N4C([H])([H])[C@@]([H])(N([H])C([H])([H])[H])C([H])([H])C4([H])[H])C([H])=C5[H] | Active | 35 |
| **ML- 108** | [H]C=1/C(=C(/O[H])C([H])=C([H])C=1C([H])=C([H])C2=C([H])C([H])=C(C([H])=C2[H])C(=NO[H])N([H])[H])C3=NC([H])=C([H])C([H])=N3 | Active | 29 |
| **ML- 109** | N#C/C4=C(\[F])C([H])=C(/C1=N/C(=C(N1/C2=C(\[H])C([H])=C(OC([H])([H])[H])C([H])=C2[H])C([H])([H])[H])C(=O)N3C([H])([H])C([H])([H])C([H])([H])[C@@]([H])(N([H])[H])C3([H])[H])C([H])=C4[H] | Active | 35 |
| **ML- 110** | [H]C=1/C(=C(/O[H])C([H])=C([H])C=1C([H])=C([H])C2=C([H])C([H])=C([H])C(=C2[H])N([H])[H])C=3/N=C(/[H])C([H])=C([H])C=3[H] | Active | 29 |
| **ML- 111** | N#C/C5=C(\[F])C([H])=C(/C1=N/C(=C(N1/C3=C(\[H])C=2C(=NN(C=2[H])C([H])([H])[H])C([H])=C3[F])C([H])([H])[H])C(=O)N4C([H])([H])[C@@]([H])(N([H])C([H])([H])[H])C([H])([H])C4([H])[H])C([H])=C5[H] | Active | 35 |
| **ML- 112** | [H]C=1/C(=C(/O[H])C([H])=C([H])C=1C([H])=C([H])C2=C([H])C([H])=C(C([H])=C2[H])N([H])[H])C=3/N=C(/[H])C([H])=C([H])C=3[H] | Active | 29 |
| **ML- 113** | N#C/C5=C(\[F])C([H])=C(/C1=N/C(=C(N1/C3=C(\[H])C=2C(=NN(C=2[H])C([H])([H])[H])C([H])=C3[F])C([H])([H])[H])C(=O)N4C([H])([H])[C@@]([H])(N(C([H])([H])[H])C([H])([H])[H])C([H])([H])C4([H])[H])C([H])=C5[H] | Active | 35 |
| **ML- 114** | [H]C=1/C(=C(/O[H])C([H])=C([H])C=1C([H])=C([H])C2=C([H])C([H])=C([H])C(=C2[H])N([H])[H])C3=NC([H])=C([H])C([H])=N3 | Active | 29 |
| **ML- 115** | N#C/C5=C(\[F])C([H])=C(/C1=N/C(=C(N1/C3=C(\[H])C=2C(=NN(C=2[H])C([H])([H])[H])C([F])=C3[H])C([H])([H])[H])C(=O)N4C([H])([H])[C@@]([H])(N(C([H])([H])[H])C([H])([H])[H])C([H])([H])C4([H])[H])C([H])=C5[H] | Active | 35 |
| **ML- 116** | N#C/C5=C(\[F])C([H])=C(/C1=N/C(=C(N1/C3=C(\[H])C=2C(=NN(C=2[Cl])C([H])([H])[H])C([H])=C3[H])C([H])([H])[H])C(=O)N4C([H])([H])C([H])([H])C([H])([H])[C@@]([H])(N([H])[H])C4([H])[H])C([H])=C5[H] | Active | 35 |
| **ML- 117** | [H]C=1/C(=C(/O[H])C([H])=C([H])C=1C([H])=C([H])C2=C([H])C([H])=C([H])C(=C2[H])N([H])[H])C=3/N=C(/[H])C([H])=C(O[H])C=3[H] | Active | 29 |

| **Table SM10**: ML Training list compounds | | | |
|---|---|---|---|
| **Compound ID** | **SMILES** | **Activity class** | **Source** |
| **ML- 118** | N#C/C5=C(\[F])C([H])=C(/C1=N/C(=C(N1/C3=C(\[H])C=2C(=NN(C=2[Cl])C([H])([H])[H])C([H])=C3[H])C([H])([H])[H])C(=O)N4C([H])([H])C([H])([H])C([H])([H])[C@@]([H])(N([H])C([H])([H])[H])C4([H])[H])C([H])=C5[H] | Active | 35 |
| **ML- 119** | [H]C=1C(=NC([H])=C([H])C=1C([H])=C([H])C2=C([H])C([H])=C([H])C(=C2[H])C(=NO[H])N([H])[H])C3=C(O[H])C([H])=C([H])C([H])=C3[H] | Active | 29 |
| **ML- 120** | [H]C=1C(=NC([H])=C([H])C=1C([H])=C([H])C2=C([H])C([H])=C(C([H])=C2[H])C(=NO[H])N([H])[H])C3=C([H])C([H])=C([H])C([H])=C3O[H] | Active | 29 |
| **ML- 121** | N#C/C5=C(\[F])C([H])=C(/C1=N/C(=C(N1/C2=C(\[H])C([H])=C(/N=C2/[H])[C@@]3([H])C([H])([H])C3([H])[H])C([H])([H])[H])C(=O)N4C([H])([H])C([H])([H])C([H])([H])[C@@]([H])(N([H])[H])C4([H])[H])C([H])=C5[H] | Active | 35 |
| **ML- 122** | [F]C=3C([H])=C([H])C(O[H])=C(C=1/N=C(/[H])C([H])=C(C=1[H])C([H])=C([H])C2=C([H])C(=C([H])C([H])=C2[H])C(=NO[H])N([H])[H])C=3[H] | Active | 29 |
| **ML- 123** | N#C/C4=C(\[F])C([H])=C(/C1=N/C(=C(N1/C2=C(\[H])C([H])=C(OC([H])([H])[H])C([F])=C2[H])C([H])([H])[H])C(=O)N3C([H])([H])C([H])([H])C([H])([H])[C@@]([H])(N([H])[H])C3([H])[H])C([H])=C4[H] | Active | 35 |
| **ML- 124** | [H]C=1C(=NC([H])=C([H])C=1C([H])=C([H])C2=C([H])C([H])=C(C([H])=C2[H])N([H])[H])C3=C([H])C([H])=C([H])C([H])=C3O[H] | Active | 29 |
| **ML- 125** | N#C/C4=C(\[F])C([H])=C(/C1=N/C(=C(N1/C2=C(\[H])C([H])=C(OC([H])([H])[H])C([F])=C2[H])C([H])([H])[H])C(=O)N3C([H])([H])C([H])([H])[C@@]([H])(N([H])[H])C3([H])[H])C([H])=C4[H] | Active | 35 |
| **ML- 126** | [S]=C(N([H])[H])N([H])N([H])C=2/N=C(/[S]C([H])([H])C#C[H])N=C(C=1C([H])=C([H])C([H])=C([H])C=1[H])C=2C#N | Active | 18 |
| **ML- 127** | N#C/C4=C(\[F])C([H])=C(/C1=N/C(=C(N1/C2=C(\[H])C([H])=C(OC([H])([H])[H])C([F])=C2[H])C([H])([H])[H])C(=O)N([H])[C@@]3([H])C([H])([H])C([H])([H])C([H])([H])N([H])C3([H])[H])C([H])=C4[H] | Active | 35 |
| **ML- 128** | [S]=C(N([H])N([H])C2=NC(=NC(/C1=C(\[H])C(OC([H])([H])[H])=C(OC([H])([H])[H])C(OC([H])([H])[H])=C1[H])=C2C#N)[S]C([H])([H])C#C[H])N([H])[H] | Active | 18 |
| **ML- 129** | [S]=C(N([H])N([H])C2=NC(=NC(C=1C([H])=C([H])C(=C([H])C=1[H])C([H])([H])[H])=C2C#N)[S]C([H])([H])C#C[H])N([H])[H] | Active | 18 |

**Table SM10**: ML Training list compounds 

| Compound ID | SMILES | Activity class | Source |
|---|---|---|---|
| **ML- 130** | [S]=C(N([H])N([H])C2=NC(=NC(C=1C([H])=C([H])C(=C([H])C=1[H])C([H])([H])C([H])(C([H])([H])[H])C([H])([H])[H])=C2C#N)[S]C([H])([H])C#C[H])N([H])[H] | Active | 18 |
| **ML- 131** | [S]=C(N([H])[H])N([H])N([H])C=2/N=C(/[S]C([H])([H])C#C[H])N=C(C=1C([H])=C([H])C([F])=C([H])C=1[H])C=2C#N | Active | 18 |
| **ML- 132** | [S]=C(N([H])[H])N([H])N([H])C=2/N=C(/[S]C([H])([H])C#C[H])N=C(C=1C([H])=C([H])C(=C([H])C=1[H])[N+](=O)[O-])C=2C#N | Active | 18 |
| **ML- 133** | [S]=C(N([H])[H])N([H])N([H])C=2/N=C(/[S]C([H])([H])C#C[H])N=C(C=1C([H])=C([H])C([Cl])=C([H])C=1[H])C=2C#N | Active | 18 |
| **ML- 134** | [S]=C(N([H])[H])N([H])N([H])C=2/N=C(/[S]C([H])([H])C#C[H])N=C(C=1C([H])=C([H])C([F])=C([F])C=1[H])C=2C#N | Active | 18 |
| **ML- 135** | [S]=C(N([H])[H])N([H])N([H])C=2/N=C(/[S]C([H])([H])C#C[H])N=C(C=1C([H])=C([H])C([Br])=C([H])C=1[H])C=2C#N | Active | 18 |
| **ML- 136** | [S]=C(N([H])[H])N([H])N([H])C=2/N=C(/[S]C([H])([H])C#C[H])N=C(C=1C([H])=C([H])C([H])=C([Cl])C=1[H])C=2C#N | Active | 18 |
| **ML- 137** | [S]=C(N([H])N([H])C2=NC(=NC(/C1=C(\[H])C([H])=C([H])C(OC([H])([H])[H])=C1[H])=C2C#N)[S]C([H])([H])C#C[H])N([H])[H] | Active | 18 |
| **ML- 138** | O=C(N([H])[H])N([H])N([H])C=2/N=C(/[S]C([H])([H])C#C[H])N=C(C=1C([H])=C([H])C([Cl])=C([H])C=1[H])C=2C#N | Active | 18 |
| **ML- 139** | O=C1N(C(=[S])N([H])C(=O)C1=C([H])C2=C([H])C([H])=C(O[H])C(O[H])=C2[H])C3=C([H])C([H])=C([Br])C([H])=C3[H] | Active | 26 |
| **ML- 140** | O=C1N(C(=O)N([H])C(=O)C1=C([H])C=2N([H])C=3C(C=2[H])=C([H])C([H])=C([H])C=3[H])C4=C([H])C([H])=C(C([H])=C4[H])C([H])([H])[H] | Active | 26 |
| **ML- 141** | O=C(N([H])N([H])C2=NC(=NC(C=1C([H])=C([H])C(=C([H])C=1[H])C([H])([H])[H])=C2C#N)[S]C([H])([H])C#C[H])N([H])[H] | Active | 18 |
| **ML- 142** | O=C1N(C(=O)N([H])C(=O)C1=C([H])C=2N([H])C=3C(C=2[H])=C([H])C([H])=C([H])C=3[H])C4=C(C([H])=C([H])C([H])=C4[H])C([H])([H])[H] | Active | 26 |
| **ML- 143** | O=C(N([H])N([H])C2=NC(=NC(C=1C([H])=C([H])C(=C([H])C=1[H])C([H])(C([H])([H])[H])C([H])([H])[H])=C2C#N)[S]C([H])([H])C#C[H])N([H])[H] | Active | 18 |

| Table SM10: ML Training list compounds | | | |
|---|---|---|---|
| **Compound ID** | **SMILES** | **Activity class** | **Source** |
| **ML- 144** | O=C1N(C(=O)N([H])C(=O)C1=C([H])C2=C([H])C([H])=C(O[H])C(O[H])=C2[H])C3=C([H])C([H])=C([Br])C([H])=C3[H] | Active | 26 |
| **ML- 145** | O=C(N([H])[H])N([H])N([H])C=2/N=C(/[S]C([H])([H])C#C[H])N=C(C=1C([H])=C([H])C([H])=C([Cl])C=1[H])C=2C#N | Active | 18 |
| **ML- 146** | O=C1N(C(=O)N([H])C(=O)C1=C([H])C2=C([H])C([H])=C(O[H])C(O[H])=C2[H])C3=C([H])C([H])=C(OC([H])([H])[H])C([H])=C3[H] | Active | 26 |
| **ML- 147** | O=C1N(C(=O)N([H])C(=O)C1=C([H])C2=C([H])C([H])=C(OC([H])([H])[H])C(O[H])=C2[H])C3=C([H])C([H])=C([Br])C([H])=C3[H] | Active | 26 |
| **ML- 148** | O=C1N(C(=O)N([H])C(=O)C1=C([H])C2=C([H])C([H])=C(OC([H])([H])[H])C(OC([H])([H])[H])=C2OC([H])([H])[H])C3=C([H])C([H])=C([Br])C([H])=C3[H] | Active | 26 |
| **ML- 149** | O=C1N(C(=O)N([H])C(=O)C1=C([H])C2=C([H])C([H])=C(OC([H])([H])[H])C(OC([H])([H])[H])=C2[H])C3=C([H])C([H])=C([Br])C([H])=C3[H] | Active | 26 |
| **ML- 150** | [Cl]/C2=C(\[H])C([H])=C1[S]C([H])=C(/C1=C2\[H])C([H])([H])[S]C=4N=C3N=C(C([H])=C(N3N=4)N([H])N([H])[H])C([H])([H])[H] | Active | 31 |
| **ML- 151** | [Cl]/C1=C(\[H])C([H])=C(C([H])=C1[H])C([H])([H])[S]C=3N=C2N=C(C([H])=C(N2N=3)N([H])N([H])[H])C([H])([H])[H] | Active | 31 |
| **CHEMBL1198896** | [H]N(/C(=N\C([H])([H])[H])N([H])C([H])([H])C([H])([H])C([H])([H])N([H])C([H])([H])C([H])([H])C([H])([H])N([H])C([H])([H])C([H])([H])C([H])([H])N([H])C(=NC([H])([H])[H])N([H])C([H])([H])[H])C([H])([H])[H] | Active | ChEMBL database |
| **CHEMBL2182004** | O=C(N([H])C=1C([H])=C([H])C([H])=C([H])C=1OC=2C([H])=C([H])C([H])=C([H])C=2[H])C([H])([H])C(=NO[H])N([H])[H] | Active | ChEMBL database |
| **CHEMBL3104344** | [H]N(C([H])([H])C([H])([H])[H])C([H])([H])C([H])([H])C([H])([H])N([H])C([H])([H])C([H])=C([H])C([H])([H])N([H])C([H])([H])C([H])([H])C([H])([H])N([H])C([H])([H])C([H])([H])[H] | Active | ChEMBL database |
| **CHEMBL3621615** | [Cl]C=2/C(=C(/OC=1C([H])=C([H])C([H])=C([H])C=1[H])C([H])=C([H])C=2[H])C([H])([H])N([H])C3=N/N=C(/N([H])[H])N3[H] | Active | ChEMBL database |
| **CHEMBL3682529** | O=C(N([H])N=C(C=1C([H])=C([Cl])C([H])=C([H])C=1O[H])C([H])([H])[H])C=2C([H])=C([H])C([Cl])=C([Br])C=2[H] | Active | ChEMBL database |

| **Table SM10**: ML Training list compounds | | | |
|---|---|---|---|
| **Compound ID** | **SMILES** | **Activity class** | **Source** |
| **CHEMBL3682531** | O=C(N([H])N=C(C=1C([H])=C([Cl])C([H])=C([H])C=1O[H])C([H])([H])[H])C=2C([H])=NC([Cl])=C([Br])C=2[H] | Active | ChEMBL database |
| **CHEMBL3682532** | O=C(N([H])N=C(C=1C([H])=C([Cl])C([H])=C([H])C=1O[H])C([H])([H])[H])C=2C([H])=NC([H])=C([Cl])C=2[H] | Active | ChEMBL database |
| **CHEMBL3682535** | O=C(N([H])N=C(C=1C([H])=C([Cl])C([H])=C([H])C=1O[H])C([H])([H])[H])C=2C([H])=C([H])C([H])=C(C=2[H])N3C([H])([H])C([H])([H])OC([H])([H])C3([H])[H] | Active | ChEMBL database |
| **CHEMBL3785141** | O=C(N([H])C=1C([H])=C([H])C(=C([H])C=1[H])C([H])([H])C([H])([H])N([H])N([H])[H])C([H])([H])C([H])([H])C([H])([H])C=2C([H])=C([H])C([H])=C([H])C=2[H] | Active | ChEMBL database |
| **CHEMBL4060903** | [H]N([H])C(=NO[H])C=2C([H])=C([H])C([H])=C(C([H])=C([H])C=1C([H])=C([H])C(O[H])=C([H])C=1[H])C=2[H] | Active | ChEMBL database |
| **CHEMBL4062570** | [H]N([H])C(=NO[H])C=2C([H])=C([H])C([H])=C(C([H])=C([H])C=1C([H])=C([H])N=C([H])C=1[H])C=2[H] | Active | ChEMBL database |
| **CHEMBL4068480** | [H]C([H])([H])C([H])([H])C([H])([H])[S]C=3/N=C(/[S]/C1=N/C([H])=C([H])C([H])=C1[H])C=2N=NN(C=2N=3)C([H])([H])C=4OC([H])=C([H])C=4[H] | Active | ChEMBL database |
| **CHEMBL4069511** | [H]C#CC([H])([H])[S]C=3/N=C(/[S]/C1=N/C([H])=C([H])C([H])=C1[H])C=2N=NN(C=2N=3)C([H])([H])C=4C([H])=C([H])C([H])=C([H])C=4[H] | Active | ChEMBL database |
| **CHEMBL4069570** | [H]N([H])C(=NO[H])C=2C([H])=C([H])C(C([H])=C([H])C=1C([H])=C([H])C(O[H])=C(O[H])C=1[H])=C([H])C=2[H] | Active | ChEMBL database |
| **CHEMBL4070601** | [H]N([H])C(=NO[H])C=2C([H])=C([H])C([H])=C(C([H])=C([H])C=1C([H])=C([H])C(O[H])=C(O[H])C=1[H])C=2[H] | Active | ChEMBL database |
| **CHEMBL4072238** | [Br]/C2=C(\C([H])=C([H])C=1C([H])=C([H])C(/C(=N/O[H])N([H])[H])=C([H])C=1[H])C([H])=C(O[H])C(O[H])=C2[H] | Active | ChEMBL database |
| **CHEMBL4073481** | O=[S](=O)(N([H])C1=C([H])C(OC([H])([H])[H])=C(C([H])=C1[H])N2N=C(C([H])=C2C([H])([H])[H])C([H])([H])[H])C=4C([H])=C([H])C=3N(C(=O)C([H])([H])[H])C([H])([H])C([H])([H])C=3C=4[H] | Active | ChEMBL database |
| **CHEMBL4079376** | [S]=C(N([H])N([H])C2=NC(=NC(C=1C([H])=C([H])C(=C([H])C=1[H])C([H])(C([H])([H])[H])C([H])([H])[H])=C2C#N)[S]C([H])([H])C#C[H])N([H])[H] | Active | ChEMBL database |
| **CHEMBL4079847** | [H][C@@]3(C=1C([H])=C([H])C(=C([H])C=1[H])C([H])([H])[H])C([H])([H])N(C(=O)[C@]2([H])C([H])([H])C([H])([H])C([H])([H])[C@@]([H])(N([H])[H])C2([H])[H])C([H])([H])[C@@]3([H])C=4C([H])=C([H])C(C#N)=C([H])C=4[H] | Active | ChEMBL database |

**Table SM10**: ML Training list compounds

| Compound ID | SMILES | Activity class | Source |
|---|---|---|---|
| **CHEMBL4080291** | [H]N([H])C(=NO[H])C=2C([H])=C([H])C(C([H])=C([H])C=1C([H])=C([H])C(O[H])=C([H])C=1[H])=C([H])C=2[H] | Active | ChEMBL database |
| **CHEMBL4081219** | [H]N([H])C(=NO[H])C=2C([H])=C([H])C([H])=C(C([H])=C([H])C=1C([H])=C(O[H])C([H])=C(O[H])C=1[H])C=2[H] | Active | ChEMBL database |
| **CHEMBL4084845** | [H][C@]4(C([H])([H])OC=2C([H])=NC(C=1C([H])=C([H])C(=C([H])C=1[H])C([H])([H])[H])=C(C=2[H])C=3C([H])=C([H])C(C#N)=C([H])C=3[H])C([H])([H])C([H])([H])N([H])C4([H])[H] | Active | ChEMBL database |
| **CHEMBL4087193** | [H]C([H])([H])C([H])([H])C([H])([H])[S]C=3/N=C(/[S]/C1=N/C([H])=C([H])C([H])=C1[H])C=2N=NN(C=2N=3)C([H])([H])C=4C([H])=C([H])C([H])=C([H])C=4[H] | Active | ChEMBL database |
| **CHEMBL4087724** | [H][C@@]3(C=1C([H])=C([H])C(=C([H])C=1[H])C([H])([H])[H])C([H])([H])N(C(=O)C([H])([H])[C@@]2([H])C([H])([H])C([H])([H])C([H])([H])N([H])C2([H])[H])C([H])([H])[C@@]3([H])C=4C([H])=C([H])C(C#N)=C([H])C=4[H] | Active | ChEMBL database |
| **CHEMBL4089088** | [F]/C2=C(\C([H])=C([H])C1=C([H])C([H])=C([H])C(/C(=N/O[H])N([H])[H])=C1[H])C([H])=C(O[H])C(O[H])=C2[H] | Active | ChEMBL database |
| **CHEMBL4090528** | [H]C([H])([S]C=3/N=C(/[S]/C1=N/C([H])=C([H])C([H])=C1[H])C=2N=NN(C=2N=3)C([H])([H])C=4C([H])=C([H])C([H])=C([H])C=4[H])C=5C([H])=C([H])C([H])=C([H])C=5[H] | Active | ChEMBL database |
| **CHEMBL4091505** | [F]/C2=C(\O[H])C([H])=C([H])C(C([H])=C([H])C1=C([H])C([H])=C([H])C(/C(=N/O[H])N([H])[H])=C1[H])=C2[H] | Active | ChEMBL database |
| **CHEMBL4093001** | [F]/C2=C(\C([H])=C([H])C=1C([H])=C([H])C(/C(=N/O[H])N([H])[H])=C([H])C=1[H])C([H])=C(O[H])C(O[H])=C2[H] | Active | ChEMBL database |
| **CHEMBL4098092** | [S]=C(N([H])N([H])[C@@]3([H])N2/N=C(/[S]C([H])([H])C=1C([H])=C([H])C([H])=C([H])C=1[H])N=C2N([H])[C@@]([H])(C([H])([H])[H])C3([H])[H])N([H])[H] | Active | ChEMBL database |
| **CHEMBL4099241** | [Br]/C2=C(\C([H])=C([H])C1=C([H])C([H])=C([H])C(/C(=N/O[H])N([H])[H])=C1[H])C([H])=C(O[H])C(O[H])=C2[H] | Active | ChEMBL database |
| **CHEMBL4104513** | [F]/C2=C(\O[H])C([H])=C([H])C(C([H])=C([H])C=1C([H])=C([H])C(/C(=N/O[H])N([H])[H])=C([H])C=1[H])=C2[H] | Active | ChEMBL database |
| **ML-152** | O=C2OC=1C([H])=C(O[H])C([H])=C(C=1/C(=C2/[H])C([H])([H])N3N=N/C(=C3/[H])C([H])([H])[S]C(=[S])N4C([H])([H])C([H])([H])N(C(=O)OC(C([H])([H])[H])(C([H])([H])[H])C([H])([H])[H])C([H])([H])C4([H])[H])C([H])([H])[H] | Inactive | 27 |
| **ML-153** | [S]=C([S]C([H])([H])C=1N=NN(C=1[H])C([H])([H])C=2C([H])=C([H])C(=C([H])C=2[H])C([H])([H])[H])N3C([H])([H])C([H])([H])C([H])([H])C([H])([H])C3([H])[H] | Inactive | 28 |

| Table SM10: ML Training list compounds | | | |
|---|---|---|---|
| **Compound ID** | **SMILES** | **Activity class** | **Source** |
| **ML-154** | [S]=C([S]C([H])([H])C=1N=NN(C=1[H])C([H])([H])C=2C([H])=C([H])C(=C([H])C=2[H])C([H])([H])[H])N3C([H])([H])C([H])([H])OC([H])([H])C3([H])[H] | Inactive | 28 |
| **ML-155** | [S]=C([S]C([H])([H])C=1N=NN(C=1[H])C([H])([H])C=2C([H])=C([H])C([H])=C([H])C=2[H])N3C([H])([H])C([H])([H])C([H])([H])C([H])([H])C3([H])[H] | Inactive | 28 |
| **ML-156** | [Cl]C=1C([H])=C([H])C([H])=C([H])C=1C([H])([H])N2N=N/C(=C2/[H])C([H])([H])[S]C(=[S])N3C([H])([H])C([H])([H])N([H])C([H])([H])C3([H])[H] | Inactive | 28 |
| **ML-157** | [Cl]C=1C([H])=C([H])C(=C([H])C=1[H])C([H])([H])N2N=N/C(=C2/[H])C([H])([H])[S]C(=[S])N3C([H])([H])C([H])([H])N([H])C([H])([H])C3([H])[H] | Inactive | 28 |
| **ML-158** | O=C(OC(C([H])([H])[H])(C([H])([H])[H])C([H])([H])[H])N4C([H])([H])C([H])([H])N(C(=[S])[S]C([H])([H])C=1N=NN(C=1[H])C([H])([H])C=3C([H])=C([H])C2=C([H])C([H])=C([H])C([H])=C2C=3[H])C([H])([H])C4([H])[H] | Inactive | 28 |
| **ML-159** | [S]=C([S]C([H])([H])C=1N=NN(C=1[H])C([H])([H])C=2C([H])=C([H])C([H])=C([H])C=2[H])N3C([H])([H])C([H])([H])OC([H])([H])C3([H])[H] | Inactive | 28 |
| **ML-160** | [S]=C([S]C([H])([H])C=1N=NN(C=1[H])C([H])([H])C=2C([H])=C([H])C(=C([H])C=2[H])C([H])([H])[H])N4C([H])([H])C([H])([H])N(C([H])([H])C=3C([H])=C([H])C([H])=C([H])C=3[H])C([H])([H])C4([H])[H] | Inactive | 28 |
| **ML-161** | O=C(OC(C([H])([H])[H])(C([H])([H])[H])C([H])([H])[H])[C@@]([H])(N([H])C(=O)C=1C([H])=C([H])C([H])=C([H])C=1[H])C([H])([H])C([H])([H])C([H])([H])C([H])([H])N([H])C([H])([H])C#C[H] | Inactive | 22 |
| **ML-162** | [H][C@@](N([H])C(=O)C=1C([H])=C([H])C([H])=C([H])C=1[H])(C(=O)OC(C([H])([H])[H])(C([H])([H])[H])C([H])([H])[H])C([H])([H])C([H])([H])C([H])([H])C([H])([H])N([H])C([H])([H])C#CC([H])([H])[H] | Inactive | 22 |
| **ML-163** | O=C(N([H])C=1C([H])=C([H])C([H])=C(OC([H])([H])C([H])([H])C([H])([H])N([H])C([H])([H])C#C[H])C=1[H])C=2C([H])=C([H])C([H])=C([H])C=2[H] | Inactive | 22 |
| **ML-164** | O=C(C=1N=C([S]C=1[H])N([H])C=2C([H])=C([H])C([H])=C([H])C=2[Cl])C([H])([H])[H] | Inactive | 30 |
| **165** | O=C(C=1N=C([S]C=1[H])N([H])[S](=O)(=O)C=2C([H])=C([H])C([H])=C([H])C=2[H])C([H])([H])[H] | Inactive | 30 |
| **ML-166** | [Cl]C=3C([H])=C([H])C(C=1N=C([S]C=1[H])N(C([H])([H])[H])[S](=O)(=O)C=2C([H])=C([H])C(=C([H])C=2[H])C([H])([H])[H])=C([H])C=3[H] | Inactive | 30 |
| **ML-167** | O=C(C=1N=C([S]C=1[H])N([H])C=2C([H])=C([H])C([H])=C([H])C=2C([H])([H])[H])C([H])([H])[H] | Inactive | 30 |
| **ML-168** | [Cl]C=1C([H])=C([H])C(=C([H])C=1[H])C=2N=C([S]C=2[H])N([H])C(=O)C=3C([H])=C([H])C([H])=C([H])C=3[H] | Inactive | 30 |
| **ML-169** | [Cl]C=1C([H])=C([H])C(=C([H])C=1[H])C=2N=C([S]C=2[H])N([H])[S](=O)(=O)C([H])([H])C=3C([H])=C([H])C([H])=C([H])C=3[H] | Inactive | 30 |

**Table SM10**: ML Training list compounds

| Compound ID | SMILES | Activity class | Source |
|---|---|---|---|
| **ML-170** | [Cl]C=1C([H])=C([H])C(=C([H])C=1[H])C=2N=C([S]C=2[H])N([H])C(=O)N([H])C=3C([H])=C([H])C([H])=C([H])C=3[H] | Inactive | 30 |
| **ML-171** | O=C(C=1N=C([S]C=1[H])N([H])C(=O)N([H])C=2C([H])=C([H])C([H])=C([H])C=2[H])C([H])([H])[H] | Inactive | 30 |
| **ML-172** | O=[S](=O)(N([H])C1=N/C(=C(/[H])[S]1)C=2C([H])=C([H])C([Cl])=C([H])C=2[H])C([H])([H])[H] | Inactive | 30 |
| **ML-173** | O=C(C=1N=C([S]C=1[H])N([H])C=2C([H])=C([H])C([H])=C(O[H])C=2[H])C([H])([H])[H] | Inactive | 30 |
| **ML-174** | O=C(C=1N=C([S]C=1[H])N([H])C(=O)C=2C([H])=C([H])C([H])=C([H])C=2[H])C([H])([H])[H] | Inactive | 30 |
| **ML-175** | O=C(C=1N=C([S]C=1[H])N([H])C=2C([H])=C([H])C(OC([H])([H])[H])=C([H])C=2[H])C([H])([H])[H] | Inactive | 30 |
| **ML-176** | [O-][N+](=O)C=1C([H])=C([H])C(=C([H])C=1[H])C=2/N=C(/[S]C([H])([H])C#C[H])N=C([Cl])C=2C#N | Inactive | 18 |
| **ML-177** | [F]C=3C([H])=C([H])C(N([H])C=2/N=C(/[S]C([H])([H])C#C[H])N=C(C=1C([H])=C([H])C([H])=C([H])C=1[H])C=2C#N)=C([H])C=3[H] | Inactive | 18 |
| **ML-178** | [Cl]C=1C([H])=C([H])C([H])=C([H])C=1C([H])([H])N2N=N/C(=C2/[H])C([H])([H])[S]C=3/N=C(/[Cl])C(C#N)=C(N=3)C=4C([H])=C([H])C([H])=C([H])C=4[H] | Inactive | 18 |
| **ML-179** | [Cl]C=1C([H])=C([H])C([H])=C(C=1[H])C=2/N=C(/[S]C([H])([H])C#C[H])N=C([Cl])C=2C#N | Inactive | 18 |
| **ML-180** | [Cl]C=1C([H])=C([H])C(=C([H])C=1[H])C=4/N=C(/[S]C([H])([H])C=2N=NN(C=2[H])C([H])([H])C=3C([H])=C([H])C([H])=C([H])C=3[Cl])N=C([Cl])C=4C#N | Inactive | 18 |
| **ML-181** | O=[N+]([O-])C=2C([H])=C([H])C(/C1=N/C(=NC(N([H])N([H])C(=N[H])N([H])[H])=C1C#N)[S]C([H])([H])C#C[H])=C([H])C=2[H] | Inactive | 18 |
| **ML-182** | N#C/C1=C(/N=C(/[S]C([H])([H])C#C[H])N=C1C=2C([H])=C([H])C([H])=C([H])C=2[H])N([H])C=3C([H])=C([H])C(=C([H])C=3[H])C([H])([H])[H] | Inactive | 18 |

**Table SM10**: ML Training list compounds

| Compound ID | SMILES | Activity class | Source |
|---|---|---|---|
| **CHEMBL1468131** | O=C(N([H])C1=C(OC([H])([H])[H])C([H])=C([H])C([H])=C1[H])C([H])([H])C(=N[H])N([H])O[H] | Inactive | ChEMBL database |
| **CHEMBL1938900** | O=C(OC([H])([H])C=1C([H])=C([H])C(=C([H])C=1[H])C([H])([H])N([H])C([H])([H])C([H])([H])C([H])([H])C([H])([H])N(O[H])C(=O)C([H])=C([H])C(=O)O[H])N([H])C3=C([H])C([H])=C([H])C=2C([H])=C([H])C([H])=C([H])C=23 | Inactive | ChEMBL database |
| **CHEMBL2181951** | O=C(N([H])C=1C([H])=C([H])C([F])=C([F])C=1[F])C([H])([H])C(=NO[H])N([H])[H] | Inactive | ChEMBL database |
| **CHEMBL2181952** | O=C(N([H])C=1C([H])=C([H])C([F])=C([H])C=1[F])C([H])([H])C(=NO[H])N([H])[H] | Inactive | ChEMBL database |
| **CHEMBL2181953** | O=C(N([H])C=1C([H])=C([H])C([H])=C([F])C=1[F])C([H])([H])C(=NO[H])N([H])[H] | Inactive | ChEMBL database |
| **CHEMBL2181954** | O=C(N([H])C=1C([H])=C([H])C([F])=C([F])C=1[H])C([H])([H])C(=NO[H])N([H])[H] | Inactive | ChEMBL database |
| **CHEMBL2181956** | O=C(N([H])C=1C([H])=C([H])C([H])=C([F])C=1[H])C([H])([H])C(=NO[H])N([H])[H] | Inactive | ChEMBL database |
| **CHEMBL2181958** | O=C(N([H])C1=C(C([H])=C([H])C([H])=C1[H])C([H])([H])[H])C([H])([H])C(=NO[H])N([H])[H] | Inactive | ChEMBL database |
| **CHEMBL2181961** | O=C(N([H])C1=C(C([H])=C([H])C([H])=C1[H])C([H])([H])C([H])([H])[H])C([H])([H])C(=NO[H])N([H])[H] | Inactive | ChEMBL database |
| **CHEMBL2181962** | O=C(N([H])C1=C(C([H])=C([H])C([H])=C1[H])C([H])(C([H])([H])[H])C([H])([H])[H])C([H])([H])C(=NO[H])N([H])[H] | Inactive | ChEMBL database |
| **CHEMBL2181963** | O=C(N([H])C=1C([H])=C([H])C([H])=C([H])C=1C=2C([H])=C([H])C([H])=C([H])C=2[H])C([H])([H])C(=NO[H])N([H])[H] | Inactive | ChEMBL database |
| **CHEMBL2182005** | O=C(N([H])C=1C([H])=C([H])C([H])=C([H])C=1C([F])([F])[F])C([H])([H])C(=NO[H])N([H])[H] | Inactive | ChEMBL database |
| **CHEMBL2182006** | O=C(N([H])C=1C([H])=C([H])C([H])=C([H])C=1[Cl])C([H])([H])C(=NO[H])N([H])[H] | Inactive | ChEMBL database |
| **CHEMBL2182007** | O=C(N([H])C=1C([H])=C([H])C([H])=C([H])C=1[Br])C([H])([H])C(=NO[H])N([H])[H] | Inactive | ChEMBL database |

| **Table SM10**: ML Training list compounds | | | |
|---|---|---|---|
| **Compound ID** | **SMILES** | **Activity class** | **Source** |
| **CHEMBL2426056** | [Br]C=2C([H])=C([H])C(C=1C([H])=C([H])C(OC([H])([H])[C@]([H])(O[H])C([H])([H])N([H])C([H])([H])C#C[H])=C([H])C=1[H])=C([H])C=2[H] | Inactive | ChEMBL database |
| **CHEMBL2426057** | [Cl]C=2C([H])=C([H])C(C=1C([H])=C([H])C(OC([H])([H])[C@]([H])(O[H])C([H])([H])N([H])C([H])([H])C#C[H])=C([H])C=1[H])=C([Cl])C=2[H] | Inactive | ChEMBL database |
| **CHEMBL2426058** | [Cl]/C2=C(\[H])C(C=1C([H])=C([H])C(OC([H])([H])[C@]([H])(O[H])C([H])([H])N([H])C([H])([H])C#C[H])=C([H])C=1[H])=C([H])C([Cl])=C2[H] | Inactive | ChEMBL database |
| **CHEMBL2426059** | O=C(OC([H])([H])[H])C=1C([H])=C([H])C(OC([H])([H])[C@]([H])(O[H])C([H])([H])N(C([H])([H])[H])C([H])([H])C#C[H])=C([H])C=1[H] | Inactive | ChEMBL database |
| **CHEMBL2426060** | O=C(OC([H])([H])[H])C=1C([H])=C([H])C(OC([H])([H])[C@]([H])(O[H])C([H])([H])N([H])C([H])([H])C#C[H])=C([H])C=1[H] | Inactive | ChEMBL database |
| **CHEMBL2426061** | O=C(OC([H])([H])[H])C([H])([H])C=1C([H])=C([H])C(OC([H])([H])[C@]([H])(O[H])C([H])([H])N(C([H])([H])[H])C([H])([H])C#C[H])=C([H])C=1[H] | Inactive | ChEMBL database |

| Table SM10: ML Training list compounds | | | |
|---|---|---|---|
| **Compound ID** | **SMILES** | **Activity class** | **Source** |
| **CHEMBL2426062** | O=C(OC([H])([H])[H])C([H])([H])C=1C([H])=C([H])C(OC([H])([H])[C@]([H])(O[H])C([H])([H])N([H])C([H])([H])C#C[H])=C([H])C=1[H] | Inactive | ChEMBL database |
| **CHEMBL2426063** | O=C(OC([H])([H])[H])C([H])([H])C([H])([H])C=1C([H])=C([H])C(OC([H])([H])[C@]([H])(O[H])C([H])([H])N(C([H])([H])[H])C([H])([H])C#C[H])=C([H])C=1[H] | Inactive | ChEMBL database |
| **CHEMBL2426064** | O=C(N([H])[H])C=1C([H])=C([H])C(OC([H])([H])[C@]([H])(O[H])C([H])([H])N([H])C([H])([H])C#C[H])=C([H])C=1[H] | Inactive | ChEMBL database |
| **CHEMBL2426129** | O=C(C=1C([H])=C([H])C(OC([H])([H])[C@@]([H])(O[H])C([H])([H])N(C([H])([H])[H])C([H])([H])C#C[H])=C([H])C=1[H])N([H])[H] | Inactive | ChEMBL database |
| **CHEMBL2426130** | [H]O[C@]([H])(C([H])([H])N([H])C([H])([H])C#C[H])C([H])([H])N([H])C=1C([H])=C([H])C(=C([H])C=1[H])C=2C([H])=C([H])C([H])=C([H])C=2[H] | Inactive | ChEMBL database |
| **CHEMBL2426131** | [H]O[C@@]([H])(C([H])([H])N(C=1C([H])=C([H])C(=C([H])C=1[H])C=2C([H])=C([H])C([H])=C([H])C=2[H])C([H])([H])[H])C([H])([H])N([H])C([H])([H])C#C[H] | Inactive | ChEMBL database |
| **CHEMBL3134311** | [Cl]C=2C([H])=C([H])C(N([H])C=1[S]C([H])=C(N=1)C([H])([H])C([H])([H])[H])=C([H])C=2[H] | Inactive | ChEMBL database |
| **CHEMBL3134312** | [Cl]C=2C([H])=C([H])C(N([H])C=1[S]C([H])=C(N=1)[C@@]([H])(O[H])C([H])([H])[H])=C([H])C=2[H] | Inactive | ChEMBL database |
| **CHEMBL3134314** | [F]C=1C([H])=C([H])C(=C([H])C=1[H])C=2N=C([S]C=2[H])N([H])C=3C([H])=C([H])C([Cl])=C([H])C=3[H] | Inactive | ChEMBL database |
| **CHEMBL3217220** | O=[N+]([O-])C1=C(N([H])C([H])([H])C([H])([H])N([H])[H])C(=C([H])C(=C1[H])C([F])([F])[F])[N+](=O)[O-] | Inactive | ChEMBL database |
| **CHEMBL4064882** | O=[S](=O)(N([H])C1=C([H])C(OC([H])([H])[H])=C(C([H])=C1[H])N2N=C(C([H])=C2C([H])([H])[H])C([H])([H])[H])C=4C([H])=C([H])C=3N(C(=O)C(=C([H])C=3C=4[H])C([H])([H])[H])C([H])([H])[H] | Inactive | ChEMBL database |

**Table SM10**: ML Training list compounds



| Compound ID | SMILES | Activity class | Source |
|---|---|---|---|
| **CHEMBL4096792** | [H]N([H])C(=NO[H])C=2C([H])=C([H])C(C([H])=C([H])C=1C([H])=C(O[H])C(O[H])=C(O[H])C=1[H])=C([H])C=2[H] | Inactive | ChEMBL database |
| **CHEMBL8706** | [Cl]C=1C([H])=C([H])C(OC([H])([H])C([H])([H])C([H])([H])N(C([H])([H])[H])C([H])([H])C#C[H])=C([Cl])C=1[H] | Inactive | ChEMBL database |
| **ML-183** | O=C(OC(C([H])([H])[H])(C([H])([H])[H])C([H])([H])[H])N4C([H])([H])C([H])([H])N(C(=[S])[S]C([H])([H])C=1N=NN(C=1[H])C([H])([H])C3=C([H])C(=O)OC=2C([H])=C([H])C([H])=C([H])C=23)C([H])([H])C4([H])[H] | Moderate | 27 |
| **ML-184** | [Br]/C1=C(/C([H])=C([H])C([H])=C1[H])C([H])([H])[S]C=3N=C2N=C(C([H])=C(N2N=3)N([H])N([H])[H])C([H])([H])[H] | Moderate | 31 |
| **ML-185** | [F]C=1C([H])=C([H])C(=C([H])C=1[H])C([H])([H])N2N=N/C(=C2/[H])C([H])([H])[S]C(=[S])N3C([H])([H])C([H])([H])N([H])C([H])([H])C3([H])[H] | Moderate | 28 |
| **ML-186** | [S]=C([S]C([H])([H])C=1N=NN(C=1[H])C([H])([H])C=2C([H])=C([H])C(=C([H])C=2[H])C([H])([H])[H])N3C([H])([H])C([H])([H])N([H])C([H])([H])C3([H])[H] | Moderate | 28 |
| **ML-187** | [H]C=2C(=NC1=NC(=NN1C=2N([H])C3=C([H])C([H])=C(C([H])=C3[H])N4C([H])([H])C([H])([H])N(C([H])([H])C4([H])[H])C([H])([H])[H])[S]C([H])([H])C([H])([H])C5=C([H])C([H])=C([H])C([H])=C5[H])C([H])([H])[H] | Moderate | 19 |
| **ML-188** | [S]=C([S]C([H])([H])C=1N=NN(C=1[H])C([H])([H])C=2C([H])=C([H])C(=C([H])C=2[H])C([H])([H])[H])N4C([H])([H])C([H])([H])N(C(=O)OC([H])([H])C=3C([H])=C([H])C([H])=C([H])C=3[H])C([H])([H])C4([H])[H] | Moderate | 28 |
| **ML-189** | O=C(OC(C([H])([H])[H])(C([H])([H])[H])C([H])([H])[H])N3C([H])([H])C([H])([H])N(C(=[S])[S]C([H])([H])C=1N=NN(C=1[H])C([H])([H])C=2C([H])=C([H])C([H])=C([H])C=2[F])C([H])([H])C3([H])[H] | Moderate | 28 |
| **ML-190** | O=C(OC(C([H])([H])[H])(C([H])([H])[H])C([H])([H])[H])N3C([H])([H])C([H])([H])N(C(=[S])[S]C([H])([H])C=1N=NN(C=1[H])C([H])([H])C=2C([H])=C([H])C([H])=C([H])C=2[Cl])C([H])([H])C3([H])[H] | Moderate | 28 |

**Table SM10**: ML Training list compounds

| Compound ID | SMILES | Activity class | Source |
|---|---|---|---|
| **ML-191** | N#C/C1=C(\[H])C([H])=C(C([H])=C1[H])[C@@]4([H])[C@]([H])(C2=C([H])C([H])=C(/N=C2/[H])OC([H])([H])[H])C([H])([H])N(C(=O)C([H])([H])[C@]3([H])C([H])([H])N([H])C([H])([H])C([H])([H])C3([H])[H])C4([H])[H] | Moderate | 34 |
| **ML-192** | [H]C=2C(=NC1=NC([H])=NN1C=2N([H])C3=C([H])C([H])=C(C([H])=C3[H])N4C([H])([H])C([H])([H])N(C([H])([H])C4([H])[H])C([H])([H])[H])C([H])([H])C([H])([H])[H] | Moderate | 19 |
| **ML-193** | [H]C=2C(=NC1=NC(=NN1C=2N([H])C([H])([H])C([H])([H])N([H])[H])[S]C([H])([H])C3=C([H])C([H])=C([H])C([H])=C3[H])C([H])([H])[H] | Moderate | 31 |
| **ML-194** | [F]C=1C([H])=C([H])C([H])=C([H])C=1C([H])([H])N2N=N/C(=C2/[H])C([H])([H])[S]C(=[S])N3C([H])([H])C([H])([H])N([H])C([H])([H])C3([H])[H] | Moderate | 28 |
| **ML-195** | O=C(OC(C([H])([H])[H])(C([H])([H])[H])C([H])([H])[H])N3C([H])([H])C([H])([H])N(C(=[S])[S]C([H])([H])C=1N=NN(C=1[H])C([H])([H])C=2C([H])=C([H])C([H])=C([H])C=2[Br])C([H])([H])C3([H])[H] | Moderate | 28 |
| **ML-196** | [H]C=2C(=NC1=NC(=NN1C=2N([H])C3=C([H])C([H])=C(C([H])=C3[H])N4C([H])([H])C([H])([H])N(C([H])([H])C4([H])[H])C([H])([H])[H])[S]C([H])([H])C([H])([H])C([H])([H])C5=C([H])C([H])=C([H])C([H])=C5[H])C([H])([H])[H] | Moderate | 19 |
| **ML-197** | [S]=C([S]C([H])([H])C=1N=NN(C=1[H])C([H])([H])C=2C([H])=C([H])C(=C([H])C=2[H])C([H])([H])[H])N4C([H])([H])C([H])([H])N(C=3/N=C(/[H])C([H])=C([H])N=3)C([H])([H])C4([H])[H] | Moderate | 28 |
| **ML-198** | [F]C=1C([H])=C([H])C([H])=C([H])C=1C([H])([H])N2N=N/C(=C2/[H])C([H])([H])[S]C(=[S])N4C([H])([H])C([H])([H])N(C=3/N=C(/[H])C([H])=C([H])N=3)C([H])([H])C4([H])[H] | Moderate | 28 |
| **ML-199** | [S]=C([S]C([H])([H])C=1N=NN(C=1[H])C([H])([H])C=2C([H])=C([H])C(=C([H])C=2[H])C([H])([H])[H])N3C([H])([H])C([H])([H])N(C([H])([H])C3([H])[H])[S](=O)(=O)C([H])([H])[H] | Moderate | 28 |

| Table SM10: ML Training list compounds | | | |
|---|---|---|---|
| **Compound ID** | **SMILES** | **Activity class** | **Source** |
| **ML-200** | [H]C=2C(=NC1=NC(=NN1C=2N([H])C3=C([H])C([H])=C(C([H])=C3[H])N4C([H])([H])C([H])([H])N(C([H])([H])C4([H])[H])C([H])([H])[H])[S]C([H])([H])C([H])([H])C([H])([H])[H])C([H])([H])[H] | Moderate | 19 |
| **ML-201** | [H]C=2C(=NC1=NC(=NN1C=2N([H])N([H])[H])[S]C([H])([H])C3=C([H])C([H])=C(C([H])=C3[H])C([H])([H])[H])C([H])([H])[H] | Moderate | 31 |
| **ML-202** | O=C(OC([H])([H])C([H])([H])[H])N3C([H])([H])C([H])([H])N(C(=[S])[S]C([H])([H])C=1N=NN(C=1[H])C([H])([H])C=2C([H])=C([H])C(=C([H])C=2[H])C([H])([H])[H])C([H])([H])C3([H])[H] | Moderate | 28 |
| **ML-203** | O=C(OC([H])(C([H])([H])[H])C([H])([H])[H])N3C([H])([H])C([H])([H])N(C(=[S])[S]C([H])([H])C=1N=NN(C=1[H])C([H])([H])C=2C([H])=C([H])C(=C([H])C=2[H])C([H])([H])[H])C([H])([H])C3([H])[H] | Moderate | 28 |
| **ML-204** | O=C(N3C([H])([H])C([H])([H])N(C(=[S])[S]C([H])([H])C=1N=NN(C=1[H])C([H])([H])C=2C([H])=C([H])C(=C([H])C=2[H])C([H])([H])[H])C([H])([H])C3([H])[H])C([H])([H])[H] | Moderate | 28 |
| **ML-205** | [H]C=2C(=NC1=NC(=NN1C=2N([H])C3=C([H])C([H])=C(C([H])=C3[H])N4C([H])([H])C([H])([H])N(C([H])([H])C4([H])[H])C([H])([H])[H])[S]C([H])([H])C([H])=C([H])[H])C([H])([H])[H] | Moderate | 19 |
| **ML-206** | [Cl]/C1=C(/C([Cl])=C([H])C([H])=C1[H])C([H])([H])[S]C=3N=C2N=C(C([H])=C(N2N=3)N([H])N([H])[H])C([H])([H])[H] | Moderate | 31 |
| **ML-207** | [H]C=2C(=NC1=NC(=NN1C=2N([H])C3=C([H])C([H])=C(C([H])=C3[H])N4C([H])([H])C([H])([H])N(C([H])([H])C4([H])[H])C([H])([H])[H])[S]C([H])([H])C#C[H])C([H])([H])[H] | Moderate | 19 |

**Table SM10**: ML Training list compounds

| Compound ID | SMILES | Activity class | Source |
|---|---|---|---|
| **ML-208** | [H]C([H])([H])C=1C([H])=C([H])C(=C([H])C=1[H])C=3/N=C(/[H])C(OC([H])([H])C2([H])C([H])([H])C([H])([H])N([H])C([H])([H])C2([H])[H])=C([H])C=3C=4C([H])=C([H])C(=C([H])C=4[H])C([H])([H])N5C([H])([H])C([H])([H])OC([H])([H])C5([H])[H] | Moderate | 25 |
| **ML-209** | [H]C=2C(=NC1=NC(=NN1C=2N([H])C3=C([H])C([H])=C(C([H])=C3[H])N4C([H])([H])C([H])([H])N(C([H])([H])C4([H])[H])C([H])([H])[H])[S]C([H])([H])C5([H])C([H])([H])C5([H])[H])C([H])([H])[H] | Moderate | 19 |
| **ML-210** | [F]C([F])([F])C1=C(C([H])=C([H])C([H])=C1[H])C([H])([H])[S]C=3N=C2N=C(C([H])=C(N2N=3)N([H])N([H])[H])C([H])([H])[H] | Moderate | 31 |
| **ML-211** | [H]C([H])(O/C1=N/C(=C(/N=C1/[H])C=2C([H])=C([H])C([H])=C([H])C=2[H])C=3C([H])=C([H])C([H])=C([H])C=3[H])C4([H])C([H])([H])C([H])([H])N([H])C([H])([H])C4([H])[H] | Moderate | 25 |
| **ML-212** | [H]C([H])([H])C=1C([H])=C([H])C(=C([H])C=1[H])C=3/N=C(/[H])C(OC([H])([H])C2([H])C([H])([H])C([H])([H])N([H])C([H])([H])C2([H])[H])=C([H])C=3C=4C([H])=C([H])C(=C([H])C=4[H])C([H])([H])N5C([H])([H])C([H])([H])N([H])C([H])([H])C5([H])[H] | Moderate | 25 |
| **ML-213** | [Br]C=1C([H])=C([H])C(=C([H])C=1[H])C=3/N=C(/OC([H])([H])C2([H])C([H])([H])C([H])([H])N([H])C([H])([H])C2([H])[H])C([H])=NC=3C=4C([H])=C([H])C(C#N)=C([H])C=4[H] | Moderate | 25 |
| **ML-214** | [H]C([H])([H])C([H])([H])N([H])C([H])([H])C=1C([H])=C([H])C(=C([H])C=1[H])C=3C([H])=C(OC([H])([H])C2([H])C([H])([H])C([H])([H])N([H])C([H])([H])C2([H])[H])C([H])=NC=3C=4C([H])=C([H])C(=C([H])C=4[H])C([H])([H])[H] | Moderate | 25 |
| **ML-215** | [Br]C=1C([H])=C([H])C(=C([H])C=1[H])C=3/N=C(/[H])C(OC([H])([H])C2([H])C([H])([H])C([H])([H])N([H])C([H])([H])C2([H])[H])=NC=3C=4C([H])=C([H])C([Br])=C([H])C=4[H] | Moderate | 25 |
| **ML-216** | [H]C=2C(=NC1=NC(=NN1C=2N([H])C3=C([H])C([H])=C(C([H])=C3[H])N4C([H])([H])C([H])([H])N(C([H])([H])C4([H])[H])C([H])([H])[H])[S]C([H])([H])C5([H])C([H])([H])C([H])([H])C([H])([H])C([H])([H])C5([H])[H])C([H])([H])[H] | Moderate | 19 |

| Table SM10: ML Training list compounds | | | |
|---|---|---|---|
| **Compound ID** | **SMILES** | **Activity class** | **Source** |
| **ML-217** | [F]C([F])([F])C=1C([H])=C(C([H])=C([H])C=1[H])C([H])([H])[S]C=3N=C2N=C(C([H])=C(N2N=3)N([H])N([H])[H])C([H])([H])[H] | Moderate | 31 |
| **ML-218** | N#CC=1C([H])=C([H])C(=C([H])C=1[H])C=3/N=C(/[H])C(OC([H])([H])C2([H])C([H])([H])C([H])([H])N([H])C([H])([H])C2([H])[H])=NC=3C=4C([H])=C([H])C(C#N)=C([H])C=4[H] | Moderate | 25 |
| **ML-219** | [H]C([H])([H])C=1C([H])=C([H])C(=C([H])C=1[H])C=3/N=C(/[H])C(OC([H])([H])C2([H])C([H])([H])C([H])([H])N([H])C([H])([H])C2([H])[H])=C([H])C=3C=4C([H])=C([H])C(=C([H])C=4[H])C([H])([H])O[H] | Moderate | 25 |
| **ML-220** | O=C(C=4C([H])=C([H])C(/C2=C(/N=C(/[H])C(OC([H])([H])[C@@]1([H])C([H])([H])C([H])([H])N([H])C([H])([H])C1([H])[H])=C2[H])C=3C([H])=C([H])C(=C([H])C=3[H])C([H])([H])[H])=C([H])C=4[H])N([H])[C@@]5([H])C([H])([H])C5([H])[H] | Moderate | 25 |
| **ML-221** | N#CC=3C([H])=C([H])C(/C1=C(/N=C(/[H])C(O[H])=C1[H])C=2C([H])=C([H])C(=C([H])C=2[H])C([H])([H])[H])=C([H])C=3[H] | Moderate | 25 |
| **ML-222** | N#CC=4C([H])=C([H])C(/C2=C(/N=C(/[H])C(OC([H])([H])C=1C([H])=C([H])C([H])=C([H])C=1[H])=C2[H])C=3C([H])=C([H])C(=C([H])C=3[H])C([H])([H])[H])=C([H])C=4[H] | Moderate | 25 |
| **ML-223** | [H]C=2C(=NC1=NC(=NN1C=2N([H])N([H])[H])[S]C([H])([H])C([H])([H])C3=C([H])C([H])=C([H])C([H])=C3[H])C([H])([H])[H] | Moderate | 31 |
| **ML-224** | [H]C=2C(=NC1=NC([H])=NN1C=2N([H])C3=C([H])C([H])=C(C([H])=C3[H])N4C([H])([H])C([H])([H])N(C([H])([H])C4([H])[H])C([H])([H])[H])C([H])([H])[H] | Moderate | 19 |

**Table SM10**: ML Training list compounds 

| Compound ID | SMILES | Activity class | Source |
|---|---|---|---|
| **ML-225** | [H]C=2C(=NC1=NC(=NN1C=2N([H])[H])[S]C([H])([H])C3=C([H])C([H])=C([H])C([H])=C3[H])C([H])([H])[H] | Moderate | 31 |
| **ML-226** | O=C(C=4C([H])=C([H])C(/C2=C(/N=C(/[H])C(OC([H])([H])C1([H])C([H])([H])C([H])([H])N([H])C([H])([H])C1([H])[H])=C2[H])C=3C([H])=C([H])C(=C([H])C=3[H])C([H])([H])[H])=C([H])C=4[H])N5C([H])([H])C([H])([H])OC([H])([H])C5([H])[H] | Moderate | 25 |
| **ML-227** | O=C(C=4C([H])=C([H])C(/C2=C(/N=C(/[H])C(OC([H])([H])C1([H])C([H])([H])C([H])([H])N([H])C([H])([H])C1([H])[H])=C2[H])C=3C([H])=C([H])C(=C([H])C=3[H])C([H])([H])[H])=C([H])C=4[H])N5C([H])([H])C([H])([H])N([H])C([H])([H])C5([H])[H] | Moderate | 25 |
| **ML-228** | O=C(C=4C([H])=C([H])C(/C2=C(/N=C(/[H])C(OC([H])([H])C1([H])C([H])([H])C([H])([H])N([H])C([H])([H])C1([H])[H])=C2[H])C=3C([H])=C([H])C(=C([H])C=3[H])C([H])([H])[H])=C([H])C=4[H])N5C([H])([H])C([H])([H])C([H])([H])C([H])([H])C5([H])[H] | Moderate | 25 |
| **ML-229** | [H]C([H])(OC=2C([H])=NC(C=1C([H])=C([H])C([H])=C([H])C=1[H])=C(C=2[H])C=3C([H])=C([H])C([H])=C([H])C=3[H])C4([H])C([H])([H])C([H])([H])N([H])C([H])([H])C4([H])[H] | Moderate | 25 |
| **ML-230** | [H]C([H])(OC=1C([H])=C([H])C(=NC=1[H])C=2C([H])=C([H])C([H])=C([H])C=2[H])C3([H])C([H])([H])C([H])([H])N([H])C([H])([H])C3([H])[H] | Moderate | 25 |
| **ML-231** | [O-][N+](=O)C=2C([H])=C([H])C=1O/C(=C(/[Cl])C(=O)C=1C=2[H])C([F])([F])[F] | Moderate | 23 |
| **ML-232** | [H]C=2C(=NC1=NC(=NN1C=2N([H])N([H])[H])[S]C([H])([H])C([H])([H])C([H])([H])C3=C([H])C([H])=C([H])C([H])=C3[H])C([H])([H])[H] | Moderate | 31 |

**Table SM10**: ML Training list compounds

| Compound ID | SMILES | Activity class | Source |
|---|---|---|---|
| **ML-233** | O=C(N([H])N=C(/C2=C(\[H])C([H])=C([H])C=1C([H])=C([H])C([H])=C([H])C=12)C([H])([H])[H])C=3C([H])=C([H])C([H])=C(C=3[H])[S](=O)(=O)N4C([H])([H])C([H])([H])OC([H])([H])C4([H])[H] | Moderate | 23 |
| **ML-234** | [Cl]/C1=C(\[H])C([H])=C(C([H])=C1[H])C([H])([H])[S]C=3N=C2N=C(C(=C(N2N=3)N([H])C4=C([H])C([H])=C(C([H])=C4[H])N5C([H])([H])C([H])([H])N(C([H])([H])C5([H])[H])C([H])([H])[H])C([H])([H])[H])C([H])([H])[H] | Moderate | 19 |
| **ML-235** | [H]C=2C(=NC1=NC(=NN1C=2N([H])N([H])[H])[S]C([H])([H])C3=C([H])C([H])=C([H])C([H])=C3[H])C([H])(C([H])([H])[H])C([H])([H])[H] | Moderate | 31 |
| **ML-236** | [Cl]/C1=C(\[H])C([H])=C(C([H])=C1[H])C([H])([H])[S]C=3N=C2N=C(C(=C(N2N=3)N([H])C4=C([H])C([H])=C(C([H])=C4[H])N5C([H])([H])C([H])([H])N(C([H])([H])C5([H])[H])C([H])([H])[H])C([H])([H])C([H])([H])C([H])([H])C([H])([H])C([H])([H])[H])C([H])([H])[H] | Moderate | 19 |
| **ML-237** | [H]C=2C(=NC1=NC(=NN1C=2N([H])N([H])[H])[S]C([H])([H])C3=C([H])C([H])=C([H])C([H])=C3[H])C4=C([H])C([H])=C([H])C([H])=C4[H] | Moderate | 31 |
| **ML-238** | O=C(N([H])C1=C([H])C([H])=C([H])C(OC([H])([H])C([H])([H])C([H])([H])N(C([H])([H])[H])C([H])([H])C#C[H])=C1[H])C=2C([H])=C([H])C([H])=C([H])C=2[H] | Moderate | 22 |
| **ML-239** | [H]O[C@]([H])(C([H])([H])N([H])C([H])([H])C#C[H])C([H])([H])OC=1C([H])=C([H])C(=C([H])C=1[H])C=2C([H])=C([H])C([H])=C([H])C=2[H] | Moderate | 22 |
| **ML-240** | [H]C([H])([H])N(C([H])([H])C#C[H])C([H])([H])[C@]([H])(O[H])C([H])([H])OC=2C([H])=C([H])C1=C([H])C([H])=C([H])C([H])=C1C=2[H] | Moderate | 22 |
| **ML-241** | [H]O[C@]([H])(C([H])([H])N([H])C([H])([H])C#C[H])C([H])([H])OC=2C([H])=C([H])C1=C([H])C([H])=C([H])C([H])=C1C=2[H] | Moderate | 22 |

**Table SM10**: ML Training list compounds

| Compound ID | SMILES | Activity class | Source |
|---|---|---|---|
| **ML-242** | [Br]C=2C([H])=C1C([H])=C([H])C(OC([H])([H])[C@@]([H])(O[H])C([H])([H])N(C([H])([H])[H])C([H])([H])C#C[H])=C([H])C1=C([H])C=2[H] | Moderate | 22 |
| **ML-243** | [H]C([H])([H])[S]C([H])([H])C([H])([H])C=1N=C(OC=1C([H])([H])[H])C=2C([H])=C([H])C([H])=C(N([H])C(=N[H])N([H])[H])C=2[H] | Moderate | 30 |
| **ML-244** | [H]C=2C(=NC1=NC([H])=NN1C=2N([H])C3=C([H])C([H])=C(C([H])=C3[H])N4C([H])([H])C([H])([H])N(C([H])([H])C4([H])[H])C([H])([H])[H])C5=C([H])C([H])=C([H])C([H])=C5[H] | Moderate | 19 |
| **ML-245** | [H]C=2C(=NC1=NC(=NN1C=2N([H])C([H])([H])C([H])([H])OC([H])([H])C([H])([H])OC([H])([H])C([H])([H])N([H])[H])[S]C([H])([H])C3=C([H])C([H])=C([H])C([H])=C3[H])C([H])([H])[H] | Moderate | 31 |
| **ML-246** | O=C(C=1N=C([S]C=1[H])N([H])C=2C([H])=C([H])C([Cl])=C([H])C=2[H])C([H])([H])[H] | Moderate | 30 |
| **ML-247** | O=C(C=1N=C([S]C=1[H])N([H])C=2C([H])=C([H])C([H])=C([Cl])C=2[H])C([H])([H])[H] | Moderate | 30 |
| **ML-248** | O=C(C=1N=C([S]C=1[H])N([H])C=2C([H])=C([H])C([Br])=C([H])C=2[H])C([H])([H])[H] | Moderate | 30 |
| **ML-249** | O=C(C=1N=C([S]C=1[H])N([H])C=2C([H])=C([H])C(O[H])=C([H])C=2[H])C([H])([H])[H] | Moderate | 30 |
| **ML-250** | [Cl]C=1C([H])=C([H])C(=C([H])C=1[H])C=2N=C([S]C=2[H])N([H])[S](=O)(=O)C=3C([H])=C([H])C([H])=C([H])C=3[H] | Moderate | 30 |
| **ML-251** | [F]C([F])([F])[S](=O)(=O)N([H])C1=N/C(=C(/[H])[S]1)C=2C([H])=C([H])C([Cl])=C([H])C=2[H] | Moderate | 30 |
| **ML-252** | O=[S](=O)(C1=C(C([H])=C([H])C([H])=C1[H])C([H])([H])[H])N([H])C2=N/C(=C(/[H])[S]2)C=3C([H])=C([H])C([Cl])=C([H])C=3[H] | Moderate | 30 |

| Table SM10: ML Training list compounds | | | |
|---|---|---|---|
| Compound ID | SMILES | Activity class | Source |
| ML-253 | O=[S](=O)(C1=C([H])C([H])=C([H])C(=C1[H])C([H])([H])[H])N([H])C2=N/C(=C(/[H])[S]2)C=3C([H])=C([H])C([Cl])=C([H])C=3[H] | Moderate | 30 |
| ML-254 | O=[S](=O)(C1=C([H])C([H])=C([H])C(OC([H])([H])[H])=C1[H])N([H])C2=N/C(=C(/[H])[S]2)C=3C([H])=C([H])C([Cl])=C([H])C=3[H] | Moderate | 30 |
| ML-255 | O=[S](=O)(C=1C([H])=C([H])C(OC([H])([H])[H])=C([H])C=1[H])N([H])C2=N/C(=C(/[H])[S]2)C=3C([H])=C([H])C([Cl])=C([H])C=3[H] | Moderate | 30 |
| ML-256 | [H]C=2C(=NC1=NC(=NN1C=2N([H])C3=C([H])C([H])=C(C([H])=C3[H])N([H])C4=C([H])C([H])=C([H])C([H])=C4[H])[S]C([H])([H])C5=C([H])C([H])=C([H])C([H])=C5[H])C([H])([H])[H] | Moderate | 19 |
| ML-257 | [H]N(/C2=C4/C(=NC1=NC(=NN12)[S]C([H])([H])C3=C([H])C([H])=C([H])C([H])=C3[H])C([H])([H])C([H])([H])C4([H])[H])N([H])[H] | Moderate | 31 |
| ML-258 | [Cl]C=1C([H])=C([H])C(=C([H])C=1[H])C=2N=C([S]C=2[H])N([H])[S](=O)(=O)C=3C([H])=C([H])C([H])=C(C=3[H])C=4C([H])=C([H])C([H])=C([H])C=4[H] | Moderate | 30 |
| ML-259 | [Cl]C=1C([H])=C([H])C(=C([H])C=1[H])C=2N=C([S]C=2[H])N([H])[S](=O)(=O)C=3C([H])=C([H])C(=C([H])C=3[H])C=4C([H])=C([H])C([H])=C([H])C=4[H] | Moderate | 30 |
| ML-260 | [Cl]C=1C([H])=C([H])C(=C([H])C=1[H])C=2N=C([S]C=2[H])N([H])[S](=O)(=O)C=3C([H])=C([H])C([H])=C([H])C=3[Cl] | Moderate | 30 |
| ML-261 | [Cl]C=1C([H])=C([H])C(=C([H])C=1[H])C=2N=C([S]C=2[H])N([H])[S](=O)(=O)C=3C([H])=C([H])C([H])=C([Cl])C=3[H] | Moderate | 30 |
| ML-262 | [Cl]C=1C([H])=C([H])C(=C([H])C=1[H])C=2N=C([S]C=2[H])N([H])[S](=O)(=O)C=3C([H])=C([H])C([Cl])=C([H])C=3[H] | Moderate | 30 |

**Table SM10**: ML Training list compounds

| Compound ID | SMILES | Activity class | Source |
|---|---|---|---|
| **ML-263** | [F]C([F])([F])C=1C([H])=C([H])C([H])=C([H])C=1[S](=O)(=O)N([H])C2=N/C(=C(/[H])[S]2)C=3C([H])=C([H])C([Cl])=C([H])C=3[H] | Moderate | 30 |
| **ML-264** | [F]C([F])([F])C=1C([H])=C([H])C([H])=C(C=1[H])[S](=O)(=O)N([H])C2=N/C(=C(/[H])[S]2)C=3C([H])=C([H])C([Cl])=C([H])C=3[H] | Moderate | 30 |
| **ML-265** | [F]C([F])([F])C=1C([H])=C([H])C(=C([H])C=1[H])[S](=O)(=O)N([H])C2=N/C(=C(/[H])[S]2)C=3C([H])=C([H])C([Cl])=C([H])C=3[H] | Moderate | 30 |
| **ML-266** | [H]C=2C(=NC1=NC(=NN1C=2N([H])C3=C([H])C([H])=C(C([H])=C3[H])N([H])[H])[S]C([H])([H])C4=C([H])C([H])=C([H])C([H])=C4[H])C([H])([H])[H] | Moderate | 19 |
| **ML-267** | [H]C=2C(=NC1=NC([H])=NN1C=2N([H])N([H])[H])C([H])([H])[H] | Moderate | 31 |
| **ML-268** | [Cl]C=1C([H])=C([H])C(=C([H])C=1[H])C=2N=C([S]C=2[H])N([H])[S](=O)(=O)C=3[S]C([H])=C([H])C=3[H] | Moderate | 30 |
| **ML-269** | [Cl]C=1C([H])=C([H])C(=C([H])C=1[H])C=2N=C([S]C=2[H])N([H])[S](=O)(=O)C=3C([H])=NC([H])=C([Br])C=3[H] | Moderate | 30 |
| **ML-270** | O=[S](=O)(C2=C([H])C([H])=C([H])C(C=1N(/N=C(/[H])C=1[H])C([H])([H])[H])=C2[H])N([H])C3=N/C(=C(/[H])[S]3)C=4C([H])=C([H])C([Cl])=C([H])C=4[H] | Moderate | 30 |
| **ML-271** | O=[S](=O)(C2=C([H])C([H])=C([H])C(C=1N=C(/N=C(/[H])C=1[H])C([H])([H])[H])=C2[H])N([H])C3=N/C(=C(/[H])[S]3)C=4C([H])=C([H])C([Cl])=C([H])C=4[H] | Moderate | 30 |

**Table SM10**: ML Training list compounds 

| Compound ID | SMILES | Activity class | Source |
|---|---|---|---|
| **ML-272** | [Cl]C=1C([H])=C([H])C([H])=C(C=1[H])[S](=O)(=O)N([H])C2=N/C(=C(/[H])[S]2)C=3C([H])=C([H])C([H])=C([H])C=3[H] | Moderate | 30 |
| **ML-273** | [Cl]C=1C([H])=C([H])C([H])=C(C=1[H])[S](=O)(=O)N([H])C2=N/C(=C(/[H])[S]2)C=3C([H])=C([H])C([H])=C([H])C=3[Cl] | Moderate | 30 |
| **ML-274** | [Cl]C=1C([H])=C([H])C([H])=C(C=1[H])C=2N=C([S]C=2[H])N([H])[S](=O)(=O)C=3C([H])=C([H])C([H])=C([Cl])C=3[H] | Moderate | 30 |
| **ML-275** | [H]C=2C(=NC1=NC(=NN1C=2N([H])C3=C([H])C([H])=C([H])C([H])=C3[H])[S]C([H])([H])C4=NC=5C(N4[H])=C([H])C([H])=C([H])C=5[H])C([H])([H])[H] | Moderate | 19 |
| **ML-276** | [H]C=2C(=NC1=NC(=NN1C=2N(C([H])([H])C([H])([H])N([H])C([H])([H])[H])C([H])([H])[H])[S]C([H])([H])C3=C([H])C([H])=C([H])C([H])=C3[H])C([H])([H])[H] | Moderate | 31 |
| **ML-277** | [Cl]C=1C([H])=C([H])C([H])=C(C=1[H])[S](=O)(=O)N([H])C2=N/C(=C(/[H])[S]2)C=3C([H])=C([H])C([H])=C([Br])C=3[H] | Moderate | 30 |
| **ML-278** | [Cl]C=1C([H])=C([H])C([H])=C(C=1[H])[S](=O)(=O)N([H])C2=N/C(=C(/[H])[S]2)C=3C([H])=C([H])C([Br])=C([H])C=3[H] | Moderate | 30 |
| **ML-279** | O=[S](=O)(N([H])C=1[S]C([H])=C(N=1)C2=C(OC([H])([H])[H])C([H])=C([H])C([H])=C2[H])C=3C([H])=C([H])C([H])=C([Cl])C=3[H] | Moderate | 30 |
| **ML-280** | O=[S](=O)(N([H])C=1[S]C([H])=C(N=1)C2=C([H])C([H])=C([H])C(OC([H])([H])[H])=C2[H])C=3C([H])=C([H])C([H])=C([Cl])C=3[H] | Moderate | 30 |
| **ML-281** | O=[S](=O)(N([H])C=1[S]C([H])=C(N=1)C=2C([H])=C([H])C(OC([H])([H])[H])=C([H])C=2[H])C=3C([H])=C([H])C([H])=C([Cl])C=3[H] | Moderate | 30 |

| **Table SM10**: ML Training list compounds | | | |
|---|---|---|---|
| **Compound ID** | **SMILES** | **Activity class** | **Source** |
| **ML-282** | [Cl]C=1C([H])=C([H])C([H])=C(C=1[H])[S](=O)(=O)N([H])C2=N/C(=C(/[H])[S]2)C=4C([H])=C([H])C=3OC([H])([H])C([H])([H])OC=3C=4[H] | Moderate | 30 |
| **ML-283** | O=[S](=O)(N([H])C=1[S]C(=C(N=1)C=2C([H])=C([H])C([H])=C([H])C=2[H])C([H])([H])[H])C=3C([H])=C([H])C([H])=C([Cl])C=3[H] | Moderate | 30 |
| **ML-284** | [Cl]C=1C([H])=C([H])C([H])=C(C=1[H])[S](=O)(=O)N([H])C3=NC(C=2C([H])=C([H])C([H])=C([H])C=2[H])=C([S]3)C=4C([H])=C([H])C([H])=C([H])C=4[H] | Moderate | 30 |
| **ML-285** | [H]C=2N=C1N=C5C(=C(N1N=2)N([H])C3=C([H])C([H])=C(C([H])=C3[H])N4C([H])([H])C([H])([H])N(C([H])([H])C4([H])[H])C([H])([H])[H])C([H])([H])C([H])([H])C5([H])[H] | Moderate | 19 |
| **ML-286** | [H]C=2C(=NC1=NC(=NN1C=2N(C([H])([H])C([H])([H])N(C([H])([H])[H])C([H])([H])[H])C([H])([H])[H])[S]C([H])([H])C3=C([H])C([H])=C([H])C([H])=C3[H])C([H])([H])[H] | Moderate | 31 |
| **ML-287** | O=[S](=O)(N([H])C1=NN=C([S]1)C=2C([H])=C([H])C(=C([H])C=2[H])C([H])([H])[H])C=3C([H])=C([H])C([H])=C([Cl])C=3[H] | Moderate | 30 |
| **ML-288** | O=[S](=O)(C1=C([H])C([H])=C([H])C(=C1[H])C([H])([H])[H])N([H])C2=NN=C([S]2)C=3C([H])=C([H])C([Cl])=C([H])C=3[H] | Moderate | 30 |
| **ML-289** | [Cl]C=1C([H])=C([Cl])C([H])=C(C=1[H])[S](=O)(=O)N([H])C2=NN(C([H])=C2[H])C=3C([H])=C([H])C([H])=C([H])C=3[H] | Moderate | 30 |
| **ML-290** | [F]C([F])([F])C=1C([H])=C([H])C([H])=C(C=1[H])[S](=O)(=O)N([H])C2=NN(C([H])=C2[H])C=3C([H])=C([H])C([Cl])=C([H])C=3[H] | Moderate | 30 |
| **ML-291** | O=[S](=O)(C=1C([H])=C([H])C(=C([H])C=1[H])C([H])([H])[H])N([H])C2=N/C(=C(/[H])[S]2)C=3C([H])=C([H])C([Cl])=C([H])C=3[H] | Moderate | 30 |

| **Table SM10**: ML Training list compounds | | | |
|---|---|---|---|
| **Compound ID** | **SMILES** | **Activity class** | **Source** |
| **ML-292** | [H]C=2C(=NC1=NC(=NN1C=2N([H])C3=C([H])C([H])=C(C([H])=C3[H])N4C([H])([H])C([H])([H])N(C([H])([H])C4([H])[H])C([H])([H])[H])[S]C([H])([H])C5=C([H])C([H])=C([H])C([H])=C5[H])C([H])([H])[H] | Moderate | 19 |
| **ML-293** | O=C(N([H])C1=C([H])C([H])=C([H])C(=C1[H])C([H])([H])OC([H])([H])[H])C=3N(C2=C([S]C([H])=C2[H])C=3[H])C([H])([H])[H] | Moderate | 36 |
| **ML-294** | O=C(N([H])C1=C([H])C([H])=C([H])C(OC([H])([H])[H])=C1[H])C=3N(C2=C([S]C([H])=C2[H])C=3[H])C([H])([H])[H] | Moderate | 36 |
| **ML-295** | O=C(N([H])C1=C([H])C([H])=C([H])C([H])=C1[H])C=3N(C2=C([S]C([H])=C2[H])C=3[H])C([H])([H])[H] | Moderate | 36 |
| **ML-296** | [F]/C1=C(\[H])C([H])=C(C([H])=C1[H])C([H])([H])[S]C=3N=C2N=C(C([H])=C(N2N=3)N([H])C4=C([H])C([H])=C(C([H])=C4[H])N5C([H])([H])C([H])([H])N(C([H])([H])C5([H])[H])C([H])([H])[H])C([H])([H])[H] | Moderate | 19 |
| **ML-297** | [H]C=2C(=NC1=NC(=NN1C=2N3C([H])([H])C([H])([H])C([H])([H])[C@@]([H])(C3([H])[H])[N+]([H])([H])[H])[S]C([H])([H])C4=C([H])C([H])=C([H])C([H])=C4[H])C([H])([H])[H] | Moderate | 31 |
| **ML-298** | [Br]C=1C([H])=C([H])C([H])=C(C=1[H])C([H])([H])[S]C=3N=C2N=C(C([H])=C(N2N=3)N([H])C4=C([H])C([H])=C(C([H])=C4[H])N5C([H])([H])C([H])([H])N(C([H])([H])C5([H])[H])C([H])([H])[H])C([H])([H])[H] | Moderate | 19 |
| **ML-299** | [H]C=2C(=NC1=NC(=NN1C=2N([H])N([H])[H])[S]C([H])([H])C([H])=C([H])[H])C([H])([H])[H] | Moderate | 31 |
| **ML-300** | O=C(N([H])C1=C([H])C([H])=C([H])C([F])=C1[H])C=3N(C2=C([S]C([H])=C2[H])C=3[H])C([H])([H])[H] | Moderate | 36 |
| **ML-301** | O=C(N([H])C1=C([H])C([H])=C([H])C([Cl])=C1[H])C=3N(C2=C([S]C([H])=C2[H])C=3[H])C([H])([H])[H] | Moderate | 36 |

| Table SM10: ML Training list compounds | | | |
|---|---|---|---|
| **Compound ID** | **SMILES** | **Activity class** | **Source** |
| **ML-302** | O=C(N([H])C1=C([H])C([H])=C([H])C([H])=C1[H])C=3N(C2=C(OC([H])=C2[H])C=3[H])C([H])([H])[H] | Moderate | 36 |
| **ML-303** | O=C(N([H])C1=C([H])C([H])=C([H])C(=C1[H])C([H])([H])[S]C([H])([H])[H])C=3N(C2=C([S]C([H])=C2[H])C=3[H])C([H])([H])[H] | Moderate | 36 |
| **ML-304** | O=C(N([H])C1=C([H])C([H])=C([H])C([H])=C1[H])C=3N(C2=C([S]C([Br])=C2[H])C=3[H])C([H])([H])[H] | Moderate | 36 |
| **ML-305** | O=C(N([H])C1=C([H])C([H])=C([H])C([H])=C1[H])C3=C([H])C2=C([S]C([H])=C2[H])N3C([H])([H])[H] | Moderate | 36 |
| **ML-306** | O=C(N([H])C1=C([H])C([H])=C([H])C([H])=C1[H])C3=C([H])C=2/N=C(/[H])[S]C=2N3C([H])([H])[H] | Moderate | 36 |
| **ML-307** | O=C(N([H])C1=C([H])C([H])=C([H])C([H])=C1[H])C=3N(C=2/N=C(/[H])[S]C=2C=3[H])C([H])([H])[H] | Moderate | 36 |
| **ML-308** | O=C(N([H])C1=C([H])C([H])=C([H])C([H])=C1[H])C=3N(C=2/N=C(/[H])N(C=2C=3[H])C([H])([H])[H])C([H])([H])[H] | Moderate | 36 |
| **ML-309** | O=C(N([H])C1=C([H])C([H])=C([H])C([H])=C1[H])C=2N(C3=C(C=2[H])N(C([H])=C3[H])C([H])([H])[H])C([H])([H])[H] | Moderate | 36 |
| **ML-310** | [Cl]/C1=C(\[H])C([H])=C(C([H])=C1[H])C([H])([H])[S]C=3N=C2N=C(C([H])=C(N2N=3)N([H])C4=C([H])C([H])=C(C([H])=C4[H])N5C([H])([H])C([H])([H])N(C([H])([H])C5([H])[H])C([H])([H])[H])C([H])([H])[H] | Moderate | 19 |
| **ML-311** | O=C(N([H])C1=C([H])C([H])=C([H])C([H])=C1[H])C=3N([H])C=2/N=C(/[H])N(C=2C=3[H])C([H])([H])[H] | Moderate | 36 |
| **ML-312** | O=C(N([H])C1=C([H])C([H])=C([H])C([H])=C1[H])C2=C([H])C3=C(N2C([H])([H])[H])N(/N=C3/[H])C([H])([H])[H] | Moderate | 36 |

**Table SM10**: ML Training list compounds

| Compound ID | SMILES | Activity class | Source |
|---|---|---|---|
| **ML-313** | O=C(N([H])C1=C([H])C([H])=C([H])C([H])=C1[H])C=3[S]C2=C([S]C([H])=C2[H])C=3[H] | Moderate | 36 |
| **ML-314** | O=C(N([H])C1=C([H])C([H])=C([H])C([H])=C1[H])C=3N(C2=C([S]/C(=C2/[H])C([H])([H])[H])C=3[H])C([H])([H])[H] | Moderate | 36 |
| **ML-315** | O=C(N([H])C1=C([H])C([H])=C([H])C([H])=C1[H])C=3N(C2=C([S]C([H])=C2C([H])([H])[H])C=3[H])C([H])([H])[H] | Moderate | 36 |
| **ML-316** | O=C(N([H])C1=C([H])C([H])=C([H])C([H])=C1[H])C=3N(C2=C([S]/C(=C2/C([H])([H])[H])C([H])([H])[H])C=3[H])C([H])([H])[H] | Moderate | 36 |
| **ML-317** | O=C(N([H])C1=C([H])C([H])=C([H])C([H])=C1[H])C=3N(C2=C([S]C([H])=C2[H])C=3[Cl])C([H])([H])[H] | Moderate | 36 |
| **ML-318** | O=C(N([H])C1=C([H])C([H])=C([H])C([H])=C1[H])C=3N(C2=C([S]C([H])=C2[H])C=3[I])C([H])([H])[H] | Moderate | 36 |
| **ML-319** | O=C(N([H])C1=C([H])C([H])=C([H])C([H])=C1[H])C=3N(C2=C([S]C([I])=C2[H])C=3[H])C([H])([H])[H] | Moderate | 36 |
| **ML-320** | O=C(N([H])C1=C([H])C([H])=C([H])C([H])=C1[H])C=3N(C2=C([S]C([I])=C2[H])C=3[I])C([H])([H])[H] | Moderate | 36 |
| **ML-321** | [Br]/C1=C(\[H])C([H])=C(C([H])=C1[H])C([H])([H])[S]C=3N=C2N=C(C([H])=C(N2N=3)N([H])C4=C([H])C([H])=C(C([H])=C4[H])N5C([H])([H])C([H])([H])N(C([H])([H])C5([H])[H])C([H])([H])[H])C([H])([H])[H] | Moderate | 19 |
| | | | |

**Table SM10**: ML Training list compounds

| Compound ID | SMILES | Activity class | Source |
|---|---|---|---|
| **ML-322** | [H]C=2C(=NC1=NC(=NN1C=2N([H])N([H])[H])[S]C([H])([H])C([H])([H])C([H])([H])[H])C([H])([H])[H] | Moderate | 31 |
| **ML-323** | O=C(N([H])C1=C([H])C([H])=C([H])C([H])=C1[H])C=3N(C2=C([S]C([F])=C2[H])C=3[H])C([H])([H])[H] | Moderate | 36 |
| **ML-324** | O=C(N([H])C1=C([H])C([H])=C([H])C([H])=C1[H])C=3N(C2=C([S]C([H])=C2[H])C=3C([H])([H])OC([H])([H])[H])C([H])([H])[H] | Moderate | 36 |
| **ML-325** | O=C(N([H])C1=C([H])C([H])=C([H])C([H])=C1[H])C=3N(C2=C([S]C([H])=C2[H])C=3C([H])([H])[H])C([H])([H])[H] | Moderate | 36 |
| **ML-326** | O=C(N([H])C1=C([H])C([H])=C([H])C([H])=C1[H])C=3N(C2=C([S]C([H])=C2[H])C=3C([H])([H])N(C([H])([H])[H])C([H])([H])[H])C([H])([H])[H] | Moderate | 36 |
| **ML-327** | O=C(N([H])C1=C([H])C([H])=C([H])C([H])=C1[H])C=3N(C2=C([S]/C(=C2/[H])C([H])([H])N(C([H])([H])[H])C([H])([H])[H])C=3[H])C([H])([H])[H] | Moderate | 36 |
| **ML-328** | O=C(N([H])C1=C([H])C([H])=C([H])C([H])=C1[H])C=3N(C2=C([S]C([H])=C2[H])C=3[H])C([H])([H])C([H])([H])[H] | Moderate | 36 |
| **ML-329** | O=C(N([H])C1=C([H])C([H])=C([H])C([H])=C1[H])C=3N(C2=C([S]C([H])=C2[H])C=3[H])C([H])([H])C([H])([H])C([H])([H])[H] | Moderate | 36 |
| **ML-330** | O=C(N([H])C1=C([H])C([H])=C([H])C([H])=C1[H])C=3N(C2=C([S]C([H])=C2[H])C=3[H])C4=C([H])C([H])=C([H])C([H])=C4[H] | Moderate | 36 |
| **ML-331** | O=C(N([H])C1=C([H])C([H])=C([H])C([H])=C1[H])C=3N([H])C2=C([S]C([H])=C2[H])C=3[H] | Moderate | 36 |
| **ML-332** | O=C(N([H])C1=C([H])C([H])=C([H])N=C1[H])C=3N(C2=C([S]C([H])=C2[H])C=3[H])C([H])([H])[H] | Moderate | 36 |

**Table SM10**: ML Training list compounds

| Compound ID | SMILES | Activity class | Source |
|---|---|---|---|
| **ML-333** | [Br]C=1/C(=C(/[H])C([H])=C([H])C=1[H])C([H])([H])[S]C=3N=C2N=C(C([H])=C(N2N=3)N([H])C4=C([H])C([H])=C(C([H])=C4[H])N5C([H])([H])C([H])([H])N(C([H])([H])C5([H])[H])C([H])([H])[H])C([H])([H])[H] | Moderate | 19 |
| **ML-334** | O=C(N([H])C=1/N=C(/[H])C([H])=C([H])C=1[H])C=3N(C2=C([S]C([H])=C2[H])C=3[H])C([H])([H])[H] | Moderate | 36 |
| **ML-335** | O=C(N([H])C1=C([H])C([H])=NC([H])=C1[H])C=3N(C2=C([S]C([H])=C2[H])C=3[H])C([H])([H])[H] | Moderate | 36 |
| **ML-336** | O=C(N([H])C1=NC([H])=C([H])C([H])=N1)C=3N(C2=C([S]C([H])=C2[H])C=3[H])C([H])([H])[H] | Moderate | 36 |
| **ML-337** | O=C(N([H])C=1/N=C(/[H])N=C([H])C=1[H])C=3N(C2=C([S]C([H])=C2[H])C=3[H])C([H])([H])[H] | Moderate | 36 |
| **ML-338** | O=C(N([H])C1=C([H])C([H])=C([H])C(=C1[H])N2C([H])([H])C([H])([H])N(C([H])([H])C2([H])[H])C([H])([H])[H])C=4N(C3=C([S]C([H])=C3[H])C=4[H])C([H])([H])[H] | Moderate | 36 |
| **ML-339** | O=C(N([H])C1=C([H])C([H])=C([H])C(=C1[H])N(C([H])([H])[H])C([H])([H])[H])C=3N(C2=C([S]C([H])=C2[H])C=3[H])C([H])([H])[H] | Moderate | 36 |
| **ML-340** | O=C(N([H])C1=C([H])C([H])=C([H])C(=C1[H])C(=O)N([H])[H])C=3N(C2=C([S]C([H])=C2[H])C=3[H])C([H])([H])[H] | Moderate | 36 |
| **ML-341** | O=C(N([H])C1=C([H])C([H])=C([H])C([Br])=C1[H])C=3N(C2=C([S]C([H])=C2[H])C=3[H])C([H])([H])[H] | Moderate | 36 |
| **ML-342** | O=C(N([H])C1=C([H])C([H])=C([H])C(=C1[H])C([H])([H])N2C([H])([H])C([H])([H])OC([H])([H])C2([H])[H])C=4N(C3=C([S]C([H])=C3[H])C=4[H])C([H])([H])[H] | Moderate | 36 |
| **ML-343** | O=C2N([H])C(=O)N(C=1/N=C(/[S][H])N(C=12)C([H])([H])C([H])([H])C([H])([H])[H])C([H])([H])[H] | Moderate | 32 |

| **Table SM10**: ML Training list compounds | | | |
|---|---|---|---|
| **Compound ID** | **SMILES** | **Activity class** | **Source** |
| **ML-344** | O=C(N([H])C1=C([H])C([H])=C([H])C(=C1[H])C([H])([H])N(C([H])([H])[H])C([H])([H])[H])C=3N(C2=C([S]C([H])=C2[H])C=3[H])C([H])([H])[H] | Moderate | 36 |
| **ML-345** | O=C(N([H])C1=C([H])C([H])=C([H])C(=C1[H])C([H])([H])O/C2=C(\[H])C([H])=C(O[H])C([H])=C2[H])C=4N(C3=C([S]C([H])=C3[H])C=4[H])C([H])([H])[H] | Moderate | 36 |
| **ML-346** | O=C(N([H])C1=C([H])C([H])=C([H])C(=C1[H])C([H])([H])O[H])C=3N(C2=C([S]C([H])=C2[H])C=3[H])C([H])([H])[H] | Moderate | 36 |
| **ML-347** | O=C(N([H])C1=C([H])C([H])=C([H])C(=C1[H])C([H])([H])O/C2=C(\[H])C([H])=C([H])C([H])=C2[H])C=4N(C3=C([S]C([H])=C3[H])C=4[H])C([H])([H])[H] | Moderate | 36 |
| **ML-348** | O=C(N([H])C1=C([H])C([H])=C([H])C(=C1[H])C([H])([H])O/C2=C(\[H])C([H])=NC([H])=C2[H])C=4N(C3=C([S]C([H])=C3[H])C=4[H])C([H])([H])[H] | Moderate | 36 |
| **ML-349** | O=C(N([H])C2=C([H])C([H])=C([H])C(O/C1=C(\[H])C([H])=NC([H])=C1[H])=C2[H])C=4N(C3=C([S]C([H])=C3[H])C=4[H])C([H])([H])[H] | Moderate | 36 |
| **ML-350** | O=C(N([H])C1=C([H])C([H])=C([H])C([H])=C1[H])C3=C([H])C2=C([S]C([H])=C2[H])C([H])=C3[H] | Moderate | 36 |
| **ML-351** | O=C(N([H])C1=C([H])C([H])=C([H])C([H])=C1[H])C3=NC=2[S]C([H])=C([H])C=2N3C([H])([H])[H] | Moderate | 36 |
| **ML-352** | O=C3N([H])C(=O)N(C=2/N=C(/[S]C([H])([H])C1=C([H])C([H])=C([H])C(=C1[H])C([H])([H])[H])N(C=23)C([H])([H])C([H])([H])C([H])([H])[H])C([H])([H])[H] | Moderate | 32 |

**Table SM10**: ML Training list compounds

| Compound ID | SMILES | Activity class | Source |
|---|---|---|---|
| **ML-353** | O=C(N([H])C1=C([H])C([H])=C([H])C([H])=C1[H])C=3N(C2=C([S]C([H])=C2[H])C=3[H])C([H])([H])C([H])([H])N([H])[H] | Moderate | 36 |
| **ML-354** | O=C(/C2=C(\[H])C=1[S]C([H])=C([H])C=1N2C([H])([H])[H])N([H])C3=C([H])C([H])=C(C([H])=C3[H])C([H])([H])O/C4=C(\[H])C([H])=NC([H])=C4[H] | Moderate | 24 |
| **ML-355** | O=C(/C2=C(\[H])C=1[S]C([H])=C([H])C=1N2C([H])([H])[H])N([H])C4=C(OC([H])([H])C3=C([H])C([H])=NC([H])=C3[H])C([H])=C([H])C([H])=C4[H] | Moderate | 24 |
| **ML-356** | O=C(/C2=C(\[H])C=1[S]C([H])=C([H])C=1N2C([H])([H])[H])N([H])C4=C([H])C([H])=C(OC([H])([H])C3=C([H])C([H])=NC([H])=C3[H])C([H])=C4[H] | Moderate | 24 |
| **ML-357** | O=C(/C2=C(\[H])C=1[S]C([H])=C([H])C=1N2C([H])([H])[H])N([H])C3=C(C([H])=C([H])C([H])=C3[H])C([H])([H])O/C6=C(\[H])C([H])=C(OC4([H])C([H])([H])C([H])([H])N(C([H])([H])C4([H])[H])C([H])([H])C5=C([H])C([H])=C([H])C([H])=C5[H])C([H])=C6[H] | Moderate | 24 |
| **ML-358** | O=C3N([H])C(=O)N(C=2/N=C(/[S]C([H])([H])C1=C([H])C([H])=C([Cl])C([H])=C1[H])N(C=23)C([H])([H])C([H])([H])C([H])([H])[H])C([H])([H])[H] | Moderate | 32 |
| **ML-359** | O=C(/C2=C(\[H])C=1[S]C([H])=C([H])C=1N2C([H])([H])[H])N([H])C3=C(C([H])=C([H])C([H])=C3[H])C([H])([H])OC([H])([H])[H] | Moderate | 24 |
| **ML-360** | O=C3N([H])C(=O)N(C=2/N=C(/[S]C([H])([H])C1=C([H])C([H])=C([H])C([F])=C1[H])N(C=23)C([H])([H])C([H])([H])C([H])([H])[H])C([H])([H])[H] | Moderate | 32 |
| **ML-361** | O=C(/C2=C(\[H])C=1[S]C([H])=C([H])C=1N2C([H])([H])[H])N([H])C3=C([H])C([H])=C(C([H])=C3[H])C([H])([H])OC([H])([H])[H] | Moderate | 24 |
| **ML-362** | O=C3N([H])C(=O)N(C=2/N=C(/[S]C([H])([H])C1=C([H])C([H])=C([F])C([H])=C1[H])N(C=23)C([H])([H])C([H])([H])C([H])([H])[H])C([H])([H])[H] | Moderate | 32 |

**Table SM10**: ML Training list compounds

| Compound ID | SMILES | Activity class | Source |
|---|---|---|---|
| **ML-363** | O=C(/C2=C(\[H])C=1[S]C([H])=C([H])C=1N2C([H])([H])[H])N([H])C3=C(OC([H])([H])[H])C([H])=C([H])C([H])=C3[H] | Moderate | 24 |
| **ML-364** | O=C(/C2=C(\[H])C=1[S]C([H])=C([H])C=1N2C([H])([H])[H])N([H])C3=C([H])C([H])=C(OC([H])([H])[H])C([H])=C3[H] | Moderate | 24 |
| **ML-365** | O=C(/C2=C(\[H])C=1[S]C([H])=C([H])C=1N2C([H])([H])[H])N([H])C3=C(C([H])=C([H])C([H])=C3[H])C([H])([H])O/C4=C(\[H])C([H])=NC([H])=C4[H] | Moderate | 24 |
| **ML-366** | O=[N+]([O-])C1=C([H])C([H])=C(C([H])=C1[H])C([H])([H])[S]C=3N=C2N=C(C([H])=C(N2N=3)N([H])C4=C([H])C([H])=C(C([H])=C4[H])N5C([H])([H])C([H])([H])N(C([H])([H])C5([H])[H])C([H])([H])[H])C([H])([H])[H] | Moderate | 19 |
| **ML-367** | [H]C=1/C(=C(/O[H])C([H])=C([H])C=1C([H])=C([H])C2=C([H])C([H])=C(C([H])=C2[H])N([H])[H])C3=NC([H])=C([H])C([H])=N3 | Moderate | 29 |
| **ML-368** | O=[N+]([O-])C=1/C(=C(/[H])C([H])=C([H])C=1[H])C([H])([H])[S]C=3N=C2N=C(C([H])=C(N2N=3)N([H])C4=C([H])C([H])=C(C([H])=C4[H])N5C([H])([H])C([H])([H])N(C([H])([H])C5([H])[H])C([H])([H])[H])C([H])([H])[H] | Moderate | 19 |
| **ML-369** | [H]C=2C(=NC1=NC(=NN1C=2N([H])N([H])[H])[S]C([H])([H])C3([H])C([H])([H])C3([H])[H])C([H])([H])[H] | Moderate | 31 |
| **ML-370** | [F]C=3C([H])=C(/C1=N/C([H])=C([H])C(=C1[H])C([H])=C([H])C2=C([H])C([H])=C(C([H])=C2[H])N([H])[H])C(O[H])=C([H])C=3[H] | Moderate | 29 |

**Table SM10**: ML Training list compounds

| Compound ID | SMILES | Activity class | Source |
|---|---|---|---|
| **ML-371** | [F]C=3C([H])=C([H])C(O[H])=C(C=1/N=C(/[H])C([H])=C(C=1[H])C([H])=C([H])C2=C([H])C(=C([H])C([H])=C2[H])N([H])[H])C=3[H] | Moderate | 29 |
| **ML-372** | O=C(N3C([H])([H])C([H])([H])C([H])(C2=N/N=C(/C1=C(\[H])C([H])=C([H])C([H])=C1[Cl])C2([H])[H])C([H])([H])C3([H])[H])C([H])([H])C([H])([H])N4/N=C(/[H])N=C4[H] | Moderate | 31 |
| **ML-373** | O=C(/C1=C(\[H])C([H])=C(/N=C1/[H])N4C([H])([H])C([H])([H])C([H])(C([H])([H])C2=NN=C(N2/C3=C(\[H])C([H])=C([H])C([H])=C3[H])C([H])([H])O[H])C([H])([H])C4([H])[H])N([H])[H] | Moderate | 31 |
| **ML-374** | O=C4C([F])=C(/N=C(/C1=C(\[H])C([H])=C(C([H])=C1[H])C([H])([H])N(C([H])([H])C2=C3/C(=C(/[H])C([H])=C2[H])C([H])=NC([H])=C3[H])C([H])([H])[H])N4[H])C([H])([H])[H] | Moderate | 31 |
| **ML-375** | [H]C=2C(=NC1=NC(=NN1C=2N([H])C3=C([H])C([H])=C(C([H])=C3[H])N4C([H])([H])C([H])([H])N(C([H])([H])C4([H])[H])C([H])([H])[H])[S]C([H])([H])C5=C([H])C([H])=C(OC([H])([H])[H])C([H])=C5[H])C([H])([H])[H] | Moderate | 19 |
| **ML-376** | [H]C=2C(=NC1=NC(=NN1C=2N([H])N([H])[H])[S]C([H])([H])C3=C4/C(=C(/[H])C([H])=C3[H])C([H])=C([H])C([H])=C4[H])C([H])([H])[H] | Moderate | 31 |
| **ML-377** | [Br]/C1=C(\[H])C([H])=C2/C(=C1/[H])C3=C(C(=O)C2=O)N(/C(=C3/C(=O)OC([H])([H])C([H])([H])[H])C([H])([H])O[H])C([H])([H])[H] | Moderate | 31 |
| **ML-378** | O=C2C=1/C(=C(/[H])C([H])=C(C=1[H])[N+](=O)[O-])N(C2=O)C([H])([H])N3C([H])([H])C([H])([H])C([H])(C([H])([H])C3([H])[H])C([H])([H])C4=C([H])C([H])=C([H])C([H])=C4[H] | Moderate | 31 |

**Table SM10**: ML Training list compounds

| Compound ID | SMILES | Activity class | Source |
|---|---|---|---|
| **ML-379** | [H]/C1=C(\[H])C([H])=C2/C(=C1/[H])N([H])C(=C2[C@@]([H])(C=3O/C(=C(/[H])C=3[H])C([H])([H])[H])C=4C5=C(N([H])C=4C([H])([H])[H])C([H])=C([H])C([H])=C5[H])C([H])([H])[H] | Moderate | 31 |
| **ML-380** | [Cl]/C1=C(\[H])C([H])=C(C([H])=C1[H])[C@]3([H])C(=NN(/C2=C(\[H])C([H])=C([H])C([H])=C2[H])C3([H])[H])C=4OC([H])=C([H])C=4[H] | Moderate | 31 |
| **ML-381** | O=C(C=2C1=C(C([H])=C([H])C([H])=C1[H])N([H])C=2[H])C([H])([H])C([H])([H])C(=O)N([H])C3=C([H])C([H])=C(C([H])=C3[H])C([H])([H])[H] | Moderate | 31 |
| **ML-382** | O=C(N([H])[H])N([H])N([H])C=2/N=C(/[S]C([H])([H])C#C[H])N=C(C=1C([H])=C([H])C([F])=C([F])C=1[H])C=2C#N | Moderate | 18 |
| **ML-383** | [Cl]/C1=C(\[H])C([H])=C(C([H])=C1[F])C([H])([H])[S]C=3N=C2N=C(C([H])=C(N2N=3)N([H])C4=C([H])C([H])=C(C([H])=C4[H])N5C([H])([H])C([H])([H])N(C([H])([H])C5([H])[H])C([H])([H])[H])C([H])([H])[H] | Moderate | 19 |
| **ML-384** | O=C(N([H])[H])N([H])N([H])C=2/N=C(/[S]C([H])([H])C#C[H])N=C(C=1C([H])=C([H])C(=C([H])C=1[H])[N+](=O)[O-])C=2C#N | Moderate | 18 |
| **ML-385** | O=C(N([H])[H])N([H])N([H])C=2/N=C(/[S]C([H])([H])C#C[H])N=C(C=1C([H])=C([H])C([Br])=C([H])C=1[H])C=2C#N | Moderate | 18 |
| **ML-386** | O=C1N(C(=O)N([H])C(=O)C1=C([H])C2=C([H])C([H])=C([H])C(O[H])=C2[H])C3=C([H])C([H])=C(C([H])=C3[H])C([H])([H])[H] | Moderate | 26 |
| **ML-387** | [Cl]C=2/N=C(/[S]C([H])([H])C#C[H])N=C(C=1C([H])=C([H])C([H])=C([H])C=1[H])C=2C#N | Moderate | 18 |

**Table SM10**: ML Training list compounds

| Compound ID | SMILES | Activity class | Source |
|---|---|---|---|
| **ML-388** | [Cl]/C2=N/C(=NC(C=1C([H])=C([H])C(=C([H])C=1[H])C([H])([H])[H])=C2C#N)[S]C([H])([H])C#C[H] | Moderate | 18 |
| **ML-389** | [Cl]/C2=N/C(=NC(C=1C([H])=C([H])C(=C([H])C=1[H])C([H])(C([H])([H])[H])C([H])([H])[H])=C2C#N)[S]C([H])([H])C#C[H] | Moderate | 18 |
| **ML-390** | [F]C=1C([H])=C([H])C(=C([H])C=1[H])C=2/N=C(/[S]C([H])([H])C#C[H])N=C([Cl])C=2C#N | Moderate | 18 |
| **ML-391** | [Cl]C=1C([H])=C([H])C(=C([H])C=1[H])C=2/N=C(/[S]C([H])([H])C#C[H])N=C([Cl])C=2C#N | Moderate | 18 |
| **ML-392** | O=C1N(C(=[S])N([H])C(=O)[C@]1([H])C([H])([H])C2=C([H])C([H])=C(OC([H])([H])[H])C(OC([H])([H])[H])=C2[H])C3=C([H])C([H])=C([Br])C([H])=C3[H] | Moderate | 26 |
| **ML-393** | [F]C=1C([H])=C([H])C(=C([H])C=1[F])C=2/N=C(/[S]C([H])([H])C#C[H])N=C([Cl])C=2C#N | Moderate | 18 |
| **ML-394** | O=C1N(C(=O)N([H])C(=O)C1=C([H])C2=C([H])C=3C(N2[H])=C([H])C([H])=C([H])C=3[H])C4=C([H])C([H])=C(C([H])=C4[H])C([H])([H])C([H])([H])[H] | Moderate | 26 |
| **ML-395** | [F]/C1=C(\[H])C([H])=C(C([H])=C1[Cl])C([H])([H])[S]C=3N=C2N=C(C([H])=C(N2N=3)N([H])C4=C([H])C([H])=C(C([H])=C4[H])N5C([H])([H])C([H])([H])N(C([H])([H])C5([H])[H])C([H])([H])[H])C([H])([H])[H] | Moderate | 19 |

| Table SM10: ML Training list compounds | | | |
|---|---|---|---|
| **Compound ID** | **SMILES** | **Activity class** | **Source** |
| **ML-396** | [Cl]C=2/N=C(/[S]C([H])([H])C#C[H])N=C(C=1C([H])=C([H])C([Br])=C([H])C=1[H])C=2C#N | Moderate | 18 |
| **ML-397** | O=C1N(C(=O)N([H])C(=O)C1=C([H])C2=C([H])C([F])=C(C([H])=C2[H])N3C([H])([H])C([H])([H])OC([H])([H])C3([H])[H])C4=C([H])C([H])=C(OC([H])([H])[H])C([H])=C4[H] | Moderate | 26 |
| **ML-398** | O=C(N([H])C1=C([H])C([H])=C([H])C(=C1C([H])([H])O/C3=C(\[H])C([H])=C(OC2([H])C([H])([H])C([H])([H])N([H])C([H])([H])C2([H])[H])C([H])=C3[H])C([H])([H])OC([H])([H])[H])C=4N([H])C=5C(C=4[H])=C([H])C([H])=C([H])C=5[H] | Moderate | 26 |
| **ML-399** | O=C(N([H])C1=C([H])C([H])=C([H])C(=C1C([H])([H])O/C3=C(\[H])C([H])=C(OC2([H])C([H])([H])C([H])([H])N([H])C([H])([H])C2([H])[H])C([H])=C3[H])C([H])([H])OC([H])([H])[H])C=4[S]C([H])=C([H])C=4[H] | Moderate | 26 |
| **ML-400** | [Cl]C=1/C(=C(/[Cl])C([H])=C([H])C=1[H])C([H])([H])[S]C=3N=C2N=C(C([H])=C(N2N=3)N([H])C4=C([H])C([H])=C(C([H])=C4[H])N5C([H])([H])C([H])([H])N(C([H])([H])C5([H])[H])C([H])([H])[H])C([H])([H])[H] | Moderate | 19 |
| **ML-401** | [F]/C1=C(\[H])C([H])=C(C([H])=C1[H])C([H])([H])[S]C=3N=C2N=C(C([H])=C(N2N=3)N([H])N([H])[H])C([H])([H])[H] | Moderate | 31 |
| **ML-402** | [H]C=2C(=NC1=NC(=NN1C=2N([H])C3=C([H])C([H])=C(C([H])=C3[H])N4C([H])([H])C([H])([H])N(C([H])([H])C4([H])[H])C([H])([H])[H])[S]C([H])([H])C5=C([H])C([H])=C(C([H])=C5[H])C([H])([H])[H])C([H])([H])[H] | Moderate | 19 |
| **CHEMBL1089** | [H]N([H])N([H])C([H])([H])C([H])([H])C=1C([H])=C([H])C([H])=C([H])C=1[H] | Moderate | ChEMBL database |
| **CHEMBL1607608** | O=[S](=O)(N([H])C1=C([H])C(OC([H])([H])[H])=C(C([H])=C1[H])N2N=C(C([H])=C2C([H])([H])[H])C([H])([H])[H])C=3C([H])=C([H])C([H])=C([H])C=3[H] | Moderate | ChEMBL database |

**Table SM10**: ML Training list compounds

| Compound ID | SMILES | Activity class | Source |
|---|---|---|---|
| **CHEMBL165** | [H]OC=2C([H])=C([H])C(C([H])=C([H])C=1C([H])=C(O[H])C([H])=C(O[H])C=1[H])=C([H])C=2[H] | Moderate | ChEMBL database |
| **CHEMBL2181950** | O=C(N([H])C=1C([H])=C([H])C([H])=C([H])C=1[H])C([H])([H])C(=NO[H])N([H])[H] | Moderate | ChEMBL database |
| **CHEMBL3108956** | O=C(OC([H])([H])[H])C=1C([H])=C([H])N=C(C=1[H])C2=NC([H])=C([H])C(=C2[H])C(=O)O[H] | Moderate | ChEMBL database |
| **CHEMBL334644** | [H]C([H])([H])[C@@]3([H])N([H])C1=NC(=NN1[C@@]([H])(N2C([H])([H])C([H])([H])OC([H])([H])C2([H])[H])C3([H])[H])[S]C([H])([H])C=4C([H])=C([H])C([H])=C([H])C=4[H] | Moderate | ChEMBL database |
| **CHEMBL3785551** | [Cl]/C2=C(\C1=N\C(=C(O1)C([H])([H])[H])C([H])([H])C([H])([H])[S]C([H])([H])[H])C([H])=C(N([H])[H])C([H])=C2[H] | Moderate | ChEMBL database |
| **CHEMBL4060656** | [H]N1C(=N/C2=C(\[H])C([H])=C([H])C([H])=C12)C([H])([H])[S]C=5N=C4N([H])[C@]([H])(C([H])([H])[C@@]([H])(N([H])C=3C([H])=C([H])C(=C([H])C=3[H])C([H])([H])[H])N4N=5)C([H])([H])[H] | Moderate | ChEMBL database |
| **CHEMBL4064818** | [H]N4C([H])([H])C([H])([H])N([C@]3([H])N2/N=C(/[S]C([H])([H])C=1C([H])=C([H])C([H])=C([H])C=1[H])N=C2N([H])[C@]([H])(C([H])([H])[H])C3([H])[H])C([H])([H])C4([H])[H] | Moderate | ChEMBL database |
| **CHEMBL4066293** | [H][C@@]2(C=1C([H])=C([H])C(OC([H])([H])[H])=C([H])C=1[H])C(=O)N([C@@]2([H])C=3C([H])=C([H])C(C#N)=C([H])C=3[H])[C@@]4([H])C([H])([H])C([H])([H])[C@]([H])(N([H])[H])C([H])([H])C4([H])[H] | Moderate | ChEMBL database |
| **CHEMBL4067124** | [H]N(C=1C([H])=C([H])C([H])=C([H])C=1[H])[C@]4([H])N3/N=C(/[S]C([H])([H])C=2C([H])=C([H])C([H])=C([H])C=2[H])N=C3N([H])[C@@]([H])(C([H])([H])[H])C4([H])[H] | Moderate | ChEMBL database |
| **CHEMBL4070519** | O=C(C=5C([H])=C([H])C(N([H])[C@]4([H])N3/N=C(/[S]C([H])([H])C2=N/C1=C(\[H])C([H])=C([H])C([H])=C1N2[H])N=C3N([H])[C@]([H])(C([H])([H])[H])C4([H])[H])=C([H])C=5[H])C([H])=C([H])C=6[S]C([H])=C([H])C=6[H] | Moderate | ChEMBL database |

**Table SM10**: ML Training list compounds

| Compound ID | SMILES | Activity class | Source |
|---|---|---|---|
| **CHEMBL4073417** | O=C(O[H])C=4C([H])=C([H])C(N([H])[C@@]3([H])N2/N=C(/[S]C([H])([H])C=1C([H])=C([H])C([H])=C([H])C=1[H])N=C2N([H])[C@@]([H])(C([H])([H])[H])C3([H])[H])=C([H])C=4[H] | Moderate | ChEMBL database |
| **CHEMBL4082098** | [H]N(C=1C([H])=C([H])C(=C([H])C=1[H])C([H])([H])[H])[C@]3([H])N2N=C(N=C2N([H])[C@@]([H])(C3([H])[H])C([H])([H])[H])[S]C([H])([H])C=4C([H])=C([H])C([H])=C([H])C=4[H] | Moderate | ChEMBL database |
| **CHEMBL4082527** | [F]C=5C([H])=C([H])C(N([H])[C@@]4([H])N3/N=C(/[S]C([H])([H])C2=N/C1=C(\[H])C([H])=C([H])C([H])=C1N2[H])N=C3N([H])[C@@]([H])(C([H])([H])[H])C4([H])[H])=C([H])C=5[H] | Moderate | ChEMBL database |
| **CHEMBL4083572** | [H]N1N=C(C([H])=C1C(=O)N4C([H])([H])C([H])([H])[C@@]([H])(C2=N/C(=C(/[H])C(=C2[H])C=3C([H])=NN(C=3[H])C([H])([H])[H])C([H])([H])[H])C4([H])[H])C([H])(C([H])([H])[H])C([H])([H])[H] | Moderate | ChEMBL database |
| **CHEMBL4083797** | [F]C=4C([H])=C([H])C(N([H])[C@]3([H])N2/N=C(/[S]C([H])([H])C=1C([H])=C([H])C([H])=C([H])C=1[H])N=C2N([H])[C@]([H])(C([H])([H])[H])C3([H])[H])=C([H])C=4[H] | Moderate | ChEMBL database |
| **CHEMBL4085006** | [H][C@@]3(C=1C([H])=C([H])C(OC([H])([H])[H])=NC=1[H])C([H])([H])N(C(=O)C([H])([H])[C@@]2([H])C([H])([H])C([H])([H])C([H])([H])N([H])C2([H])[H])C([H])([H])[C@@]3([H])C=4C([H])=C([H])C(C#N)=C([H])C=4[H] | Moderate | ChEMBL database |
| **CHEMBL4087815** | [H]C([H])([H])[C@@]4([H])N([H])C1=NC(=NN1[C@@]([H])(N3C([H])([H])C([H])([H])[C@@]([H])(N2C([H])([H])C([H])([H])C([H])([H])C([H])([H])C2([H])[H])C([H])([H])C3([H])[H])C4([H])[H])[S]C([H])([H])C=5C([H])=C([H])C([H])=C([H])C=5[H] | Moderate | ChEMBL database |

| **Table SM10**: ML Training list compounds | | | |
|---|---|---|---|
| **Compound ID** | **SMILES** | **Activity class** | **Source** |
| **CHEMBL4090367** | [H]N1C([H])=C(C2=C1C([H])=C([H])C([H])=C2[H])C([H])([H])C([H])([H])N([H])[C@]5([H])N4/N=C(/[S]C([H])([H])C=3C([H])=C([H])C([H])=C([H])C=3[H])N=C4N([H])[C@]([H])(C([H])([H])[H])C5([H])[H] | Moderate | ChEMBL database |
| **CHEMBL4091439** | O=C(C([H])=C([H])C1=C([H])C(OC([H])([H])[H])=C(OC([H])([H])[H])C(OC([H])([H])[H])=C1[H])C=6C([H])=C([H])C(N([H])[C@]3([H])N2N=C(N=C2N([H])[C@@]([H])(C3([H])[H])C([H])([H])[H])[S]C([H])([H])C5=N/C4=C(\[H])C([H])=C([H])C([H])=C4N5[H])=C([H])C=6[H] | Moderate | ChEMBL database |
| **CHEMBL4099054** | [Cl]C=5C([H])=C([H])C(N([H])[C@]4([H])N3/N=C(/[S]C([H])([H])C2=N/C1=C(\[H])C([H])=C([H])C([H])=C1N2[H])N=C3N([H])[C@]([H])(C([H])([H])[H])C4([H])[H])=C([H])C=5[H] | Moderate | ChEMBL database |
| **CHEMBL4099168** | N#CC=5C([H])=C([H])C(N([H])[C@]4([H])N3/N=C(/[S]C([H])([H])C2=N/C1=C(\[H])C([H])=C([H])C([H])=C1N2[H])N=C3N([H])[C@]([H])(C([H])([H])[H])C4([H])[H])=C([H])C=5[H] | Moderate | ChEMBL database |
| **CHEMBL4101488** | [Br]C=5C([H])=C([H])C(N([H])[C@@]4([H])N3/N=C(/[S]C([H])([H])C2=N/C1=C(\[H])C([H])=C([H])C([H])=C1N2[H])N=C3N([H])[C@@]([H])(C([H])([H])[H])C4([H])[H])=C([H])C=5[H] | Moderate | ChEMBL database |

**Table SM11**: ML Testing list compounds

| Compound ID | SMILES | Activity class | Source |
|---|---|---|---|
| **CHEMBL4101839** | [H]C([H])([H])[C@]3([H])N([H])C1=NC(=NN1[C@@]([H])(OC([H])([H])C([H])([H])N2C([H])([H])C([H])([H])C([H])([H])C2([H])[H])C3([H])[H])[S]C([H])([H])C=4C([H])=C([H])C([H])=C([H])C=4[H] | Moderate | ChEMBL database |
| **CHEMBL4104027** | [H][C@]2(C=1C([H])=C([H])C(=C([H])C=1[H])C([H])([H])[H])C(=O)N([C@@]2([H])C=3C([H])=C([H])C(C#N)=C([H])C=3[H])[C@]4([H])C([H])([H])C([H])([H])[C@@]([H])(N([H])[H])C([H])([H])C4([H])[H] | Moderate | ChEMBL database |
| **ML-403** | O=C(N1C([H])([H])C([H])([H])C([H])([H])[C@@]([H])(N([H])[H])C1([H])[H])C([H])([H])N(/C2=C(\[H])C([H])=C(C#N)C([H])=C2[H])[S](=O)(=O)C3=C([H])C4=C(C([H])=C3[H])N(C(=O)C([H])([H])[H])C([H])([H])C4([H])[H] | Active | 33 |
| **ML-404** | O=C(N1C([H])([H])C([H])([H])C([H])(N([H])[H])C([H])([H])C1([H])[H])C([H])([H])N(/C2=C(\[H])C([H])=C(C#N)C([H])=C2[H])[S](=O)(=O)C3=C([H])C4=C(C([H])=C3[H])N(C(=O)C([H])([H])[H])C([H])([H])C4([H])[H] | Active | 33 |
| **ML-405** | O=C(OC(C([H])([H])[H])(C([H])([H])[H])C([H])([H])[H])N4C([H])([H])C([H])([H])N(C(=[S])[S]C([H])([H])C=1N=NN(C=1[H])C([H])([H])C3=C([H])C(=O)OC=2C([H])=C(O[H])C([H])=C(O[H])C=23)C([H])([H])C4([H])[H] | Active | 27 |
| **ML-406** | O=C(OC(C([H])([H])[H])(C([H])([H])[H])C([H])([H])[H])N4C([H])([H])C([H])([H])N(C(=[S])[S]C([H])([H])C=1N=NN(C=1[H])C([H])([H])C3=C([H])C(=O)OC=2C(O[H])=C(O[H])C([H])=C([H])C=23)C([H])([H])C4([H])[H] | Active | 27 |
| **ML-407** | O=C2OC1=C(C([H])=C([H])C(OC([H])([H])[H])=C1[H])C(=C2[H])C([H])([H])N3N=N/C(=C3/[H])C([H])([H])[S]C(=[S])N4C([H])([H])C([H])([H])N(C(=O)OC(C([H])([H])[H])(C([H])([H])[H])C([H])([H])[H])C([H])([H])C4([H])[H] | Active | 27 |
| **ML-408** | O=C(N1C([H])([H])C([H])([H])C([H])(N([H])[H])C([H])([H])C1([H])[H])C([H])([H])N(/C2=C(\[H])C([H])=C(C#N)C([H])=C2[H])C([H])([H])C3=C([H])C([H])=C(C#N)C([F])=C3[H] | Active | 33 |
| **ML-409** | O=C(N1C([H])([H])C([H])([H])C([H])(N([H])[H])C([H])([H])C1([H])[H])C([H])([H])N(/C2=C(\[H])C([H])=C(C#N)C([H])=C2[H])C([H])([H])C3=C([H])C([H])=C(OC([H])([H])[H])C([F])=C3[H] | Active | 33 |
| **ML-410** | O=C2OC1=C(C([H])=C([H])C(=C1[H])C([H])([H])[H])C(=C2[H])C([H])([H])N3N=N/C(=C3/[H])C([H])([H])[S]C(=[S])N4C([H])([H])C([H])([H])N(C(=O)OC(C([H])([H])[H])(C([H])([H])[H])C([H])([H])[H])C([H])([H])C4([H])[H] | Active | 27 |

| **Table SM11**: ML Testing list compounds | | | |
|---|---|---|---|
| **Compound ID** | **SMILES** | **Activity class** | **Source** |
| **ML-411** | O=C(N1C([H])([H])C([H])([H])C([H])(C([H])([H])C1([H])[H])C([H])([H])N([H])[H])C([H])([H])N(/C2=C(\[H])C([H])=C(C#N)C([H])=C2[H])C([H])([H])C3=C([H])C([H])=C([H])C(=C3[H])C(=O)N([H])[H] | Active | 33 |
| **ML-412** | O=C(N1C([H])([H])C([H])([H])C([H])(C([H])([H])C1([H])[H])C([H])([H])N([H])[H])C([H])([H])N(/C2=C(\[H])C([H])=C(C#N)C([H])=C2[H])C([H])([H])C3=C([H])C([H])=C(C([H])=C3[H])C(=O)N([H])[H] | Active | 33 |
| **ML-413** | O=C(N1C([H])([H])C([H])([H])C([H])(C([H])([H])C1([H])[H])C([H])([H])N([H])[H])C([H])([H])N(/C2=C(\[H])C([H])=C(C#N)C([H])=C2[H])C([H])([H])C3=C([H])C([H])=C(OC([H])([H])[H])C([F])=C3[H] | Active | 33 |
| **ML-414** | O=C(N1C([H])([H])C([H])([H])C([F])(C([H])([H])C1([H])[H])C([H])([H])N([H])[H])C([H])([H])N(/C2=C(\[H])C([H])=C(C#N)C([H])=C2[H])C([H])([H])C3=C([H])C([H])=C(OC([H])([H])[H])C([F])=C3[H] | Active | 33 |
| **ML-415** | N#C/C3=C(\[H])C([H])=C(N1N=C(C([H])=C1OC([H])([H])C(=O)N(C([H])([H])[H])C([H])([H])[H])C(=O)N2C([H])([H])[C@]([H])(N([H])[H])C([H])([H])C([H])([H])C2([H])[H])C([H])=C3[H] | Active | 34 |
| **ML-416** | N#C/C4=C(\[H])C([H])=C(N1N=C(C([H])=C1OC([H])([H])C(=O)N2C([H])([H])C([H])([H])OC([H])([H])C2([H])[H])C(=O)N3C([H])([H])[C@]([H])(N([H])[H])C([H])([H])C([H])([H])C3([H])[H])C([H])=C4[H] | Active | 34 |
| **ML-417** | N#C/C4=C(\[H])C([H])=C(N1N=C(C([H])=C1OC([H])([H])[C@@]2([H])N([H])C(=O)C([H])([H])C2([H])[H])C(=O)N3C([H])([H])[C@]([H])(N([H])[H])C([H])([H])C([H])([H])C3([H])[H])C([H])=C4[H] | Active | 34 |
| **ML-418** | N#C/C3=C(\[H])C([H])=C(N1N=C(C([H])=C1OC([H])([H])[C@@]([H])(O[H])C([H])([H])[H])C(=O)N2C([H])([H])[C@]([H])(N([H])[H])C([H])([H])C([H])([H])C2([H])[H])C([H])=C3[H] | Active | 34 |

**Table SM11**: ML Testing list compounds

| Compound ID | SMILES | Activity class | Source |
|---|---|---|---|
| **ML-419** | N#C/C4=C(\[H])C([H])=C(N1N=C(C([H])=C1OC([H])([H])C2=C([H])C(=NN2C([H])([H])[H])C([H])([H])[H])C(=O)N3C([H])([H])[C@]([H])(N([H])[H])C([H])([H])C([H])([H])C3([H])[H])C([H])=C4[H] | Active | 34 |
| **ML-420** | N#C/C4=C(\[H])C([H])=C(N1N=C(C([H])=C1OC([H])([H])C2=NC([H])=NN2C([H])([H])[H])C(=O)N3C([H])([H])[C@]([H])(N([H])[H])C([H])([H])C([H])([H])C3([H])[H])C([H])=C4[H] | Active | 34 |
| **ML-421** | N#C/C4=C(\[H])C([H])=C(N1N=C(C([H])=C1OC([H])([H])C2=NN(C([H])=N2)C([H])([H])[H])C(=O)N3C([H])([H])[C@]([H])(N([H])[H])C([H])([H])C([H])([H])C3([H])[H])C([H])=C4[H] | Active | 34 |
| **ML-422** | N#C/C4=C(\[H])C([H])=C(N1N=C(C([H])=C1OC([H])([H])C=2N=C(ON=2)C([H])([H])[H])C(=O)N3C([H])([H])[C@]([H])(N([H])[H])C([H])([H])C([H])([H])C3([H])[H])C([H])=C4[H] | Active | 34 |
| **ML-423** | N#C/C4=C(\[H])C([H])=C(N1N=C(C([H])=C1OC([H])([H])C2=NN=C(O2)C([H])([H])[H])C(=O)N3C([H])([H])[C@]([H])(N([H])[H])C([H])([H])C([H])([H])C3([H])[H])C([H])=C4[H] | Active | 34 |
| **ML-424** | N#C/C4=C(\[H])C([H])=C(N1N=C(C([H])=C1OC([H])([H])C=2ON=C(C=2[H])C([H])(C([H])([H])[H])C([H])([H])[H])C(=O)N3C([H])([H])[C@]([H])(N([H])[H])C([H])([H])C([H])([H])C3([H])[H])C([H])=C4[H] | Active | 34 |
| **ML-425** | N#C/C4=C(\[H])C([H])=C(N1N=C(C([H])=C1OC([H])([H])C=2ON=C(C=2[H])C([H])([H])[H])C(=O)N3C([H])([H])[C@@]([H])(N([H])[H])C([H])([H])C([H])([H])C3([H])[H])C([H])=C4[H] | Active | 34 |
| **ML-426** | N#C/C4=C(\[H])C([H])=C(N1N=C(C([H])=C1OC([H])([H])C=2ON=C(C=2[H])C([H])([H])[H])C(=O)N3C([H])([H])[C@]([H])(N([H])C([H])([H])[H])C([H])([H])C([H])([H])C3([H])[H])C([H])=C4[H] | Active | 34 |
| **ML-427** | N#C/C4=C(\[H])C([H])=C(N1N=C(C([H])=C1OC([H])([H])C=2ON=C(C=2[H])C([H])([H])[H])C(=O)N3C([H])([H])[C@@]([H])(N(C([H])([H])[H])C([H])([H])[H])C([H])([H])C([H])([H])C3([H])[H])C([H])=C4[H] | Active | 34 |

| Table SM11: ML Testing list compounds | | | |
|---|---|---|---|
| **Compound ID** | **SMILES** | **Activity class** | **Source** |
| **ML-428** | N#C/C4=C(\[H])C([H])=C(N1N=C(C([H])=C1OC([H])([H])C=2ON=C(C=2[H])C([H])([H])[H])C(=O)N3C([H])([H])[C@@]([H])(N(C([H])([H])C([H])([H])[H])C([H])([H])C([H])([H])[H])C([H])([H])C3([H])[H])C([H])=C4[H] | Active | 34 |
| **ML-429** | N#C/C5=C(\[H])C([H])=C(N1N=C(C([H])=C1OC([H])([H])C=2ON=C(C=2[H])C([H])([H])[H])C(=O)N4C([H])([H])[C@@]([H])(N3C([H])([H])C([H])([H])C([H])([H])C3([H])[H])C([H])([H])C([H])([H])C4([H])[H])C([H])=C5[H] | Active | 34 |
| **ML-430** | N#C/C4=C(\[H])C([H])=C(N1N=C(C([H])=C1OC([H])([H])C=2ON=C(C=2[H])C([H])([H])[H])C(=O)N3C([H])([H])[C@@]([H])(OC([H])([H])C3([H])[H])C([H])([H])N([H])[H])C([H])=C4[H] | Active | 34 |
| **ML-431** | N#C/C4=C(\[H])C([H])=C(N1N=C(C([H])=C1OC([H])([H])C=2ON=C(C=2[H])C([H])([H])[H])C(=O)N3C([H])([H])[C@]([H])(N([H])[H])[C@@]([F])([H])C([H])([H])C3([H])[H])C([H])=C4[H] | Active | 34 |
| **ML-432** | N#C/C4=C(\[H])C([H])=C(N1N=C(C([H])=C1OC([H])([H])C=2ON=C(C=2[H])C([H])([H])[H])C(=O)N3C([H])([H])C([H])([H])C([H])(N([H])[H])C([H])([H])C3([H])[H])C([H])=C4[H] | Active | 34 |
| **ML-433** | N#C/C4=C(\[H])C([H])=C(N1N=C(C([H])=C1OC([H])([H])C=2ON=C(C=2[H])C([H])([H])[H])C(=O)N3C([H])([H])C([H])([H])C([H])(C([H])([H])C3([H])[H])C([H])([H])N([H])[H])C([H])=C4[H] | Active | 34 |
| **ML-434** | O=[S](=O)(N(C([H])([H])[H])C([H])([H])[H])C=1C([H])=C([H])C([H])=C(C=1[H])C(=O)N([H])N=C(C=2C([H])=C([Cl])C([H])=C([H])C=2O[H])C([H])([H])[H] | Active | 23 |
| **ML-435** | [Cl]C=1C([H])=C([H])C(OC([H])([H])[H])=C(C=1[H])C(=O)N([H])N([H])C(=O)C=2C([H])=C(C([H])=C([H])C=2[F])[S](=O)(=O)N3C([H])([H])C([H])([H])OC([H])([H])C3([H])[H] | Active | 23 |

| **Table SM11**: ML Testing list compounds | | | |
|---|---|---|---|
| **Compound ID** | **SMILES** | **Activity class** | **Source** |
| **ML-436** | [F]C=1C([H])=C([H])C(=C([H])C=1C(=O)N([H])N([H])C(=O)C=2C([H])=C([H])C([H])=C([Cl])C=2[H])[S](=O)(=O)N3C([H])([H])C([H])([H])OC([H])([H])C3([H])[H] | Active | 23 |
| **ML-437** | O=C(N([H])N=C(C=1C([H])=C([H])C([H])=C([Cl])C=1[F])C([H])([H])[H])C=2C([H])=C([H])C([H])=C(C=2[H])[S](=O)(=O)N3C([H])([H])C([H])([H])OC([H])([H])C3([H])[H] | Active | 23 |
| **ML-438** | O=C(N([H])C1=C([H])C([H])=C([H])C(=C1[H])C([H])([H])O/C3=C(\[H])C([H])=C(OC2([H])C([H])([H])C([H])([H])N(C([H])([H])C2([H])[H])C([H])([H])[H])C([H])=C3[H])C=5N(C4=C([S]C([H])=C4[H])C=5[H])C([H])([H])[H] | Active | 36 |
| **ML-439** | O=C(/C2=C(\[H])C=1[S]C([H])=C([H])C=1N2C([H])([H])[H])N([H])C3=C([H])C([H])=C(C([H])=C3[H])C([H])([H])O/C5=C(\[H])C([H])=C(OC4([H])C([H])([H])C([H])([H])N(C([H])([H])C4([H])[H])C([H])([H])[H])C([H])=C5[H] | Active | 24 |
| **ML-440** | O=C(/C2=C(\[H])C=1[S]C([H])=C([H])C=1N2C([H])([H])[H])N([H])C3=C([H])C([H])=C([H])C([H])=C3C([H])([H])O/C5=C(\[H])C(OC4([H])C([H])([H])C([H])([H])N(C([H])([H])C4([H])[H])C([H])([H])[H])=C([H])C([H])=C5[H] | Active | 24 |
| **ML-441** | O=C(/C2=C(\[H])C=1[S]C([H])=C([H])C=1N2C([H])([H])[H])N([H])C5=C(OC([H])([H])C4=C([H])C([H])=C(OC3([H])C([H])([H])C([H])([H])N(C([H])([H])C3([H])[H])C([H])([H])[H])C([H])=C4[H])C([H])=C([H])C([H])=C5[H] | Active | 24 |

**Table SM11**: ML Testing list compounds

| Compound ID | SMILES | Activity class | Source |
|---|---|---|---|
| **ML-442** | O=C(/C2=C(\[H])C=1[S]C([H])=C([H])C=1N2C([H])([H])[H])N([H])C3=C(C([H])=C([H])C([H])=C3[H])C([H])([H])O/C4=C(\[H])C([H])=C(C([H])=C4[H])C([H])([H])N5C([H])([H])C([H])([H])N(C([H])([H])C5([H])[H])C([H])([H])[H] | Active | 24 |
| **ML-443** | O=C(/C2=C(\[H])C=1[S]C([H])=C([H])C=1N2C([H])([H])[H])N([H])C3=C(C([H])=C([H])C([H])=C3[H])C([H])([H])O/C5=C(\[H])C([H])=C(OC4([H])C([H])([H])C([H])([H])N(C([H])([H])C4([H])[H])C([H])([H])C([H])([H])[H])C([H])=C5[H] | Active | 24 |
| **ML-444** | O=C(/C2=C(\[H])C=1[S]C([H])=C([H])C=1N2C([H])([H])[H])N([H])C3=C(C([H])=C([H])C([H])=C3[H])C([H])([H])O/C6=C(\[H])C([H])=C(O[C@]4([H])C([H])([H])[C@]5([H])N([H])[C@]([H])(C4([H])[H])C([H])([H])C5([H])[H])C([H])=C6[H] | Active | 24 |
| **ML-445** | O=C(/C2=C(\[H])C=1[S]C([H])=C([H])C=1N2C([H])([H])[H])N([H])C3=C(C([H])=C([H])C([H])=C3[H])C([H])([H])O/C5=C(\[H])C([H])=C(OC([H])([H])C4([H])C([H])([H])C([H])([H])N([H])C([H])([H])C4([H])[H])C([H])=C5[H] | Active | 24 |
| **ML-446** | O=C(/C2=C(\[H])C=1[S]C([H])=C([H])C=1N2C([H])([H])[H])N([H])C3=C(/C(=C(/[H])C([H])=C3[H])C([H])([H])OC([H])([H])[H])C([H])([H])O/C5=C(\[H])C([H])=C(OC4([H])C([H])([H])C([H])([H])N([H])C([H])([H])C4([H])[H])C([H])=C5[H] | Active | 24 |
| **ML-447** | O=C(/C2=C(\[H])C=1[S]C([H])=C([H])C=1N2C([H])([H])[H])N([H])C3=C(/C(=C(/[H])C([H])=C3[H])C([H])([H])OC([H])([H])[H])C([H])([H])O/C5=C(\[H])C([H])=C(OC([H])([H])[C@]4([H])C([H])([H])C([H])([H])N([H])C4([H])[H])C([H])=C5[H] | Active | 24 |
| **ML-448** | O=C(/C2=C(\[H])C=1[S]C([H])=C([H])C=1N2C([H])([H])[H])N([H])C3=C(/C(=C(/[H])C([H])=C3[H])C([H])([H])OC([H])(C([H])([H])[H])C([H])([H])[H])C([H])([H])O/C5=C(\[H])C([H])=C(OC4([H])C([H])([H])C([H])([H])N([H])C([H])([H])C4([H])[H])C([H])=C5[H] | Active | 24 |

**Table SM11**: ML Testing list compounds

| Compound ID | SMILES | Activity class | Source |
|---|---|---|---|
| **ML-449** | N#C/C4=C(\[F])C([H])=C(/C1=N/C(=C(N1/C2=C(\[H])C([H])=C(/N=C2/[H])OC([H])([H])[H])C([H])([H])[H])C(=O)N3C([H])([H])C([H])([H])C([H])([H])[C@@]([H])(N([H])[H])C3([H])[H])C([H])=C4[H] | Active | 35 |
| **ML-450** | O=C1N([H])C(=O)[C@@]([H])(N1[H])C([H])([H])C([H])([H])C(=O)N([H])C([H])([H])C([H])([H])C=2C3=C(N([H])C=2[H])C([H])=C([H])C([H])=C3[H] | Active | 31 |
| **ML-451** | O=C1N(C(=O)N([H])C(=O)C1=C([H])C2=C([H])C([H])=C(O[H])C(O[H])=C2[H])C3=C([H])C([H])=C(C#N)C([H])=C3[H] | Active | 26 |
| **ML-452** | O=C1N(C(=O)N([H])C(=O)C1=C([H])C2=C([H])C([H])=C(O[H])C(OC([H])([H])[H])=C2[H])C3=C([H])C([H])=C([Br])C([H])=C3[H] | Active | 26 |
| **ML-453** | [Cl]/C2=N/C(=NC(/C1=C(\[H])C(OC([H])([H])[H])=C(OC([H])([H])[H])C(OC([H])([H])[H])=C1[H])=C2C#N)[S]C([H])([H])C#C[H] | Active | 18 |
| **ML-454** | O=C1N(C(=O)N([H])C(=O)C1=C([H])C2=C([H])C(OC([H])([H])[H])=C(O[H])C(OC([H])([H])[H])=C2[H])C3=C([H])C([H])=C([Br])C([H])=C3[H] | Active | 26 |
| **ML-455** | O=C(N([H])C1=C([H])C([H])=C([H])C(=C1C([H])([H])O/C3=C(\[H])C([H])=C(OC2([H])C([H])([H])C([H])([H])N([H])C([H])([H])C2([H])[H])C([H])=C3[H])C([H])([H])OC([H])([H])[H])C=4N(C=5C(C=4[H])=C([H])C([H])=C([H])C=5[H])C([H])([H])[H] | Active | 26 |
| **ML-456** | O=C(N1C([H])([H])C([H])([H])C(N([H])[H])(C([H])([H])C1([H])[H])C([H])([H])[H])C([H])([H])N(/C2=C(\[H])C([H])=C(C#N)C([H])=C2[H])[S](=O)(=O)C3=C([H])C([H])=C(C([H])=C3[H])C([H])([H])[H] | Active | 33 |
| **CHEMBL3104345** | O=C(OC([H])([H])[H])C=1C([H])=C(C([H])=C(C=1[H])C(=O)N3C([H])([H])C([H])([H])N(C(=O)C=2C([H])=C([H])C(C([H])=N[H])=C([H])C=2[H])C([H])([H])C3([H])[H])C([H])([H])N4C([H])([H])C([H])([H])N(/C(=N/[H])N([H])[H])C([H])([H])C4([H])[H] | Active | ChEMBL database |

**Table SM11**: ML Testing list compounds

| Compound ID | SMILES | Activity class | Source |
|---|---|---|---|
| **CHEMBL3682527** | O=[S](=O)(N(C([H])([H])C([H])([H])[H])C([H])([H])C([H])([H])[H])C=1C([H])=C([H])C([F])=C(C=1[H])C(=O)N([H])N([H])C(=O)C=2C([H])=C([H])C(=C([H])C=2[H])C([H])([H])[H] | Active | ChEMBL database |
| **CHEMBL3682528** | O=C(/C1=C(\O[H])C([H])=C([H])C([H])=C1[H])N([H])N([H])C(=O)C=2C([H])=C(C([H])=C([H])C=2[F])[S](=O)(=O)N3C([H])([H])C([H])([H])OC([H])([H])C3([H])[H] | Active | ChEMBL database |
| **CHEMBL3682533** | O=C(N([H])N=C(C=1C([H])=C([Cl])C([H])=C([H])C=1O[H])C([H])([H])C([H])([H])[H])C=2C([H])=C([H])C([H])=C(C=2[H])[S](=O)(=O)N3C([H])([H])C([H])([H])OC([H])([H])C3([H])[H] | Active | ChEMBL database |
| **CHEMBL3682536** | O=C(N([H])N=C(C=1C([H])=C([Cl])C([H])=C([H])C=1O[H])C([H])([H])[H])C=2C([H])=NC([H])=C(C=2[H])[S](=O)(=O)C([H])([H])[H] | Active | ChEMBL database |
| **CHEMBL3682537** | O=C(N([H])N=C(C=1C([H])=C([Cl])C([H])=C([H])C=1O[H])C([H])([H])[H])C=2C([H])=C([H])C([H])=C(C=2[H])[S](=O)(=O)C([H])([H])[H] | Active | ChEMBL database |
| **CHEMBL3786420** | O=C(N([H])C=1C([H])=C([H])C(=C([Cl])C=1[H])C([H])([H])[H])C([H])([H])C([H])([H])C(=O)OC([H])([H])C(=O)C=2C([H])=C([H])C(=C([H])C=2[H])C=3C([H])=C([H])C([H])=C([H])C=3[H] | Active | ChEMBL database |
| **CHEMBL4065771** | [H]C([H])([H])C([H])([H])C([H])([H])[S]C=3/N=C(/[S]/C1=N/C([H])=C([H])C([H])=C1[H])C=2N=NN(C=2N=3)C([H])([H])C([H])([H])C=4[S]C([H])=C([H])C=4[H] | Active | ChEMBL database |
| **CHEMBL4074390** | [H]C([H])([H])C([H])([H])C([H])([H])[S]C=3/N=C(/[S]/C1=N/C([H])=C([H])C([H])=C1[H])C=2N=NN(C=2N=3)C([H])([H])C([H])(C([H])([H])[H])C([H])([H])[H] | Active | ChEMBL database |
| **CHEMBL4076163** | N#CC=4C([H])=C([H])C(N1N=C(C([H])=C1OC([H])([H])C=2ON=C(C=2[H])C([H])([H])[H])C(=O)N3C([H])([H])C([H])([H])C([H])([H])[C@@]([H])(N([H])[H])C3([H])[H])=C([H])C=4[H] | Active | ChEMBL database |
| **CHEMBL4080173** | N#CC=5C([H])=C([H])C(N1N=C(C([H])=C1OC([H])([H])C=2ON=C(C=2[H])C([H])([H])[H])C(=O)N4C([H])([H])C([H])([H])C([H])([H])[C@@]([H])(N3C([H])([H])C([H])([H])C([H])([H])C3([H])[H])C4([H])[H])=C([H])C=5[H] | Active | ChEMBL database |

**Table SM11**: ML Testing list compounds

| Compound ID | SMILES | Activity class | Source |
|---|---|---|---|
| **CHEMBL4089024** | O=C(N([H])N([H])[C@]4([H])N3/N=C(/[S]C([H])([H])C2=N/C1=C(\[H])C([H])=C([H])C([H])=C1N2[H])N=C3N([H])[C@]([H])(C([H])([H])[H])C4([H])[H])C5=NC([H])=C([H])N=C5[H] | Active | ChEMBL database |
| **CHEMBL4092725** | N#CC=3C([H])=C([H])C(N1N=C(C([H])=C1OC([H])([H])[C@]([H])(O[H])C([H])([H])[H])C(=O)N2C([H])([H])C([H])([H])C([H])([H])[C@@]([H])(N([H])[H])C2([H])[H])=C([H])C=3[H] | Active | ChEMBL database |
| **CHEMBL4094875** | [H]C([H])([H])C([H])([H])C([H])([H])[S]C=3/N=C(/[S]/C1=N/C([H])=C([H])C([H])=C1[H])C=2N=NN(C=2N=3)C4([H])C([H])([H])C([H])([H])C([H])([H])C4([H])[H] | Active | ChEMBL database |
| **CHEMBL4095768** | O=C(N1C([H])([H])C([H])([H])C([H])([H])[C@@]([H])(N(C([H])([H])[H])C([H])([H])[H])C1([H])[H])C3=NN(C(OC([H])([H])C=2ON=C(C=2[H])C([H])([H])[H])=C3[H])C=4C([H])=C([H])C(C#N)=C([H])C=4[H] | Active | ChEMBL database |
| **CHEMBL4095820** | [H]OC([H])([H])C([H])([H])N2N=NC=1C(=NC([S]C([H])([H])C([H])([H])C([H])([H])[H])=NC=12)[S]/C3=N/C([H])=C([H])C([H])=C3[H] | Active | ChEMBL database |
| **CHEMBL4096682** | O=C(N1C([H])([H])C([H])([H])[C@@]([H])(N(C([H])([H])C([H])([H])[H])C([H])([H])C([H])([H])[H])C1([H])[H])C3=NN(C(OC([H])([H])C=2ON=C(C=2[H])C([H])([H])[H])=C3[H])C=4C([H])=C([H])C(C#N)=C([H])C=4[H] | Active | ChEMBL database |
| **CHEMBL4099019** | O=C(N([H])N([H])[C@]3([H])N2/N=C(/[S]C([H])([H])C=1C([H])=C([H])C([H])=C([H])C=1[H])N=C2N([H])[C@]([H])(C([H])([H])[H])C3([H])[H])C4=NC([H])=C([H])N=C4[H] | Active | ChEMBL database |
| **CHEMBL4100915** | [H][C@@]4(N([H])[H])C([H])([H])C([H])([H])C([H])([H])N(C(=O)C2=NN(C(OC([H])([H])C1=NC([H])=C([H])C([H])=C1[H])=C2[H])C=3C([H])=C([H])C(C#N)=C([H])C=3[H])C4([H])[H] | Active | ChEMBL database |
| **CHEMBL4104407** | N#CC=4C([H])=C([H])C(N1N=C(C([H])=C1OC([H])([H])C=2ON=C(C=2[H])C([H])([H])[H])C(=O)N3C([H])([H])C([H])([H])O[C@]([H])(C([H])([H])N([H])[H])C3([H])[H])=C([H])C=4[H] | Active | ChEMBL database |

**Table SM11**: ML Testing list compounds

| Compound ID | SMILES | Activity class | Source |
|---|---|---|---|
| **ML-457** | [F]C([F])([F])C=3C([H])=C([H])C([H])=C(N([H])C=2/N=C(/[S]C([H])([H])C#C[H])N=C(C=1C([H])=C([H])C([H])=C([H])C=1[H])C=2C#N)C=3[H] | Inactive | 18 |
| **ML-458** | O=C2OC=1C([H])=C([H])C(=C([H])C=1/C(=C2/[H])C([H])([H])N3N=N/C(=C3/[H])C([H])([H])[S]C(=[S])N4C([H])([H])C([H])([H])N(C(=O)OC(C([H])([H])[H])(C([H])([H])[H])C([H])([H])[H])C([H])([H])C4([H])[H])C([H])([H])[H] | Inactive | 27 |
| **ML-459** | O=C(OC(C([H])([H])[H])(C([H])([H])[H])C([H])([H])[H])N4C([H])([H])C([H])([H])N(C(=[S])[S]C([H])([H])C=1N=NN(C=1[H])C([H])([H])C3=C([H])C=2C([H])=C([H])C([H])=C([H])C=2OC3=O)C([H])([H])C4([H])[H] | Inactive | 27 |
| **ML-460** | O=C(OC(C([H])([H])[H])(C([H])([H])[H])C([H])([H])[H])N4C([H])([H])C([H])([H])N(C(=[S])[S]C([H])([H])C=1N=NN(C=1[H])C([H])([H])C3=C([H])C(=O)OC=2C([H])=C([H])C([Cl])=C([H])C=23)C([H])([H])C4([H])[H] | Inactive | 27 |
| **ML-461** | [S]=C([S]C([H])([H])C=1N=NN(C=1[H])C([H])([H])C=2C([H])=C([H])C([H])=C([H])C=2[F])N3C([H])([H])C([H])([H])N(C([H])([H])[H])C([H])([H])C3([H])[H] | Inactive | 28 |
| **ML-462** | [S]=C([S]C([H])([H])C=1N=NN(C=1[H])C([H])([H])C=2C([H])=C([H])C([F])=C([H])C=2[H])N3C([H])([H])C([H])([H])N(C([H])([H])[H])C([H])([H])C3([H])[H] | Inactive | 28 |
| **ML-463** | [S]=C([S]C([H])([H])C=1N=NN(C=1[H])C([H])([H])C=2C([H])=C([H])C([H])=C([H])C=2[F])N3C([H])([H])C([H])([H])N(C([H])([H])C([H])([H])[H])C([H])([H])C3([H])[H] | Inactive | 28 |
| **ML-476** | [F]C=1C([H])=C([H])C([H])=C([H])C=1C([H])([H])N2N=N/C(=C2/[H])C([H])([H])[S]C(=[S])N3C([H])([H])C([H])([H])OC([H])([H])C3([H])[H] | Inactive | 28 |

**Table SM11**: ML Testing list compounds



| Compound ID | SMILES | Activity class | Source |
|---|---|---|---|
| **ML-477** | [S]=C([S]C([H])([H])C=1N=NN(C=1[H])C([H])([H])C([H])([H])C=2C([H])=C([H])C(=C([H])C=2[H])C([H])([H])[H])N3C([H])([H])C([H])([H])N(C(=O)OC(C([H])([H])[H])(C([H])([H])[H])C([H])([H])[H])C([H])([H])C3([H])[H] | Inactive | 28 |
| **ML-478** | [S]=C([S]C([H])([H])C=1N=NN(C=1[H])C([H])([H])C2=C([H])C(OC([H])([H])[H])=C(OC([H])([H])[H])C(OC([H])([H])[H])=C2[H])N3C([H])([H])C([H])([H])N([H])C([H])([H])C3([H])[H] | Inactive | 28 |
| **ML-479** | O=C(OC(C([H])([H])[H])(C([H])([H])[H])C([H])([H])[H])N3C([H])([H])C([H])([H])N(C(=[S])[S]C([H])([H])C=1N=NN(C=1[H])C=2C([H])=C([H])C([H])=C(C=2[H])C([F])([F])[F])C([H])([H])C3([H])[H] | Inactive | 28 |
| **ML-480** | [S]=C([S]C([H])([H])C=2N=NN(C=1C([H])=C([H])C(OC([H])([H])[H])=C([H])C=1[H])C=2[H])N3C([H])([H])C([H])([H])N(C(=O)OC(C([H])([H])[H])(C([H])([H])[H])C([H])([H])[H])C([H])([H])C3([H])[H] | Inactive | 28 |
| **ML-481** | [F]C=1C([H])=C([H])C(=C([H])C=1[H])C([H])([H])N2N=N/C(=C2/[H])C([H])([H])[S]C(=[S])N3C([H])([H])C([H])([H])C([H])([H])C([H])([H])C3([H])[H] | Inactive | 28 |
| **ML-482** | [F]C=1C([H])=C([H])C(=C([H])C=1[H])C([H])([H])N2N=N/C(=C2/[H])C([H])([H])[S]C(=[S])N3C([H])([H])C([H])([H])OC([H])([H])C3([H])[H] | Inactive | 28 |
| **ML-483** | [Cl]C=1C([H])=C([H])C(=C([H])C=1[H])C([H])([H])N2N=N/C(=C2/[H])C([H])([H])[S]C(=[S])N3C([H])([H])C([H])([H])OC([H])([H])C3([H])[H] | Inactive | 28 |
| **ML-484** | O=C(OC(C([H])([H])[H])(C([H])([H])[H])C([H])([H])[H])N4C([H])([H])C([H])([H])N(C(=[S])[S]C([H])([H])C=1N=NN(C=1[H])C([H])([H])C3=C([H])C(=O)N([H])C=2C([H])=C([H])C([H])=C([H])C=23)C([H])([H])C4([H])[H] | Inactive | 28 |
| **ML-485** | [S]=C([S]C([H])([H])C=1N=NN(C=1[H])C([H])([H])C=2C([H])=C([H])C(=C([H])C=2[H])C([H])([H])[H])N3C([H])([H])C([H])([H])N(C([H])([H])[H])C([H])([H])C3([H])[H] | Inactive | 28 |

**Table SM11**: ML Testing list compounds

| Compound ID | SMILES | Activity class | Source |
|---|---|---|---|
| **ML-486** | [F]C=1C([H])=C([H])C(=C([H])C=1[H])C([H])([H])N2N=N/C(=C2/[H])C([H])([H])[S]C(=[S])N4C([H])([H])C([H])([H])N(C=3C([H])=C([H])C([H])=C([H])C=3[H])C([H])([H])C4([H])[H] | Inactive | 28 |
| **ML-487** | O=C(OC(C([H])([H])[H])(C([H])([H])[H])C([H])([H])[H])N3C([H])([H])C([H])([H])N(C(=[S])[S]C([H])([H])C=1N=NN(C=1[H])C=2C([H])=C([H])C([H])=C([H])C=2[F])C([H])([H])C3([H])[H] | Inactive | 28 |
| **ML-464** | [Cl]C=1C([H])=C([H])C(=C([H])C=1[H])C([H])([H])N2N=N/C(=C2/[H])C([H])([H])[S]C(=[S])N4C([H])([H])C([H])([H])N(C(=O)OC([H])([H])C=3C([H])=C([H])C([H])=C([H])C=3[H])C([H])([H])C4([H])[H] | Inactive | 28 |
| **ML-465** | [S]=C([S]C([H])([H])C=1N=NN(C=1[H])C([H])([H])C2=C(C([H])=C([H])C([H])=C2[H])C([H])([H])[H])N3C([H])([H])C([H])([H])N(C(=O)OC(C([H])([H])[H])(C([H])([H])[H])C([H])([H])[H])C([H])([H])C3([H])[H] | Inactive | 28 |
| **ML-466** | O=C(OC(C([H])([H])[H])(C([H])([H])[H])C([H])([H])[H])N3C([H])([H])C([H])([H])N(C(=[S])[S]C([H])([H])C=1N=NN(C=1[H])C([H])([H])C=2C([H])=C([H])C(=C([H])C=2[H])[N+](=O)[O-])C([H])([H])C3([H])[H] | Inactive | 28 |
| **ML-467** | O=C(OC(C([H])([H])[H])(C([H])([H])[H])C([H])([H])[H])N4C([H])([H])C([H])([H])N(C(=[S])[S]C([H])([H])C=1N=NN(C=1[H])C([H])([H])C3=N/C2=C(\[H])C([H])=C([H])C([H])=C2N3[H])C([H])([H])C4([H])[H] | Inactive | 28 |
| **ML-468** | O=C(OC(C([H])([H])[H])(C([H])([H])[H])C([H])([H])[H])N3C([H])([H])C([H])([H])N(C(=[S])[S]C([H])([H])C=1N=NN(C=1[H])C([H])([H])C2=C([H])C(=O)OC2([H])[H])C([H])([H])C3([H])[H] | Inactive | 28 |
| **ML-469** | O=C(OC(C([H])([H])[H])(C([H])([H])[H])C([H])([H])[H])N4C([H])([H])C([H])([H])N(C(=[S])[S]C([H])([H])C=1N=NN(C=1[H])C3=C([H])C(=O)OC=2C([H])=C([H])C([H])=C([H])C=23)C([H])([H])C4([H])[H] | Inactive | 28 |

**Table SM11**: ML Testing list compounds

| Compound ID | SMILES | Activity class | Source |
|---|---|---|---|
| **ML-470** | [S]=C([S]C([H])([H])C=1N=NN(C=1[H])C([H])([H])C=2C([H])=C([H])C(=C([H])C=2[H])C([H])([H])[H])N3C([H])([H])C([H])([H])N(C([H])([H])C([H])([H])[H])C([H])([H])C3([H])[H] | Inactive | 28 |
| **ML-471** | O=C(OC(C([H])([H])[H])(C([H])([H])[H])C([H])([H])[H])N3C([H])([H])C([H])([H])N(C(=[S])[S]C([H])([H])C=1N=NN(C=1[H])C([H])([H])C=2C([H])=C([H])C(=C([H])C=2[H])C([F])([F])[F])C([H])([H])C3([H])[H] | Inactive | 28 |
| **ML-472** | O=C(OC(C([H])([H])[H])(C([H])([H])[H])C([H])([H])[H])N3C([H])([H])C([H])([H])N(C(=[S])[S]C([H])([H])C=1N=NN(C=1[H])C([H])([H])C=2C([H])=C([H])C([H])=C([H])C=2C([F])([F])[F])C([H])([H])C3([H])[H] | Inactive | 28 |
| **ML-473** | [F]C=1C([H])=C([H])C([H])=C([H])C=1C([H])([H])N2N=N/C(=C2/[H])C([H])([H])[S]C(=[S])N4C([H])([H])C([H])([H])N(C([H])([H])C=3C([H])=C([H])C([H])=C([H])C=3[H])C([H])([H])C4([H])[H] | Inactive | 28 |
| **ML-474** | [F]C=1C([H])=C([H])C([H])=C([H])C=1C([H])([H])N2N=N/C(=C2/[H])C([H])([H])[S]C(=[S])N4C([H])([H])C([H])([H])N(C=3C([H])=C([H])C([H])=C([H])C=3[H])C([H])([H])C4([H])[H] | Inactive | 28 |
| **ML-475** | [Cl]C=1C([H])=C([H])C(=C([H])C=1[Cl])C([H])([H])N2N=N/C(=C2/[H])C([H])([H])[S]C(=[S])N3C([H])([H])C([H])([H])N([H])C([H])([H])C3([H])[H] | Inactive | 28 |
| **ML-476** | [F]C=1C([H])=C([H])C([H])=C([H])C=1C([H])([H])N2N=N/C(=C2/[H])C([H])([H])[S]C(=[S])N3C([H])([H])C([H])([H])OC([H])([H])C3([H])[H] | Inactive | 28 |
| **ML-477** | [S]=C([S]C([H])([H])C=1N=NN(C=1[H])C([H])([H])C([H])([H])C=2C([H])=C([H])C(=C([H])C=2[H])C([H])([H])[H])N3C([H])([H])C([H])([H])N(C(=O)OC(C([H])([H])[H])(C([H])([H])[H])C([H])([H])[H])C([H])([H])C3([H])[H] | Inactive | 28 |

**Table SM11**: ML Testing list compounds

| Compound ID | SMILES | Activity class | Source |
|---|---|---|---|
| **ML-478** | [S]=C([S]C([H])([H])C=1N=NN(C=1[H])C([H])([H])C2=C([H])C(OC([H])([H])[H])=C(OC([H])([H])[H])C(OC([H])([H])[H])=C2[H])N3C([H])([H])C([H])([H])N([H])C([H])([H])C3([H])[H] | Inactive | 28 |
| **ML-479** | O=C(OC(C([H])([H])[H])(C([H])([H])[H])C([H])([H])[H])N3C([H])([H])C([H])([H])N(C(=[S])[S]C([H])([H])C=1N=NN(C=1[H])C=2C([H])=C([H])C([H])=C(C=2[H])C([F])([F])[F])C([H])([H])C3([H])[H] | Inactive | 28 |
| **ML-480** | [S]=C([S]C([H])([H])C=2N=NN(C=1C([H])=C([H])C(OC([H])([H])[H])=C([H])C=1[H])C=2[H])N3C([H])([H])C([H])([H])N(C(=O)OC(C([H])([H])[H])(C([H])([H])[H])C([H])([H])[H])C([H])([H])C3([H])[H] | Inactive | 28 |
| **ML-481** | [F]C=1C([H])=C([H])C(=C([H])C=1[H])C([H])([H])N2N=N/C(=C2/[H])C([H])([H])[S]C(=[S])N3C([H])([H])C([H])([H])C([H])([H])C([H])([H])C3([H])[H] | Inactive | 28 |
| **ML-482** | [F]C=1C([H])=C([H])C(=C([H])C=1[H])C([H])([H])N2N=N/C(=C2/[H])C([H])([H])[S]C(=[S])N3C([H])([H])C([H])([H])OC([H])([H])C3([H])[H] | Inactive | 28 |
| **ML-483** | [Cl]C=1C([H])=C([H])C(=C([H])C=1[H])C([H])([H])N2N=N/C(=C2/[H])C([H])([H])[S]C(=[S])N3C([H])([H])C([H])([H])OC([H])([H])C3([H])[H] | Inactive | 28 |
| **ML-484** | O=C(OC(C([H])([H])[H])(C([H])([H])[H])C([H])([H])[H])N4C([H])([H])C([H])([H])N(C(=[S])[S]C([H])([H])C=1N=NN(C=1[H])C([H])([H])C3=C([H])C(=O)N([H])C=2C([H])=C([H])C([H])=C([H])C=23)C([H])([H])C4([H])[H] | Inactive | 28 |
| **ML-485** | [S]=C([S]C([H])([H])C=1N=NN(C=1[H])C([H])([H])C=2C([H])=C([H])C(=C([H])C=2[H])C([H])([H])[H])N3C([H])([H])C([H])([H])N(C([H])([H])[H])C([H])([H])C3([H])[H] | Inactive | 28 |
| **ML-486** | [F]C=1C([H])=C([H])C(=C([H])C=1[H])C([H])([H])N2N=N/C(=C2/[H])C([H])([H])[S]C(=[S])N4C([H])([H])C([H])([H])N(C=3C([H])=C([H])C([H])=C([H])C=3[H])C([H])([H])C4([H])[H] | Inactive | 28 |

**Table SM11**: ML Testing list compounds

| Compound ID | SMILES | Activity class | Source |
|---|---|---|---|
| **ML-487** | O=C(OC(C([H])([H])[H])(C([H])([H])[H])C([H])([H])[H])N3C([H])([H])C([H])([H])N(C(=[S])[S]C([H])([H])C=1N=NN(C=1[H])C=2C([H])=C([H])C([H])=C([H])C=2[F])C([H])([H])C3([H])[H] | Inactive | 28 |
| **ML-488** | [S]=C([S]C([H])([H])C=2N=NN(C=1C([H])=C([H])C(=C([H])C=1[H])C([H])([H])[H])C=2[H])N3C([H])([H])C([H])([H])N(C(=O)OC(C([H])([H])[H])(C([H])([H])[H])C([H])([H])[H])C([H])([H])C3([H])[H] | Inactive | 28 |
| **ML-489** | [Cl]C=1C([H])=C([H])C(=C([H])C=1[H])C([H])([H])N2N=N/C(=C2/[H])C([H])([H])[S]C(=[S])N3C([H])([H])C([H])([H])C([H])([H])C([H])([H])C3([H])[H] | Inactive | 28 |
| **ML-490** | O=C(OC(C([H])([H])[H])(C([H])([H])[H])C([H])([H])[H])N3C([H])([H])C([H])([H])N(C(=[S])[S]C([H])([H])C=1N=NN(C=1[H])C([H])([H])C([H])([H])C=2C([H])=C([H])C([F])=C([H])C=2[H])C([H])([H])C3([H])[H] | Inactive | 28 |
| **ML-491** | [S]=C([S]C([H])([H])C=1N=NN(C=1[H])C([H])([H])C=2C([H])=C([H])C([H])=C([H])C=2[Cl])N3C([H])([H])C([H])([H])N(C([H])([H])[H])C([H])([H])C3([H])[H] | Inactive | 28 |
| **ML-492** | [S]=C([S]C([H])([H])C=1N=NN(C=1[H])C([H])([H])C=2C([H])=C([H])C([F])=C([H])C=2[H])N3C([H])([H])C([H])([H])N(C([H])([H])C([H])([H])O[H])C([H])([H])C3([H])[H] | Inactive | 28 |
| **ML-493** | [F]C=1C([H])=C([H])C([H])=C([F])C=1C([H])([H])N2N=N/C(=C2/[H])C([H])([H])[S]C(=[S])N3C([H])([H])C([H])([H])N([H])C([H])([H])C3([H])[H] | Inactive | 28 |
| **ML-494** | [F]C=1C([H])=C([H])C([H])=C([H])C=1C([H])([H])N2N=N/C(=C2/[H])C([H])([H])[S]C(=[S])N3C([H])([H])C([H])([H])C([H])([H])C([H])([H])C3([H])[H] | Inactive | 28 |
| **ML-495** | O=C(OC(C([H])([H])[H])(C([H])([H])[H])C([H])([H])[H])N4C([H])([H])C([H])([H])N(C(=[S])[S]C([H])([H])C=1N=NN(C=1[H])C([H])([H])C([H])([H])O/C3=C(\[H])C(=O)OC=2C([H])=C([H])C([H])=C([H])C=23)C([H])([H])C4([H])[H] | Inactive | 28 |

**Table SM11**: ML Testing list compounds



| Compound ID | SMILES | Activity class | Source |
|---|---|---|---|
| **ML-496** | [F]C=1C([H])=C([H])C(=C([H])C=1[H])C([H])([H])[S]/C3=N/C(/C2=C(\[H])C(OC([H])([H])[H])=C(OC([H])([H])[H])C(OC([H])([H])[H])=C2[H])=C(C#N)C(=N3)N([H])N([H])C(=[S])N([H])[H] | Inactive | 18 |
| **ML-497** | [Cl]C=3C([H])=C([H])C(/C2=N/C(=NC(N([H])C=1C([H])=C([H])C(OC([H])([H])[H])=C([H])C=1[H])=C2C#N)[S]C([H])([H])C#C[H])=C([H])C=3[H] | Inactive | 18 |
| **ML-498** | [Cl]C=3C([H])=C([H])C(N([H])C=2/N=C(/[S]C([H])([H])C#C[H])N=C(C=1C([H])=C([H])C([Br])=C([H])C=1[H])C=2C#N)=C([H])C=3[H] | Inactive | 18 |
| **ML-499** | N#C/C1=C(/N=C(/[S]C([H])([H])C#C[H])N=C1C=2C([H])=C(OC([H])([H])[H])C(OC([H])([H])[H])=C(OC([H])([H])[H])C=2[H])N([H])C=3C([H])=C([H])C(OC([H])([H])[H])=C([H])C=3[H] | Inactive | 18 |
| **ML-500** | O=C(N([H])C1=C([H])C([H])=C([H])N=C1[H])C([H])([H])N(/C2=C(\[H])C([H])=C(C#N)C([H])=C2[H])[S](=O)(=O)C3=C([H])C([H])=C(C([H])=C3[H])C([H])([H])[H] | Inactive | 33 |
| **ML-501** | [F]C([F])([F])C3=C([H])C([H])=C([H])C(N([H])C2=NC(=NC(/C1=C(\[H])C(OC([H])([H])[H])=C(OC([H])([H])[H])C(OC([H])([H])[H])=C1[H])=C2C#N)[S]C([H])([H])C#C[H])=C3[H] | Inactive | 18 |
| **ML-502** | [Cl]C=3C([H])=C([H])C(N([H])C=2/N=C(/[S]C([H])([H])C#C[H])N=C(C=1C([H])=C([H])C([H])=C([H])C=1[H])C=2C#N)=C([H])C=3[H] | Inactive | 18 |
| **ML-503** | [O-][N+](=O)C=3C([H])=C([H])C(N([H])C=2/N=C(/[S]C([H])([H])C#C[H])N=C(C=1C([H])=C([H])C([H])=C([H])C=1[H])C=2C#N)=C([H])C=3[H] | Inactive | 18 |
| **ML-504** | N#C/C1=C(/N=C(/[S]C([H])([H])C#C[H])N=C1C=2C([H])=C([H])C([H])=C([H])C=2[H])N([H])C=3C([H])=C([H])C(OC([H])([H])[H])=C([H])C=3[H] | Inactive | 18 |
| **ML-505** | [Cl]C=1C([H])=C([H])C([H])=C([H])C=1N([H])C=3/N=C(/[S]C([H])([H])C#C[H])N=C(C=2C([H])=C([H])C([H])=C([H])C=2[H])C=3C#N | Inactive | 18 |
| **ML-506** | [Cl]C=3C([H])=C([H])C([H])=C(N([H])C=2/N=C(/[S]C([H])([H])C#C[H])N=C(C=1C([H])=C([H])C([H])=C([H])C=1[H])C=2C#N)C=3[H] | Inactive | 18 |

| **Table SM11**: ML Testing list compounds | | | |
|---|---|---|---|
| **Compound ID** | **SMILES** | **Activity class** | **Source** |
| **ML-507** | [F]C([F])([F])C=3C([H])=C([H])C([H])=C(N([H])C=2/N=C(/[S]C([H])([H])C#C[H])N=C(C=1C([H])=C([H])C([Cl])=C([H])C=1[H])C=2C#N)C=3[H] | Inactive | 18 |
| **CHEMBL121** | O=C1[S][C@]([H])(C(=O)N1[H])C([H])([H])C=3C([H])=C([H])C(OC([H])([H])C([H])([H])N(/C2=N/C([H])=C([H])C([H])=C2[H])C([H])([H])[H])=C([H])C=3[H] | Inactive | ChEMBL database |
| **CHEMBL225304** | [H]N([H])N=C(N([H])[H])N([H])[H] | Inactive | ChEMBL database |
| **CHEMBL4061050** | O=C(OC(C([H])([H])[H])(C([H])([H])[H])C([H])([H])[H])N4C([H])([H])C([H])([H])N(/C1=N/C([S]C([H])([H])C#C[H])=NC2=C1N=NN2C([H])([H])C=3C([H])=C([H])C([H])=C([H])C=3[H])C([H])([H])C4([H])[H] | Inactive | ChEMBL database |
| **CHEMBL4065294** | [Cl]C=3C([H])=C([H])C(N([H])C1=NC([S]C([H])([H])C([H])([H])C([H])([H])[H])=NC2=C1N=NN2C([H])([H])C([H])([H])O[H])=C([H])C=3[H] | Inactive | ChEMBL database |
| **CHEMBL4068009** | [H]N(/C1=N/C([S]C([H])([H])C([H])([H])C([H])([H])[H])=NC2=C1N=NN2C([H])([H])C([H])([H])O[H])C=3C([H])=C(OC([H])([H])[H])C(OC([H])([H])[H])=C(OC([H])([H])[H])C=3[H] | Inactive | ChEMBL database |
| **CHEMBL4079306** | [H]OC([H])([H])C([H])([H])N2N=NC=1C(=NC([S]C([H])([H])C([H])([H])C([H])([H])[H])=NC=12)N4C([H])([H])C([H])([H])N(C([H])([H])C=3C([H])=C([H])C([H])=C([H])C=3[H])C([H])([H])C4([H])[H] | Inactive | ChEMBL database |
| **CHEMBL408** | O=C1[S][C@@]([H])(C(=O)N1[H])C([H])([H])C=4C([H])=C([H])C(OC([H])([H])[C@@]3(OC2=C(/C(=C(/O[H])C(=C2C([H])([H])C3([H])[H])C([H])([H])[H])C([H])([H])[H])C([H])([H])[H])C([H])([H])[H])=C([H])C=4[H] | Inactive | ChEMBL database |
| **CHEMBL4105581** | [Br]C=3C([H])=C([H])C(N([H])C1=NC([S]C([H])([H])C([H])([H])C([H])([H])[H])=NC2=C1N=NN2C([H])([H])C([H])([H])O[H])=C([H])C=3[H] | Inactive | ChEMBL database |

**Table SM11**: ML Testing list compounds

| Compound ID | SMILES | Activity class | Source |
|---|---|---|---|
| **CHEMBL595** | O=C1[S][C@]([H])(C(=O)N1[H])C([H])([H])C=3C([H])=C([H])C(OC([H])([H])C([H])([H])C2=NC([H])=C(C([H])=C2[H])C([H])([H])C([H])([H])[H])=C([H])C=3[H] | Inactive | ChEMBL database |
| **ML-508** | O=C(N1C([H])([H])C([H])([H])C([H])(N(C([H])([H])[H])C([H])([H])[H])C([H])([H])C1([H])[H])C([H])([H])N(/C2=C(\[H])C([H])=C(C#N)C([H])=C2[H])[S](=O)(=O)C3=C([H])C([H])=C(C([H])=C3[H])C([H])([H])[H] | Moderate | 33 |
| **ML-509** | O=C(N1C([H])([H])C([H])([H])C([H])([H])[C@@]([H])(N([H])[H])C1([H])[H])C([H])([H])N(/C3=C(\[H])C=2C(=NN(C=2[H])C([H])([H])[H])C([H])=C3[H])[S](=O)(=O)C4=C([H])C([H])=C(C#N)C([H])=C4[H] | Moderate | 33 |
| **ML-510** | O=C(N1C([H])([H])C([H])([H])[C@@]([H])(N([H])[H])C([H])([H])C1([H])[H])C([H])([H])N(/C2=C(\[H])C([H])=C(C(OC([H])([H])[H])=C2[H])N3N=C(C([H])=C3C([H])([H])[H])C([H])([H])[H])[S](=O)(=O)C4=C([H])C5=C(C([H])=C4[H])N(C(=O)C([H])([H])[H])C([H])([H])C5([H])[H] | Moderate | 33 |
| **ML-511** | O=C(OC(C([H])([H])[H])(C([H])([H])[H])C([H])([H])[H])N4C([H])([H])C([H])([H])N(C(=[S])[S]C([H])([H])C=1N=NN(C=1[H])C([H])([H])C3=C([H])C=2C([H])=C([H])C(O[H])=C([H])C=2OC3=O)C([H])([H])C4([H])[H] | Moderate | 27 |
| **ML-512** | O=C(OC(C([H])([H])[H])(C([H])([H])[H])C([H])([H])[H])N4C([H])([H])C([H])([H])N(C(=[S])[S]C([H])([H])C=1N=NN(C=1[H])C([H])([H])C3=C([H])C(=O)OC=2C([H])=C([F])C([H])=C([H])C=23)C([H])([H])C4([H])[H] | Moderate | 27 |
| **ML-513** | [F]C([F])([F])C1=C([H])C([H])=C(C([H])=C1[H])C([H])([H])[S]C=3N=C2N=C(C([H])=C(N2N=3)N([H])C4=C([H])C([H])=C(C([H])=C4[H])N5C([H])([H])C([H])([H])N(C([H])([H])C5([H])[H])C([H])([H])[H])C([H])([H])[H] | Moderate | 19 |
| **ML-514** | [S]=C([S]C([H])([H])C=1N=NN(C=1[H])C([H])([H])C=2C([H])=C([H])C(OC([H])([H])[H])=C([H])C=2[H])N3C([H])([H])C([H])([H])N([H])C([H])([H])C3([H])[H] | Moderate | 28 |
| **ML-515** | [F]C=1C([H])=C([H])C([H])=C([H])C=1C([H])([H])N2N=N/C(=C2/[H])C([H])([H])[S]C(=[S])N4C([H])([H])C([H])([H])N(C(=O)OC([H])([H])C=3C([H])=C([H])C([H])=C([H])C=3[H])C([H])([H])C4([H])[H] | Moderate | 28 |
| **ML-516** | [F]C=1C([H])=C([H])C(=C([H])C=1[H])C([H])([H])N2N=N/C(=C2/[H])C([H])([H])[S]C(=[S])N4C([H])([H])C([H])([H])N(C(=O)OC([H])([H])C=3C([H])=C([H])C([H])=C([H])C=3[H])C([H])([H])C4([H])[H] | Moderate | 28 |
| **ML-517** | O=[N+]([O-])C1=C([H])C([H])=C(C([H])=C1[H])C([H])([H])[S]C=3N=C2N=C(C([H])=C(N2N=3)N([H])N([H])[H])C([H])([H])[H] | Moderate | 31 |

**Table SM11**: ML Testing list compounds

| Compound ID | SMILES | Activity class | Source |
|---|---|---|---|
| **ML-518** | [Cl]C=1C([H])=C([H])C([H])=C([H])C=1C([H])([H])N2N=N/C(=C2/[H])C([H])([H])[S]C(=[S])N4C([H])([H])C([H])([H])N(C(=O)OC([H])([H])C=3C([H])=C([H])C([H])=C([H])C=3[H])C([H])([H])C4([H])[H] | Moderate | 28 |
| **ML-519** | [S]=C([S]C([H])([H])C=1N=NN(C=1[H])C([H])([H])C=2C([H])=C([H])C(OC([H])([H])[H])=C([H])C=2[H])N4C([H])([H])C([H])([H])N(C(=O)OC([H])([H])C=3C([H])=C([H])C([H])=C([H])C=3[H])C([H])([H])C4([H])[H] | Moderate | 28 |
| **ML-520** | [Cl]C=1C([H])=C([H])C(=C([H])C=1[Cl])C([H])([H])N2N=N/C(=C2/[H])C([H])([H])[S]C(=[S])N4C([H])([H])C([H])([H])N(C(=O)OC([H])([H])C=3C([H])=C([H])C([H])=C([H])C=3[H])C([H])([H])C4([H])[H] | Moderate | 28 |
| **ML-521** | [S]=C([S]C([H])([H])C=1N=NN(C=1[H])C([H])([H])C2=C([H])C(OC([H])([H])[H])=C(OC([H])([H])[H])C(OC([H])([H])[H])=C2[H])N4C([H])([H])C([H])([H])N(C(=O)OC([H])([H])C=3C([H])=C([H])C([H])=C([H])C=3[H])C([H])([H])C4([H])[H] | Moderate | 28 |
| **ML-522** | O=C(OC(C([H])([H])[H])(C([H])([H])[H])C([H])([H])[H])N3C([H])([H])C([H])([H])N(C(=[S])[S]C([H])([H])C=1N=NN(C=1[H])C([H])([H])C=2C([H])=C([H])C([F])=C([H])C=2[H])C([H])([H])C3([H])[H] | Moderate | 28 |
| **ML-523** | O=C(OC(C([H])([H])[H])(C([H])([H])[H])C([H])([H])[H])N3C([H])([H])C([H])([H])N(C(=[S])[S]C([H])([H])C=1N=NN(C=1[H])C([H])([H])C=2C([H])=C([H])C([Cl])=C([H])C=2[H])C([H])([H])C3([H])[H] | Moderate | 28 |
| **ML-524** | [S]=C([S]C([H])([H])C=1N=NN(C=1[H])C([H])([H])C=2C([H])=C([H])C(OC([H])([H])[H])=C([H])C=2[H])N3C([H])([H])C([H])([H])N(C(=O)OC(C([H])([H])[H])(C([H])([H])[H])C([H])([H])[H])C([H])([H])C3([H])[H] | Moderate | 28 |
| **ML-525** | O=C(OC(C([H])([H])[H])(C([H])([H])[H])C([H])([H])[H])N3C([H])([H])C([H])([H])N(C(=[S])[S]C([H])([H])C=1N=NN(C=1[H])C([H])([H])C=2C([H])=C([H])C([Cl])=C([Cl])C=2[H])C([H])([H])C3([H])[H] | Moderate | 28 |
| **ML-526** | [S]=C([S]C([H])([H])C=1N=NN(C=1[H])C([H])([H])C2=C([H])C(OC([H])([H])[H])=C(OC([H])([H])[H])C(OC([H])([H])[H])=C2[H])N3C([H])([H])C([H])([H])N(C(=O)OC(C([H])([H])[H])(C([H])([H])[H])C([H])([H])[H])C([H])([H])C3([H])[H] | Moderate | 28 |
| **ML-527** | [S]=C([S]C([H])([H])C=1N=NN(C=1[H])C([H])([H])C2=C([H])C([H])=C([H])C(=C2[H])C([H])([H])[H])N3C([H])([H])C([H])([H])N(C(=O)OC(C([H])([H])[H])(C([H])([H])[H])C([H])([H])[H])C([H])([H])C3([H])[H] | Moderate | 28 |
| **ML-528** | N#C/C3=C(\[H])C([H])=C(N1N=C(C([H])=C1O[C@@]([H])(C(=O)N([H])[H])C([H])([H])[H])C(=O)N2C([H])([H])[C@]([H])(N([H])[H])C([H])([H])C([H])([H])C2([H])[H])C([H])=C3[H] | Moderate | 34 |

| **Table SM11**: ML Testing list compounds | | | |
|---|---|---|---|
| **Compound ID** | **SMILES** | **Activity class** | **Source** |
| **ML-529** | O=C(OC(C([H])([H])[H])(C([H])([H])[H])C([H])([H])[H])N3C([H])([H])C([H])([H])N(C(=[S])[S]C([H])([H])C=1N=NN(C=1[H])C([H])([H])C=2C([H])=C([H])C(O[H])=C([H])C=2[H])C([H])([H])C3([H])[H] | Moderate | 28 |
| **ML-530** | O=C(OC(C([H])([H])[H])(C([H])([H])[H])C([H])([H])[H])N3C([H])([H])C([H])([H])N(C(=[S])[S]C([H])([H])C=1N=NN(C=1[H])C([H])([H])C=2C([H])=C([H])C([H])=C([H])C=2O[H])C([H])([H])C3([H])[H] | Moderate | 28 |
| **ML-531** | O=C(OC(C([H])([H])[H])(C([H])([H])[H])C([H])([H])[H])N3C([H])([H])C([H])([H])N(C(=[S])[S]C([H])([H])C=1N=NN(C=1[H])C([H])([H])C=2C([H])=C([H])C([H])=C(O[H])C=2[H])C([H])([H])C3([H])[H] | Moderate | 28 |
| **ML-532** | O=C(OC(C([H])([H])[H])(C([H])([H])[H])C([H])([H])[H])N3C([H])([H])C([H])([H])N(C(=[S])[S]C([H])([H])C=1N=NN(C=1[H])C([H])([H])C=2C([H])=C([H])C(O[H])=C([H])C=2O[H])C([H])([H])C3([H])[H] | Moderate | 28 |
| **ML-533** | [H]C=2C(=NC1=NC(=NN1C=2N([H])N([H])[H])[S]C([H])([H])C3=C([H])C([H])=C(OC([H])([H])[H])C([H])=C3[H])C([H])([H])[H] | Moderate | 31 |
| **ML-534** | O=C(OC(C([H])([H])[H])(C([H])([H])[H])C([H])([H])[H])N3C([H])([H])C([H])([H])N(C(=[S])[S]C([H])([H])C=1N=NN(C=1[H])C([H])([H])C=2C([F])=C([H])C([H])=C([H])C=2[F])C([H])([H])C3([H])[H] | Moderate | 28 |
| **ML-535** | [S]=C([S]C([H])([H])C=1N=NN(C=1[H])C([H])([H])C=2C([H])=C([H])C(=C([H])C=2[H])C([H])([H])[H])N3C([H])([H])C([H])([H])N(C([H])([H])C([H])([H])O[H])C([H])([H])C3([H])[H] | Moderate | 28 |
| **ML-536** | [S]=C([S]C([H])([H])C=1N=NN(C=1[H])C([H])([H])C=2C([H])=C([H])C([H])=C([H])C=2[F])N3C([H])([H])C([H])([H])N(C([H])([H])C([H])([H])O[H])C([H])([H])C3([H])[H] | Moderate | 28 |
| **ML-537** | [S]=C([S]C([H])([H])C=1N=NN(C=1[H])C([H])([H])C=2C([H])=C([H])C(=C([H])C=2[H])C([H])([H])[H])N4C([H])([H])C([H])([H])N(C=3C([H])=C([H])C([H])=C([H])C=3[H])C([H])([H])C4([H])[H] | Moderate | 28 |
| **ML-538** | [F]C=1C([H])=C([H])C(=C([H])C=1[H])C([H])([H])N2N=N/C(=C2/[H])C([H])([H])[S]C(=[S])N4C([H])([H])C([H])([H])N(C=3/N=C(/[H])C([H])=C([H])N=3)C([H])([H])C4([H])[H] | Moderate | 28 |
| **ML-539** | O=[S](=O)(N3C([H])([H])C([H])([H])N(C(=[S])[S]C([H])([H])C=1N=NN(C=1[H])C([H])([H])C=2C([H])=C([H])C([H])=C([H])C=2[F])C([H])([H])C3([H])[H])C([H])([H])[H] | Moderate | 28 |
| **ML-540** | O=[S](=O)(N3C([H])([H])C([H])([H])N(C(=[S])[S]C([H])([H])C=1N=NN(C=1[H])C([H])([H])C=2C([H])=C([H])C([F])=C([H])C=2[H])C([H])([H])C3([H])[H])C([H])([H])[H] | Moderate | 28 |

**Table SM11**: ML Testing list compounds

| Compound ID | SMILES | Activity class | Source |
|---|---|---|---|
| **ML-541** | [F]C([F])([F])C=1C([H])=C([H])C(=C([H])C=1[H])C([H])([H])N2N=N/C(=C2/[H])C([H])([H])[S]C(=[S])N3C([H])([H])C([H])([H])N([H])C([H])([H])C3([H])[H] | Moderate | 28 |
| **ML-542** | O=C(N3C([H])([H])C([H])([H])N(C(=[S])[S]C([H])([H])C=1N=NN(C=1[H])C([H])([H])C=2C([H])=C([H])C([H])=C([H])C=2[F])C([H])([H])C3([H])[H])C([H])([H])[H] | Moderate | 28 |
| **ML-543** | O=C(N3C([H])([H])C([H])([H])N(C(=[S])[S]C([H])([H])C=1N=NN(C=1[H])C([H])([H])C=2C([H])=C([H])C([F])=C([H])C=2[H])C([H])([H])C3([H])[H])C([H])([H])[H] | Moderate | 28 |
| **ML-544** | [H]C([H])([H])C=1C([H])=C([H])C(=C([H])C=1[H])C=3/N=C(/[H])C(OC([H])([H])C2([H])C([H])([H])C([H])([H])N([H])C([H])([H])C2([H])[H])=C([H])C=3C=4C([H])=C([H])C(=C([H])C=4[H])C([H])([H])N5C([H])([H])C([H])([H])C([H])([H])C([H])([H])C5([H])[H] | Moderate | 25 |
| **ML-545** | [Cl]/C2=C(\[H])C=1/C(=C(/[H])[S]C=1C([H])=C2[H])C([H])([H])[S]C=4N=C3N=C(C([H])=C(N3N=4)N([H])C5=C([H])C([H])=C(C([H])=C5[H])N6C([H])([H])C([H])([H])N(C([H])([H])C6([H])[H])C([H])([H])[H])C([H])([H])[H] | Moderate | 19 |
| **ML-546** | [O-][N+](=O)C=1C([H])=C([H])C([H])=C([H])C=1[S](=O)(=O)N([H])C2=N/C(=C(/[H])[S]2)C=3C([H])=C([H])C([Cl])=C([H])C=3[H] | Moderate | 30 |
| **ML-547** | [O-][N+](=O)C=1C([H])=C([H])C([H])=C(C=1[H])[S](=O)(=O)N([H])C2=N/C(=C(/[H])[S]2)C=3C([H])=C([H])C([Cl])=C([H])C=3[H] | Moderate | 30 |
| **ML-548** | [O-][N+](=O)C=1C([H])=C([H])C(=C([H])C=1[H])[S](=O)(=O)N([H])C2=N/C(=C(/[H])[S]2)C=3C([H])=C([H])C([Cl])=C([H])C=3[H] | Moderate | 30 |

**Table SM11**: ML Testing list compounds



| Compound ID | SMILES | Activity class | Source |
|---|---|---|---|
| **ML-549** | [O-][N+](=O)C=1C([H])=C([H])C([H])=C([H])C=1C=2N=C([S]C=2[H])N([H])[S](=O)(=O)C=3C([H])=C([H])C([H])=C([Cl])C=3[H] | Moderate | 30 |
| **ML-550** | [O-][N+](=O)C=1C([H])=C([H])C([H])=C(C=1[H])C=2N=C([S]C=2[H])N([H])[S](=O)(=O)C=3C([H])=C([H])C([H])=C([Cl])C=3[H] | Moderate | 30 |
| **ML-551** | [O-][N+](=O)C=1C([H])=C([H])C(=C([H])C=1[H])C=2N=C([S]C=2[H])N([H])[S](=O)(=O)C=3C([H])=C([H])C([H])=C([Cl])C=3[H] | Moderate | 30 |
| **ML-552** | O=C(OC([H])([H])C([H])([H])[H])C=1C([H])=C([H])C([H])=C(C=1[H])C=2N=C([S]C=2[H])N([H])[S](=O)(=O)C=3C([H])=C([H])C([H])=C([Cl])C=3[H] | Moderate | 30 |
| **ML-553** | O=C(OC(C([H])([H])[H])(C([H])([H])[H])C([H])([H])[H])N([H])[C@@]4([H])C([H])([H])C([H])([H])C([H])([H])N(/C2=C(\[H])C(=NC1=NC(=NN12)[S]C([H])([H])C3=C([H])C([H])=C([H])C([H])=C3[H])C([H])([H])[H])C4([H])[H] | Moderate | 31 |
| **ML-554** | O=C(/C2=C(\[H])C=1[S]C([H])=C([H])C=1N2C([H])([H])[H])N([H])C3=C(C([H])=C([H])C([H])=C3[H])C([H])([H])O/C5=C(\OC4([H])C([H])([H])C([H])([H])N(C([H])([H])C4([H])[H])C([H])([H])[H])C([H])=C([H])C([H])=C5[H] | Moderate | 24 |
| **ML-555** | O=C3N([H])C(=O)N(C=2/N=C(/[S]C([H])([H])C1=C([H])C([H])=C(C([H])=C1[H])C(C([H])([H])[H])(C([H])([H])[H])C([H])([H])[H])N(C=23)C([H])([H])C([H])([H])C([H])([H])[H])C([H])([H])[H] | Moderate | 32 |
| **ML-556** | O=C3N([H])C(=O)N(C=2/N=C(/[S]C([H])([H])C(=O)N([H])C1=C([H])C([H])=C(C([H])=C1[Cl])[N+](=O)[O-])N(C=23)C([H])([H])C([H])([H])C([H])([H])[H])C([H])([H])[H] | Moderate | 32 |

**Table SM11**: ML Testing list compounds

| Compound ID | SMILES | Activity class | Source |
|---|---|---|---|
| **ML-557** | O=C(/C1=C(\[H])C([H])=C([H])C(=C1[H])C([H])([H])[C@]4([H])C([H])([H])N(C(=O)C2=NC3=C(/C(=C2/[H])C([H])([H])[H])C(=O)N([H])N3[H])C([H])([H])C4([H])[H])N([H])[H] | Moderate | 31 |
| **ML-558** | O=C(/C1=C(\[H])C([H])=C([H])C([H])=C1[H])C([H])([H])[S]C=3N=C2N=C(C([H])=C(N2N=3)N([H])N([H])[H])C([H])([H])[H] | Moderate | 31 |
| **ML-559** | [Cl]C=1C([H])=C([H])C(=C([H])C=1[Cl])C([H])([H])[S]/C3=N/C(/C2=C(\[H])C(OC([H])([H])[H])=C(OC([H])([H])[H])C(OC([H])([H])[H])=C2[H])=C(C#N)C(=N3)N([H])N([H])C(=[S])N([H])[H] | Moderate | 18 |
| **ML-560** | O=C(N([H])C1=C([H])C([H])=C([H])C(=C1C([H])([H])O/C3=C(\[H])C([H])=C(OC2([H])C([H])([H])C([H])([H])N([H])C([H])([H])C2([H])[H])C([H])=C3[H])C([H])([H])OC([H])([H])[H])C=4OC=5C(C=4[H])=C([H])C([H])=C([H])C=5[H] | Moderate | 26 |
| **ML-561** | O=C(N([H])C1=C([H])C([H])=C([H])C(=C1C([H])([H])O/C3=C(\[H])C([H])=C(OC2([H])C([H])([H])C([H])([H])N([H])C([H])([H])C2([H])[H])C([H])=C3[H])C([H])([H])OC([H])([H])[H])C5=C([H])C([H])=C4OC([H])([H])O/C4=C5\[H] | Moderate | 26 |
| **ML-562** | O=C(N([H])C1=C([H])C([H])=C([H])C(=C1C([H])([H])O/C3=C(\[H])C([H])=C(OC2([H])C([H])([H])C([H])([H])N([H])C([H])([H])C2([H])[H])C([H])=C3[H])C([H])([H])OC([H])([H])[H])C4=C([H])C([H])=C([H])C([H])=C4[H] | Moderate | 26 |
| **ML-563** | O=C(N([H])C1=C([H])C([H])=C([H])C(=C1C([H])([H])O/C3=C(\[H])C([H])=C(OC2([H])C([H])([H])C([H])([H])N([H])C([H])([H])C2([H])[H])C([H])=C3[H])C([H])([H])OC([H])([H])[H])C4=C([H])C([H])=C5/C(=C4/[H])C([H])=C([H])C([H])=C5[H] | Moderate | 26 |
| **ML-564** | O=C(N([H])[C@@]1([H])C([H])([H])N2C([H])([H])C([H])([H])C1([H])C([H])([H])C2([H])[H])C([H])([H])N(/C3=C(\[H])C([H])=C(C#N)C([H])=C3[H])[S](=O)(=O)C4=C([H])C([H])=C(C([H])=C4[H])C([H])([H])[H] | Moderate | 33 |
| **ML-565** | O=C(N1[C@@]2([H])C([H])([H])N([H])[C@@]([H])(C1([H])[H])C2([H])[H])C([H])([H])N(/C3=C(\[H])C([H])=C(C#N)C([H])=C3[H])[S](=O)(=O)C4=C([H])C([H])=C(C([H])=C4[H])C([H])([H])[H] | Moderate | 33 |

| Table SM11: ML Testing list compounds | | | |
|---|---|---|---|
| **Compound ID** | **SMILES** | **Activity class** | **Source** |
| **ML-566** | O=C(N1C([H])([H])C([H])([H])C([H])([H])[C@@]([H])(N([H])[H])C1([H])[H])C([H])([H])N(/C2=C(\[H])C([H])=C(C#N)C([H])=C2[H])[S](=O)(=O)C3=C([H])C([H])=C(C([H])=C3[H])C([H])([H])[H] | Moderate | 33 |
| **ML-567** | O=C(N1C([H])([H])C([H])([H])C([H])(N([H])[H])C([H])([H])C1([H])[H])C([H])([H])N(/C2=C(\[H])C([H])=C(C#N)C([H])=C2[H])[S](=O)(=O)C3=C([H])C([H])=C(C([H])=C3[H])C([H])([H])[H] | Moderate | 33 |
| **ML-568** | O=C(N([H])C1([H])C([H])([H])C([H])([H])N([H])C([H])([H])C1([H])[H])C([H])([H])N(/C2=C(\[H])C([H])=C(C#N)C([H])=C2[H])[S](=O)(=O)C3=C([H])C([H])=C(C([H])=C3[H])C([H])([H])[H] | Moderate | 33 |
| **ML-569** | O=C(N1C([H])([H])C([H])([H])[C@]([H])(N([H])[H])C1([H])[H])C([H])([H])N(/C2=C(\[H])C([H])=C(C#N)C([H])=C2[H])[S](=O)(=O)C3=C([H])C([H])=C(C([H])=C3[H])C([H])([H])[H] | Moderate | 33 |
| **ML-570** | O=C(N1C([H])([H])C([H])([H])C([H])(N([H])C([H])([H])[H])C([H])([H])C1([H])[H])C([H])([H])N(/C2=C(\[H])C([H])=C(C#N)C([H])=C2[H])[S](=O)(=O)C3=C([H])C([H])=C(C([H])=C3[H])C([H])([H])[H] | Moderate | 33 |
| **CHEMBL3786232** | O=C(O[H])C([H])=C(C([H])([H])C([H])([H])C([H])=C(C([H])([H])C([H])([H])C([H])=C(C([H])([H])C([H])([H])C([H])([H])C([H])(C([H])([H])[H])C([H])([H])[H])C([H])([H])[H])C([H])([H])[H])C([H])([H])[H] | Moderate | ChEMBL database |
| **CHEMBL3786908** | [Cl]C=1C([H])=C([H])C(O[H])=C(C=1[H])N([H])C3=N/C2=C(\[H])C(=C([H])C([H])=C2N3[H])[S](=O)(=O)N4C([H])([H])C([H])([H])OC([H])([H])C4([H])[H] | Moderate | ChEMBL database |
| **CHEMBL4060712** | [H]N([C@]3([H])N2/N=C(/[S]C([H])([H])C=1C([H])=C([H])C([H])=C([H])C=1[H])N=C2N([H])[C@]([H])(C([H])([H])[H])C3([H])[H])C([H])([H])C([H])([H])C([H])([H])N4C([H])([H])C([H])([H])OC([H])([H])C4([H])[H] | Moderate | ChEMBL database |
| **CHEMBL4060820** | [H]N1C(=N/C2=C(\[H])C([H])=C([H])C([H])=C12)C([H])([H])[S]C=5N=C4N([H])[C@]([H])(C([H])([H])[C@@]([H])(N([H])C3=C([H])C(OC([H])([H])[H])=C(OC([H])([H])[H])C(OC([H])([H])[H])=C3[H])N4N=5)C([H])([H])[H] | Moderate | ChEMBL database |
| **CHEMBL4061895** | O=C6C=5C([H])=C([H])C([H])=C(N([H])[C@@]4([H])N3/N=C(/[S]C([H])([H])C2=N/C1=C(\[H])C([H])=C([H])C([H])=C1N2[H])N=C3N([H])[C@@]([H])(C([H])([H])[H])C4([H])[H])C=5C([H])([H])N6[C@@]7([H])C(=O)N([H])C(=O)C([H])([H])C7([H])[H] | Moderate | ChEMBL database |

**Table SM11**: ML Testing list compounds

| Compound ID | SMILES | Activity class | Source |
|---|---|---|---|
| **CHEMBL4065645** | [H]OC([H])([H])C([H])([H])N4C([H])([H])C([H])([H])N([C@]3([H])N2/N=C(/[S]C([H])([H])C=1C([H])=C([H])C([H])=C([H])C=1[H])N=C2N([H])[C@]([H])(C([H])([H])[H])C3([H])[H])C([H])([H])C4([H])[H] | Moderate | ChEMBL database |
| **CHEMBL4069335** | [H]N([C@@]3([H])N2/N=C(/[S]C([H])([H])C=1C([H])=C([H])C([H])=C([H])C=1[H])N=C2N([H])[C@@]([H])(C([H])([H])[H])C3([H])[H])C([H])([H])C([H])([H])C([H])([H])O[H] | Moderate | ChEMBL database |
| **CHEMBL4080060** | [H]N([C@]3([H])N2/N=C(/[S]C([H])([H])C=1C([H])=C([H])C([H])=C([H])C=1[H])N=C2N([H])[C@]([H])(C([H])([H])[H])C3([H])[H])C([H])([H])C([H])([H])C([H])([H])N4C([H])=C([H])N=C4[H] | Moderate | ChEMBL database |
| **CHEMBL4083660** | O=C6N([H])C(=O)[C@@](C=5C([H])=C([H])C(N([H])[C@]2([H])N1N=C(N=C1N([H])[C@@]([H])(C2([H])[H])C([H])([H])[H])[S]C([H])([H])C4=N/C3=C(\[H])C([H])=C([H])C([H])=C3N4[H])=C([H])C=5[H])(C([H])([H])C([H])([H])[H])C([H])([H])C6([H])[H] | Moderate | ChEMBL database |
| **CHEMBL4094062** | [H]N(/C1=C(\[H])C(OC([H])([H])[H])=C(OC([H])([H])[H])C(OC([H])([H])[H])=C1[H])[C@@]3([H])N2N=C(N=C2N([H])[C@]([H])(C3([H])[H])C([H])([H])[H])[S]C([H])([H])C=4C([H])=C([H])C([H])=C([H])C=4[H] | Moderate | ChEMBL database |
